# polars-bio—fast, scalable, and out-of-core operations on large genomic interval datasets

**DOI:** 10.1093/bioinformatics/btaf640

**Published:** 2025-12-01

**Authors:** Marek Wiewiórka, Pavel Khamutou, Marek Zbysiński, Tomasz Gambin

**Affiliations:** Institute of Computer Science, Warsaw University of Technology, 00-665 Warsaw, Poland; Institute of Computer Science, Warsaw University of Technology, 00-665 Warsaw, Poland; Department of Computer Science, University of Oxford, OX1 3QD Oxford, United Kingdom; Institute of Computer Science, Warsaw University of Technology, 00-665 Warsaw, Poland

## Abstract

**Motivation:**

Genomic studies very often rely on computationally intensive analyses of relationships between features, which are typically represented as intervals along a 1D coordinate system (such as positions on a chromosome). In this context, the Python programming language is extensively used for manipulating and analyzing data stored in a tabular form of rows and columns, called a DataFrame. Pandas is the most widely used Python DataFrame package and has been criticized for inefficiencies and scalability issues, which its modern alternative—Polars—aims to address with a native backend written in the Rust programming language.

**Results:**

*polars-bio* is a Python library that enables fast, parallel and out-of-core operations on large genomic interval datasets. Its main components are implemented in Rust, using the Apache DataFusion query engine and Apache Arrow for efficient data representation. It is compatible with Polars and Pandas DataFrame formats. In a real-world comparison ($10^{7}$ versus $1.2\times 10^{6}$ intervals), our library runs *overlap* queries 6.5×, *nearest* queries 15.5×, *count_overlaps* queries 38×, and *coverage* queries 15× faster than Bioframe. On equally sized synthetic sets ($10^{7}$ versus $10^{7}$), the corresponding speedups are 1.6×, 5.5×, 6×, and 6×. In streaming mode, on real and synthetic interval pairs, our implementation uses 90× and 15× less memory for *overlap*, 4.5× and 6.5× less for *nearest*, 60× and 12× less for *count_overlaps*, and 34× and 7× less for *coverage* than Bioframe. Multi-threaded benchmarks show good scalability characteristics. To the best of our knowledge, *polars-bio* is the most efficient single-node library for genomic interval DataFrames in Python.

**Availability and implementation:**

*polars-bio* is an open-source Python package distributed under the Apache License available for major platforms, including Linux, macOS, and Windows in the PyPI registry. The online documentation is https://biodatageeks.org/polars-bio/ and the source code is available on GitHub: https://github.com/biodatageeks/polars-bio and Zenodo: https://doi.org/10.5281/zenodo.16374290. are available at *Bioinformatics* online.

## 1 Introduction

Operations on genomic intervals (ranges), such as finding intervals that overlap with or are closest to a given interval, are fundamental to many bioinformatics analyses, including the process of annotating genetic variants (McLaren *et al.* 2016). Dedicated efficient data structures and/or accompanying command-line interface tools, such as NCList (Alekseyenko and Lee 2007), AIList (Feng *et al.* 2019), Bedtk (Li and Rong 2021), or Coitrees (https://crates.io/crates/coitrees) have been proposed. In addition, the growing popularity of the Python programming language for data analysis has resulted in the emergence of libraries, such as Pybedtools (Dale *et al.* 2011), a Python port of GenomicRanges (Lawrence *et al.* 2013), or more recently PyRanges (Stovner and Sætrom 2020) and Bioframe (Abdennur *et al.* 2024). The existing approaches (see Section 3.5, available as supplementary data at *Bioinformatics* online “Comparison of the libraries” of the Supplementary Materials), however, suffer from one or more problems that include:

- not adhering to standardized Python data formats, such as Pandas/Polars DataFrames and widely adopted efficient data representation formats such as Apache Arrow (https://arrow.apache.org/),
- lacking vectorized, parallel and out-of-core execution engines resulting in suboptimal performance,
- missing capability of streaming processing and federated queries of large-scale datasets directly from cloud storage services without the need to download them first. In many cases this approach might not be feasible due to dataset sizes that can exceed both a computer’s main memory and the capacity of locally attached drives. Such a functionality is beneficial as many research projects share their datasets using services like Amazon Simple Storage Service (S3) or Google Cloud Storage (GCS).

To address the aforementioned challenges, we present *polars-bio*, a Python library that combines the strengths of Apache DataFusion (Lamb *et al.* 2024)—extensible, columnar, streaming, multi-threaded, vectorized execution engine, Apache Arrow—columnar memory format enabling efficient data representation and exchange coupled with the user-friendly and fast Polars (Oketunji 2024) library.

## 2 Methods

### 2.1 Architecture and implementation

**Figure 1.**
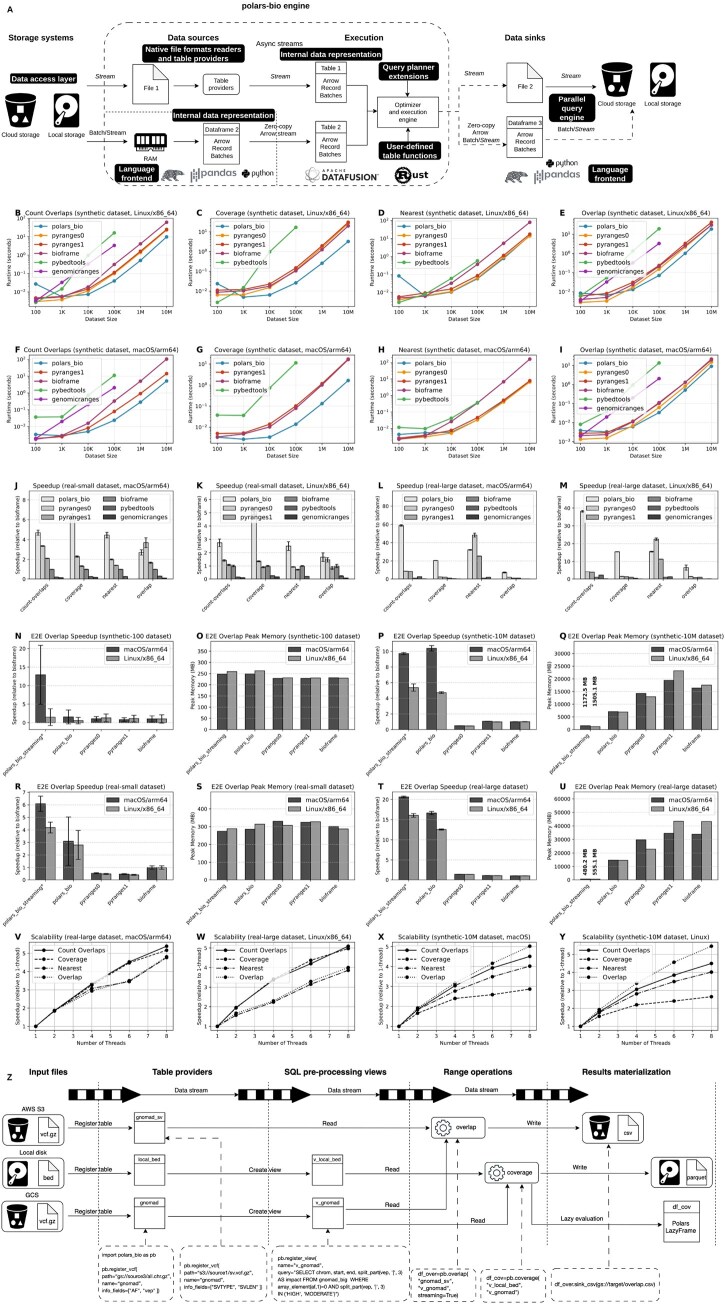
Panel A shows the main components of the *polars-bio* architecture (see Section 2.1 for details). Panels B–I show performance characteristics for the synthetic-dataset benchmarks of *count_overlaps*, *coverage*, *nearest*, and *overlap* operations (single thread, $10^{2}-10^{7}$ intervals) for macOS/arm64 and Linux/x86-64. Panels J–M display speedup (relative to Bioframe, single thread) on small and large real-world datasets for macOS/arm64 and Linux/x86-64. Panels N–U show performance results (speedup and peak-memory usage) for end-to-end processing of the overlap operation on real-world and synthetic datasets, *In *polars_bio_streaming*, despite limiting the number of threads in DataFusion and Polars, CPU utilization remained approximately 160%. Panels V–Y present scalability results for *polars-bio* (scalability was evaluated as speedup versus the number of threads). Panel Z shows an example of a practical use case scenario (see Section 2.2 for details).

When designing *polars-bio* we took inspiration from the recently proposed paradigm of Composable Data Management Systems (Pedreira *et al.* 2023) and built it upon modular and reusable components to achieve standardization of the **language frontend** [Structured Query Language (SQL) and DataFrames], extensibility of **query optimization** and high performance of the **execution engine**. From the data processing perspective, we adopted **lazy evaluation**, **asynchronous**, **streaming** (out-of-core) and **parallel** programming patterns to enable large-scale computations and resource-usage efficiency. *polars-bio* comprises the following elements (Fig. 1A):

- **Data access layer—**in order to provide a unified access to network storage systems (public cloud object storage, e.g. S3 or GCS) the Apache OpenDAL project is used as an abstraction layer for data streams over the existing cloud-specific access methods.
- **Language frontend—**by adhering to the popular Polars and Pandas APIs, users can easily plug in *polars-bio* into the existing Python bioinformatics analysis workflows that rely on the aforementioned DataFrame interfaces. Additionally, the SQL API can be used for implementing efficient data pre-processing steps.
- **Native file-format readers and table providers—**Apache DataFusion has built-in support for popular file formats, such as comma-separated values (CSV) and Parquet. For bioinformatic file formats, such as Variant Call Format for variants (VCF) or Browser Extensible Data (BED), custom table providers based on the noodles (https://crates.io/crates/noodles) library have been developed. They are also implemented in an asynchronous streaming fashion.
- **Internal data representation—**Apache Arrow is used as a columnar memory model to facilitate efficient (in most cases, zero-copy) data exchange between components in the form of streams of record batches (i.e. arrays of array references representing typed columns with Arrow C data/stream interfaces). It is feasible because both Pandas and Polars also use Arrow-compatible memory layouts.
- **Query planner extensions—**a set of custom physical optimization rules for interval operations have been added to detect and override default operators (based on hash or nested-loop join algorithms), so that our more efficient, interval-tree-based methods are used instead.
- **User-defined table functions—**for range operations, such as *coverage* or *count_overlaps*, that cannot be efficiently expressed with either DataFusion DataFrame or SQL application programming interfaces (APIs).
- **Parallel query engine—**Apache DataFusion was selected due to its great extensibility (e.g. user-defined functions/table functions, custom table providers, logical and physical plan optimization rules or execution plan operators), high efficiency (based on asynchronous, vectorized, parallel and out-of-core execution model of processing streams of partitioned DataFrames represented logically as tables backed by different providers) and, finally, an active community of open-source contributors and companies that embedded it in their specialized data management systems.

*polars-bio* is implemented as a Python package with a Rust backend that is distributed as platform-specific binaries targeting Linux (x86-64), macOS (x86-64 and arm64), and Windows (x86-64) platforms. It is developed using the PyO3 (Flitton 2022) Rust bindings for Python.

### 2.2 Functionality

#### 2.2.1 Supported operations and input formats

*polars-bio* provides a rich set of genomics operations. *overlap* determines which genomic intervals from one dataset overlap those in another. *nearest* takes two sets of intervals (say A and B) and for each interval *a* in A finds the interval in B that either overlaps with *a* or is closest to *a*. It can be used to find the nearest gene to a genetic variant. *count_overlaps* calculates, for each interval in a primary set A, how many intervals from a secondary set B overlap it. It is useful for applications such as counting how many reads (intervals in B) cover each gene (interval in A), or how many regulatory elements fall within each genomic bin. *coverage* calculates the number of base pairs of A covered by intervals in B. Besides, the aforementioned operations support out-of-core processing without requiring *all* your data to be in memory at the same time—only the build (search) side needs to be materialized while the probe side is streamed.

To facilitate real-world genomics workflows, *polars-bio* includes built-in readers for standard genomic file formats, such as BED, GFF3 (for genomic intervals), VCF, BAM, and FASTQ/FASTA. *polars-bio* uses, by default, a 1-based coordinate system for both reading bioinformatic input files and all interval operations, while a 0-based coordinate system is also supported. See the online documentation for methods and their implementation details.

#### 2.2.2 Practical use cases

*polars-bio* exposes an API that works with both Pandas and Polars. Users can call high-level functions (e.g. *overlap* or *count_overlaps*) or use its SQL interface (powered by Apache DataFusion) to register datasets as in-memory table references. A major strength is its support for federated analysis, processing data from multiple sources without central consolidation.

For example, consider: (i) a local BED file of genomic intervals; (ii) $>10^{6}$ structural variants (∼2 GB) from gnomAD (Karczewski *et al.* 2020) stored on GCS; and >$9\times 10^{8}$ SNVs (Single Nucleotide Variants) and INDELs (∼500 GB) on Amazon S3 (see Fig. 1Z).

Traditionally, one would need to download these files or use a distributed query engine. With *polars-bio*, one can register the remote files as tables and: (i) parse specific columns (e.g. extract the IMPACT value from Variant Effect Predictor annotations); (ii) filter variants via SQL to select those that are rare and have HIGH or MODERATE impact; (iii) overlap the filtered variants with structural variants. Reading, preprocessing and overlap operations are executed in streaming mode: *polars-bio* reads chunks from the remote files, transforms and filters them, finds overlaps and writes results (to CSV or Parquet) without ever loading the full 500 GB into memory. One can also easily count overlaps between a set of genomic intervals defined in a local BED file and remote variant dataset to determine the number of variants in each genomic region.

## 3 Performance

*polars-bio* has been carefully benchmarked for speed, scalability and memory consumption in two runtime environments: Linux with x86-64 and macOS with arm64 architectures. Two benchmarking datasets were used: a real-world dataset from the AIList (Feng *et al.* 2019) manuscript as well as a synthetic dataset proposed in the Bioframe (Abdennur *et al.* 2024) manuscript. In single-threaded mode the following Python libraries were compared: Bioframe, PyRanges (major versions 0 and 1; the latter is a complete rewrite of the former), Pybedtools and GenomicRanges running four popular operations: *overlap*, *nearest*, *count_overlaps* and *coverage*.

For small to moderate interval counts ($10^{2}$–$10^{5}$), Bioframe, PyRanges, and polars-bio complete typical operations in well under a second, with no meaningful speed differences between them. However, at larger scales ($10^{7}$ intervals), polars-bio consistently outperforms Bioframe—achieving an average speedup of 4.9× to 13.6× depending on the runtime environment—while in the case of PyRanges, the speed difference is less visible, especially for the nearest operation. In contrast, other tools slow dramatically once you exceed about $10^{5}$ intervals (Fig. 1B–I). Additionally, an end-to-end test scenario that encompassed reading from Parquet files, computing interval overlaps and saving results to CSV files was profiled (Fig. 1N–Q). In this benchmark *polars-bio* was run twice: with full results materialization (polars_bio) in RAM prior to saving the output and in streaming mode (polars_bio_streaming). Again, for small interval counts there are no substantial differences in memory consumption—but at larger scales ($10^{7}$ intervals) *polars-bio* can accomplish the operation considerably faster (5–10× depending on the testing environment) with highly reduced peak memory requirements by up to 15× in streaming mode. Scalability benchmark (Fig. 1X and Y) across all four operations reveals good characteristics, with an average speedup of 2.9× at 4 threads and 4.2× at 8 threads ($10^{7}$ intervals).

The real-world benchmark provides even stronger evidence for the efficiency of *polars-bio*: in the case of large dataset ($10^{7}$ versus $1.2\times 10^{6}$ intervals), our library achieves the following speedups over Bioframe: *overlap*—6.5×, *nearest*—15.5×, *count_overlaps*—38×, and *coverage*—15× (Fig. 1J–M). Peak memory consumption for overlap operation in streaming mode is 90× < Bioframe (Fig. 1R–U). Finally, the corresponding average scalability factors are 2.8× and 4.5× (Fig. 1V and W).

See Supplementary Materials for the algorithm description (Section 2, available as supplementary data at *Bioinformatics* online); benchmarking configuration (Section 3.1, available as supplementary data at *Bioinformatics* online); datasets (Section 3.2, available as supplementary data at *Bioinformatics* online); speedup and peak memory: summary statistics and raw data for 50 real-world dataset pairs (Section 3.3.1, available as supplementary data at *Bioinformatics* online); detailed performance results on selected synthetic and real datasets (Sections 3.3.2–3.3.4, available as supplementary data at *Bioinformatics* online); memory profile evaluation (Section 3.4, available as supplementary data at *Bioinformatics* online); and discussion of differences among tested libraries (Section 3.5, available as supplementary data at *Bioinformatics* online).

## 4 Conclusion

*polars-bio* bridges the gap between the efficiency of command-line tools written in natively compiled languages, such as C++ or Rust, and the ergonomics of the Python DataFrame ecosystem. The out-of-core (streaming) feature addresses the growing need for big-data and federated-query capabilities without incurring the complexity of the distributed computing machinery. It enables both *ad hoc* exploratory analyses and recurring data pipelines for small and large datasets to be implemented in a fast, scalable and consistent way.

## Supplementary Material

btaf640_Supplementary_Data

## Data Availability

Links to all datasets used in this study are provided in Section 3.2 of the Supplementary Materials.
