# Supplementary material for: polars-bio—fast, scalable, and out-of-core operations on large genomic interval datasets
Source: Bioinformatics. 2025 Dec 1;41(12):btaf640. doi: 10.1093/bioinformatics/btaf640 (PMC12701801; doi:10.1093/bioinformatics/btaf640)
Supplement: btaf640_Supplementary_Data [file btaf640_supplementary_data.pdf]

# Supplementary Materials of: **polars-bio – fast, scalable and out-of-core operations on large genomic interval datasets**

# Contents


**1 Introduction** **3**

**2 Algorithm description** **3**
2.1 Out-of-core (streaming) processing . . . 4
2.2 Parallelization . . . 4
2.3 Implementation . . . 4

**3 Benchmarks** **4**
3.1 Configuration . . . 4
3.1.1 Code and benchmarking scenarios . . . 4
3.1.2 Memory profiling . . . 4
3.1.3 Operating systems and hardware configurations . . . 5
3.1.3.1 apple-m3-max . . . 5
3.1.3.2 gcp-linux . . . 5
3.1.4 Libraries versions used in benchmarks . . . 5
3.2 Datasets . . . 5
3.2.1 Real dataset . . . 5
3.2.2 Selection of 50 ordered pairs from the real-world dataset for benchmarking . . . 6
3.2.3 Synthetic dataset . . . 7
3.2.4 Overlap detection counts across libraries . . . 8
3.3 Performance evaluation . . . 8
3.3.1 Summary statistics . . . 8
3.3.1.1 Speedup summary statistics . . . 8
3.3.1.2 Memory peak summary statistics . . . 10
3.3.2 Single-thread results . . . 12
3.3.2.1 apple-m3-max . . . 13
3.3.2.2 gcp-linux . . . 19
3.3.3 Multi-thread results . . . 26
3.3.3.1 apple-m3-max . . . 26
3.3.3.2 gcp-linux . . . 29
3.3.4 End to end pipelines results . . . 31
3.3.4.1 apple-m3-max . . . 32
3.3.4.2 gcp-linux . . . 35
3.4 Memory profiles evaluation . . . 39
3.4.1 Operation: overlap for dataset: fBrain-vs-exons on platform: apple-m3-max . . . 40
3.4.2 Operation: nearest for dataset: fBrain-vs-exons on platform: apple-m3-max . . . 42
3.4.3 Operation: coverage for dataset: fBrain-vs-exons on platform: apple-m3-max . . . 45
3.4.4 Operation: count-overlaps for dataset: fBrain-vs-exons on platform: apple-m3-max . . . 47
3.4.5 Operation: overlap for dataset: ex-rna-vs-ex-anno on platform: apple-m3-max . . . 49
3.4.6 Operation: nearest for dataset: ex-rna-vs-ex-anno on platform: apple-m3-max . . . 51
3.4.7 Operation: coverage for dataset: ex-rna-vs-ex-anno on platform: apple-m3-max . . . 54
3.4.8 Operation: count-overlaps for dataset: ex-rna-vs-ex-anno on platform: apple-m3-max 56
3.4.9 Operation: overlap for dataset: 100-1p on platform: apple-m3-max . . . 58
3.4.10 Operation: nearest for dataset: 100-1p on platform: apple-m3-max . . . 60
3.4.11 Operation: coverage for dataset: 100-1p on platform: apple-m3-max . . . 63
3.4.12 Operation: count-overlaps for dataset: 100-1p on platform: apple-m3-max . . . 65
3.4.13 Operation: overlap for dataset: 10000000-1p on platform: apple-m3-max . . . 67
3.4.14 Operation: nearest for dataset: 10000000-1p on platform: apple-m3-max . . . 69
3.4.15 Operation: coverage for dataset: 10000000-1p on platform: apple-m3-max . . . 72
3.4.16 Operation: count-overlaps for dataset: 10000000-1p on platform: apple-m3-max . . . 74
3.4.17 Operation: overlap for dataset: fBrain-vs-exons on platform: gcp-linux . . . 76
3.4.18 Operation: nearest for dataset: fBrain-vs-exons on platform: gcp-linux . . . 78

3.4.19 Operation: coverage for dataset: fBrain-vs-exons on platform: gcp-linux . . . . . . . . 81
3.4.20 Operation: count-overlaps for dataset: fBrain-vs-exons on platform: gcp-linux . . . . . 83
3.4.21 Operation: overlap for dataset: ex-rna-vs-ex-anno on platform: gcp-linux . . . . . . . 85
3.4.22 Operation: nearest for dataset: ex-rna-vs-ex-anno on platform: gcp-linux . . . . . . . 87
3.4.23 Operation: coverage for dataset: ex-rna-vs-ex-anno on platform: gcp-linux . . . . . . . 90
3.4.24 Operation: count-overlaps for dataset: ex-rna-vs-ex-anno on platform: gcp-linux . . . 92
3.4.25 Operation: overlap for dataset: 100-1p on platform: gcp-linux . . . . . . . . . . . . . . 94
3.4.26 Operation: nearest for dataset: 100-1p on platform: gcp-linux . . . . . . . . . . . . . . 96
3.4.27 Operation: coverage for dataset: 100-1p on platform: gcp-linux . . . . . . . . . . . . . 99
3.4.28 Operation: count-overlaps for dataset: 100-1p on platform: gcp-linux . . . . . . . . . . 101
3.4.29 Operation: overlap for dataset: 10000000-1p on platform: gcp-linux . . . . . . . . . . . 103
3.4.30 Operation: nearest for dataset: 10000000-1p on platform: gcp-linux . . . . . . . . . . . 105
3.4.31 Operation: coverage for dataset: 10000000-1p on platform: gcp-linux . . . . . . . . . . 108
3.4.32 Operation: count-overlaps for dataset: 10000000-1p on platform: gcp-linux . . . . . . 110
3.5 Comparison of the libraries . . . . . . . . . . . . . . . . . . . . . . . . . . . . . . . . . 111
3.5.1 Data processing comparison . . . . . . . . . . . . . . . . . . . . . . . . . . . . . . 111
3.5.2 API comparison . . . . . . . . . . . . . . . . . . . . . . . . . . . . . . . . . . . . . 112
3.5.3 Schema and data types comparison . . . . . . . . . . . . . . . . . . . . . . . . . . 113

# 1 Introduction

This document provides additional information about the algorithms, benchmarking setup, data, and results that were presented in the manuscript.

# 2 Algorithm description

`polars-bio` implements a set of *binary* interval operations on genomic ranges, such as *overlap*, *nearest*, *count-overlaps*, and *coverage*. All these operations share the very similar algorithmic structure, which is presented in the diagram below.

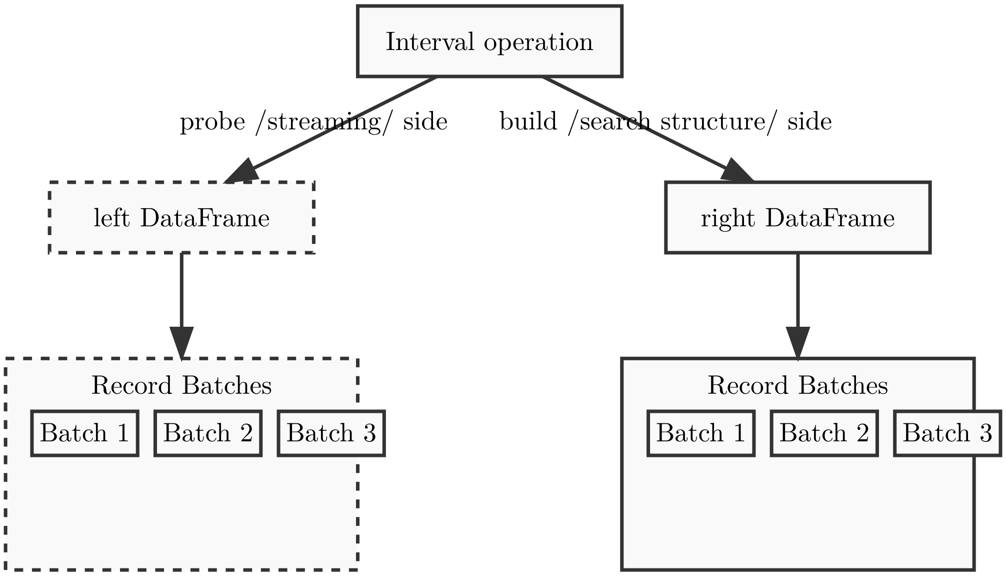


Figure 1: Algorithmic structure of interval operations in polars-bio

The basic concept is that each operation consists of two sides: the **probe** side and the **build** side. The **probe** side is the one that is streamed, while the **build** side is the one that is implemented as a search data structure (for generic *overlap* operation the search structure can be changed using algorithm parameter, for other operations is always Cache Oblivious Interval Trees as according to the benchmark COITrees outperforms other data structures). In the case of *nearest* operation there is an additional sorted list of intervals used for searching for closest intervals in the case of non-existing overlaps.

Note

Available search structure implementations for overlap operation:

- COITrees
- IITree
- AVL-tree
- rust-lapper
- superintervals - available since `polars-bio` version `0.12.0`

Once the **build** side data structure is ready, then records from the **probe** side are processed against the search structure organized as record batches. Each record batch can be processed independently. Search structure nodes contains identifiers of the rows from the **build** side that are then used to construct a new record that is returned as a result of the operation.

## 2.1 Out-of-core (streaming) processing

This algorithm allows you to process your results without requiring **all** your data to be in memory at the same time. In particular, the **probe** side can be streamed from a file stored locally or on a cloud object storage, while the **build** side needs to be fully (i.e. data structure, such as COITrees, holding genomic intervals with identifiers to the whole DataFrame rows) materialized in memory. In real applications, the **probe** side is usually a large file with genomic intervals, while the **build** side is a smaller file with annotations or other genomic features. This allows you to process large genomic datasets without running out of memory.

Note

1. In this sense, the **order** of the sides is important, as the **probe** side is streamed and processed in batches, while the **build** side is fully materialized in memory.
2. The **smaller** the *build* side and **larger** the number of overlaps are, the **higher** is the gain of memory efficiency. For instance, when we compare the real `ex-rna-vs-ex-anno` ($10^7$ `vs.` $1.2*10^6$) and synthetic ($10^7$ `vs.` $10^7$) datasets, we can see that we benefit more from using streaming mode in the **former** benchmark.

## 2.2 Parallelization

In the current implementation, the **probe** side can be processed in parallel using multiple threads on partitioned (implicitly or explicilty partitioned inputs - see partitioning strategies). The **build** side is predominantly single-threaded (with the notable exception of BGZF compressed or partitioned Parquet/CSV input data files reading, which can be parallelized).

## 2.3 Implementation

`polars-bio` uses the following Apache DataFusion extension points:

- DefaultPhysicalPlanner and PhysicalOptimizerRule for detecting and rewriting **generic** interval join operations (i.e. *overlap* and *nearest*) with optimizied execution strategies. This is implemented as a part of our another project sequila-native that exposes optimized interval join operations for Apache DataFusion with both SQL and DataFrame APIs.
- TableProvider and User-Defined Table Function mechanism for implementing **specialized** operations, such as *coverage* and *count-overlaps*.

# 3 Benchmarks

## 3.1 Configuration

### 3.1.1 Code and benchmarking scenarios

Repository: https://github.com/biodatageeks/polars-bio-bench

### 3.1.2 Memory profiling

For memory profiling Python memory-profiler `version 0.61.0` was used. A helper run-memory-profiler.py script was developed and a sample invocation was used to run the tests as it is presented in the snippet below:

```
PRFOF_FILE="polars_bio_1-2.dat"
mprof run --output $PRFOF_FILE python src/run-memory-profiler.py \
--bench-config conf/paper/benchmark-e2e-overlap.yaml \
--tool polars_bio --test-case 1-2

mprof plot $PRFOF_FILE
```

Note

On each memory profile plot, the maximum memory is marked at the intersection of the two dashed lines.

#### 3.1.3 Operating systems and hardware configurations

##### 3.1.3.1 apple-m3-max

- cpu architecture: `arm64`
- cpu name: `Apple M3 Max`
- cpu cores: `16`
- memory: `64 GB`
- kernel: `Darwin Kernel Version 24.2.0: Fri Dec 6 19:02:12 PST 2024; root:xnu-11215.61.5~2/RELEASE_ARM64_T6031`
- system: `Darwin`
- os-release: `macOS-15.2-arm64-arm-64bit`
- python: `3.12.4`
- polars-bio: `0.8.3`

**3.1.3.2 gcp-linux** c3-standard-22 machine was used for benchmarking.

- cpu architecture: `x86_64`
- cpu name: `Intel(R) Xeon(R) Platinum 8481C CPU @ 2.70GHz`
- cpu cores: `22`
- memory: `88 GB`
- kernel: `Linux-6.8.0-1025-gcp-x86_64-with-glibc2.35`
- system: `Linux`
- os-release: `#27~22.04.1-Ubuntu SMP Mon Feb 24 16:42:24 UTC 2025`
- python: `3.12.8`
- polars-bio: `0.8.3`

#### 3.1.4 Libraries versions used in benchmarks

- Bioframe-0.7.2
- PyRanges0-0.0.132
- PyRanges1-e634a11
- pybedtools-0.10.0
- PyGenomics-0.1.1
- GenomicRanges-0.5.0

### 3.2 Datasets

#### 3.2.1 Real dataset

The AIList dataset after transcoding into the Parquet file format (with the Snappy compression) was used for benchmarking (see Table 1). This dataset was published with the AIList paper:

Jianglin Feng , Aakrosh Ratan , Nathan C Sheffield, *Augmented Interval List: a novel data structure for efficient genomic interval search*, Bioinformatics 2019.

Table 1: Real-world datasets used for benchmarking. Source: AIList Github

| Dataset# | Name | Size(x1000) | Description |
|---|---|---|---|
| 0 | chainRn4 | 2,351 | Source |
| 1 | fBrain | 199 | Source |
| 2 | exons | 439 | Dataset used in the BEDTools tutorial. |

Table 1: Real-world datasets used for benchmarking. Source: AIList Github

| Dataset# | Name | Size(x1000) | Description |
|---|---|---|---|
| 3 | chainOrnAna1 | 1,957 | Source |
| 4 | chainVicPac2 | 7,684 | Source |
| 5 | chainXenTro3Link | 50,981 | Source |
| 6 | chainMonDom5Link | 128,187 | Source |
| 7 | ex-anno | 1,194 | Dataset contains GenCode annotations with ~1.2 million lines, mixing all types of features. |
| 8 | ex-rna | 9,945 | Dataset contains ~10 million direct-RNA mappings. |

#### 3.2.2 Selection of 50 ordered pairs from the real-world dataset for benchmarking

To select the 50 real dataset of ordered pairs that are further used for benchmarking, we started from nine unique datasets (AIList benchmark described earlier). We then computed the number of overlaps for each possible combination of these datasets, as shown in Table 2. From the full set of 81 possible ordered pairs, we excluded 22 ordered pairs ($11 \times 2$) that produced more than 1.1 billion overlaps, as these would be computationally extreme. We also excluded self-comparisons (9 pairs). Consequently, the final benchmark dataset comprised of $81 - 22 - 9 = 50$ ordered pairs.

Table 2: Number of overlaps detected for a given combination of datasets calculated with polars-bio (using 0-based coordinate system) in streaming mode.

| Rank | Dataset 1 | Dataset 2 | # of overlaps |
|---|---|---|---|
| 1 | chainMonDom5Link | chainXenTro3Link | 416,157,506,000 |
| 2 | chainMonDom5Link | chainVicPac2 | 248,984,248,721 |
| 3 | chainVicPac2 | chainXenTro3Link | 117,131,343,532 |
| 4 | chainMonDom5Link | chainOrnAna1 | 52,992,648,116 |
| 5 | chainMonDom5Link | chainRn4 | 27,741,145,443 |
| 6 | chainXenTro3Link | chainOrnAna1 | 26,405,758,645 |
| 7 | chainRn4 | chainXenTro3Link | 18,432,254,632 |
| 8 | chainVicPac2 | chainOrnAna1 | 6,864,638,705 |
| 9 | chainMonDom5Link | ex-rna | 4,349,989,219 |
| 10 | chainRn4 | chainVicPac2 | 3,892,115,928 |
| 11 | ex-rna | chainXenTro3Link | 1,830,555,949 |
| 12 | chainRn4 | chainOrnAna1 | 1,086,692,495 |
| 13 | ex-rna | ex-anno | 307,184,634 |
| 14 | ex-rna | chainVicPac2 | 227,832,153 |
| 15 | ex-rna | chainRn4 | 164,196,784 |
| 16 | chainMonDom5Link | exons | 116,300,901 |
| 17 | ex-rna | chainOrnAna1 | 109,300,082 |
| 18 | chainXenTro3Link | exons | 52,395,369 |
| 19 | ex-rna | exons | 36,411,474 |

Table 2: Number of overlaps detected for a given combination of datasets calculated with polars-bio (using 0-based coordinate system) in streaming mode.

| Rank | Dataset 1 | Dataset 2 | # of overlaps |
|---|---|---|---|
| 20 | chainMonDom5Link | ex-anno | 33,966,070 |
| 21 | chainXenTro3Link | ex-anno | 13,693,852 |
| 22 | chainVicPac2 | exons | 10,566,462 |
| 23 | ex-rna | fBrain | 8,385,799 |
| 24 | chainVicPac2 | ex-anno | 5,745,319 |
| 25 | chainOrnAna1 | ex-anno | 4,408,383 |
| 26 | chainOrnAna1 | exons | 3,255,513 |
| 27 | chainRn4 | ex-anno | 2,761,621 |
| 28 | chainRn4 | exons | 2,633,098 |
| 29 | chainMonDom5Link | fBrain | 2,380,147 |
| 30 | fBrain | chainXenTro3Link | 625,718 |
| 31 | fBrain | chainOrnAna1 | 398,738 |
| 32 | fBrain | chainVicPac2 | 357,564 |
| 33 | chainRn4 | fBrain | 320,955 |
| 34 | ex-anno | exons | 273,500 |
| 35 | fBrain | ex-anno | 73,437 |
| 36 | fBrain | exons | 54,246 |

All Parquet files from this dataset shared the same schema:

```
contig STRING
pos_start INT32
pos_end INT32
```

#### 3.2.3 Synthetic dataset

Randomly generated intervals (100-10,000,000) inspired by Bioframe performance analysis. Generated with generate_dataset.py

```
poetry run python src/generate_dataset.py
```

All Parquet files from this dataset shared the same schema:

```
contig STRING
pos_start INT64
pos_end INT64
```

Note

Test datasets in the *Parquet* format can be downloaded from:

- single-thread benchmarks
  - databio.zip
  - random_intervals_20250622_221714-1p.zip
- parallel benchmarks (partitioned)
  - databio-8p.zip
  - random_intervals_20250622_221714-8p.zip

### 3.2.4 Overlap detection counts across libraries

Number of overlaps detected for a given combination of datasets (Test case) calculated with polars-bio, Bioframe, PyRanges0, and PyRanges1 is presented in Table 3.

Table 3: Number of overlaps detected for a given combination of datasets calculated with polars-bio, Bioframe, PyRanges0, and PyRanges1.

| Test case | polars-bio[1] | Bioframe[2] | PyRanges0 | PyRanges1 |
|---|---|---|---|---|
| fBrain-vs-exons | 54,246 | 54,246 | 54,246 | 54,246 |
| ex-rna-vs-ex-anno | 307,184,634 | 307,184,634 | 307,184,634 | 307,184,634 |
| 100-vs-100 | 781 | 781 | 781 | 781 |
| 1000-vs-1000 | 8,859 | 8,859 | 8,859 | 8,859 |
| 10000-vs-10000 | 90,236 | 90,236 | 90,236 | 90,236 |
| 100000-vs-100000 | 902,553 | 902,553 | 902,553 | 902,553 |
| 1000000-vs-1000000 | 9,007,817 | 9,007,817 | 9,007,817 | 9,007,817 |
| 10000000-vs-10000000 | 90,005,371 | 90,005,371 | 90,005,371 | 90,005,371 |

[1] Bioframe and PyRanges are zero-based, this is why we need to set `use_zero_based=True` (polars-bio >= 0.10.3) in polars-bio to get the same results as in Bioframe and PyRanges.

[2] Bioframe `how` parameter is set to `inner` (`left` by default)

## 3.3 Performance evaluation

### 3.3.1 Summary statistics

In this section, we summarize the results of the performance evaluation conducted on 50 real-world dataset pairs described in Section 3.2.2. We compared the execution times and memory peak utitlization of `polars-bio` with other commonly used genomic data processing libraries across several operations (`count_overlaps`, `coverage`, `nearest`, and `overlap`). For each operation and library, we computed summary statistics of the speedup and memory peak usage relative to `Bioframe`, including the minimum, first quartile, median, mean, third quartile, maximum, and standard deviation.

**3.3.1.1 Speedup summary statistics** Figure 2 visualizes the distributions of these summary statistics, illustrating the performance differences between libraries. The figure also highlights the scalability of `polars-bio`, showing performance improvements with increasing number of parallel threads. Detailed numerical results for each operation and library are presented in Table 4.

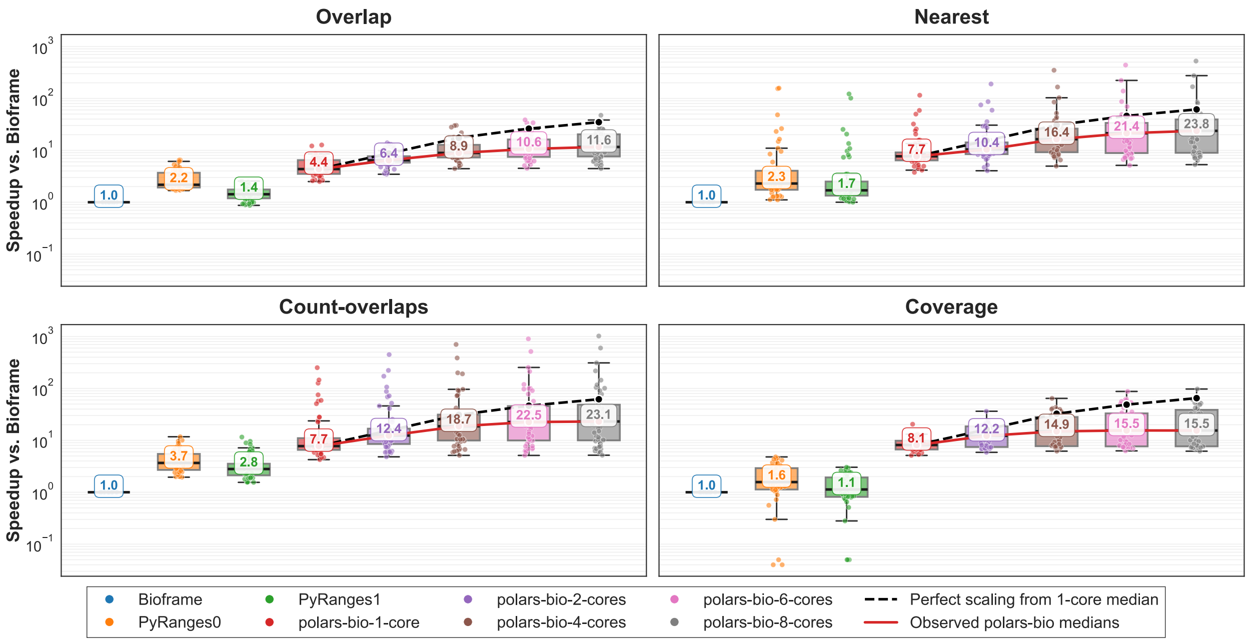


Figure 2: Distribution of the summary statistics for the speedup (relative to `Bioframe`) across the 50 real-world dataset pairs selected as described in Section 3.2.2. The figure shows the distributions for individual libraries and for `polars-bio` using different numbers of threads, illustrating its scalability across parallel configurations.

Table 4: Speedup relative to Bioframe by operation and library

| operation | Library | pairs # | min | q1 | median | mean | q3 | max | std_dev |
|---|---|---|---|---|---|---|---|---|---|
| count-overlaps | Bioframe | 50 | 1 | 1 | 1 | 1 | 1 | 1 | 0 |
| count-overlaps | PyRanges0 | 50 | 1.95 | 2.695 | 3.665 | 4.4076 | 5.4675 | 11.72 | 2.30292 |
| count-overlaps | PyRanges1 | 50 | 1.55 | 2.1225 | 2.8 | 3.5978 | 3.545 | 11.56 | 2.33937 |
| count-overlaps | polars-bio-1-core | 50 | 4.25 | 6.5525 | 7.74 | 23.0962 | 11.0075 | 251.1 | 43.6558 |
| count-overlaps | polars-bio-2-cores | 50 | 4.81 | 8.5075 | 12.365 | 35.38 | 16.79 | 448.82 | 72.7333 |
| count-overlaps | polars-bio-4-cores | 50 | 5.13 | 9.935 | 18.685 | 52.6408 | 31.3325 | 703.71 | 114.151 |
| count-overlaps | polars-bio-6-cores | 50 | 5.12 | 9.95 | 22.535 | 65.241 | 45.6675 | 901.72 | 146.821 |
| count-overlaps | polars-bio-8-cores | 50 | 5.17 | 9.9925 | **23.09** | 72.7148 | 48.5175 | 1022.25 | 168.018 |
| coverage | Bioframe | 50 | 1 | 1 | 1 | 1 | 1 | 1 | 0 |
| coverage | PyRanges0 | 50 | 0.04 | 1.13 | 1.57 | 2.063 | 2.9075 | 4.79 | 1.3065 |
| coverage | PyRanges1 | 50 | 0.05 | 0.815 | 1.13 | 1.3786 | 1.9275 | 3.03 | 0.790766 |
| coverage | polars-bio-1-core | 50 | 5.12 | 6.665 | 8.125 | 8.4878 | 9.815 | 20.45 | 2.55064 |
| coverage | polars-bio-2-cores | 50 | 5.87 | 7.4475 | 12.22 | 13.2026 | 18.59 | 36.35 | 6.4305 |
| coverage | polars-bio-4-cores | 50 | 6.19 | 7.7175 | 14.9 | 19.2838 | 28.09 | 64.32 | 13.5017 |
| coverage | polars-bio-6-cores | 50 | 6.27 | 7.65 | **15.5** | 23.8294 | 33.105 | 87.5 | 19.8054 |
| coverage | polars-bio-8-cores | 50 | 6.18 | 7.665 | 15.455 | 25.4024 | 38.7875 | 97.4 | 22.0352 |
| nearest | Bioframe | 50 | 1 | 1 | 1 | 1 | 1 | 1 | 0 |
| nearest | PyRanges0 | 50 | 1.11 | 1.7375 | 2.295 | 11.2596 | 4.0375 | 157.89 | 30.9698 |
| nearest | PyRanges1 | 50 | 1 | 1.34 | 1.695 | 7.8664 | 2.5025 | 121.95 | 22.0401 |
| nearest | polars-bio-1-core | 50 | 3.76 | 6.415 | 7.675 | 13.0408 | 9.1175 | 115.1 | 18.1307 |
| nearest | polars-bio-2-cores | 50 | 4.04 | 8.4525 | 10.385 | 19.4568 | 14.1875 | 189.69 | 29.5579 |
| nearest | polars-bio-4-cores | 50 | 4.96 | 9.1975 | 16.405 | 30.565 | 26.17 | 348.88 | 53.5395 |

| operation | Library | pairs # | min | q1 | median | mean | q3 | max | std_dev |
|---|---|---|---|---|---|---|---|---|---|
| nearest | polars-bio-6-cores | 50 | 5.13 | 8.8675 | 21.355 | 37.475 | 33.6925 | 439.1 | 68.3856 |
| nearest | polars-bio-8-cores | 50 | 5.3 | 8.955 | **23.755** | 43.1192 | 39.3275 | 524.96 | 82.4501 |
| overlap | Bioframe | 50 | 1 | 1 | 1 | 1 | 1 | 1 | 0 |
| overlap | PyRanges0 | 50 | 1.68 | 1.92 | 2.17 | 2.9496 | 3.69 | 6.52 | 1.36965 |
| overlap | PyRanges1 | 50 | 0.87 | 1.19 | 1.425 | 1.4942 | 1.7575 | 2.56 | 0.430946 |
| overlap | polars-bio-1-core | 50 | 2.5 | 3.5125 | 4.355 | 5.1444 | 6.475 | 12.68 | 2.23919 |
| overlap | polars-bio-2-cores | 50 | 3.46 | 5.785 | 6.445 | 7.171 | 7.6875 | 13.93 | 2.54744 |
| overlap | polars-bio-4-cores | 50 | 4.43 | 7.3 | 8.905 | 11.0772 | 12.7475 | 30.24 | 6.02291 |
| overlap | polars-bio-6-cores | 50 | 4.5 | 7.4725 | 10.63 | 13.2754 | 16.1825 | 38.56 | 7.92406 |
| overlap | polars-bio-8-cores | 50 | 4.45 | 7.61 | **11.6** | 15.0246 | 20.35 | 47.33 | 9.81393 |

**3.3.1.2 Memory peak summary statistics** Figure 3 summarizes *peak memory* usage (relative to `Bioframe`) for the 50 real-world dataset pairs introduced in Section 3.2.2. We report per-library distributions and, for `polars-bio`, both non-streaming and streaming modes. To contextualize memory behavior on large outputs, we additionally show a subset labeled (">10GB") that includes only the 16 pairs for which the `Bioframe` overlap operation consumes more than 10GB of memory (i.e. memory intensive because of inputs and/or output size). Detailed numerical results by operation and library are provided in Table 5.

For the this subset (">10GB"), `polars-bio` exhibits the greatest reduction in peak memory among all libraries, with the effect most pronounced in *streaming* mode.

We identified two main situations in which `polars-bio` may exhibit slightly higher peak memory usage than `Bioframe` (Table 6). The first one is related to output batches *explosion*, which occurs when the number of overlapping pairs greatly exceeds the size of the input datasets; this issue has been addressed by introducing the `low_memory` mode, in which the size of each output batch produced is constrained to a fix number ($10^5$ by default). The second case concerns the *order* of input datasets: when the index is built from the larger dataset instead of the smaller one, the temporary memory footprint can increase slightly. Although this applies to only a small subset of cases (the median and third quartile of memory ratio distributions relative to `Bioframe` remain well below 1) and can be easily mitigated by reversing the dataset order. In future releases of `polars-bio` we plan to introduce an automatic mechanism for detecting the larger dataset and reordering the inputs to the overlap operation.

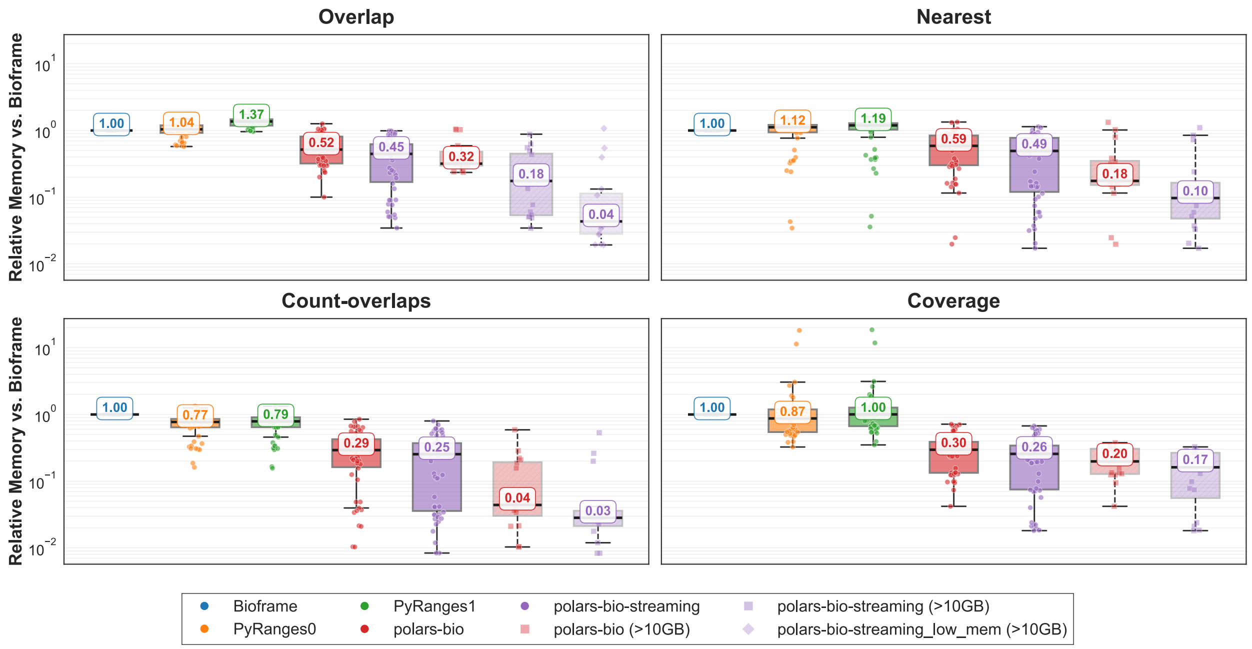


Figure 3: Distribution of peak memory (relative to `Bioframe`) across the 50 real-world dataset pairs selected in Section 3.2.2. The figure shows per-library distributions, including `polars-bio` with and without streaming mode. To highlight relative memory savings for high-output pairs, we also include boxplots labeled (">10GB") that are restricted to the 16 pairs for which `Bioframe` overlap operation consumes more than 10GB. Two streaming configurations of `polars-bio` are shown: `polars-bio-streaming`, which enables standard streaming mode allowing data to be processed sequentially without materializing the full result in memory, and `polars-bio-streaming_low_mem`, which further limits the output materialized in a single batch to $10^5$ rows at a time to minimize transient memory usage during generating large overlap outputs. Note that `polars-bio-streaming_low_mem` is enabled for the *overlap* operation only, since only this operation can produce extremely large output data volumes.

Table 5: Memory usage relative to Bioframe by operation and library

| operation | Library | pairs # | min | q1 | median | mean | q3 | max | std_dev |
|---|---|---|---|---|---|---|---|---|---|
| count-overlaps | Bioframe | 50 | 1.0000 | 1.0000 | 1.0000 | 1.0000 | 1.0000 | 1.0000 | 0.0000 |
| count-overlaps | polars-bio | 50 | 0.0103 | 0.1627 | 0.2926 | 0.3148 | 0.4257 | 0.8501 | 0.2241 |
| count-overlaps | polars-bio-streaming | 50 | 0.0084 | 0.0359 | **0.2543** | 0.2555 | 0.3719 | 0.7997 | 0.2202 |
| count-overlaps | PyRanges0 | 50 | 0.1619 | 0.6423 | 0.7728 | 0.7415 | 0.8560 | 1.3392 | 0.2827 |
| count-overlaps | PyRanges1 | 50 | 0.1564 | 0.6449 | 0.7893 | 0.7347 | 0.9083 | 1.3276 | 0.2620 |
| coverage | Bioframe | 50 | 1.0000 | 1.0000 | 1.0000 | 1.0000 | 1.0000 | 1.0000 | 0.0000 |
| coverage | polars-bio | 50 | 0.0420 | 0.1336 | 0.2963 | 0.3058 | 0.3883 | 0.7162 | 0.1811 |
| coverage | polars-bio-streaming | 50 | 0.0182 | 0.0752 | **0.2568** | 0.2539 | 0.3421 | 0.6739 | 0.1833 |
| coverage | PyRanges0 | 50 | 0.3255 | 0.5450 | 0.8726 | 1.4964 | 1.1932 | 18.2441 | 2.8835 |
| coverage | PyRanges1 | 50 | 0.3499 | 0.6680 | 1.0036 | 1.6119 | 1.2688 | 18.6209 | 2.9367 |
| nearest | Bioframe | 50 | 1.0000 | 1.0000 | 1.0000 | 1.0000 | 1.0000 | 1.0000 | 0.0000 |
| nearest | polars-bio | 50 | 0.0198 | 0.3022 | 0.5888 | 0.5760 | 0.8426 | 1.3399 | 0.3409 |
| nearest | polars-bio-streaming | 50 | 0.0172 | 0.1207 | **0.4941** | 0.4672 | 0.7749 | 1.1417 | 0.3548 |

Continued on next page

| operation | Library | pairs # | min | q1 | median | mean | q3 | max | std_dev |
|---|---|---|---|---|---|---|---|---|---|
| nearest | PyRanges0 | 50 | 0.0344 | 0.9404 | 1.1179 | 0.9930 | 1.2143 | 1.5231 | 0.3880 |
| nearest | PyRanges1 | 50 | 0.0359 | 1.0298 | 1.1926 | 1.0420 | 1.2874 | 1.6070 | 0.4019 |
| overlap | Bioframe | 50 | 1.0000 | 1.0000 | 1.0000 | 1.0000 | 1.0000 | 1.0000 | 0.0000 |
| overlap | polars-bio | 50 | 0.1004 | 0.3206 | 0.5180 | 0.5654 | 0.8111 | 1.2639 | 0.2879 |
| overlap | polars-bio (>10GB) | 16 | 0.2352 | 0.2978 | 0.3188 | 0.4688 | 0.4796 | 1.0529 | 0.2945 |
| overlap | polars-bio-streaming | 50 | 0.0345 | 0.1691 | 0.4459 | 0.4435 | 0.6236 | 0.9897 | 0.2944 |
| overlap | polars-bio-streaming (>10GB) | 16 | 0.0345 | 0.0538 | 0.1751 | 0.2626 | 0.4490 | 0.8809 | 0.2591 |
| overlap | polars-bio-streaming_low_mem (>10GB) | 16 | 0.0193 | 0.0282 | **0.0434** | 0.1662 | 0.1135 | 1.0754 | 0.2847 |
| overlap | PyRanges0 | 50 | 0.5756 | 0.9228 | 1.0407 | 1.0202 | 1.1957 | 1.4879 | 0.2276 |
| overlap | PyRanges1 | 50 | 0.9617 | 1.1846 | 1.3667 | 1.3337 | 1.4724 | 2.0385 | 0.2124 |

Table 6: Analysis of the cases when polars-bio consumes more memory than Bioframe for the overlap operation

| pair | cause | Bioframe [MB] | polars-bio [MB] | streaming [MB] | streaming_low_mem [MB] | memory ratio (before/after optimization) |
|---|---|---|---|---|---|---|
| 0-3 | batch explosion | 38631.56 | 39938.56 | 24104.67 | **749.14** | 1.0338 |
| 3-0 | - | 40675.70 | 41434.16 | 20056.09 | **783.11** | **0.0194** |
| 1-6 | order | 9444.61 | 11937.17 | 9300.53 | - | 1.2639 |
| 6-1 | - | 9172.75 | 3289.38 | **721.39** | - | **0.0763** |
| 7-6 | order | 12036.02 | 12672.88 | 10602.44 | - | 1.0529 |
| 6-7 | - | 11923.64 | 2804.50 | **912.52** | - | **0.0758** |
| 7-6[1] | order | 12036.02 | 12672.88 | 10602.44 | 12943.33 | 1.0754 |
| 6-7 | - | 11923.64 | 2804.50 | **912.52** | - | **0.0758** |
| 1-7 | order | 369.92 | 379.11 | 332.34 | - | 1.0248 |
| 7-1 | - | 376.92 | 307.22 | **281.67** | - | **0.7614** |
| 1-2 | order | 287.59 | 305.44 | 284.64 | - | 1.0620 |
| 2-1 | - | 291.98 | 285.67 | **272.25** | - | **0.9466** |

### 3.3.2 Single-thread results

Results for `overlap`, `nearest`, `count-overlaps`, and `coverage` operations with single-thread performance on `apple-m3-max` and `gcp-linux` platforms.

Note

Please note that in case of `PyRanges0` we were unable to compute the results of *coverage* and *count-overlaps* operations for macOS and Linux in the synthetic benchmark, so the results are not presented here.

We report detailed single-thread results for a subset of representative dataset pairs. For the *real-world* benchmark, we show per-operation timings for the smallest pair (`fBrain-vs-exons`) and for the second-largest pair (`ex-rna-vs-ex-anno`), which yields on the order of 300M candidate intersections (we do *not* include the extreme ∼1.1B case in this section). For the *synthetic* benchmark, we provide results across

[1] This is a duplicate case – but this time when low memory optimization compounds the problem of not optimal dataset input order

six configurations that span five orders of magnitude: `100-1p`, `1000-1p`, `10000-1p`, `100000-1p`, `1000000-1p`, and `10000000-1p`—to illustrate performance trends from very small to very large inputs.

#### 3.3.2.1 apple-m3-max

Table 7: Performance of the *overlap* operation on fBrain-vs-exons

| Library | Min (s) | Max (s) | Mean (s) | Speedup |
|---|---|---|---|---|
| polars-bio | 0.035619 | 0.043113 | 0.0383 | 2.70x |
| Bioframe | 0.102257 | 0.104425 | 0.103354 | 1.00x |
| PyRanges0 | 0.025425 | 0.032821 | 0.028001 | 3.69x |
| PyRanges1 | 0.059608 | 0.064147 | 0.061763 | 1.67x |
| pybedtools | 0.343204 | 0.352804 | 0.348434 | 0.30x |
| GenomicRanges | 1.042893 | 1.044245 | 1.043488 | 0.10x |

Table 8: Performance of the *nearest* operation on fBrain-vs-exons

| Library | Min (s) | Max (s) | Mean (s) | Speedup |
|---|---|---|---|---|
| polars-bio | 0.039943 | 0.045166 | 0.042109 | 4.45x |
| Bioframe | 0.185452 | 0.189631 | 0.187388 | 1.00x |
| PyRanges0 | 0.092334 | 0.09634 | 0.093688 | 2.00x |
| PyRanges1 | 0.133631 | 0.134179 | 0.133981 | 1.40x |
| pybedtools | 0.756676 | 0.761866 | 0.75953 | 0.25x |

Table 9: Performance of the *count overlaps* operation on fBrain-vs-exons

| Library | Min (s) | Max (s) | Mean (s) | Speedup |
|---|---|---|---|---|
| polars-bio | 0.026706 | 0.029754 | 0.028142 | 4.69x |
| Bioframe | 0.131124 | 0.133729 | 0.132052 | 1.00x |
| PyRanges0 | 0.039136 | 0.039774 | 0.039377 | 3.35x |
| PyRanges1 | 0.061976 | 0.063181 | 0.062658 | 2.11x |
| pybedtools | 0.665804 | 0.673844 | 0.668534 | 0.20x |
| GenomicRanges | 0.994963 | 1.006435 | 0.999389 | 0.13x |

Table 10: Performance of the *coverage* operation on fBrain-vs-exons

| Library | Min (s) | Max (s) | Mean (s) | Speedup |
|---|---|---|---|---|
| polars-bio | 0.0262 | 0.028749 | 0.027418 | 6.30x |
| Bioframe | 0.16949 | 0.176628 | 0.172842 | 1.00x |
| PyRanges0 | 0.07376 | 0.076708 | 0.075369 | 2.29x |
| PyRanges1 | 0.128027 | 0.133263 | 0.130247 | 1.33x |
| pybedtools | 0.701817 | 0.708726 | 0.705839 | 0.24x |
| GenomicRanges | 1.032651 | 1.049059 | 1.040799 | 0.17x |

Table 11: Performance of the *overlap* operation on ex-rna-vs-ex-anno

| Library | Min (s) | Max (s) | Mean (s) | Speedup |
|---|---|---|---|---|
| polars-bio | 3.987391 | 4.648581 | 4.235518 | 7.17x |
| Bioframe | 29.793837 | 30.991576 | 30.375518 | 1.00x |
| PyRanges0 | 15.632212 | 15.974075 | 15.857213 | 1.92x |
| PyRanges1 | 31.622804 | 33.699074 | 32.680701 | 0.93x |
| pybedtools | 916.711575 | 919.974811 | 918.154834 | 0.03x |
| GenomicRanges | 479.214112 | 487.832054 | 484.579554 | 0.06x |

Table 12: Performance of the *nearest* operation on ex-rna-vs-ex-anno

| Library | Min (s) | Max (s) | Mean (s) | Speedup |
|---|---|---|---|---|
| polars-bio | 2.116922 | 2.169534 | 2.139006 | 32.13x |
| Bioframe | 68.581465 | 68.992651 | 68.725495 | 1.00x |
| PyRanges0 | 1.381964 | 1.508513 | 1.424446 | 48.25x |
| PyRanges1 | 2.697684 | 2.728407 | 2.717532 | 25.29x |
| pybedtools | 35.528719 | 35.876667 | 35.699544 | 1.93x |

Table 13: Performance of the *count overlaps* operation on ex-rna-vs-ex-anno

| Library | Min (s) | Max (s) | Mean (s) | Speedup |
|---|---|---|---|---|
| polars-bio | 1.445467 | 1.484052 | 1.46225 | 58.77x |
| Bioframe | 85.632767 | 86.26148 | 85.935955 | 1.00x |
| PyRanges0 | 9.674847 | 9.833233 | 9.753982 | 8.81x |
| PyRanges1 | 10.170249 | 10.254359 | 10.201813 | 8.42x |
| pybedtools | 33.101592 | 33.966188 | 33.423595 | 2.57x |
| GenomicRanges | 488.972732 | 490.395787 | 489.548184 | 0.18x |

Table 14: Performance of the *coverage* operation on ex-rna-vs-ex-anno

| Library | Min (s) | Max (s) | Mean (s) | Speedup |
|---|---|---|---|---|
| polars-bio | 1.195279 | 1.205765 | 1.199323 | 20.45x |
| Bioframe | 24.423391 | 24.682901 | 24.525909 | 1.00x |
| PyRanges0 | 11.093644 | 11.328071 | 11.220416 | 2.19x |
| PyRanges1 | 11.987003 | 12.147925 | 12.066045 | 2.03x |
| pybedtools | 59.699275 | 60.04087 | 59.84965 | 0.41x |
| GenomicRanges | 500.041974 | 503.31936 | 502.043072 | 0.05x |

Table 15: Performance of the *overlap* operation on 100-1p

| Library | Min (s) | Max (s) | Mean (s) | Speedup |
|---|---|---|---|---|
| polars-bio | 0.002471 | 0.006262 | 0.003855 | 0.54x |
| Bioframe | 0.001374 | 0.002735 | 0.002067 | 1.00x |
| PyRanges0 | 0.000977 | 0.001952 | 0.001337 | 1.55x |

Table 15: Performance of the *overlap* operation on 100-1p

| Library | Min (s) | Max (s) | Mean (s) | Speedup |
|---|---|---|---|---|
| PyRanges1 | 0.002276 | 0.003591 | 0.002739 | 0.75x |
| pybedtools | 0.006856 | 0.010064 | 0.008032 | 0.26x |
| GenomicRanges | 0.001784 | 0.002115 | 0.001938 | 1.07x |
| PyGenomics | 0.000475 | 0.000541 | 0.000509 | 4.06x |

Table 16: Performance of the *nearest* operation on 100-1p

| Library | Min (s) | Max (s) | Mean (s) | Speedup |
|---|---|---|---|---|
| polars-bio | 0.002802 | 0.007312 | 0.004371 | 0.51x |
| Bioframe | 0.00157 | 0.00347 | 0.002251 | 1.00x |
| PyRanges0 | 0.00135 | 0.004085 | 0.002281 | 0.99x |
| PyRanges1 | 0.002084 | 0.003622 | 0.002633 | 0.85x |
| pybedtools | 0.005288 | 0.023073 | 0.011717 | 0.19x |

Table 17: Performance of the *count overlaps* operation on 100-1p

| Library | Min (s) | Max (s) | Mean (s) | Speedup |
|---|---|---|---|---|
| polars-bio | 0.001892 | 0.006355 | 0.003397 | 0.52x |
| Bioframe | 0.001563 | 0.002165 | 0.001775 | 1.00x |
| PyRanges1 | 0.00181 | 0.002209 | 0.001972 | 0.90x |
| pybedtools | 0.020892 | 0.062978 | 0.036866 | 0.05x |
| GenomicRanges | 0.001896 | 0.002057 | 0.001957 | 0.91x |

Table 18: Performance of the *coverage* operation on 100-1p

| Library | Min (s) | Max (s) | Mean (s) | Speedup |
|---|---|---|---|---|
| polars-bio | 0.001911 | 0.006057 | 0.003343 | 1.03x |
| Bioframe | 0.003065 | 0.00411 | 0.003452 | 1.00x |
| PyRanges1 | 0.004455 | 0.005845 | 0.005021 | 0.69x |
| pybedtools | 0.02477 | 0.059532 | 0.037421 | 0.09x |

Table 19: Performance of the *overlap* operation on 1000-1p

| Library | Min (s) | Max (s) | Mean (s) | Speedup |
|---|---|---|---|---|
| polars-bio | 0.00262 | 0.004367 | 0.003278 | 0.71x |
| Bioframe | 0.001909 | 0.002988 | 0.002313 | 1.00x |
| PyRanges0 | 0.001361 | 0.00182 | 0.001543 | 1.50x |
| PyRanges1 | 0.002678 | 0.003166 | 0.002927 | 0.79x |
| pybedtools | 0.037238 | 0.039737 | 0.038453 | 0.06x |
| GenomicRanges | 0.019265 | 0.019945 | 0.01957 | 0.12x |
| PyGenomics | 0.006876 | 0.006994 | 0.006949 | 0.33x |

Table 20: Performance of the *nearest* operation on 1000-1p

| Library | Min (s) | Max (s) | Mean (s) | Speedup |
|---|---|---|---|---|
| polars-bio | 0.003048 | 0.0083 | 0.00553 | 0.65x |
| Bioframe | 0.003269 | 0.004119 | 0.003604 | 1.00x |
| PyRanges0 | 0.002514 | 0.003506 | 0.003099 | 1.16x |
| PyRanges1 | 0.003722 | 0.00418 | 0.003935 | 0.92x |
| pybedtools | 0.00881 | 0.011281 | 0.009729 | 0.37x |

Table 21: Performance of the *count overlaps* operation on 1000-1p

| Library | Min (s) | Max (s) | Mean (s) | Speedup |
|---|---|---|---|---|
| polars-bio | 0.001854 | 0.004714 | 0.002898 | 1.00x |
| Bioframe | 0.002523 | 0.003547 | 0.002898 | 1.00x |
| PyRanges1 | 0.002302 | 0.002838 | 0.002498 | 1.16x |
| pybedtools | 0.032681 | 0.047822 | 0.037981 | 0.08x |
| GenomicRanges | 0.020029 | 0.02029 | 0.020192 | 0.14x |

Table 22: Performance of the *coverage* operation on 1000-1p

| Library | Min (s) | Max (s) | Mean (s) | Speedup |
|---|---|---|---|---|
| polars-bio | 0.002202 | 0.003516 | 0.002696 | 1.77x |
| Bioframe | 0.004238 | 0.005691 | 0.004758 | 1.00x |
| PyRanges1 | 0.004909 | 0.005934 | 0.005284 | 0.90x |
| pybedtools | 0.030735 | 0.045004 | 0.03646 | 0.13x |

Table 23: Performance of the *overlap* operation on 10000-1p

| Library | Min (s) | Max (s) | Mean (s) | Speedup |
|---|---|---|---|---|
| polars-bio | 0.004603 | 0.008294 | 0.006073 | 1.81x |
| Bioframe | 0.010529 | 0.011367 | 0.011014 | 1.00x |
| PyRanges0 | 0.006498 | 0.007306 | 0.006811 | 1.62x |
| PyRanges1 | 0.01096 | 0.012611 | 0.011684 | 0.94x |
| pybedtools | 0.94646 | 0.94995 | 0.948121 | 0.01x |
| GenomicRanges | 0.198868 | 0.200266 | 0.199428 | 0.06x |
| PyGenomics | 0.080325 | 0.08121 | 0.080663 | 0.14x |

Table 24: Performance of the *nearest* operation on 10000-1p

| Library | Min (s) | Max (s) | Mean (s) | Speedup |
|---|---|---|---|---|
| polars-bio | 0.004851 | 0.007782 | 0.005908 | 4.50x |
| Bioframe | 0.025947 | 0.027779 | 0.026584 | 1.00x |
| PyRanges0 | 0.00501 | 0.005703 | 0.00526 | 5.05x |
| PyRanges1 | 0.007517 | 0.007937 | 0.00769 | 3.46x |
| pybedtools | 0.040749 | 0.043864 | 0.041889 | 0.63x |

Table 25: Performance of the *count overlaps* operation on 10000-1p

| Library | Min (s) | Max (s) | Mean (s) | Speedup |
|---|---|---|---|---|
| polars-bio | 0.003283 | 0.008069 | 0.005083 | 3.12x |
| Bioframe | 0.014669 | 0.016689 | 0.015834 | 1.00x |
| PyRanges1 | 0.007637 | 0.008979 | 0.008178 | 1.94x |
| pybedtools | 0.720797 | 0.730655 | 0.725407 | 0.02x |
| GenomicRanges | 0.202131 | 0.209398 | 0.204628 | 0.08x |

Table 26: Performance of the *coverage* operation on 10000-1p

| Library | Min (s) | Max (s) | Mean (s) | Speedup |
|---|---|---|---|---|
| polars-bio | 0.002756 | 0.004613 | 0.003377 | 3.06x |
| Bioframe | 0.009849 | 0.011243 | 0.010339 | 1.00x |
| PyRanges1 | 0.01326 | 0.015308 | 0.013973 | 0.74x |
| pybedtools | 0.727294 | 0.733098 | 0.73116 | 0.01x |

Table 27: Performance of the *overlap* operation on 100000-1p

| Library | Min (s) | Max (s) | Mean (s) | Speedup |
|---|---|---|---|---|
| polars-bio | 0.030583 | 0.038892 | 0.033394 | 3.33x |
| Bioframe | 0.108358 | 0.115233 | 0.111059 | 1.00x |
| PyRanges0 | 0.059633 | 0.065599 | 0.061791 | 1.80x |
| PyRanges1 | 0.100074 | 0.105947 | 0.102267 | 1.09x |
| pybedtools | 13.434458 | 13.602339 | 13.496321 | 0.01x |
| GenomicRanges | 2.030365 | 2.052434 | 2.039897 | 0.05x |
| PyGenomics | 1.001974 | 1.018231 | 1.009213 | 0.11x |

Table 28: Performance of the *nearest* operation on 100000-1p

| Library | Min (s) | Max (s) | Mean (s) | Speedup |
|---|---|---|---|---|
| polars-bio | 0.03013 | 0.036718 | 0.03339 | 10.61x |
| Bioframe | 0.352786 | 0.356839 | 0.354241 | 1.00x |
| PyRanges0 | 0.032403 | 0.034701 | 0.033667 | 10.52x |
| PyRanges1 | 0.044958 | 0.046169 | 0.045629 | 7.76x |
| pybedtools | 0.369122 | 0.379131 | 0.3729 | 0.95x |

Table 29: Performance of the *count overlaps* operation on 100000-1p

| Library | Min (s) | Max (s) | Mean (s) | Speedup |
|---|---|---|---|---|
| polars-bio | 0.021035 | 0.026894 | 0.023802 | 13.86x |
| Bioframe | 0.308013 | 0.347919 | 0.329806 | 1.00x |
| PyRanges1 | 0.076199 | 0.085019 | 0.079372 | 4.16x |
| pybedtools | 11.056327 | 11.280248 | 11.149039 | 0.03x |
| GenomicRanges | 2.057607 | 2.07651 | 2.067998 | 0.16x |

Table 30: Performance of the *coverage* operation on 100000-1p

| Library | Min (s) | Max (s) | Mean (s) | Speedup |
|---|---|---|---|---|
| polars-bio | 0.013263 | 0.014998 | 0.013874 | 5.68x |
| Bioframe | 0.077717 | 0.081116 | 0.078865 | 1.00x |
| PyRanges1 | 0.094753 | 0.114552 | 0.10257 | 0.77x |
| pybedtools | 11.374602 | 11.428316 | 11.393849 | 0.01x |

Table 31: Performance of the *overlap* operation on 1000000-1p

| Library | Min (s) | Max (s) | Mean (s) | Speedup |
|---|---|---|---|---|
| polars-bio | 0.482548 | 0.538737 | 0.507383 | 2.55x |
| Bioframe | 1.26082 | 1.35031 | 1.296195 | 1.00x |
| PyRanges0 | 0.775969 | 0.828801 | 0.810501 | 1.60x |
| PyRanges1 | 1.272326 | 1.29706 | 1.28585 | 1.01x |

Table 32: Performance of the *nearest* operation on 1000000-1p

| Library | Min (s) | Max (s) | Mean (s) | Speedup |
|---|---|---|---|---|
| polars-bio | 0.439544 | 0.488414 | 0.458975 | 14.86x |
| Bioframe | 6.592501 | 7.111734 | 6.818208 | 1.00x |
| PyRanges0 | 0.398173 | 0.413055 | 0.406623 | 16.77x |
| PyRanges1 | 0.51649 | 0.520946 | 0.518407 | 13.15x |

Table 33: Performance of the *count overlaps* operation on 1000000-1p

| Library | Min (s) | Max (s) | Mean (s) | Speedup |
|---|---|---|---|---|
| polars-bio | 0.257781 | 0.305275 | 0.28525 | 17.65x |
| Bioframe | 4.640915 | 5.437883 | 5.033454 | 1.00x |
| PyRanges1 | 0.916714 | 0.925945 | 0.920594 | 5.47x |

Table 34: Performance of the *coverage* operation on 1000000-1p

| Library | Min (s) | Max (s) | Mean (s) | Speedup |
|---|---|---|---|---|
| polars-bio | 0.128241 | 0.137198 | 0.132474 | 7.71x |
| Bioframe | 0.996542 | 1.065777 | 1.021738 | 1.00x |
| PyRanges1 | 1.115134 | 1.247674 | 1.172964 | 0.87x |

Table 35: Performance of the *overlap* operation on 10000000-1p

| Library | Min (s) | Max (s) | Mean (s) | Speedup |
|---|---|---|---|---|
| polars-bio | 8.532137 | 9.738828 | 8.978132 | 2.20x |
| Bioframe | 19.276665 | 20.295566 | 19.708064 | 1.00x |

Table 35: Performance of the *overlap* operation on 10000000-1p

| Library | Min (s) | Max (s) | Mean (s) | Speedup |
|---|---|---|---|---|
| PyRanges0 | 14.819439 | 15.339048 | 15.092611 | 1.31x |
| PyRanges1 | 20.153432 | 22.654892 | 21.56345 | 0.91x |

Table 36: Performance of the *nearest* operation on 10000000-1p

| Library | Min (s) | Max (s) | Mean (s) | Speedup |
|---|---|---|---|---|
| polars-bio | 7.12779 | 7.490779 | 7.263011 | 22.17x |
| Bioframe | 156.356696 | 169.531002 | 160.989714 | 1.00x |
| PyRanges0 | 6.402183 | 6.879779 | 6.62806 | 24.29x |
| PyRanges1 | 7.526236 | 8.176338 | 7.857803 | 20.49x |

Table 37: Performance of the *count overlaps* operation on 10000000-1p

| Library | Min (s) | Max (s) | Mean (s) | Speedup |
|---|---|---|---|---|
| polars-bio | 4.887937 | 5.553197 | 5.165014 | 20.21x |
| Bioframe | 102.637625 | 105.903506 | 104.389343 | 1.00x |
| PyRanges1 | 13.35283 | 15.167609 | 14.19713 | 7.35x |

Table 38: Performance of the *coverage* operation on 10000000-1p

| Library | Min (s) | Max (s) | Mean (s) | Speedup |
|---|---|---|---|---|
| polars-bio | 1.627897 | 1.683304 | 1.655288 | 9.86x |
| Bioframe | 15.586487 | 16.774274 | 16.316676 | 1.00x |
| PyRanges1 | 16.99118 | 17.447484 | 17.195844 | 0.95x |

#### 3.3.2.2 gcp-linux

Table 39: Performance of the *overlap* operation on fBrain-vs-exons

| Library | Min (s) | Max (s) | Mean (s) | Speedup |
|---|---|---|---|---|
| polars-bio | 0.045943 | 0.064732 | 0.054234 | 1.66x |
| Bioframe | 0.084137 | 0.099481 | 0.090107 | 1.00x |
| PyRanges0 | 0.056206 | 0.065654 | 0.061844 | 1.46x |
| PyRanges1 | 0.09908 | 0.119018 | 0.106228 | 0.85x |
| pybedtools | 0.38246 | 0.406379 | 0.39153 | 0.23x |
| GenomicRanges | 1.19939 | 1.224621 | 1.208255 | 0.07x |

Table 40: Performance of the *nearest* operation on fBrain-vs-exons

| Library | Min (s) | Max (s) | Mean (s) | Speedup |
|---|---|---|---|---|
| polars-bio | 0.057012 | 0.073822 | 0.064665 | 2.49x |
| Bioframe | 0.158764 | 0.165707 | 0.161273 | 1.00x |
| PyRanges0 | 0.172297 | 0.176259 | 0.17363 | 0.93x |
| PyRanges1 | 0.217619 | 0.234088 | 0.22335 | 0.72x |
| pybedtools | 0.845945 | 0.84898 | 0.847447 | 0.19x |

Table 41: Performance of the *count overlaps* operation on fBrain-vs-exons

| Library | Min (s) | Max (s) | Mean (s) | Speedup |
|---|---|---|---|---|
| polars-bio | 0.035631 | 0.043555 | 0.04066 | 2.74x |
| Bioframe | 0.108015 | 0.116522 | 0.111266 | 1.00x |
| PyRanges0 | 0.077336 | 0.080282 | 0.07844 | 1.42x |
| PyRanges1 | 0.100883 | 0.106671 | 0.103181 | 1.08x |
| pybedtools | 0.745958 | 0.759006 | 0.754393 | 0.15x |
| GenomicRanges | 1.154942 | 1.164158 | 1.158506 | 0.10x |

Table 42: Performance of the *coverage* operation on fBrain-vs-exons

| Library | Min (s) | Max (s) | Mean (s) | Speedup |
|---|---|---|---|---|
| polars-bio | 0.036476 | 0.040001 | 0.037897 | 5.10x |
| Bioframe | 0.189201 | 0.20046 | 0.193401 | 1.00x |
| PyRanges0 | 0.141659 | 0.14424 | 0.143188 | 1.35x |
| PyRanges1 | 0.206033 | 0.224902 | 0.213089 | 0.91x |
| pybedtools | 0.773732 | 0.780424 | 0.776934 | 0.25x |
| GenomicRanges | 1.186341 | 1.194172 | 1.189255 | 0.16x |

Table 43: Performance of the *overlap* operation on ex-rna-vs-ex-anno

| Library | Min (s) | Max (s) | Mean (s) | Speedup |
|---|---|---|---|---|
| polars-bio | 6.235223 | 9.61441 | 7.723144 | 6.54x |
| Bioframe | 50.319263 | 50.956633 | 50.537202 | 1.00x |
| PyRanges0 | 36.371926 | 36.581642 | 36.448645 | 1.39x |
| PyRanges1 | 63.336711 | 63.455435 | 63.40654 | 0.80x |
| pybedtools | 1149.001487 | 1152.127068 | 1150.070659 | 0.04x |
| GenomicRanges | 597.951648 | 599.960895 | 599.002871 | 0.08x |

Table 44: Performance of the *nearest* operation on ex-rna-vs-ex-anno

| Library | Min (s) | Max (s) | Mean (s) | Speedup |
|---|---|---|---|---|
| polars-bio | 3.576373 | 3.679698 | 3.633697 | 15.54x |
| Bioframe | 56.301865 | 56.776617 | 56.464305 | 1.00x |
| PyRanges0 | 2.45308 | 2.60494 | 2.505172 | 22.54x |

Table 44: Performance of the *nearest* operation on ex-rna-vs-ex-anno

| Library | Min (s) | Max (s) | Mean (s) | Speedup |
|---|---|---|---|---|
| PyRanges1 | 4.975662 | 5.011008 | 4.997007 | 11.30x |
| pybedtools | 44.181913 | 44.79409 | 44.386971 | 1.27x |

Table 45: Performance of the *count overlaps* operation on ex-rna-vs-ex-anno

| Library | Min (s) | Max (s) | Mean (s) | Speedup |
|---|---|---|---|---|
| polars-bio | 2.052196 | 2.104447 | 2.075706 | 38.15x |
| Bioframe | 79.174164 | 79.234115 | 79.194209 | 1.00x |
| PyRanges0 | 18.797436 | 18.851941 | 18.824498 | 4.21x |
| PyRanges1 | 20.399172 | 20.436149 | 20.418562 | 3.88x |
| pybedtools | 35.850631 | 36.142479 | 36.041115 | 2.20x |
| GenomicRanges | 612.985873 | 613.52087 | 613.229997 | 0.13x |

Table 46: Performance of the *coverage* operation on ex-rna-vs-ex-anno

| Library | Min (s) | Max (s) | Mean (s) | Speedup |
|---|---|---|---|---|
| polars-bio | 1.829478 | 1.838981 | 1.834999 | 15.44x |
| Bioframe | 28.29136 | 28.361417 | 28.326821 | 1.00x |
| PyRanges0 | 18.611247 | 20.021441 | 19.473105 | 1.45x |
| PyRanges1 | 22.118838 | 22.210733 | 22.161329 | 1.28x |
| pybedtools | 74.477086 | 74.868659 | 74.618066 | 0.38x |
| GenomicRanges | 623.865655 | 623.94955 | 623.896645 | 0.05x |

Table 47: Performance of the *overlap* operation on 100-1p

| Library | Min (s) | Max (s) | Mean (s) | Speedup |
|---|---|---|---|---|
| polars-bio | 0.004992 | 0.01456 | 0.008429 | 0.47x |
| Bioframe | 0.003258 | 0.005112 | 0.00392 | 1.00x |
| PyRanges0 | 0.002368 | 0.003408 | 0.002777 | 1.41x |
| PyRanges1 | 0.005606 | 0.006547 | 0.005975 | 0.66x |
| pybedtools | 0.005909 | 0.006483 | 0.006194 | 0.63x |
| GenomicRanges | 0.003124 | 0.003404 | 0.003233 | 1.21x |
| PyGenomics | 0.000777 | 0.000879 | 0.000818 | 4.79x |

Table 48: Performance of the *nearest* operation on 100-1p

| Library | Min (s) | Max (s) | Mean (s) | Speedup |
|---|---|---|---|---|
| polars-bio | 0.039758 | 0.157059 | 0.082499 | 0.06x |
| Bioframe | 0.004496 | 0.005139 | 0.004808 | 1.00x |
| PyRanges1 | 0.005232 | 0.006285 | 0.005613 | 0.86x |
| pybedtools | 0.002655 | 0.002957 | 0.002758 | 1.74x |

Table 49: Performance of the *count overlaps* operation on 100-1p

| Library | Min (s) | Max (s) | Mean (s) | Speedup |
|---|---|---|---|---|
| polars-bio | 0.004349 | 0.072032 | 0.027109 | 0.16x |
| Bioframe | 0.003966 | 0.004761 | 0.004247 | 1.00x |
| PyRanges0 | 0.002885 | 0.00314 | 0.002973 | 1.43x |
| PyRanges1 | 0.004525 | 0.004943 | 0.004694 | 0.90x |
| pybedtools | 0.002502 | 0.002934 | 0.0027 | 1.57x |
| GenomicRanges | 0.003229 | 0.003376 | 0.003278 | 1.30x |

Table 50: Performance of the *coverage* operation on 100-1p

| Library | Min (s) | Max (s) | Mean (s) | Speedup |
|---|---|---|---|---|
| polars-bio | 0.004251 | 0.062087 | 0.0237 | 0.37x |
| Bioframe | 0.007449 | 0.011114 | 0.008755 | 1.00x |
| PyRanges1 | 0.010586 | 0.012078 | 0.011134 | 0.79x |
| pybedtools | 0.002555 | 0.002829 | 0.002686 | 3.26x |

Table 51: Performance of the *overlap* operation on 1000-1p

| Library | Min (s) | Max (s) | Mean (s) | Speedup |
|---|---|---|---|---|
| polars-bio | 0.005234 | 0.008876 | 0.006523 | 0.77x |
| Bioframe | 0.004581 | 0.005864 | 0.005016 | 1.00x |
| PyRanges0 | 0.003191 | 0.003455 | 0.003296 | 1.52x |
| PyRanges1 | 0.008031 | 0.008103 | 0.008074 | 0.62x |
| pybedtools | 0.053782 | 0.054005 | 0.053929 | 0.09x |
| GenomicRanges | 0.032026 | 0.032674 | 0.032265 | 0.16x |
| PyGenomics | 0.010626 | 0.01142 | 0.010918 | 0.46x |

Table 52: Performance of the *nearest* operation on 1000-1p

| Library | Min (s) | Max (s) | Mean (s) | Speedup |
|---|---|---|---|---|
| polars-bio | 0.005791 | 0.006665 | 0.006123 | 1.01x |
| Bioframe | 0.005982 | 0.006628 | 0.00621 | 1.00x |
| PyRanges0 | 0.006279 | 0.006752 | 0.006447 | 0.96x |
| PyRanges1 | 0.009039 | 0.009504 | 0.009217 | 0.67x |
| pybedtools | 0.007826 | 0.007978 | 0.007917 | 0.78x |

Table 53: Performance of the *count overlaps* operation on 1000-1p

| Library | Min (s) | Max (s) | Mean (s) | Speedup |
|---|---|---|---|---|
| polars-bio | 0.004343 | 0.007692 | 0.005481 | 0.98x |
| Bioframe | 0.005139 | 0.005735 | 0.005359 | 1.00x |
| PyRanges1 | 0.005589 | 0.005976 | 0.005719 | 0.94x |
| pybedtools | 0.01436 | 0.014635 | 0.014456 | 0.37x |
| GenomicRanges | 0.032931 | 0.03307 | 0.033016 | 0.16x |

Table 54: Performance of the *coverage* operation on 1000-1p

| Library | Min (s) | Max (s) | Mean (s) | Speedup |
|---|---|---|---|---|
| polars-bio | 0.004259 | 0.005305 | 0.004947 | 2.08x |
| Bioframe | 0.009969 | 0.010782 | 0.010297 | 1.00x |
| PyRanges1 | 0.011982 | 0.012304 | 0.012103 | 0.85x |
| pybedtools | 0.014775 | 0.015246 | 0.014956 | 0.69x |

Table 55: Performance of the *overlap* operation on 10000-1p

| Library | Min (s) | Max (s) | Mean (s) | Speedup |
|---|---|---|---|---|
| polars-bio | 0.01015 | 0.018572 | 0.013027 | 1.84x |
| Bioframe | 0.02268 | 0.025605 | 0.023968 | 1.00x |
| PyRanges0 | 0.016065 | 0.018936 | 0.017143 | 1.40x |
| PyRanges1 | 0.030509 | 0.031181 | 0.030868 | 0.78x |
| pybedtools | 1.335037 | 1.358509 | 1.345311 | 0.02x |
| GenomicRanges | 0.322956 | 0.326403 | 0.324169 | 0.07x |
| PyGenomics | 0.136783 | 0.141169 | 0.13853 | 0.17x |

Table 56: Performance of the *nearest* operation on 10000-1p

| Library | Min (s) | Max (s) | Mean (s) | Speedup |
|---|---|---|---|---|
| polars-bio | 0.009616 | 0.011293 | 0.010447 | 3.08x |
| Bioframe | 0.031761 | 0.032938 | 0.032167 | 1.00x |
| PyRanges0 | 0.010939 | 0.011387 | 0.01109 | 2.90x |
| PyRanges1 | 0.015275 | 0.015676 | 0.015419 | 2.09x |
| pybedtools | 0.059244 | 0.059899 | 0.059542 | 0.54x |

Table 57: Performance of the *count overlaps* operation on 10000-1p

| Library | Min (s) | Max (s) | Mean (s) | Speedup |
|---|---|---|---|---|
| polars-bio | 0.007028 | 0.007725 | 0.007363 | 2.50x |
| Bioframe | 0.018051 | 0.019179 | 0.018436 | 1.00x |
| PyRanges1 | 0.014252 | 0.014683 | 0.014423 | 1.28x |
| pybedtools | 0.926946 | 1.012523 | 0.973852 | 0.02x |
| GenomicRanges | 0.330064 | 0.33175 | 0.331123 | 0.06x |

Table 58: Performance of the *coverage* operation on 10000-1p

| Library | Min (s) | Max (s) | Mean (s) | Speedup |
|---|---|---|---|---|
| polars-bio | 0.005994 | 0.006967 | 0.006396 | 2.78x |
| Bioframe | 0.017402 | 0.018389 | 0.017779 | 1.00x |
| PyRanges1 | 0.022651 | 0.023034 | 0.022779 | 0.78x |
| pybedtools | 0.952175 | 1.000698 | 0.97678 | 0.02x |

Table 59: Performance of the *overlap* operation on 100000-1p

| Library | Min (s) | Max (s) | Mean (s) | Speedup |
|---|---|---|---|---|
| polars-bio | 0.059271 | 0.08788 | 0.070483 | 3.02x |
| Bioframe | 0.209074 | 0.218512 | 0.21252 | 1.00x |
| PyRanges0 | 0.144653 | 0.164863 | 0.151749 | 1.40x |
| PyRanges1 | 0.228314 | 0.247017 | 0.234636 | 0.91x |
| pybedtools | 19.263571 | 19.313483 | 19.286741 | 0.01x |
| GenomicRanges | 3.290473 | 3.294306 | 3.291987 | 0.06x |
| PyGenomics | 1.881858 | 1.924059 | 1.896222 | 0.11x |

Table 60: Performance of the *nearest* operation on 100000-1p

| Library | Min (s) | Max (s) | Mean (s) | Speedup |
|---|---|---|---|---|
| polars-bio | 0.054573 | 0.060741 | 0.057958 | 6.31x |
| Bioframe | 0.363422 | 0.368554 | 0.365524 | 1.00x |
| PyRanges0 | 0.062446 | 0.06448 | 0.06321 | 5.78x |
| PyRanges1 | 0.084614 | 0.086633 | 0.085545 | 4.27x |
| pybedtools | 0.570352 | 0.57555 | 0.572301 | 0.64x |

Table 61: Performance of the *count overlaps* operation on 100000-1p

| Library | Min (s) | Max (s) | Mean (s) | Speedup |
|---|---|---|---|---|
| polars-bio | 0.034675 | 0.047883 | 0.039593 | 7.85x |
| Bioframe | 0.309819 | 0.311936 | 0.310958 | 1.00x |
| PyRanges1 | 0.113469 | 0.114316 | 0.113866 | 2.73x |
| pybedtools | 15.265868 | 16.802575 | 16.206183 | 0.02x |
| GenomicRanges | 3.369224 | 3.374411 | 3.371411 | 0.09x |

Table 62: Performance of the *coverage* operation on 100000-1p

| Library | Min (s) | Max (s) | Mean (s) | Speedup |
|---|---|---|---|---|
| polars-bio | 0.02543 | 0.026808 | 0.026079 | 4.08x |
| Bioframe | 0.104575 | 0.1096 | 0.106393 | 1.00x |
| PyRanges1 | 0.147505 | 0.151673 | 0.149512 | 0.71x |
| pybedtools | 16.382024 | 17.619212 | 16.802475 | 0.01x |

Table 63: Performance of the *overlap* operation on 1000000-1p

| Library | Min (s) | Max (s) | Mean (s) | Speedup |
|---|---|---|---|---|
| polars-bio | 0.947673 | 1.10915 | 1.003667 | 2.49x |
| Bioframe | 2.490142 | 2.513556 | 2.499533 | 1.00x |
| PyRanges0 | 2.119717 | 2.178453 | 2.148959 | 1.16x |
| PyRanges1 | 3.274957 | 3.298976 | 3.288601 | 0.76x |

Table 64: Performance of the *nearest* operation on 1000000-1p

| Library | Min (s) | Max (s) | Mean (s) | Speedup |
|---|---|---|---|---|
| polars-bio | 0.771199 | 0.783292 | 0.777746 | 6.96x |
| Bioframe | 5.394265 | 5.434618 | 5.411728 | 1.00x |
| PyRanges0 | 0.874484 | 0.932857 | 0.901145 | 6.01x |
| PyRanges1 | 1.127032 | 1.149141 | 1.140538 | 4.74x |

Table 65: Performance of the *count overlaps* operation on 1000000-1p

| Library | Min (s) | Max (s) | Mean (s) | Speedup |
|---|---|---|---|---|
| polars-bio | 0.501775 | 0.530884 | 0.514401 | 8.01x |
| Bioframe | 4.117035 | 4.131015 | 4.121744 | 1.00x |
| PyRanges1 | 1.583204 | 1.678121 | 1.631619 | 2.53x |

Table 66: Performance of the *coverage* operation on 1000000-1p

| Library | Min (s) | Max (s) | Mean (s) | Speedup |
|---|---|---|---|---|
| polars-bio | 0.247626 | 0.266565 | 0.256522 | 4.86x |
| Bioframe | 1.243608 | 1.250394 | 1.246153 | 1.00x |
| PyRanges1 | 1.916323 | 2.005555 | 1.949487 | 0.64x |

Table 67: Performance of the *overlap* operation on 10000000-1p

| Library | Min (s) | Max (s) | Mean (s) | Speedup |
|---|---|---|---|---|
| polars-bio | 18.531273 | 18.806266 | 18.632293 | 1.62x |
| Bioframe | 30.074841 | 30.116671 | 30.097846 | 1.00x |
| PyRanges0 | 29.579651 | 30.536834 | 29.904783 | 1.01x |
| PyRanges1 | 42.196037 | 42.278681 | 42.232728 | 0.71x |

Table 68: Performance of the *nearest* operation on 10000000-1p

| Library | Min (s) | Max (s) | Mean (s) | Speedup |
|---|---|---|---|---|
| polars-bio | 14.43136 | 14.548645 | 14.496101 | 5.56x |
| Bioframe | 80.443039 | 80.705181 | 80.548879 | 1.00x |
| PyRanges0 | 13.64936 | 14.330292 | 13.882901 | 5.80x |
| PyRanges1 | 17.384461 | 17.654503 | 17.561143 | 4.59x |

Table 69: Performance of the *count overlaps* operation on 10000000-1p

| Library | Min (s) | Max (s) | Mean (s) | Speedup |
|---|---|---|---|---|
| polars-bio | 9.812223 | 9.925855 | 9.856571 | 6.23x |

Table 69: Performance of the *count overlaps* operation on 10000000-1p

| Library | Min (s) | Max (s) | Mean (s) | Speedup |
|---|---|---|---|---|
| Bioframe | 61.348815 | 61.558393 | 61.444649 | 1.00x |
| PyRanges1 | 24.969282 | 25.069392 | 25.029806 | 2.45x |

Table 70: Performance of the *coverage* operation on 10000000-1p

| Library | Min (s) | Max (s) | Mean (s) | Speedup |
|---|---|---|---|---|
| polars-bio | 3.188742 | 3.31096 | 3.235061 | 6.11x |
| Bioframe | 19.748385 | 19.802079 | 19.778009 | 1.00x |
| PyRanges1 | 30.304058 | 30.446378 | 30.353857 | 0.65x |

### 3.3.3 Multi-thread results

This section presents detailed parallel scaling results for selected dataset pairs. For the *real-world* benchmark, we focus on `ex-rna-vs-ex-anno` as a representative large pair. For the *synthetic* benchmark, we report scaling on two large configurations: `1000000-8p` and `10000000-8p`. For each operation, we show performance with 1, 2, 4, 6, and 8 threads to quantify speedups and scalability.

#### 3.3.3.1 apple-m3-max

Table 71: Performance of the *overlap* operation on ex-rna-vs-ex-anno-8p

| Library | Min (s) | Max (s) | Mean (s) | Speedup |
|---|---|---|---|---|
| polars-bio | 3.247022 | 3.803021 | 3.370889 | 1.00x |
| polars-bio-2 | 1.798569 | 1.848162 | 1.811417 | 1.86x |
| polars-bio-4 | 1.140229 | 1.158243 | 1.147355 | 2.94x |
| polars-bio-6 | 0.959703 | 0.968725 | 0.962915 | 3.50x |
| polars-bio-8 | 0.694637 | 0.710492 | 0.701048 | 4.81x |

Table 72: Performance of the *nearest* operation on ex-rna-vs-ex-anno-8p

| Library | Min (s) | Max (s) | Mean (s) | Speedup |
|---|---|---|---|---|
| polars-bio | 2.186354 | 2.248171 | 2.220822 | 1.00x |
| polars-bio-2 | 1.162969 | 1.222115 | 1.187505 | 1.87x |
| polars-bio-4 | 0.708508 | 0.735763 | 0.720115 | 3.08x |
| polars-bio-6 | 0.632877 | 0.652955 | 0.642816 | 3.45x |
| polars-bio-8 | 0.456674 | 0.476473 | 0.465284 | 4.77x |

Table 73: Performance of the *count overlaps* operation on ex-rna-vs-ex-anno-8p

| Library | Min (s) | Max (s) | Mean (s) | Speedup |
|---|---|---|---|---|
| polars-bio | 1.502551 | 1.534006 | 1.515078 | 1.00x |
| polars-bio-2 | 0.811236 | 0.821365 | 0.815682 | 1.86x |
| polars-bio-4 | 0.440628 | 0.46778 | 0.455358 | 3.33x |
| polars-bio-6 | 0.331317 | 0.338207 | 0.334638 | 4.53x |
| polars-bio-8 | 0.280465 | 0.282707 | 0.281311 | 5.39x |

Table 74: Performance of the *coverage* operation on ex-rna-vs-ex-anno-8p

| Library | Min (s) | Max (s) | Mean (s) | Speedup |
|---|---|---|---|---|
| polars-bio | 1.181806 | 1.185549 | 1.183889 | 1.00x |
| polars-bio-2 | 0.644288 | 0.645076 | 0.644587 | 1.84x |
| polars-bio-4 | 0.362752 | 0.363411 | 0.363036 | 3.26x |
| polars-bio-6 | 0.258583 | 0.272702 | 0.264111 | 4.48x |
| polars-bio-8 | 0.222888 | 0.234884 | 0.229052 | 5.17x |

Table 75: Performance of the *overlap* operation on 1000000-8p

| Library | Min (s) | Max (s) | Mean (s) | Speedup |
|---|---|---|---|---|
| polars-bio | 0.468442 | 0.523065 | 0.494609 | 1.00x |
| polars-bio-2 | 0.262861 | 0.26828 | 0.265028 | 1.87x |
| polars-bio-4 | 0.1629 | 0.166657 | 0.164536 | 3.01x |
| polars-bio-6 | 0.137724 | 0.146893 | 0.143772 | 3.44x |
| polars-bio-8 | 0.111952 | 0.11465 | 0.113521 | 4.36x |

Table 76: Performance of the *nearest* operation on 1000000-8p

| Library | Min (s) | Max (s) | Mean (s) | Speedup |
|---|---|---|---|---|
| polars-bio | 0.393067 | 0.415076 | 0.404032 | 1.00x |
| polars-bio-2 | 0.234559 | 0.235746 | 0.235051 | 1.72x |
| polars-bio-4 | 0.158996 | 0.167352 | 0.16349 | 2.47x |
| polars-bio-6 | 0.14634 | 0.14935 | 0.148215 | 2.73x |
| polars-bio-8 | 0.125472 | 0.128158 | 0.126606 | 3.19x |

Table 77: Performance of the *count overlaps* operation on 1000000-8p

| Library | Min (s) | Max (s) | Mean (s) | Speedup |
|---|---|---|---|---|
| polars-bio | 0.267875 | 0.296727 | 0.277677 | 1.00x |
| polars-bio-2 | 0.163662 | 0.170045 | 0.165917 | 1.67x |
| polars-bio-4 | 0.111136 | 0.114835 | 0.112891 | 2.46x |
| polars-bio-6 | 0.097944 | 0.104607 | 0.101477 | 2.74x |

Table 77: Performance of the *count overlaps* operation on 1000000-8p

| Library | Min (s) | Max (s) | Mean (s) | Speedup |
|---|---|---|---|---|
| polars-bio-8 | 0.099474 | 0.117493 | 0.106059 | 2.62x |

Table 78: Performance of the *coverage* operation on 1000000-8p

| Library | Min (s) | Max (s) | Mean (s) | Speedup |
|---|---|---|---|---|
| polars-bio | 0.128377 | 0.131261 | 0.129598 | 1.00x |
| polars-bio-2 | 0.081762 | 0.085104 | 0.08324 | 1.56x |
| polars-bio-4 | 0.064151 | 0.066197 | 0.064851 | 2.00x |
| polars-bio-6 | 0.066926 | 0.06892 | 0.06768 | 1.91x |
| polars-bio-8 | 0.072767 | 0.074339 | 0.073589 | 1.76x |

Table 79: Performance of the *overlap* operation on 10000000-8p

| Library | Min (s) | Max (s) | Mean (s) | Speedup |
|---|---|---|---|---|
| polars-bio | 9.081732 | 9.388126 | 9.203018 | 1.00x |
| polars-bio-2 | 4.696455 | 4.912478 | 4.793254 | 1.92x |
| polars-bio-4 | 2.885023 | 2.902893 | 2.896218 | 3.18x |
| polars-bio-6 | 2.196605 | 2.217945 | 2.209839 | 4.16x |
| polars-bio-8 | 1.813586 | 1.860947 | 1.833498 | 5.02x |

Table 80: Performance of the *nearest* operation on 10000000-8p

| Library | Min (s) | Max (s) | Mean (s) | Speedup |
|---|---|---|---|---|
| polars-bio | 7.299887 | 7.659385 | 7.495962 | 1.00x |
| polars-bio-2 | 4.01928 | 4.158504 | 4.069511 | 1.84x |
| polars-bio-4 | 2.683383 | 2.720981 | 2.704975 | 2.77x |
| polars-bio-6 | 2.141075 | 2.162109 | 2.150595 | 3.49x |
| polars-bio-8 | 1.859186 | 1.865634 | 1.862653 | 4.02x |

Table 81: Performance of the *count overlaps* operation on 10000000-8p

| Library | Min (s) | Max (s) | Mean (s) | Speedup |
|---|---|---|---|---|
| polars-bio | 5.30938 | 5.450332 | 5.381068 | 1.00x |
| polars-bio-2 | 2.893766 | 2.91378 | 2.906401 | 1.85x |
| polars-bio-4 | 1.748771 | 1.797485 | 1.768895 | 3.04x |
| polars-bio-6 | 1.352671 | 1.385655 | 1.369312 | 3.93x |
| polars-bio-8 | 1.178559 | 1.199971 | 1.192577 | 4.51x |

Table 82: Performance of the *coverage* operation on 10000000-8p

| Library | Min (s) | Max (s) | Mean (s) | Speedup |
|---|---|---|---|---|
| polars-bio | 1.638818 | 1.678156 | 1.655573 | 1.00x |
| polars-bio-2 | 0.994195 | 0.996554 | 0.995701 | 1.66x |
| polars-bio-4 | 0.678722 | 0.701234 | 0.689151 | 2.40x |
| polars-bio-6 | 0.620289 | 0.662175 | 0.639026 | 2.59x |
| polars-bio-8 | 0.570659 | 0.582937 | 0.57688 | 2.87x |

#### 3.3.3.2 gcp-linux

Table 83: Performance of the *overlap* operation on ex-rna-vs-ex-anno-8p

| Library | Min (s) | Max (s) | Mean (s) | Speedup |
|---|---|---|---|---|
| polars-bio | 6.325617 | 8.185275 | 7.005925 | 1.00x |
| polars-bio-2 | 3.920645 | 4.617084 | 4.198055 | 1.67x |
| polars-bio-4 | 3.036273 | 3.060781 | 3.0452 | 2.30x |
| polars-bio-6 | 2.127994 | 2.134505 | 2.131016 | 3.29x |
| polars-bio-8 | 1.731485 | 1.789347 | 1.752986 | 4.00x |

Table 84: Performance of the *nearest* operation on ex-rna-vs-ex-anno-8p

| Library | Min (s) | Max (s) | Mean (s) | Speedup |
|---|---|---|---|---|
| polars-bio | 4.047329 | 4.439016 | 4.198198 | 1.00x |
| polars-bio-2 | 2.624132 | 2.722843 | 2.682361 | 1.57x |
| polars-bio-4 | 1.809028 | 1.917798 | 1.871763 | 2.24x |
| polars-bio-6 | 1.309557 | 1.362131 | 1.333989 | 3.15x |
| polars-bio-8 | 1.066945 | 1.113168 | 1.087907 | 3.86x |

Table 85: Performance of the *count overlaps* operation on ex-rna-vs-ex-anno-8p

| Library | Min (s) | Max (s) | Mean (s) | Speedup |
|---|---|---|---|---|
| polars-bio | 2.426441 | 2.456318 | 2.439266 | 1.00x |
| polars-bio-2 | 1.22516 | 1.272066 | 1.245401 | 1.96x |
| polars-bio-4 | 0.711421 | 0.744023 | 0.724315 | 3.37x |
| polars-bio-6 | 0.563797 | 0.607321 | 0.580574 | 4.20x |
| polars-bio-8 | 0.459308 | 0.493886 | 0.479126 | 5.09x |

Table 86: Performance of the *coverage* operation on ex-rna-vs-ex-anno-8p

| Library | Min (s) | Max (s) | Mean (s) | Speedup |
|---|---|---|---|---|
| polars-bio | 2.212958 | 2.23035 | 2.222531 | 1.00x |

Table 86: Performance of the *coverage* operation on ex-rna-vs-ex-anno-8p

| Library | Min (s) | Max (s) | Mean (s) | Speedup |
|---|---|---|---|---|
| polars-bio-2 | 1.132056 | 1.15405 | 1.146413 | 1.94x |
| polars-bio-4 | 0.645737 | 0.661564 | 0.652277 | 3.41x |
| polars-bio-6 | 0.50589 | 0.511256 | 0.50839 | 4.37x |
| polars-bio-8 | 0.439503 | 0.450924 | 0.447075 | 4.97x |

Table 87: Performance of the *overlap* operation on 1000000-8p

| Library | Min (s) | Max (s) | Mean (s) | Speedup |
|---|---|---|---|---|
| polars-bio | 0.903831 | 1.098046 | 0.974229 | 1.00x |
| polars-bio-2 | 0.50099 | 0.512259 | 0.504852 | 1.93x |
| polars-bio-4 | 0.300453 | 0.328605 | 0.318188 | 3.06x |
| polars-bio-6 | 0.257792 | 0.278203 | 0.268718 | 3.63x |
| polars-bio-8 | 0.22321 | 0.243244 | 0.230621 | 4.22x |

Table 88: Performance of the *nearest* operation on 1000000-8p

| Library | Min (s) | Max (s) | Mean (s) | Speedup |
|---|---|---|---|---|
| polars-bio | 0.758815 | 0.78919 | 0.769877 | 1.00x |
| polars-bio-2 | 0.465192 | 0.47484 | 0.468824 | 1.64x |
| polars-bio-4 | 0.332101 | 0.336953 | 0.334461 | 2.30x |
| polars-bio-6 | 0.276071 | 0.29266 | 0.281794 | 2.73x |
| polars-bio-8 | 0.237269 | 0.263256 | 0.254046 | 3.03x |

Table 89: Performance of the *count overlaps* operation on 1000000-8p

| Library | Min (s) | Max (s) | Mean (s) | Speedup |
|---|---|---|---|---|
| polars-bio | 0.496938 | 0.517659 | 0.505043 | 1.00x |
| polars-bio-2 | 0.295325 | 0.313859 | 0.302686 | 1.67x |
| polars-bio-4 | 0.194371 | 0.20433 | 0.200853 | 2.51x |
| polars-bio-6 | 0.175505 | 0.181913 | 0.178222 | 2.83x |
| polars-bio-8 | 0.15672 | 0.163036 | 0.160701 | 3.14x |

Table 90: Performance of the *coverage* operation on 1000000-8p

| Library | Min (s) | Max (s) | Mean (s) | Speedup |
|---|---|---|---|---|
| polars-bio | 0.245895 | 0.250118 | 0.247479 | 1.00x |
| polars-bio-2 | 0.167378 | 0.173578 | 0.171251 | 1.45x |
| polars-bio-4 | 0.122749 | 0.126635 | 0.124491 | 1.99x |
| polars-bio-6 | 0.11385 | 0.119157 | 0.116185 | 2.13x |
| polars-bio-8 | 0.108127 | 0.110327 | 0.10942 | 2.26x |

Table 91: Performance of the *overlap* operation on 10000000-8p

| Library | Min (s) | Max (s) | Mean (s) | Speedup |
|---|---|---|---|---|
| polars-bio | 18.215782 | 19.091392 | 18.510207 | 1.00x |
| polars-bio-2 | 9.399565 | 9.680242 | 9.566631 | 1.93x |
| polars-bio-4 | 5.303647 | 5.555487 | 5.442898 | 3.40x |
| polars-bio-6 | 4.022274 | 4.066371 | 4.051045 | 4.57x |
| polars-bio-8 | 3.369559 | 3.416123 | 3.388564 | 5.46x |

Table 92: Performance of the *nearest* operation on 10000000-8p

| Library | Min (s) | Max (s) | Mean (s) | Speedup |
|---|---|---|---|---|
| polars-bio | 14.325736 | 14.444885 | 14.39027 | 1.00x |
| polars-bio-2 | 8.095907 | 8.178189 | 8.136852 | 1.77x |
| polars-bio-4 | 5.096407 | 5.15379 | 5.122893 | 2.81x |
| polars-bio-6 | 3.986362 | 4.205706 | 4.128561 | 3.49x |
| polars-bio-8 | 3.491618 | 3.711814 | 3.577309 | 4.02x |

Table 93: Performance of the *count overlaps* operation on 10000000-8p

| Library | Min (s) | Max (s) | Mean (s) | Speedup |
|---|---|---|---|---|
| polars-bio | 9.701679 | 9.796148 | 9.740035 | 1.00x |
| polars-bio-2 | 5.346433 | 5.399117 | 5.370757 | 1.81x |
| polars-bio-4 | 3.150557 | 3.203458 | 3.178719 | 3.06x |
| polars-bio-6 | 2.485947 | 2.56386 | 2.52768 | 3.85x |
| polars-bio-8 | 2.156472 | 2.176608 | 2.163483 | 4.50x |

Table 94: Performance of the *coverage* operation on 10000000-8p

| Library | Min (s) | Max (s) | Mean (s) | Speedup |
|---|---|---|---|---|
| polars-bio | 3.091184 | 3.216964 | 3.135982 | 1.00x |
| polars-bio-2 | 1.998423 | 2.041581 | 2.01331 | 1.56x |
| polars-bio-4 | 1.412483 | 1.45218 | 1.426102 | 2.20x |
| polars-bio-6 | 1.281432 | 1.328666 | 1.301256 | 2.41x |
| polars-bio-8 | 1.176944 | 1.193294 | 1.18414 | 2.65x |

#### 3.3.4 End to end pipelines results

Results for an end-to-end test with calculating overlaps, nearest, coverage and count overlaps and saving results to a CSV file.

Note:

1. Please note that in case of `PyRanges0` we were unable to export the results of *coverage* and *count-overlaps* operations to a CSV file, so the results are not presented here.
2. `pb_stream` denotes polars-bio in the streaming mode.

#### 3.3.4.1 apple-m3-max

Table 95: Performance and peak memory usage of the *overlap* operation on fBrain-vs-exons

| Library | Min (s) | Max (s) | Mean (s) | Speedup | Peak memory (MB) |
|---|---|---|---|---|---|
| polars-bio | 0.042378 | 0.130957 | 0.071929 | 3.10x | 285.468 |
| pb_stream | 0.035498 | 0.037438 | 0.036653 | 6.09x | 274.093 |
| Bioframe | 0.208548 | 0.251457 | 0.223219 | 1.00x | 300.75 |
| PyRanges0 | 0.409707 | 0.415361 | 0.412135 | 0.54x | 329.968 |
| PyRanges1 | 0.47518 | 0.491508 | 0.482739 | 0.46x | 324.468 |

Table 96: Performance and peak memory usage of the *nearest* operation on fBrain-vs-exons

| Library | Min (s) | Max (s) | Mean (s) | Speedup | Peak memory (MB) |
|---|---|---|---|---|---|
| polars-bio | 0.053349 | 0.058382 | 0.055362 | 9.14x | 321.062 |
| pb_stream | 0.051385 | 0.053979 | 0.052764 | 9.59x | 311.422 |
| Bioframe | 0.503887 | 0.510257 | 0.506123 | 1.00x | 316.969 |
| PyRanges0 | 1.135469 | 1.183369 | 1.151801 | 0.44x | 364.594 |
| PyRanges1 | 1.327935 | 1.334101 | 1.331346 | 0.38x | 357.734 |

Table 97: Performance and peak memory usage of the *coverage* operation on fBrain-vs-exons

| Library | Min (s) | Max (s) | Mean (s) | Speedup | Peak memory (MB) |
|---|---|---|---|---|---|
| polars-bio | 0.034756 | 0.038718 | 0.036421 | 13.40x | 290.078 |
| pb_stream | 0.03607 | 0.037332 | 0.036534 | 13.35x | 274.344 |
| Bioframe | 0.48449 | 0.492328 | 0.487891 | 1.00x | 419.312 |
| PyRanges1 | 0.971084 | 0.980085 | 0.975012 | 0.50x | 407.562 |

Table 98: Performance and peak memory usage of the *count overlaps* operation on fBrain-vs-exons

| Library | Min (s) | Max (s) | Mean (s) | Speedup | Peak memory (MB) |
|---|---|---|---|---|---|
| polars-bio | 0.03452 | 0.037714 | 0.035927 | 9.27x | 294.266 |
| pb_stream | 0.035863 | 0.036756 | 0.036414 | 9.14x | 278.438 |
| Bioframe | 0.328145 | 0.338734 | 0.332951 | 1.00x | 306.234 |
| PyRanges1 | 0.532739 | 0.544914 | 0.538646 | 0.62x | 328.328 |

Table 99: Performance and peak memory usage of the *overlap* operation on ex-rna-vs-ex-anno

| Library | Min (s) | Max (s) | Mean (s) | Speedup | Peak memory (MB) |
|---|---|---|---|---|---|
| polars-bio | 22.781745 | 23.916568 | 23.161559 | 16.64x | 14677.0468 |
| pb_stream | 18.501279 | 18.797602 | 18.676707 | 20.63x | 555.109 |
| Bioframe | 383.108514 | 387.500069 | 385.309331 | 1.00x | 33806.062 |
| PyRanges0 | 276.421312 | 279.839508 | 277.845198 | 1.39x | 29777.312 |
| PyRanges1 | 355.703878 | 367.680249 | 360.875151 | 1.07x | 34526.859 |

Table 100: Performance and peak memory usage of the *nearest* operation on ex-rna-vs-ex-anno

| Library | Min (s) | Max (s) | Mean (s) | Speedup | Peak memory (MB) |
|---|---|---|---|---|---|
| polars-bio | 2.597955 | 2.760537 | 2.674482 | 32.02x | 1060.031 |
| pb_stream | 2.65088 | 2.685157 | 2.665171 | 32.13x | 560.453 |
| Bioframe | 85.238305 | 86.131916 | 85.644961 | 1.00x | 6894.062 |
| PyRanges0 | 13.530549 | 13.705834 | 13.620471 | 6.29x | 3031.797 |
| PyRanges1 | 16.290782 | 16.385961 | 16.322671 | 5.25x | 3509.984 |

Table 101: Performance and peak memory usage of the *coverage* operation on ex-rna-vs-ex-anno

| Library | Min (s) | Max (s) | Mean (s) | Speedup | Peak memory (MB) |
|---|---|---|---|---|---|
| polars-bio | 1.523833 | 1.555472 | 1.541038 | 21.41x | 717.984 |
| pb_stream | 1.336613 | 1.397324 | 1.364051 | 24.19x | 411.703 |
| Bioframe | 32.294844 | 33.421618 | 32.99334 | 1.00x | 16651.922 |
| PyRanges1 | 26.382409 | 27.382901 | 27.020202 | 1.22x | 6119.125 |

Table 102: Performance and peak memory usage of the *count overlaps* operation on ex-rna-vs-ex-anno

| Library | Min (s) | Max (s) | Mean (s) | Speedup | Peak memory (MB) |
|---|---|---|---|---|---|
| polars-bio | 1.806838 | 1.845584 | 1.82594 | 54.33x | 729.078 |
| pb_stream | 1.681187 | 1.767811 | 1.714943 | 57.85x | 416.094 |
| Bioframe | 97.91802 | 101.736351 | 99.210461 | 1.00x | 23029.219 |
| PyRanges1 | 19.498264 | 19.676838 | 19.561322 | 5.07x | 5270.234 |

Table 103: Performance and peak memory usage of the *overlap* operation on 100-1p

| Library | Min (s) | Max (s) | Mean (s) | Speedup | Peak memory (MB) |
|---|---|---|---|---|---|
| polars-bio | 0.009118 | 0.077181 | 0.032054 | 1.55x | 248.594 |
| pb_stream g | 0.003382 | 0.004769 | 0.003853 | 12.92x | 247.562 |
| Bioframe | 0.030154 | 0.088667 | 0.049769 | 1.00x | 231.641 |
| PyRanges0 | 0.045764 | 0.051035 | 0.047857 | 1.04x | 228.516 |
| PyRanges1 | 0.053751 | 0.072545 | 0.060221 | 0.83x | 228.609 |

Table 104: Performance and peak memory usage of the *nearest* operation on 100-1p

| Library | Min (s) | Max (s) | Mean (s) | Speedup | Peak memory (MB) |
|---|---|---|---|---|---|
| polars-bio | 0.009145 | 0.038799 | 0.019201 | 2.24x | 253.156 |
| pb_stream | 0.003964 | 0.005051 | 0.004504 | 9.53x | 248.188 |
| Bioframe | 0.033372 | 0.061107 | 0.042931 | 1.00x | 229.906 |
| PyRanges0 | 0.049586 | 0.057381 | 0.052364 | 0.82x | 231.812 |
| PyRanges1 | 0.054496 | 0.059205 | 0.056362 | 0.76x | 231.688 |

Table 105: Performance and peak memory usage of the *coverage* operation on 100-1p

| Library | Min (s) | Max (s) | Mean (s) | Speedup | Peak memory (MB) |
|---|---|---|---|---|---|
| polars-bio | 0.005492 | 0.020652 | 0.012584 | 5.20x | 245.578 |
| pb_stream | 0.003059 | 0.003746 | 0.003397 | 19.25x | 243.5 |
| Bioframe | 0.060684 | 0.074157 | 0.065378 | 1.00x | 230.953 |
| PyRanges1 | 0.093668 | 0.096265 | 0.094567 | 0.69x | 243.5 |

Table 106: Performance and peak memory usage of the *count overlaps* operation on 100-1p

| Library | Min (s) | Max (s) | Mean (s) | Speedup | Peak memory (MB) |
|---|---|---|---|---|---|
| polars-bio | 0.005291 | 0.008843 | 0.006568 | 5.53x | 249.406 |
| pb_stream | 0.003279 | 0.003697 | 0.003447 | 10.53x | 245.672 |
| Bioframe | 0.032914 | 0.042309 | 0.036302 | 1.00x | 234.141 |
| PyRanges1 | 0.045085 | 0.045477 | 0.045224 | 0.80x | 232.703 |

Table 107: Performance and peak memory usage of the *overlap* operation on 10000000-1p

| Library | Min (s) | Max (s) | Mean (s) | Speedup | Peak memory (MB) |
|---|---|---|---|---|---|
| polars-bio | 11.109423 | 11.871893 | 11.397992 | 10.38x | 7064.312 |
| pb_stream | 12.049206 | 12.327491 | 12.191582 | 9.71x | 1505.109 |
| Bioframe | 117.701516 | 119.51073 | 118.356016 | 1.00x | 16380.234 |
| PyRanges0 | 235.484308 | 243.216406 | 239.726101 | 0.49x | 14245.203 |
| PyRanges1 | 109.722359 | 112.326873 | 111.23273 | 1.06x | 19423.172 |

Table 108: Performance and peak memory usage of the *nearest* operation on 10000000-1p

| Library | Min (s) | Max (s) | Mean (s) | Speedup | Peak memory (MB) |
|---|---|---|---|---|---|
| polars-bio | 7.842314 | 8.84828 | 8.510181 | 21.04x | 2301.0 |
| pb_stream | 7.589706 | 8.153016 | 7.842404 | 22.83x | 1327.531 |
| Bioframe | 174.790383 | 183.458906 | 179.035999 | 1.00x | 10996.234 |
| PyRanges0 | 32.793505 | 32.826686 | 32.809101 | 5.46x | 4882.656 |
| PyRanges1 | 18.866156 | 19.570609 | 19.142653 | 9.35x | 5253.281 |

Table 109: Performance and peak memory usage of the *coverage* operation on 10000000-1p

| Library | Min (s) | Max (s) | Mean (s) | Speedup | Peak memory (MB) |
|---|---|---|---|---|---|
| polars-bio | 1.901833 | 1.957711 | 1.928367 | 12.15x | 956.844 |
| pb_stream | 1.797332 | 1.802527 | 1.800497 | 13.01x | 651.266 |
| Bioframe | 23.269774 | 23.55838 | 23.430125 | 1.00x | 6493.234 |
| PyRanges1 | 26.370249 | 27.172173 | 26.879266 | 0.87x | 10397.531 |

Table 110: Performance and peak memory usage of the *count overlaps* operation on 10000000-1p

| Library | Min (s) | Max (s) | Mean (s) | Speedup | Peak memory (MB) |
|---|---|---|---|---|---|
| polars-bio | 5.025462 | 5.234103 | 5.129963 | 20.79x | 1036.734 |
| pb_stream | 4.956087 | 5.076052 | 5.014242 | 21.27x | 968.719 |
| Bioframe | 105.322287 | 107.758078 | 106.64158 | 1.00x | 12803.828 |
| PyRanges1 | 22.079391 | 23.069931 | 22.618209 | 4.71x | 10039.297 |

#### 3.3.4.2 gcp-linux

Table 111: Performance and peak memory usage of the *overlap* operation on fBrain-vs-exons

| Library | Min (s) | Max (s) | Mean (s) | Speedup | Peak memory (MB) |
|---|---|---|---|---|---|
| polars-bio | 0.072393 | 0.151871 | 0.09916 | 2.80x | 314.234 |
| pb_stream | 0.064092 | 0.067914 | 0.066202 | 4.19x | 288.621 |
| Bioframe | 0.258278 | 0.31288 | 0.277225 | 1.00x | 287.101 |
| PyRanges0 | 0.591745 | 0.599954 | 0.595204 | 0.47x | 307.218 |
| PyRanges1 | 0.683388 | 0.702289 | 0.690362 | 0.40x | 327.863 |

Table 112: Performance and peak memory usage of the *nearest* operation on fBrain-vs-exons

| Library | Min (s) | Max (s) | Mean (s) | Speedup | Peak memory (MB) |
|---|---|---|---|---|---|
| polars-bio | 0.123656 | 1.702108 | 0.659015 | 1.99x | 331.398 |
| pb_stream | 0.111801 | 0.762227 | 0.328874 | 3.98x | 308.738 |
| Bioframe | 0.881782 | 2.161628 | 1.309551 | 1.00x | 297.695 |
| PyRanges0 | 1.728053 | 2.579086 | 2.030527 | 0.64x | 308.93 |
| PyRanges1 | 1.953048 | 2.161655 | 2.049615 | 0.64x | 337.352 |

Table 113: Performance and peak memory usage of the *coverage* operation on fBrain-vs-exons

| Library | Min (s) | Max (s) | Mean (s) | Speedup | Peak memory (MB) |
|---|---|---|---|---|---|
| polars-bio | 0.064626 | 0.146983 | 0.093048 | 8.11x | 299.094 |
| pb_stream | 0.065193 | 0.072839 | 0.068651 | 10.99x | 280.387 |
| Bioframe | 0.704155 | 0.791049 | 0.754463 | 1.00x | 328.184 |
| PyRanges1 | 1.41166 | 1.432833 | 1.42261 | 0.53x | 352.582 |

Table 114: Performance and peak memory usage of the *count overlaps* operation on fBrain-vs-exons

| Library | Min (s) | Max (s) | Mean (s) | Speedup | Peak memory (MB) |
|---|---|---|---|---|---|
| polars-bio | 0.063012 | 0.207803 | 0.113156 | 4.03x | 309.176 |
| pb_stream | 0.062735 | 0.071474 | 0.065886 | 6.92x | 286.336 |
| Bioframe | 0.436935 | 0.491839 | 0.455688 | 1.00x | 303.07 |
| PyRanges1 | 0.785823 | 0.786847 | 0.786487 | 0.58x | 316.227 |

Table 115: Performance and peak memory usage of the *overlap* operation on ex-rna-vs-ex-anno

| Library | Min (s) | Max (s) | Mean (s) | Speedup | Peak memory (MB) |
|---|---|---|---|---|---|
| polars-bio | 44.539766 | 45.543038 | 45.196903 | 12.55x | 14575.14 |
| pb_stream | 34.007093 | 35.972075 | 35.309756 | 16.06x | 480.207 |
| Bioframe | 566.167037 | 567.617695 | 567.13069 | 1.00x | 43295.378 |
| PyRanges0 | 417.291061 | 421.875539 | 419.571591 | 1.35x | 22915.917 |
| PyRanges1 | 538.365637 | 548.624613 | 543.918168 | 1.04x | 43408.699 |

Table 116: Performance and peak memory usage of the *nearest* operation on ex-rna-vs-ex-anno

| Library | Min (s) | Max (s) | Mean (s) | Speedup | Peak memory (MB) |
|---|---|---|---|---|---|
| polars-bio | 6.565142 | 7.696104 | 6.973448 | 12.21x | 1070.016 |
| pb_stream | 5.840416 | 6.828222 | 6.203 | 13.73x | 527.008 |
| Bioframe | 84.30831 | 86.512823 | 85.150539 | 1.00x | 2418.629 |
| PyRanges0 | 20.679566 | 21.424632 | 20.949203 | 4.06x | 2239.047 |
| PyRanges1 | 25.352803 | 27.604137 | 26.544063 | 3.21x | 2534.629 |

Table 117: Performance and peak memory usage of the *coverage* operation on ex-rna-vs-ex-anno

| Library | Min (s) | Max (s) | Mean (s) | Speedup | Peak memory (MB) |
|---|---|---|---|---|---|
| polars-bio | 3.532309 | 3.660049 | 3.579428 | 12.53x | 738.887 |
| pb_stream | 3.167344 | 3.169622 | 3.168694 | 14.15x | 416.164 |
| Bioframe | 41.150587 | 51.89725 | 44.839673 | 1.00x | 14297.098 |
| PyRanges1 | 40.065526 | 41.350493 | 40.892187 | 1.10x | 3096.812 |

Table 118: Performance and peak memory usage of the *count overlaps* operation on ex-rna-vs-ex-anno

| Library | Min (s) | Max (s) | Mean (s) | Speedup | Peak memory (MB) |
|---|---|---|---|---|---|
| polars-bio | 3.827717 | 3.83517 | 3.830823 | 25.47x | 737.059 |
| pb_stream | 3.346898 | 3.388796 | 3.372987 | 28.93x | 428.422 |
| Bioframe | 97.272988 | 97.790775 | 97.572564 | 1.00x | 25981.051 |
| PyRanges1 | 30.021737 | 30.181438 | 30.124339 | 3.24x | 3102.84 |

Table 119: Performance and peak memory usage of the *overlap* operation on 100-1p

| Library | Min (s) | Max (s) | Mean (s) | Speedup | Peak memory (MB) |
|---|---|---|---|---|---|
| polars-bio | 0.012022 | 0.433918 | 0.153077 | 0.55x | 262.656 |
| pb_stream | 0.007427 | 0.153294 | 0.056144 | 1.49x | 259.039 |
| Bioframe | 0.039406 | 0.172494 | 0.08386 | 1.00x | 229.824 |
| PyRanges0 | 0.059086 | 0.075573 | 0.06466 | 1.30x | 231.199 |
| PyRanges1 | 0.069077 | 0.088036 | 0.075488 | 1.11x | 230.684 |

Table 120: Performance and peak memory usage of the *nearest* operation on 100-1p

| Library | Min (s) | Max (s) | Mean (s) | Speedup | Peak memory (MB) |
|---|---|---|---|---|---|
| polars-bio | 0.012814 | 0.408332 | 0.145315 | 1.24x | 263.16 |
| pb_stream | 0.007605 | 0.007975 | 0.00779 | 23.20x | 260.242 |
| Bioframe | 0.044222 | 0.45263 | 0.180742 | 1.00x | 230.684 |
| PyRanges0 | 0.066032 | 0.074886 | 0.068992 | 2.62x | 231.195 |
| PyRanges1 | 0.07111 | 0.075383 | 0.072851 | 2.48x | 230.68 |

Table 121: Performance and peak memory usage of the *coverage* operation on 100-1p

| Library | Min (s) | Max (s) | Mean (s) | Speedup | Peak memory (MB) |
|---|---|---|---|---|---|
| polars-bio | 0.008954 | 0.106466 | 0.041616 | 2.35x | 258.184 |
| pb_stream | 0.006726 | 0.007524 | 0.007124 | 13.72x | 255.258 |
| Bioframe | 0.07866 | 0.135591 | 0.097742 | 1.00x | 230.68 |
| PyRanges1 | 0.120404 | 0.12302 | 0.121487 | 0.80x | 231.023 |

Table 122: Performance and peak memory usage of the *count overlaps* operation on 100-1p

| Library | Min (s) | Max (s) | Mean (s) | Speedup | Peak memory (MB) |
|---|---|---|---|---|---|
| polars-bio | 0.008886 | 0.095835 | 0.038506 | 1.53x | 262.312 |
| pb_stream | 0.006988 | 0.008915 | 0.007637 | 7.71x | 259.555 |
| Bioframe | 0.043766 | 0.08625 | 0.058895 | 1.00x | 230.852 |
| PyRanges1 | 0.060574 | 0.060725 | 0.06064 | 0.97x | 231.195 |

Table 123: Performance and peak memory usage of the *overlap* operation on 10000000-1p

| Library | Min (s) | Max (s) | Mean (s) | Speedup | Peak memory (MB) |
|---|---|---|---|---|---|
| polars-bio | 35.783773 | 37.155414 | 36.274578 | 4.73x | 6926.227 |
| pb_stream | 28.364026 | 33.834447 | 32.005189 | 5.37x | 1172.484 |
| Bioframe | 170.321558 | 173.826371 | 171.750864 | 1.00x | 17544.5 |
| PyRanges0 | 374.384106 | 377.106338 | 375.972726 | 0.46x | 12951.133 |
| PyRanges1 | 174.205234 | 176.465726 | 174.996859 | 0.98x | 23198.973 |

Table 124: Performance and peak memory usage of the *nearest* operation on 10000000-1p

| Library | Min (s) | Max (s) | Mean (s) | Speedup | Peak memory (MB) |
|---|---|---|---|---|---|
| polars-bio | 16.577973 | 16.599008 | 16.591114 | 5.88x | 2208.477 |
| pb_stream | 14.957252 | 15.214483 | 15.115055 | 6.45x | 1202.555 |
| Bioframe | 96.638005 | 98.21701 | 97.559755 | 1.00x | 7832.391 |
| PyRanges0 | 54.678927 | 55.140916 | 54.905002 | 1.78x | 3125.051 |
| PyRanges1 | 31.874441 | 33.028755 | 32.303297 | 3.02x | 4447.332 |

Table 125: Performance and peak memory usage of the *coverage* operation on 10000000-1p

| Library | Min (s) | Max (s) | Mean (s) | Speedup | Peak memory (MB) |
|---|---|---|---|---|---|
| polars-bio | 4.184608 | 4.403694 | 4.281924 | 6.97x | 768.75 |
| pb_stream | 3.792566 | 3.917329 | 3.838723 | 7.77x | 591.812 |
| Bioframe | 29.609636 | 29.972788 | 29.838014 | 1.00x | 4040.508 |
| PyRanges1 | 41.671912 | 42.238756 | 41.949904 | 0.71x | 8503.844 |

Table 126: Performance and peak memory usage of the *count overlaps* operation on 10000000-1p

| Library | Min (s) | Max (s) | Mean (s) | Speedup | Peak memory (MB) |
|---|---|---|---|---|---|
| polars-bio | 11.003474 | 11.126526 | 11.052697 | 6.35x | 1012.105 |
| pb_stream | 9.939793 | 10.434084 | 10.264927 | 6.83x | 696.078 |
| Bioframe | 70.00646 | 70.300308 | 70.14716 | 1.00x | 8315.176 |
| PyRanges1 | 33.521685 | 33.726979 | 33.593637 | 2.09x | 8495.672 |

## 3.4 Memory profiles evaluation

Figures in this section present memory–profile evaluations for four representative cases: a small real–world pair (`fBrain` → `exons`, also labeled as “1→2” dataset), a large real–world pair (`ex-rna` → `ex-anno`, also labeled as “1→2” dataset), a small synthetic dataset (100–1p), and a large synthetic dataset (10000000–1p).

### 3.4.1 Operation: overlap for dataset: fBrain-vs-exons on platform: apple-m3-max

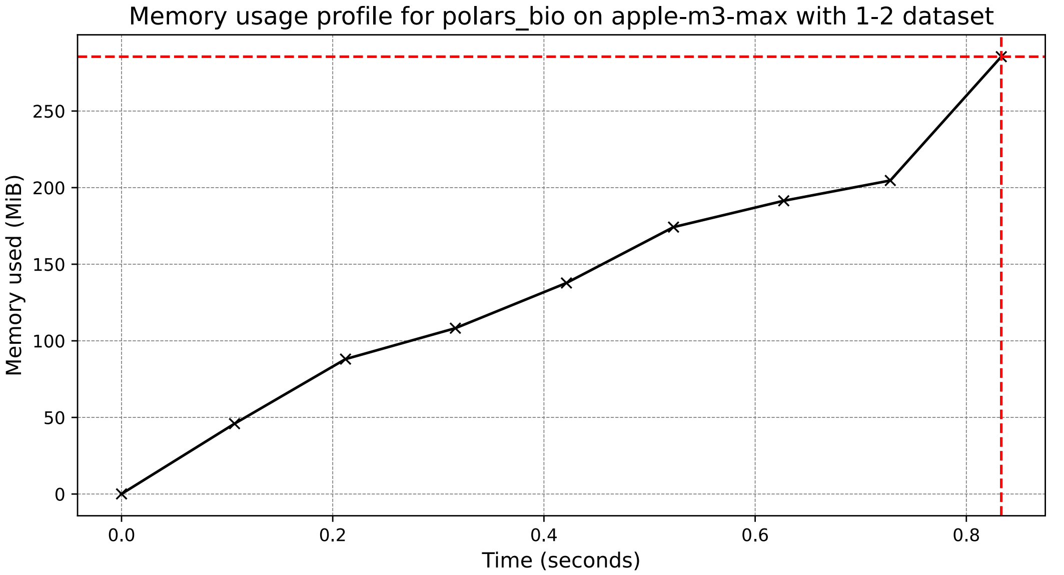


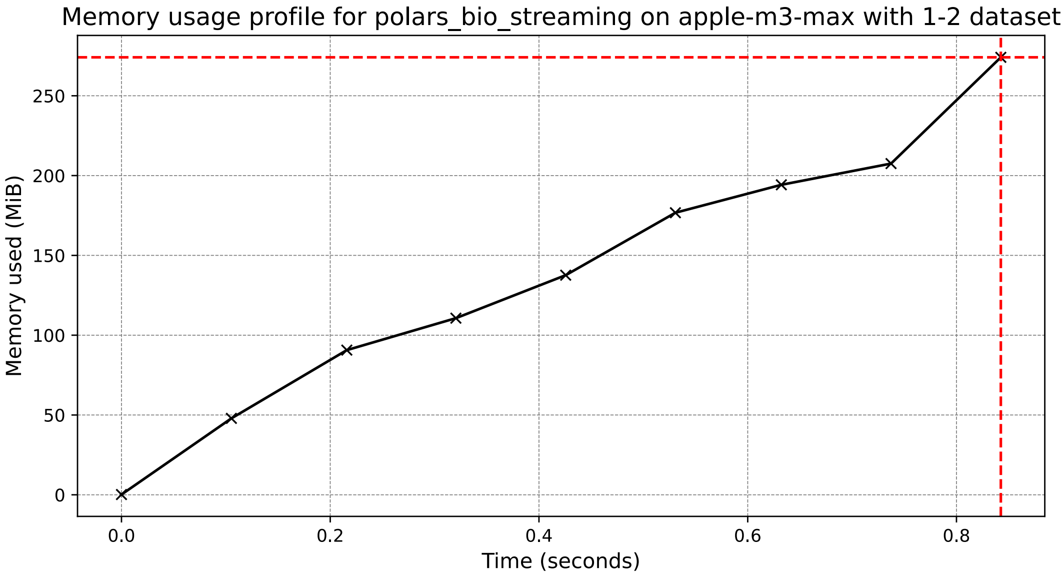

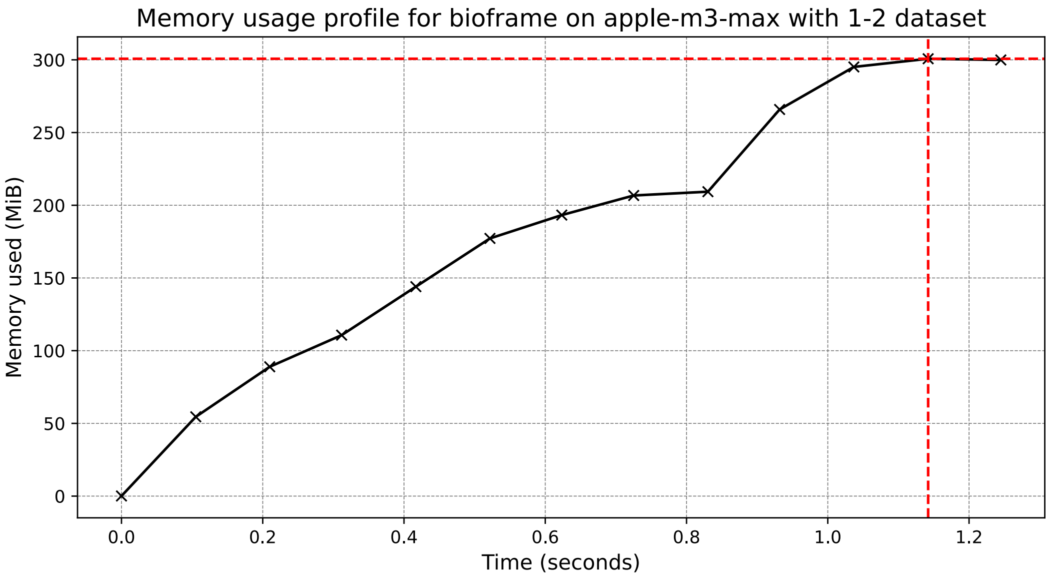
Memory usage profile for bioframe on apple-m3-max with 1-2 dataset
Memory used (MiB)
Time (seconds)
0
50
100
150
200
250
300
0.0
0.2
0.4
0.6
0.8
1.0
1.2

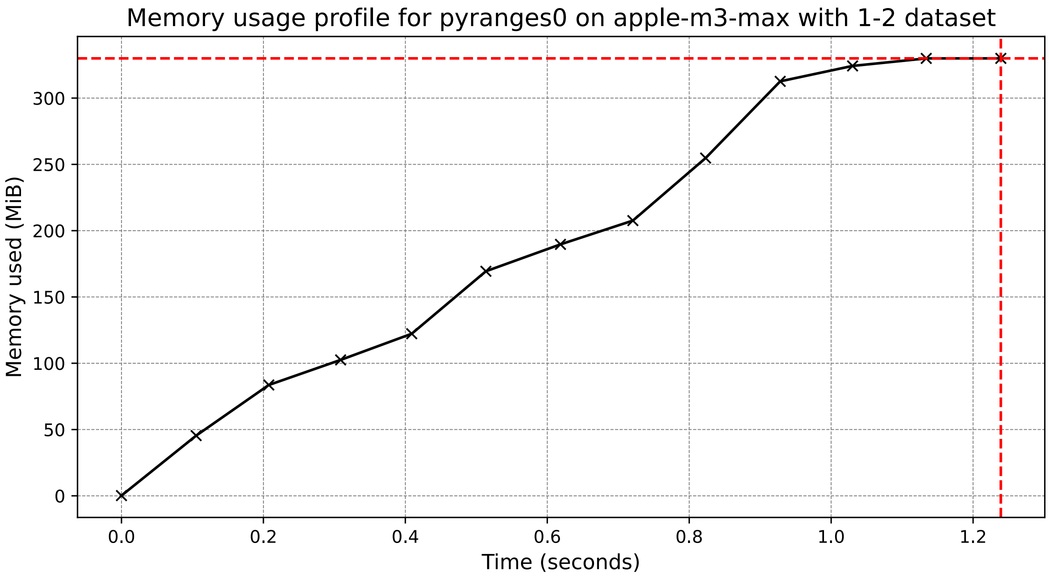
Memory usage profile for pyranges0 on apple-m3-max with 1-2 dataset
Memory used (MiB)
Time (seconds)
0
50
100
150
200
250
300
0.0
0.2
0.4
0.6
0.8
1.0
1.2

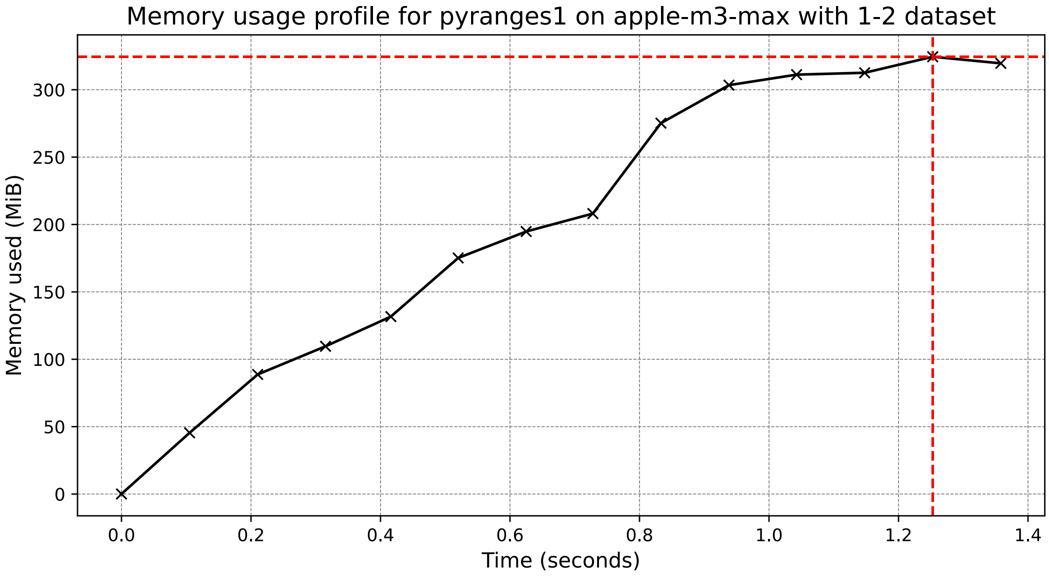


### 3.4.2 Operation: nearest for dataset: fBrain-vs-exons on platform: apple-m3-max

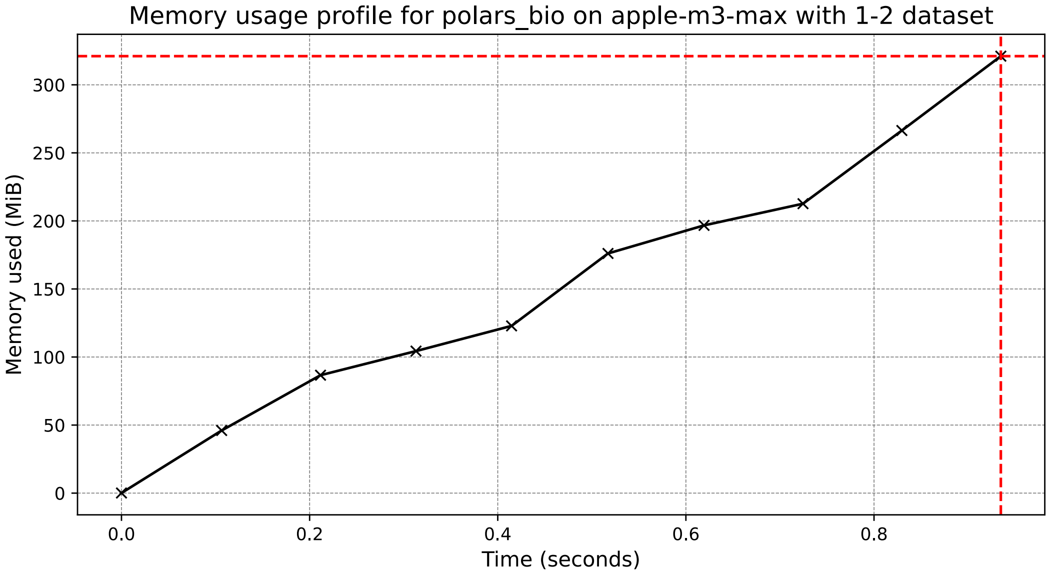

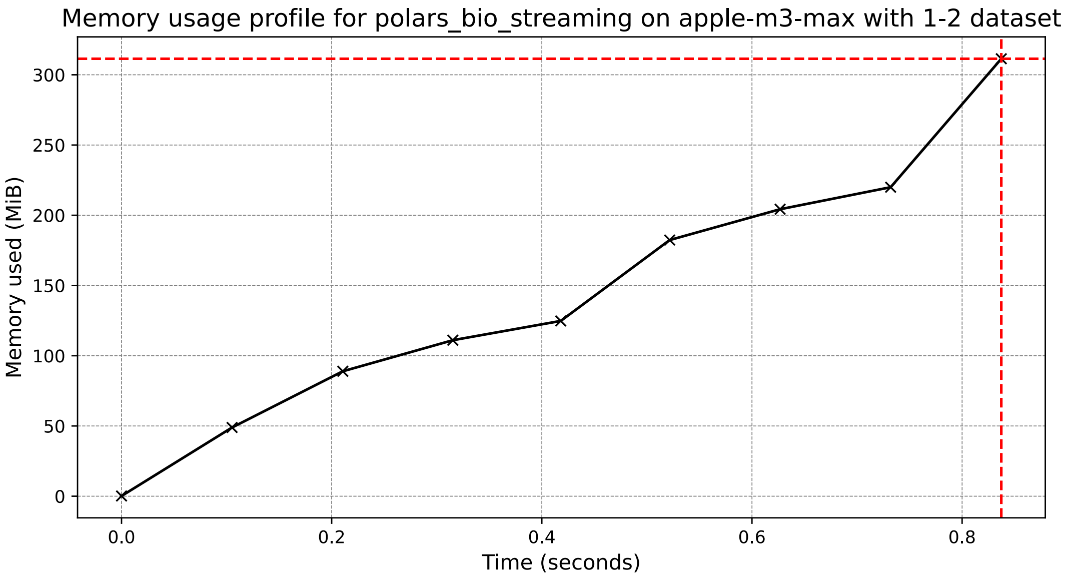
Memory usage profile for polars_bio_streaming on apple-m3-max with 1-2 dataset
Memory used (MiB)
Time (seconds)
0
50
100
150
200
250
300
0.0
0.2
0.4
0.6
0.8

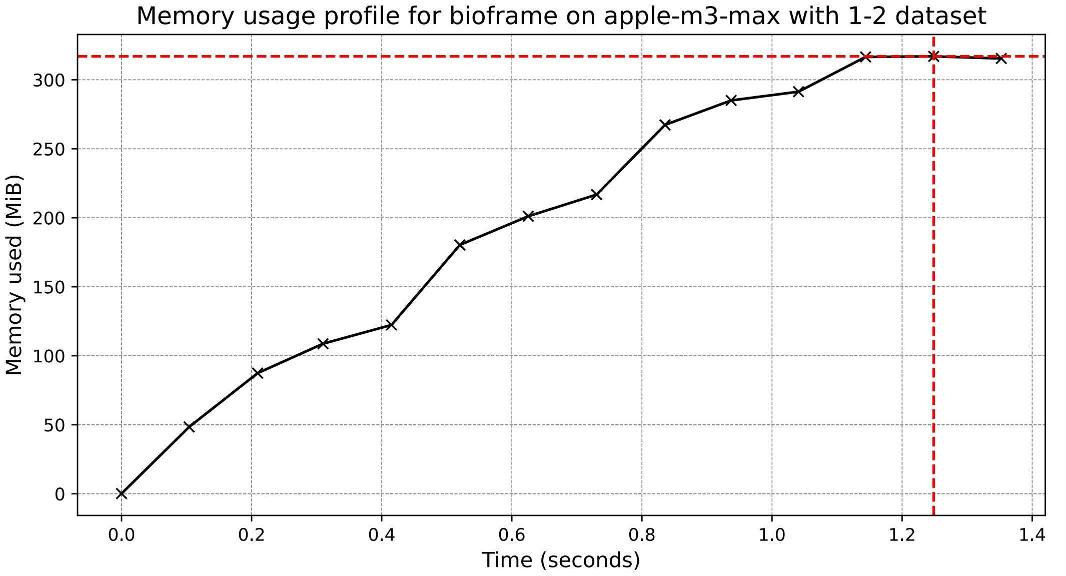
Memory usage profile for bioframe on apple-m3-max with 1-2 dataset
Memory used (MiB)
Time (seconds)
0
50
100
150
200
250
300
0.0
0.2
0.4
0.6
0.8
1.0
1.2
1.4

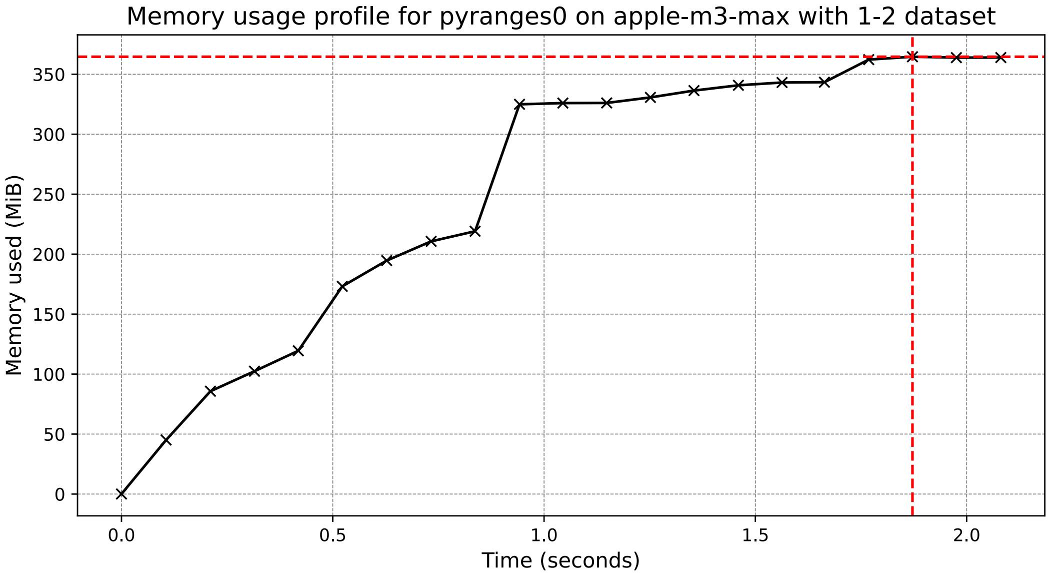
Memory usage profile for pyranges0 on apple-m3-max with 1-2 dataset
Memory used (MiB)
Time (seconds)
0
50
100
150
200
250
300
350
0.0
0.5
1.0
1.5
2.0

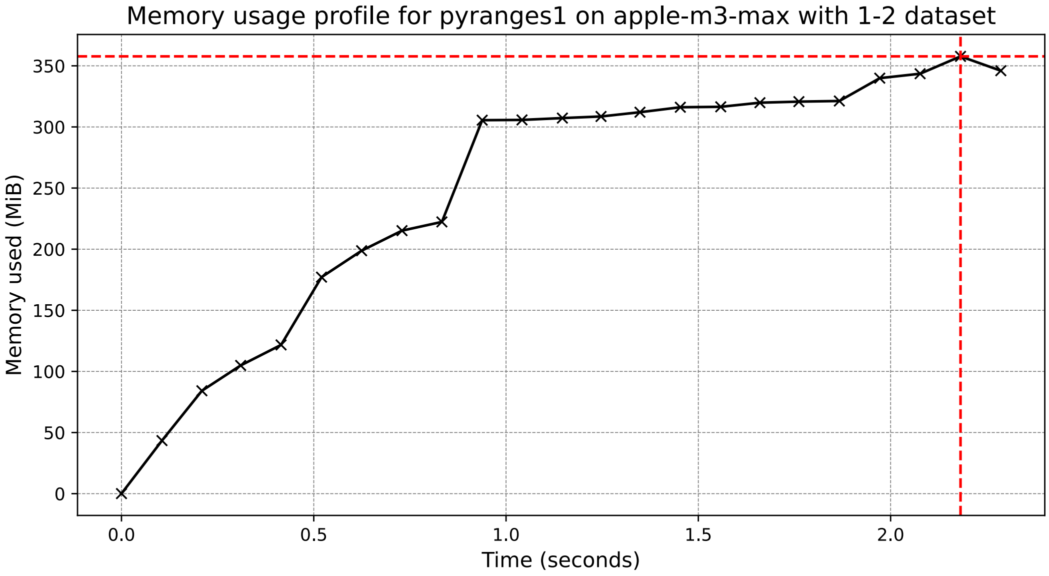
Memory usage profile for pyranges1 on apple-m3-max with 1-2 dataset
Memory used (MiB)
Time (seconds)
0
50
100
150
200
250
300
350
0.0
0.5
1.0
1.5
2.0

### 3.4.3 Operation: coverage for dataset: fBrain-vs-exons on platform: apple-m3-max

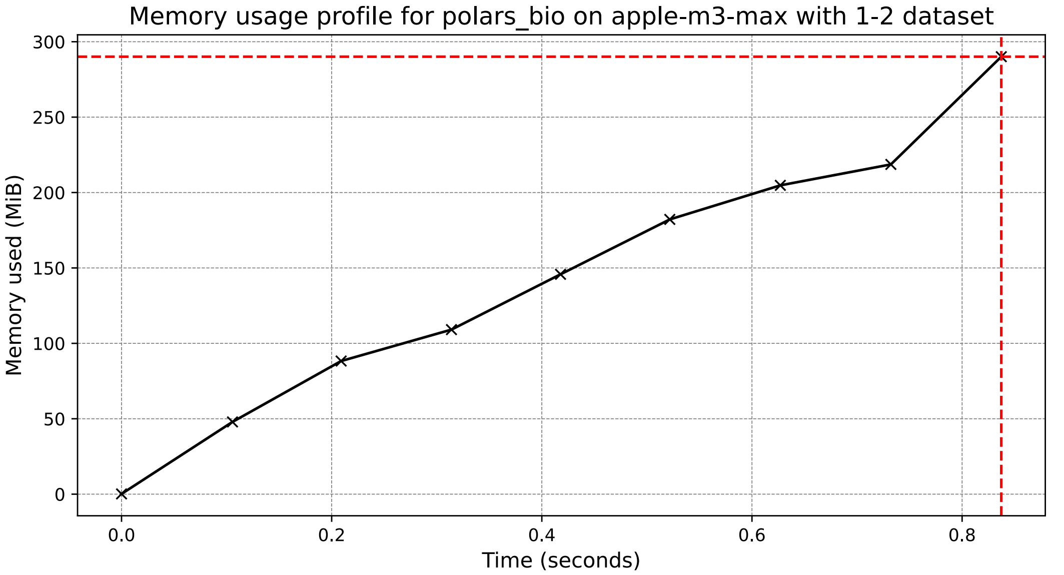


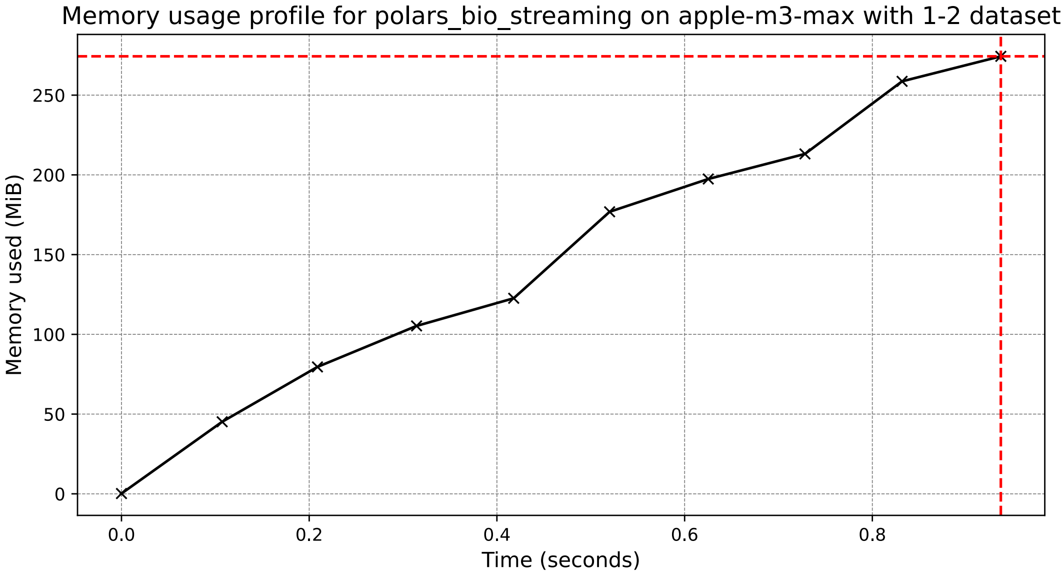

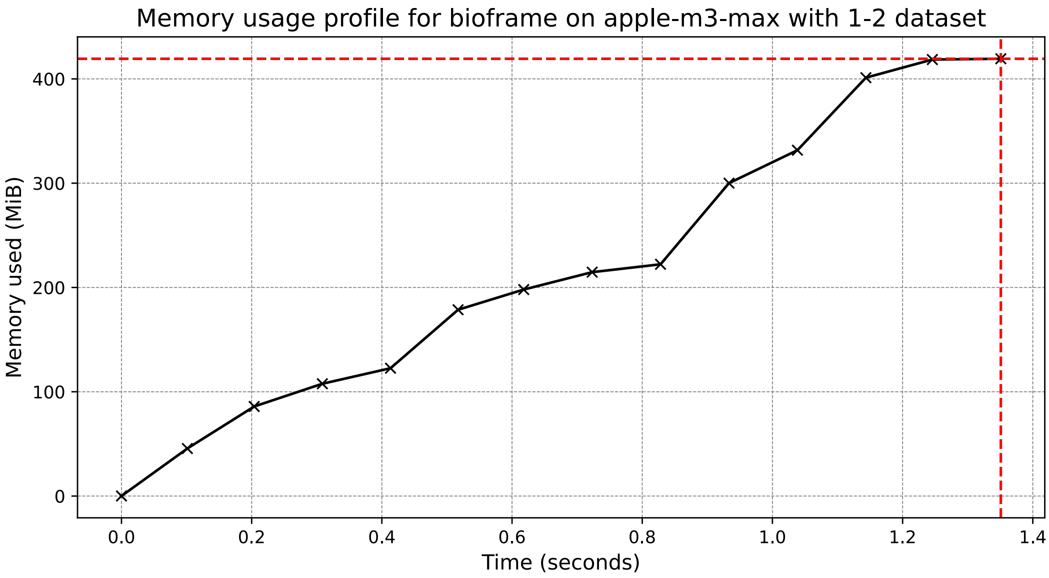
Memory usage profile for bioframe on apple-m3-max with 1-2 dataset
Memory used (MiB)
Time (seconds)
0
100
200
300
400
0.0
0.2
0.4
0.6
0.8
1.0
1.2
1.4

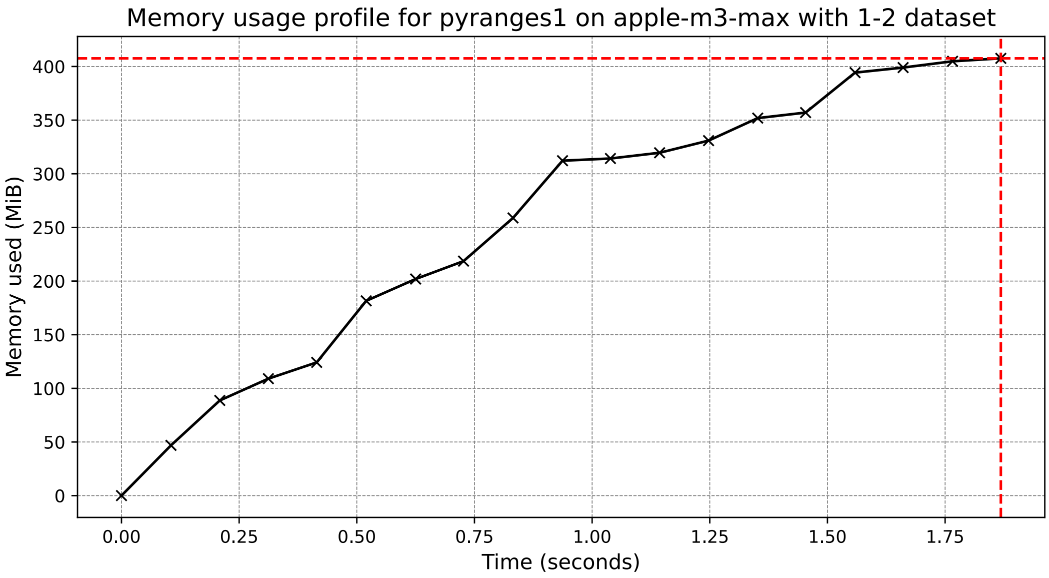
Memory usage profile for pyranges1 on apple-m3-max with 1-2 dataset
Memory used (MiB)
Time (seconds)
0
50
100
150
200
250
300
350
400
0.00
0.25
0.50
0.75
1.00
1.25
1.50
1.75

### 3.4.4 Operation: count-overlaps for dataset: fBrain-vs-exons on platform: apple-m3-max

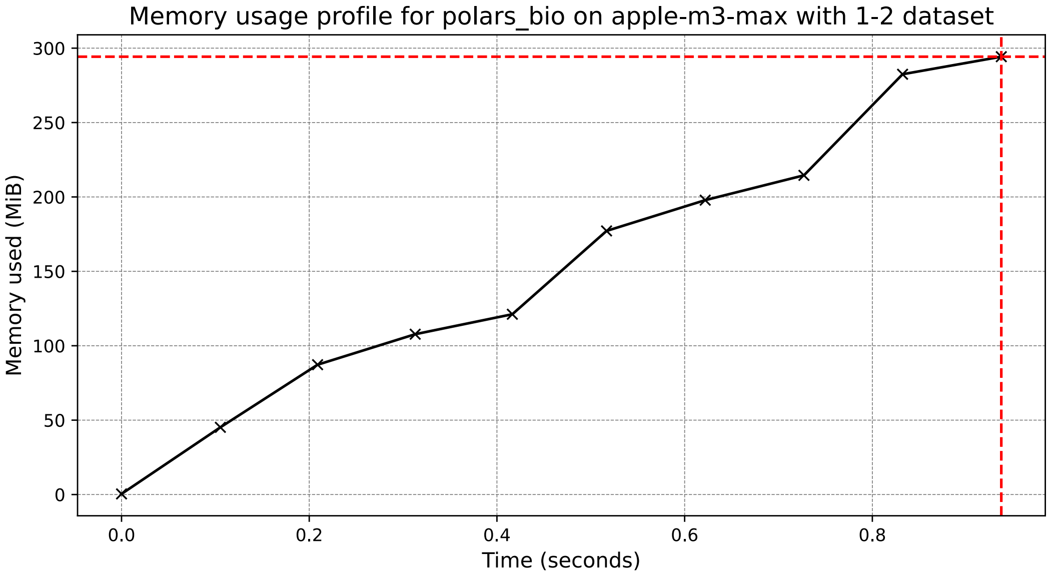


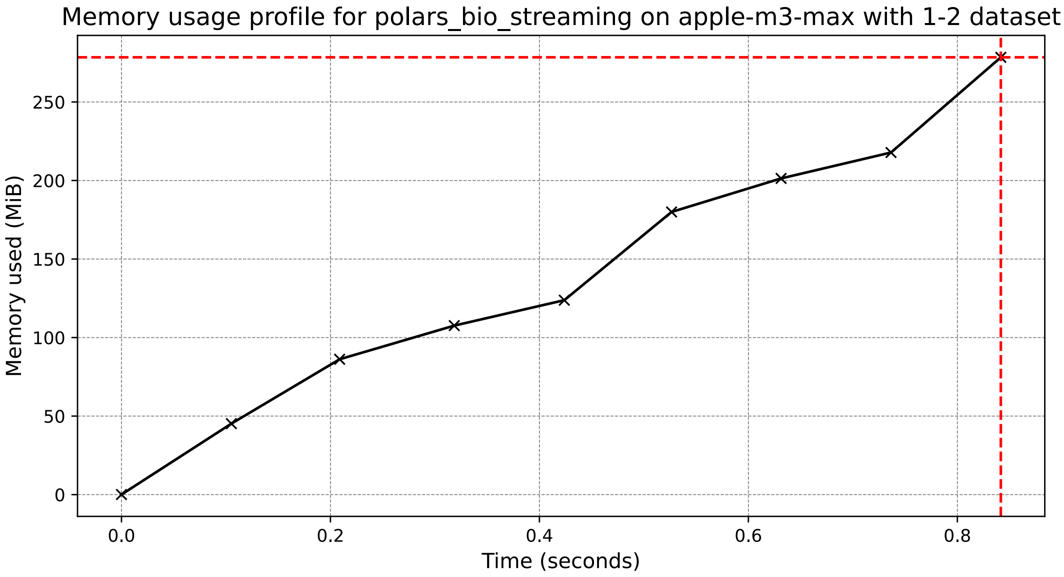

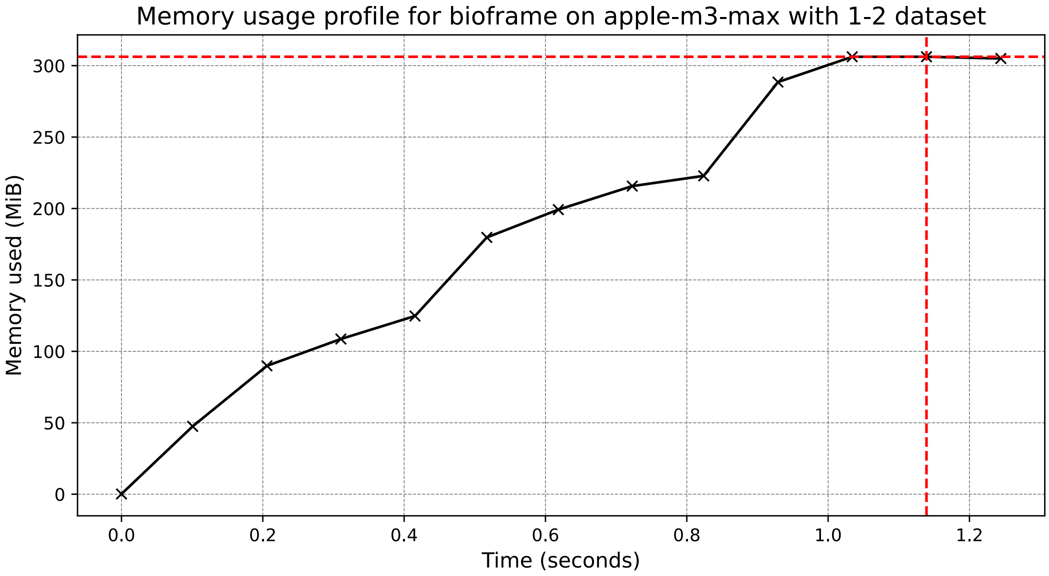
Memory usage profile for bioframe on apple-m3-max with 1-2 dataset
Memory used (MiB)
Time (seconds)
0
50
100
150
200
250
300
0.0
0.2
0.4
0.6
0.8
1.0
1.2

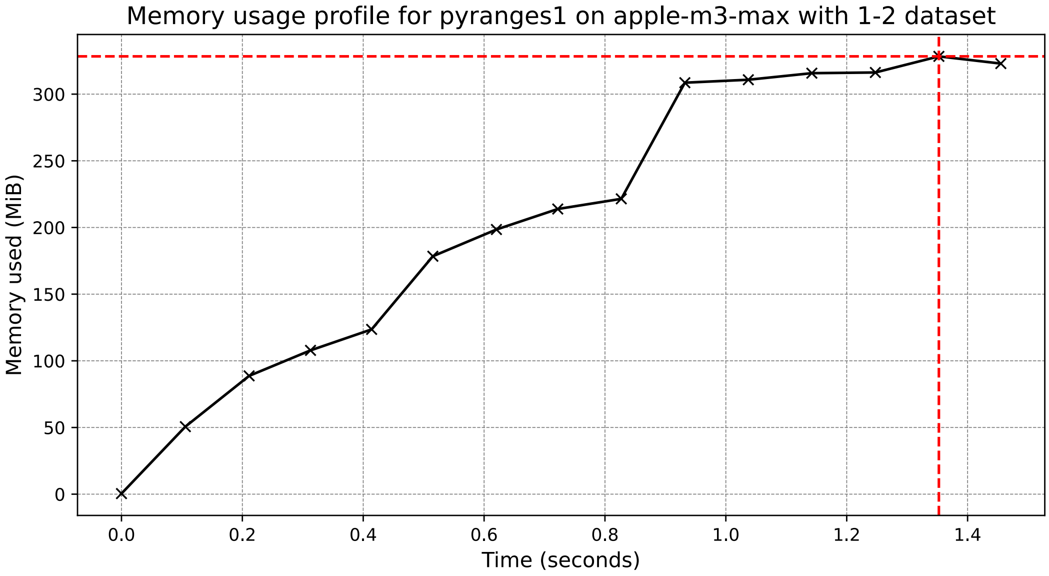
Memory usage profile for pyranges1 on apple-m3-max with 1-2 dataset
Memory used (MiB)
Time (seconds)
0
50
100
150
200
250
300
0.0
0.2
0.4
0.6
0.8
1.0
1.2
1.4

### 3.4.5 Operation: overlap for dataset: ex-rna-vs-ex-anno on platform: apple-m3-max

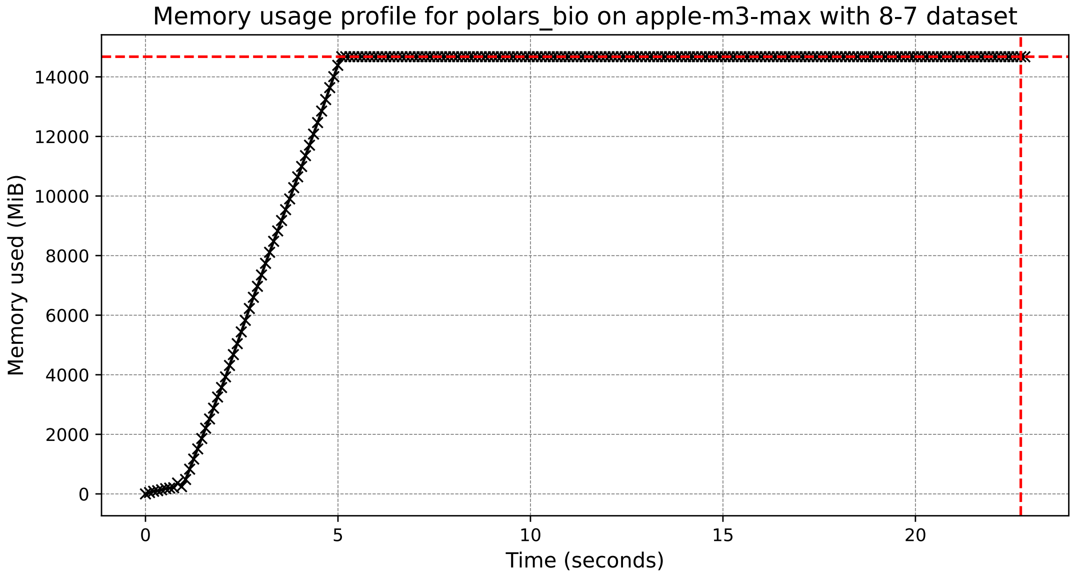


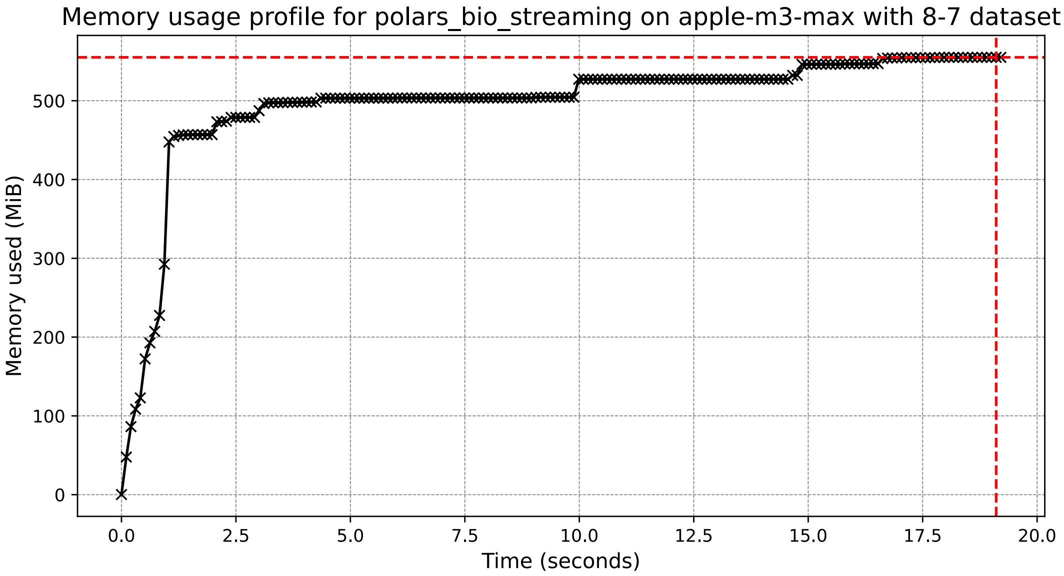

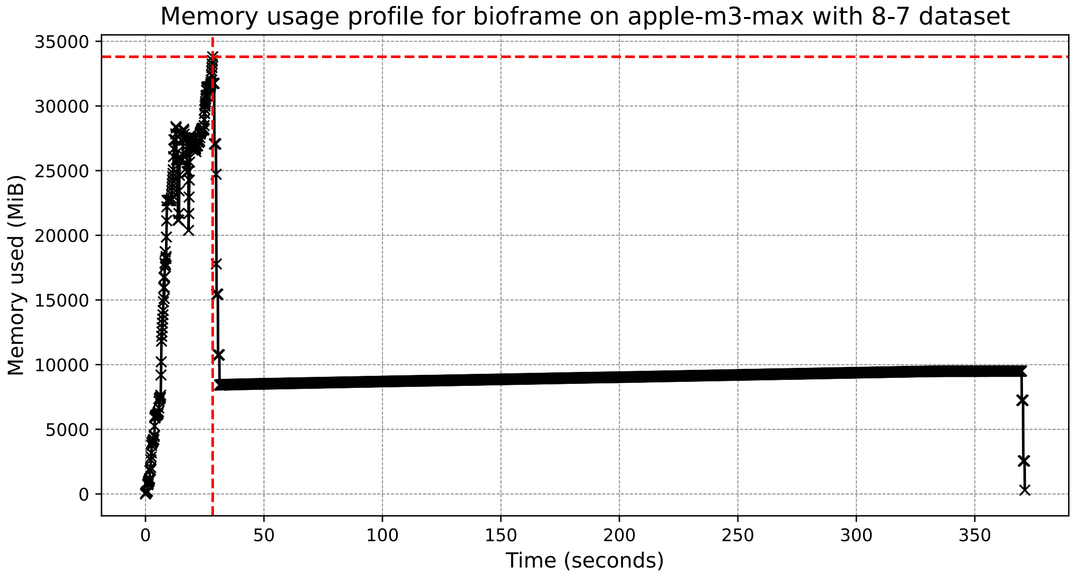
Memory usage profile for bioframe on apple-m3-max with 8-7 dataset
Memory used (MiB)
Time (seconds)
0
5000
10000
15000
20000
25000
30000
35000
0
50
100
150
200
250
300
350

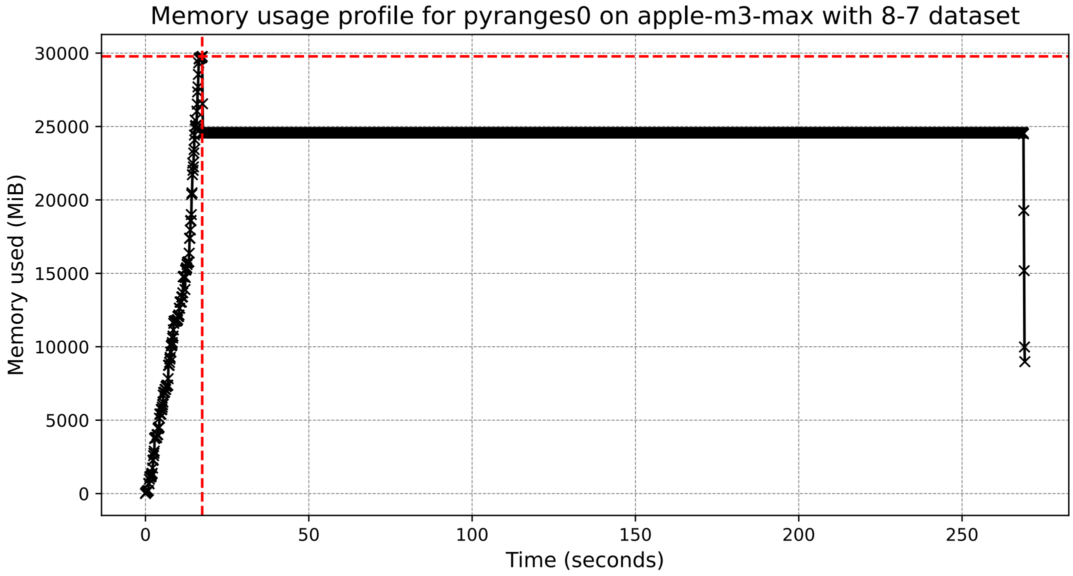
Memory usage profile for pyranges0 on apple-m3-max with 8-7 dataset
Memory used (MiB)
Time (seconds)
0
5000
10000
15000
20000
25000
30000
0
50
100
150
200
250

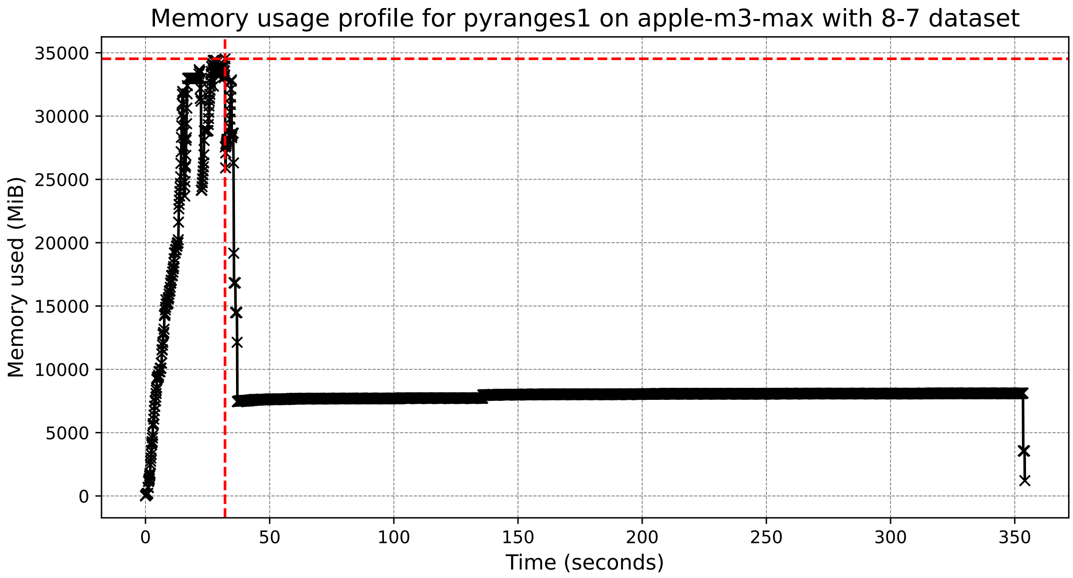


### 3.4.6 Operation: nearest for dataset: ex-rna-vs-ex-anno on platform: apple-m3-max

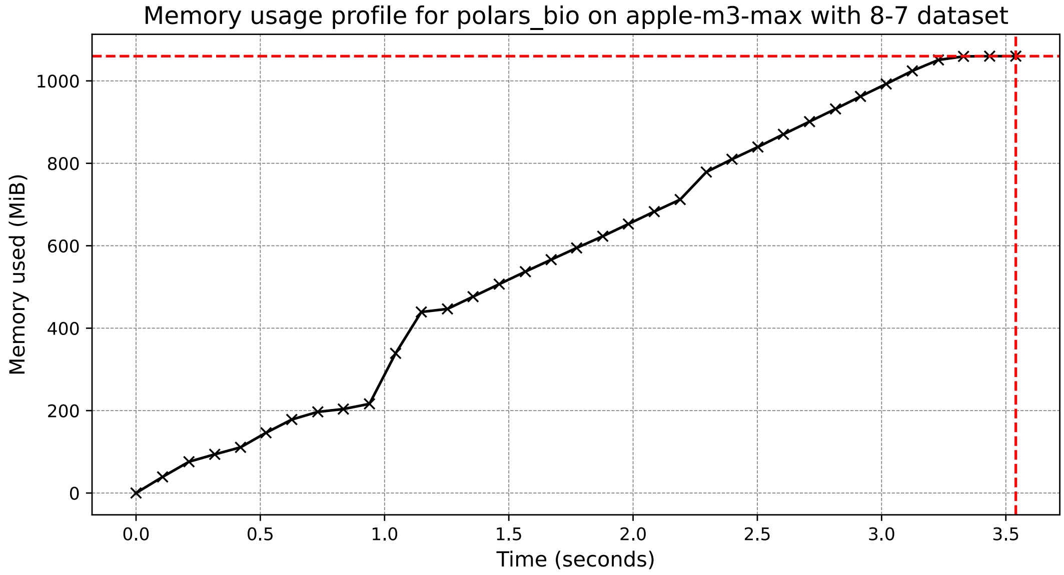

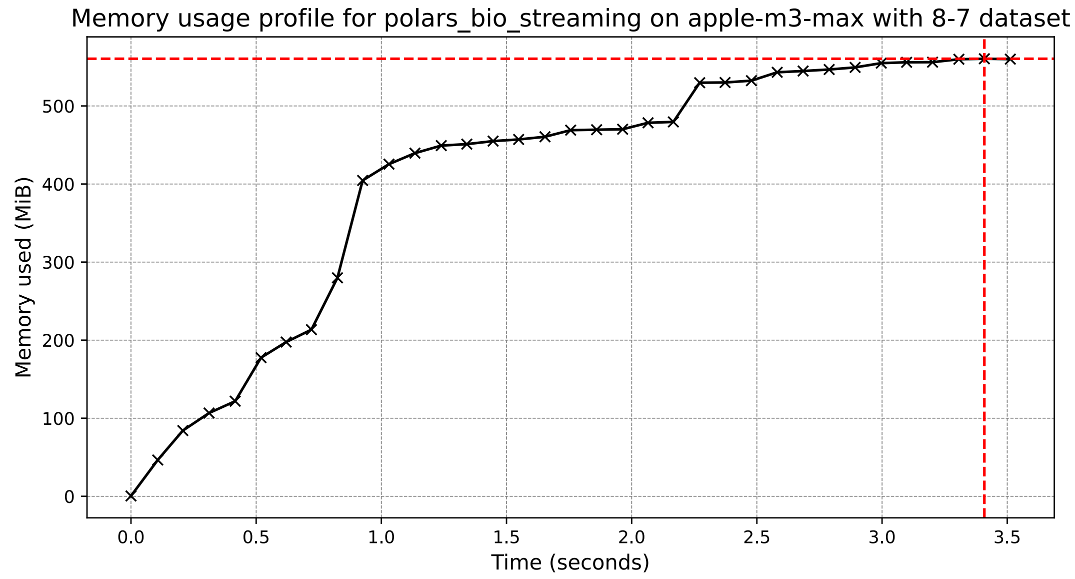
Memory usage profile for polars_bio_streaming on apple-m3-max with 8-7 dataset
Memory used (MiB)
0
100
200
300
400
500
0.0
0.5
1.0
1.5
2.0
2.5
3.0
3.5
Time (seconds)

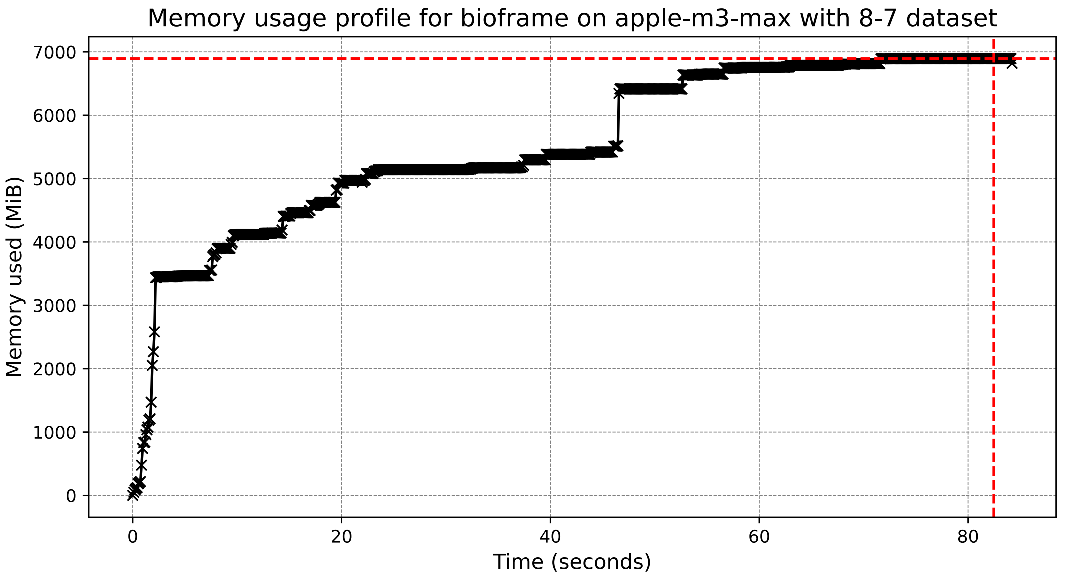
Memory usage profile for bioframe on apple-m3-max with 8-7 dataset
Memory used (MiB)
0
1000
2000
3000
4000
5000
6000
7000
0
20
40
60
80
Time (seconds)

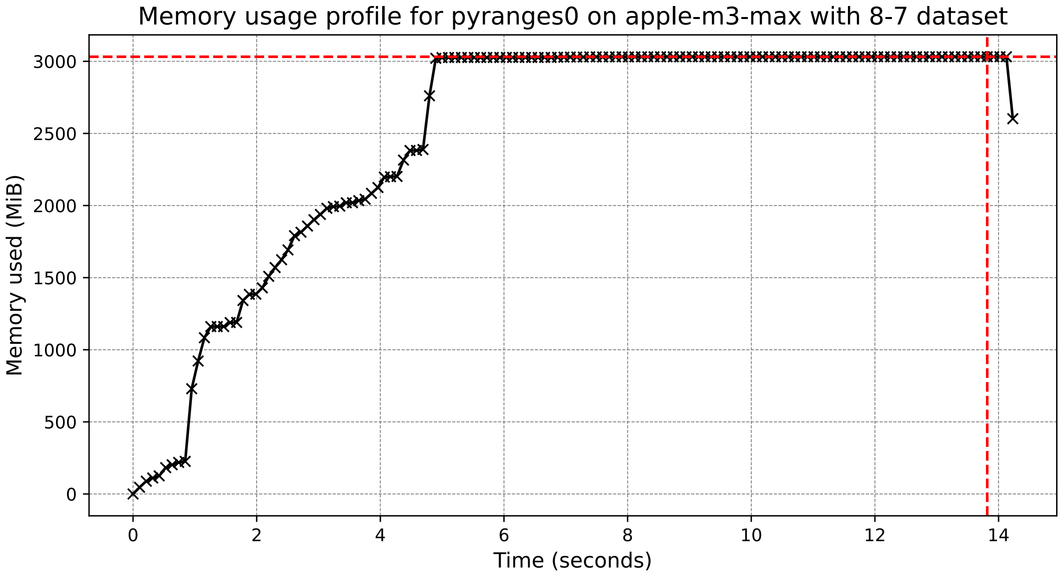

Memory usage profile for pyranges0 on apple-m3-max with 8-7 dataset
Memory used (MiB)
Time (seconds)


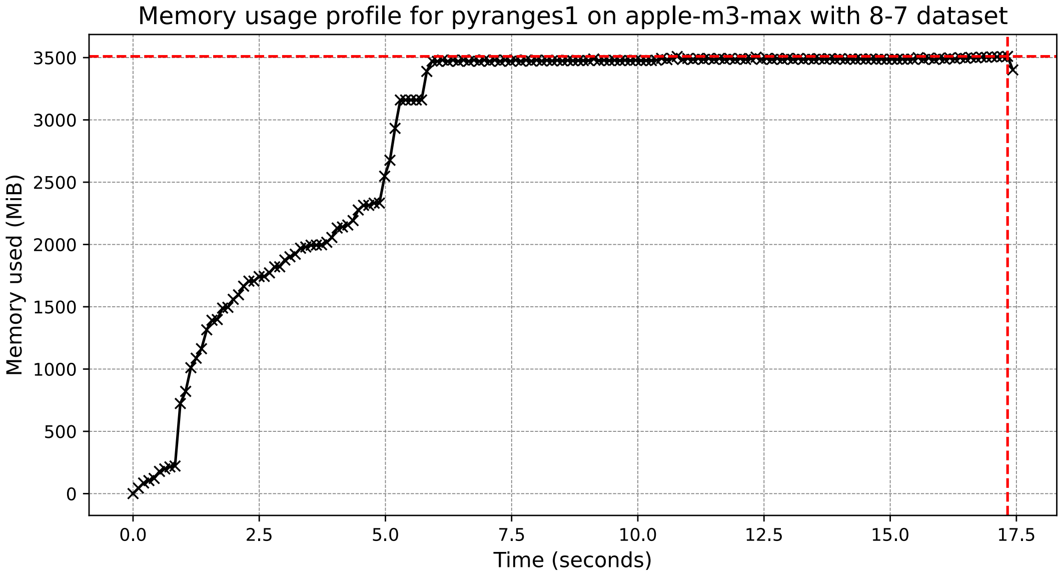

Memory usage profile for pyranges1 on apple-m3-max with 8-7 dataset
Memory used (MiB)
Time (seconds)

### 3.4.7 Operation: coverage for dataset: ex-rna-vs-ex-anno on platform: apple-m3-max

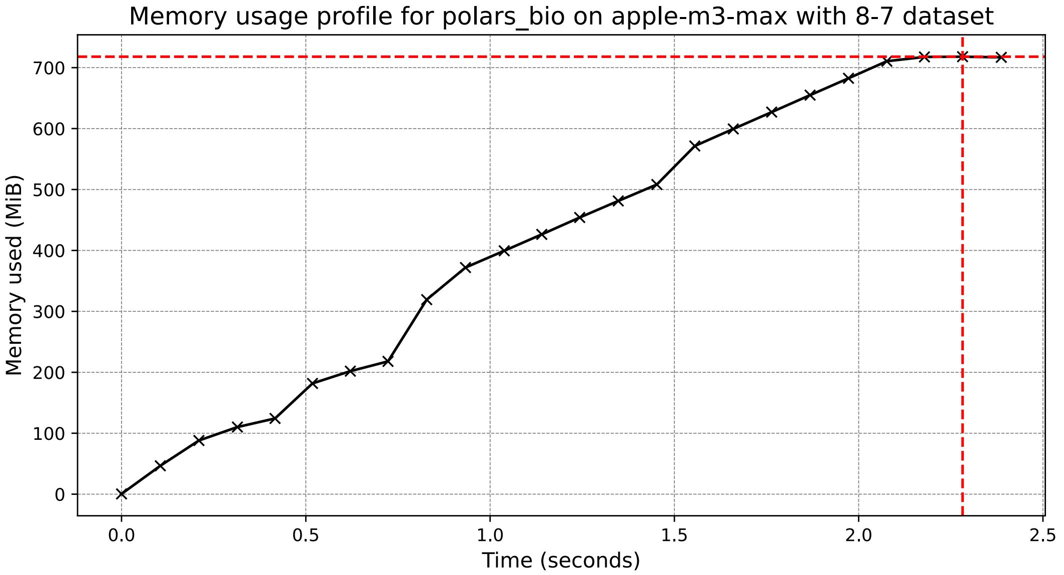


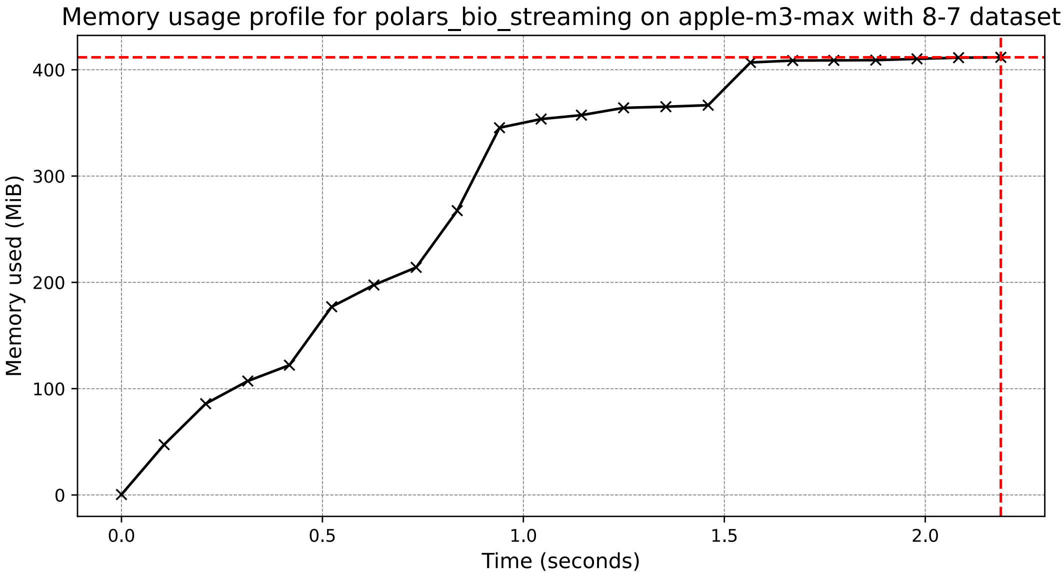

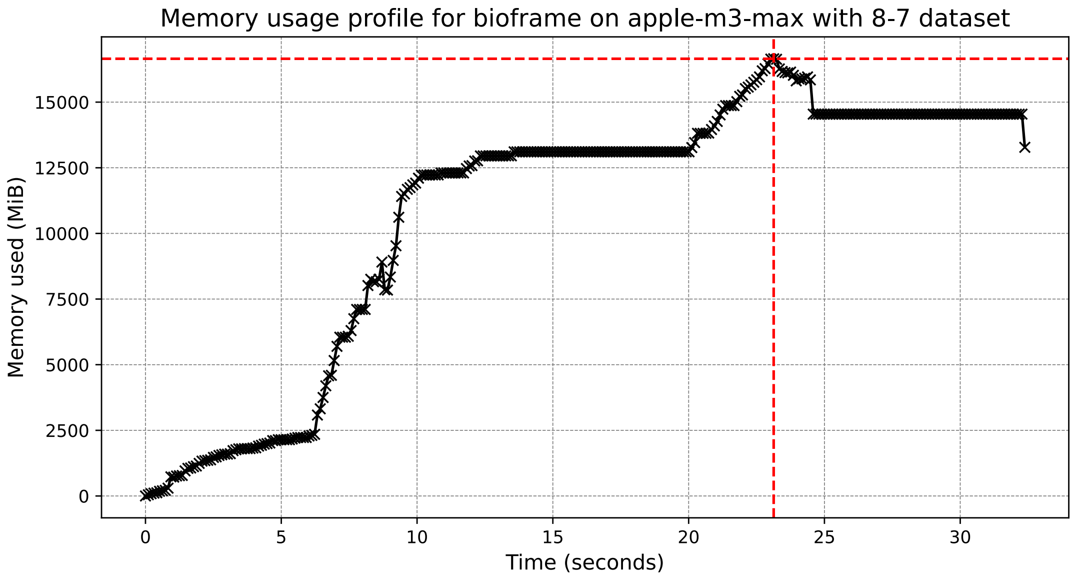

Memory usage profile for bioframe on apple-m3-max with 8-7 dataset
Memory used (MiB)
Time (seconds)


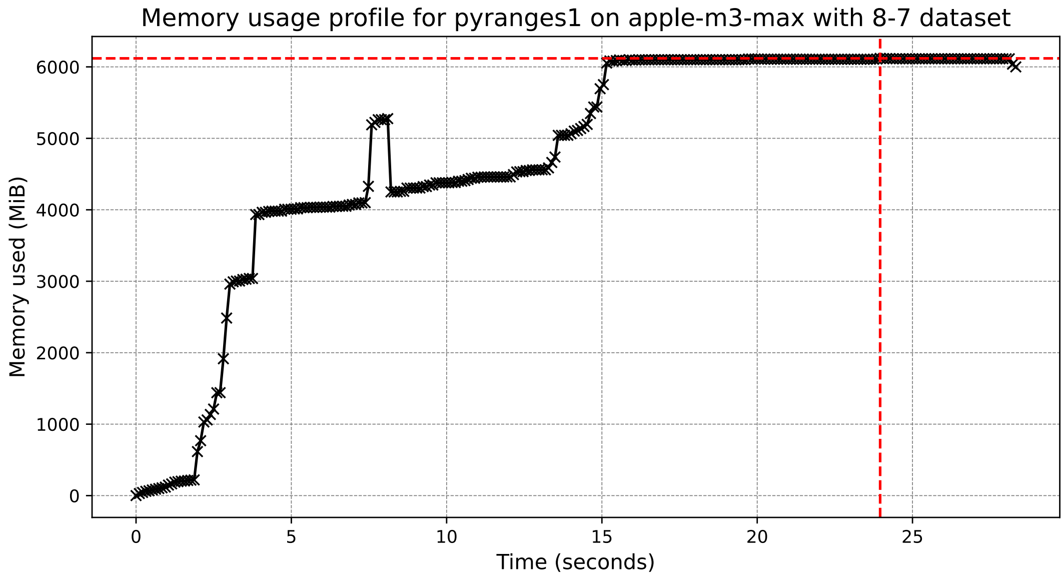

Memory usage profile for pyranges1 on apple-m3-max with 8-7 dataset
Memory used (MiB)
Time (seconds)

#### 3.4.8 Operation: count-overlaps for dataset: ex-rna-vs-ex-anno on platform: apple-m3-max

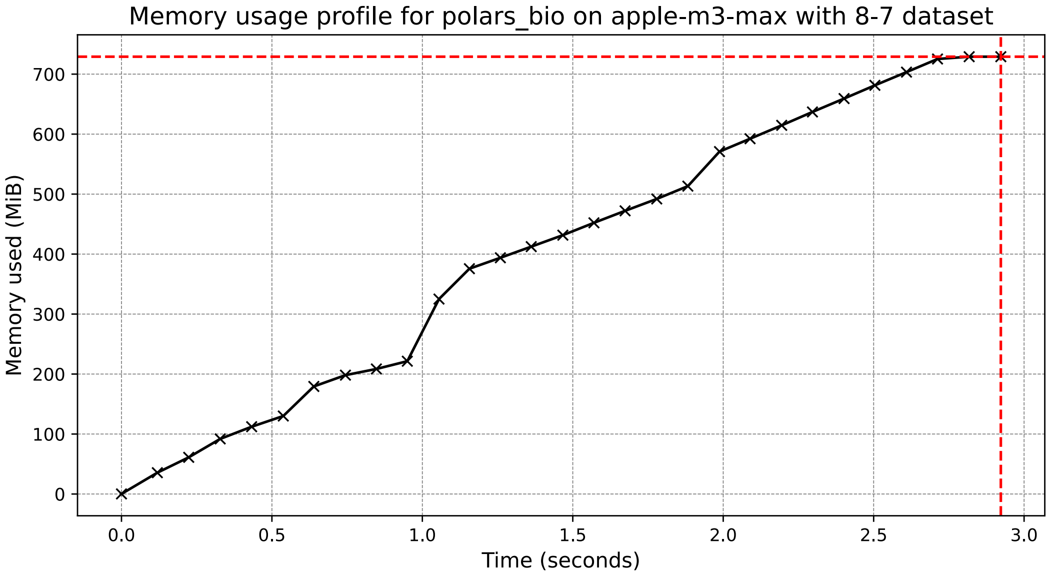


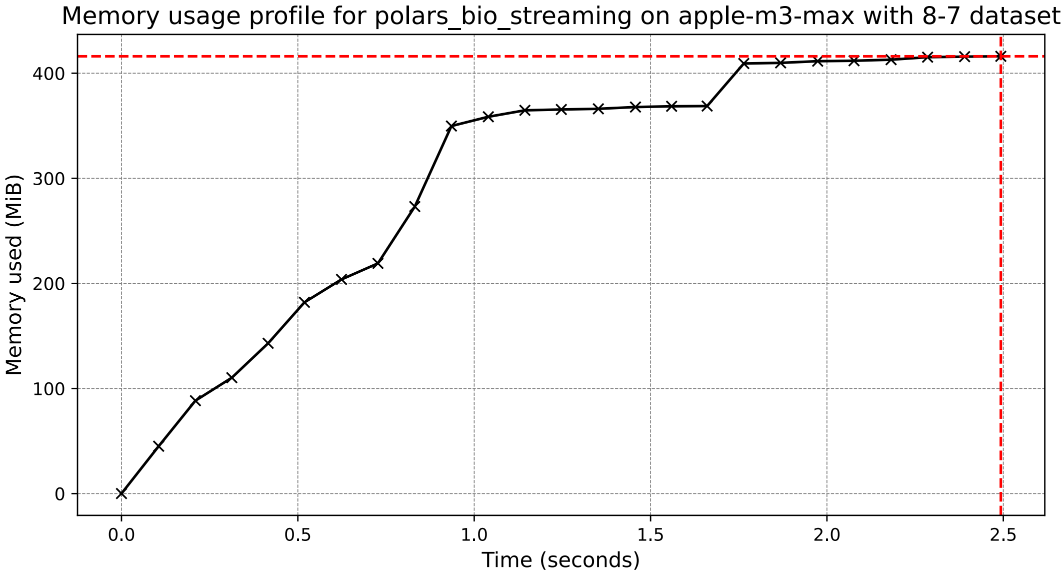

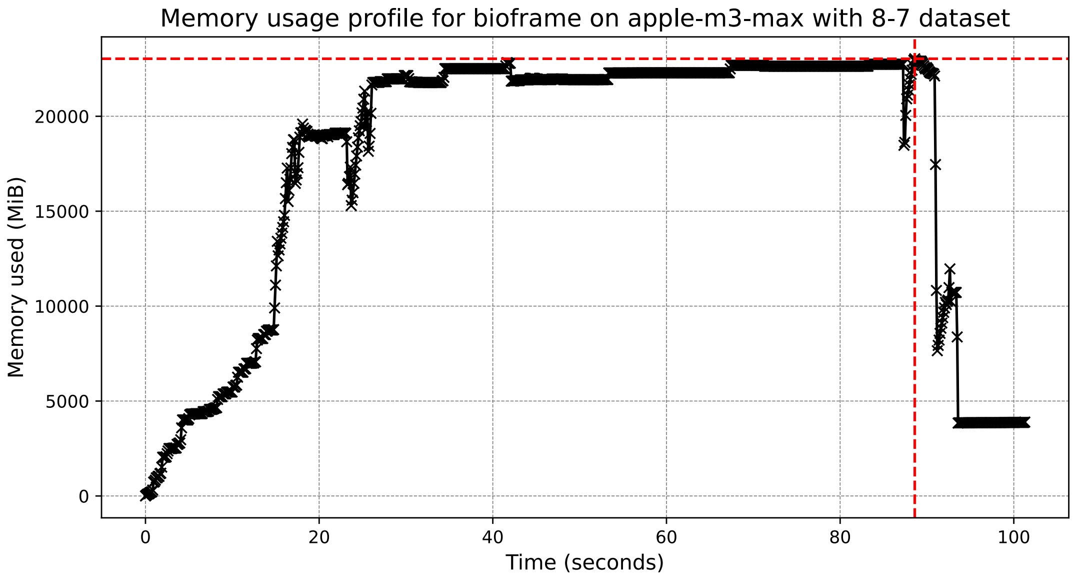
Memory usage profile for bioframe on apple-m3-max with 8-7 dataset
Memory used (MiB)
0
5000
10000
15000
20000
0
20
40
60
80
100
Time (seconds)

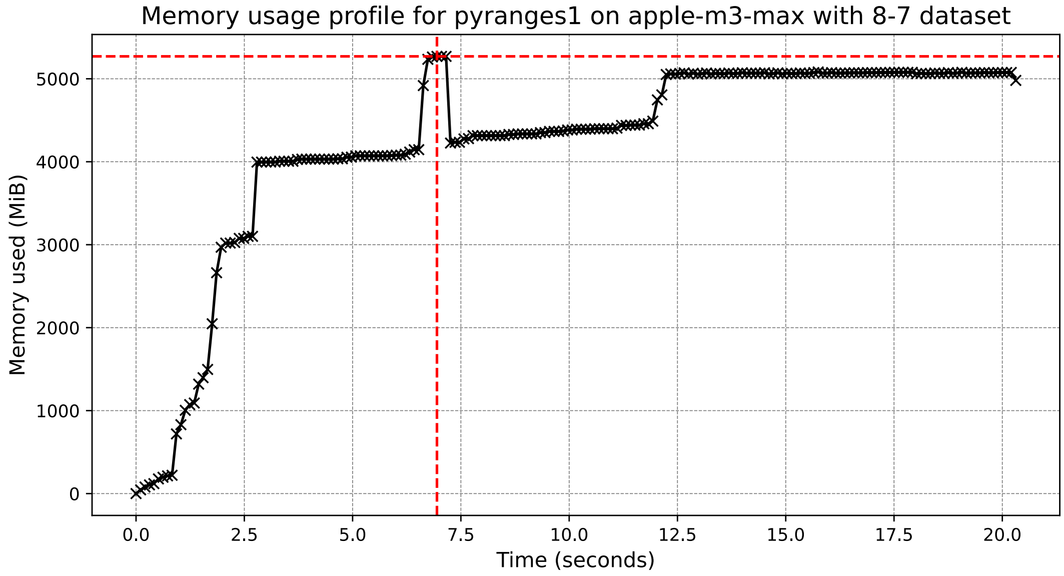
Memory usage profile for pyranges1 on apple-m3-max with 8-7 dataset
Memory used (MiB)
0
1000
2000
3000
4000
5000
0.0
2.5
5.0
7.5
10.0
12.5
15.0
17.5
20.0
Time (seconds)

#### 3.4.9 Operation: overlap for dataset: 100-1p on platform: apple-m3-max

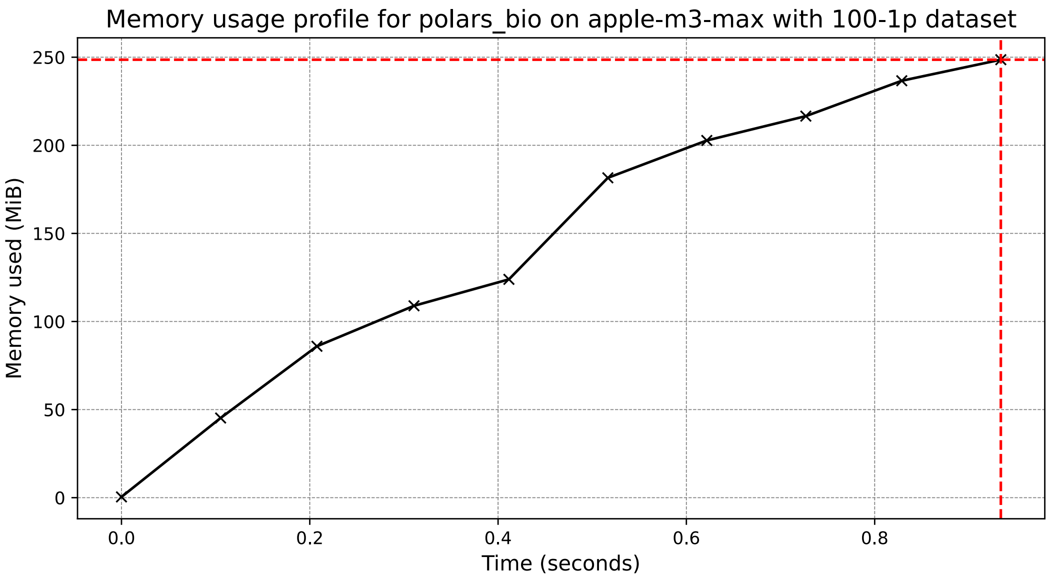


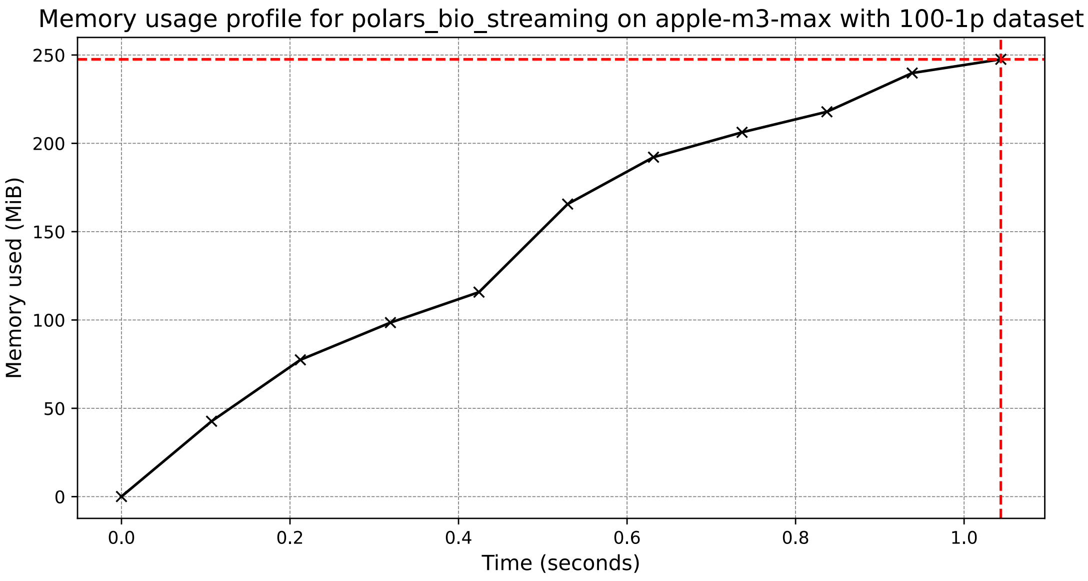

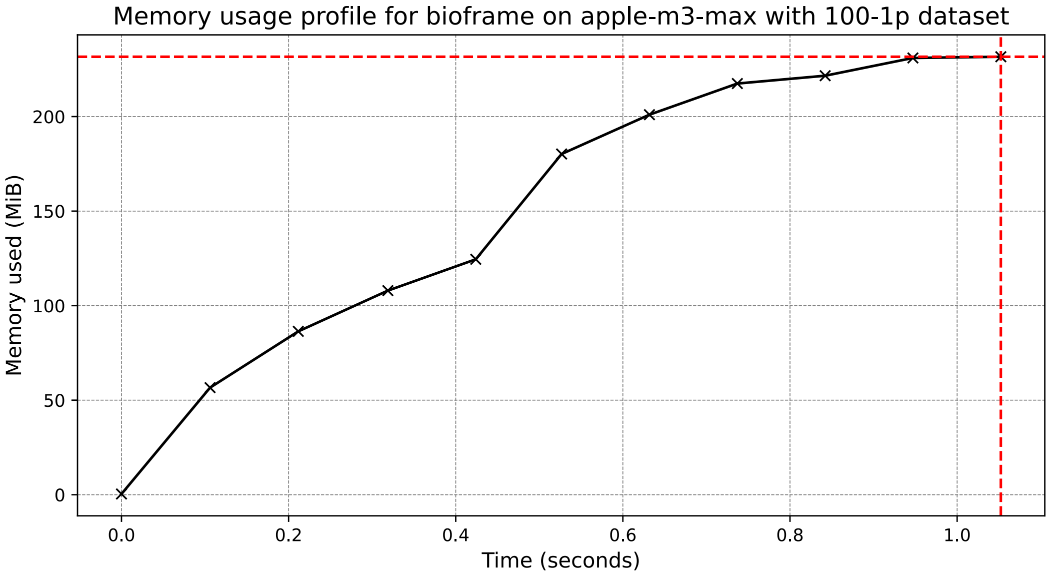
Memory usage profile for bioframe on apple-m3-max with 100-1p dataset
Memory used (MiB)
Time (seconds)
0
50
100
150
200
0.0
0.2
0.4
0.6
0.8
1.0

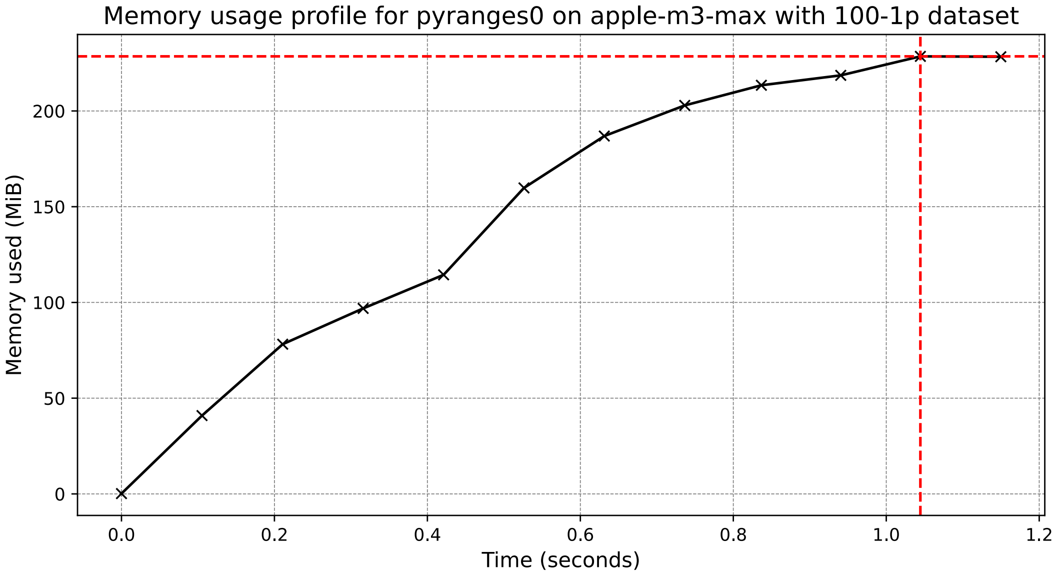
Memory usage profile for pyranges0 on apple-m3-max with 100-1p dataset
Memory used (MiB)
Time (seconds)
0
50
100
150
200
0.0
0.2
0.4
0.6
0.8
1.0
1.2

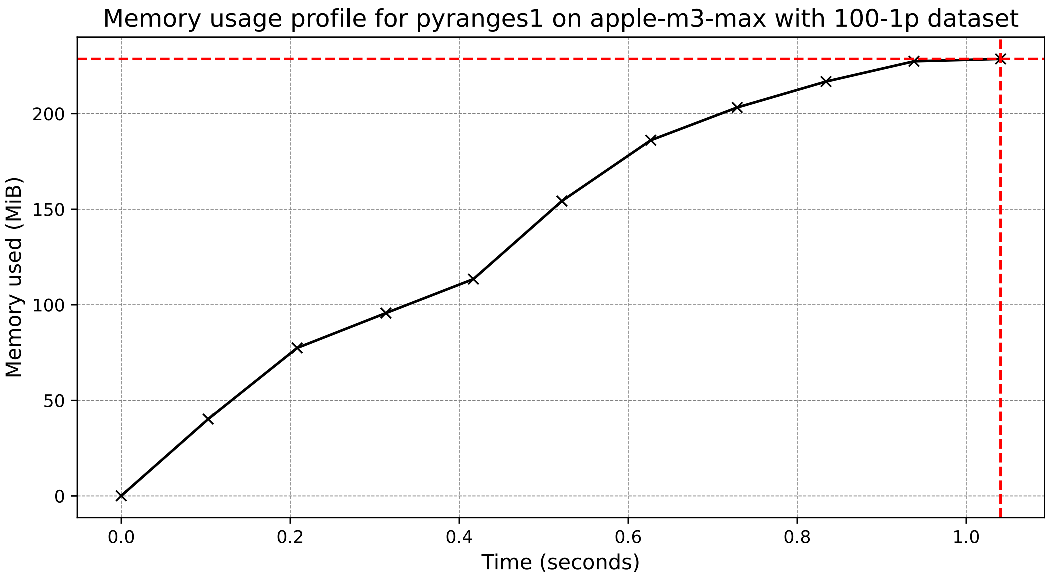


### 3.4.10 Operation: nearest for dataset: 100-1p on platform: apple-m3-max

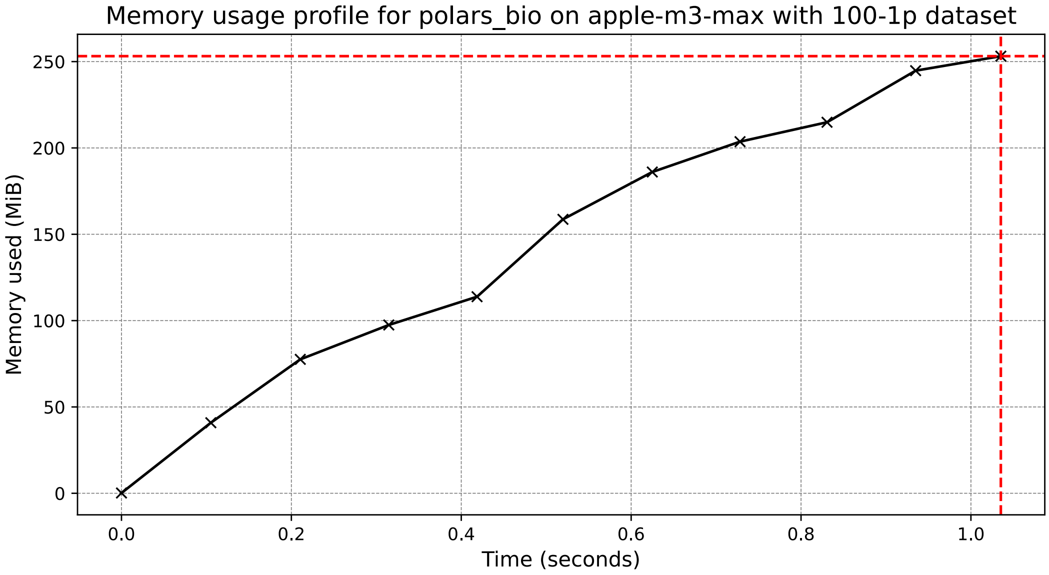

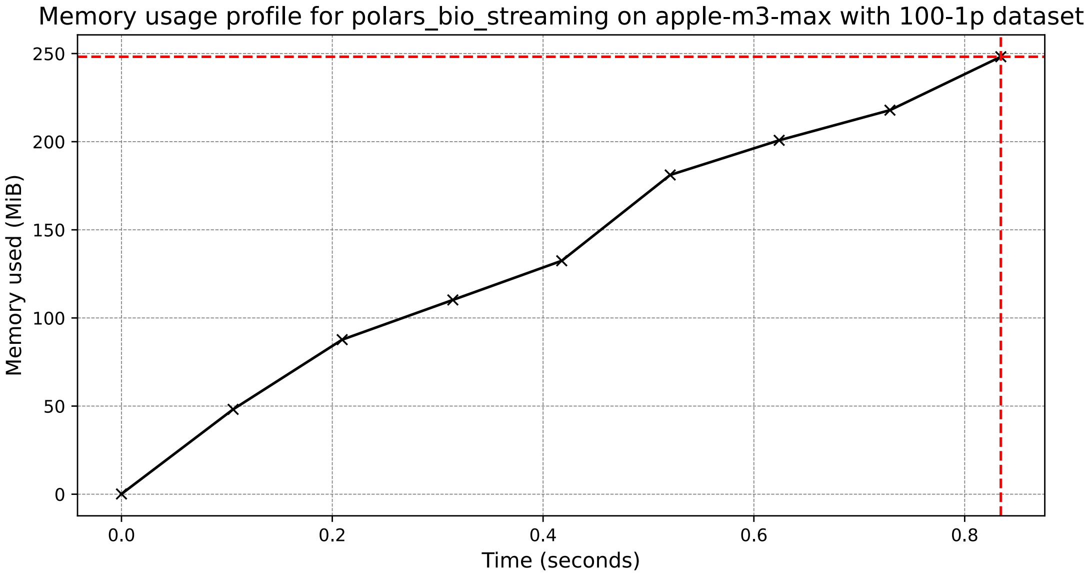
Memory usage profile for polars_bio_streaming on apple-m3-max with 100-1p dataset
Memory used (MiB)
Time (seconds)
0
50
100
150
200
250
0.0
0.2
0.4
0.6
0.8

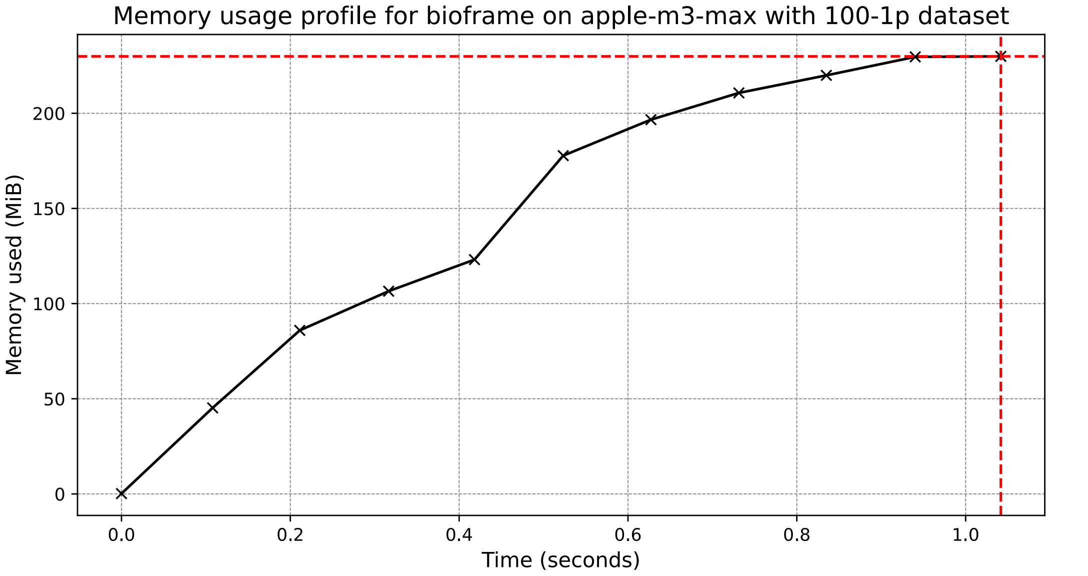
Memory usage profile for bioframe on apple-m3-max with 100-1p dataset
Memory used (MiB)
Time (seconds)
0
50
100
150
200
0.0
0.2
0.4
0.6
0.8
1.0

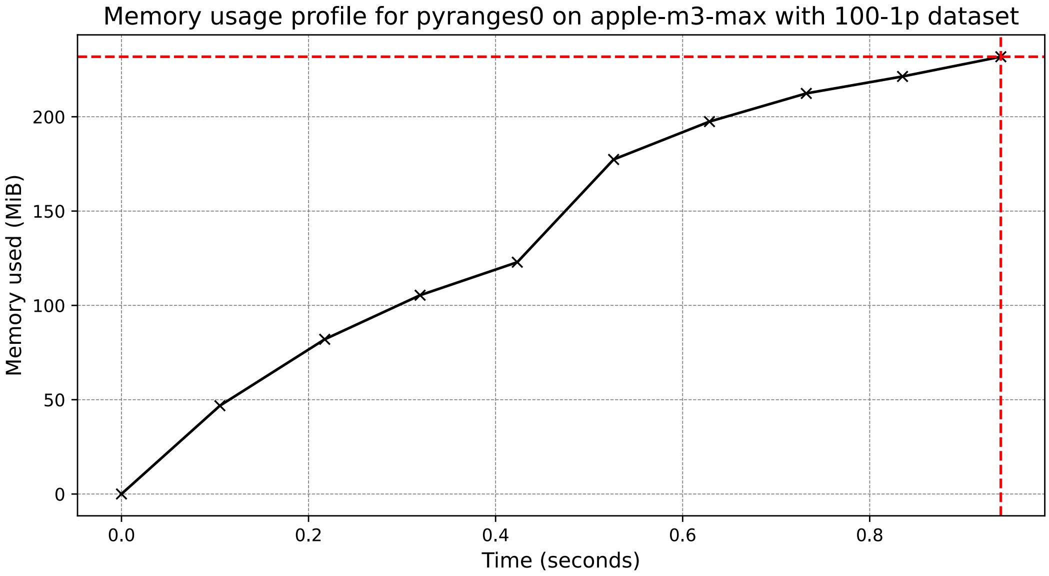
Memory usage profile for pyranges0 on apple-m3-max with 100-1p dataset
Memory used (MiB)
Time (seconds)
0
50
100
150
200
0.0
0.2
0.4
0.6
0.8

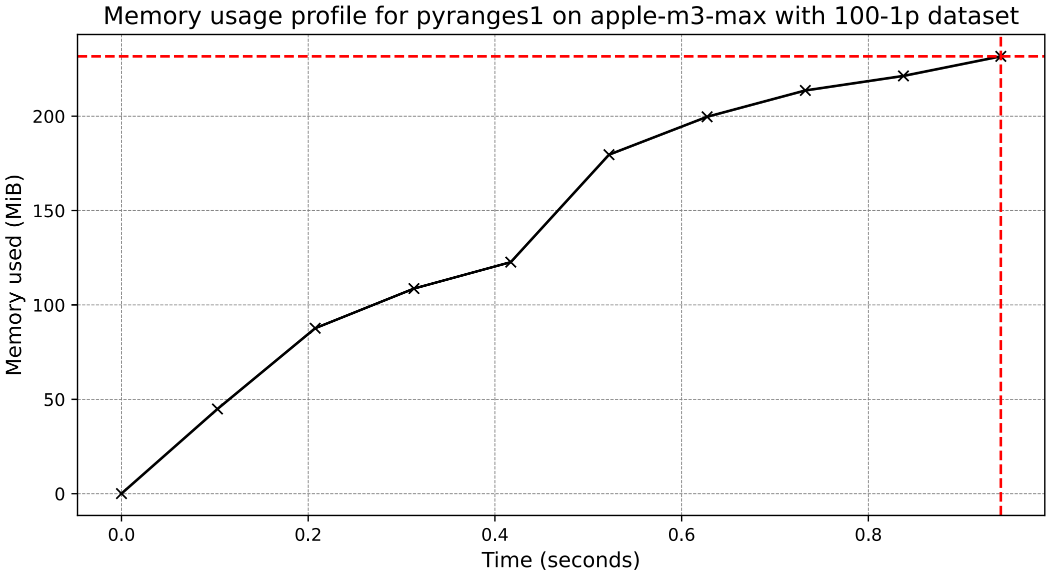
Memory usage profile for pyranges1 on apple-m3-max with 100-1p dataset
Memory used (MiB)
Time (seconds)
0
50
100
150
200
0.0
0.2
0.4
0.6
0.8

### 3.4.11 Operation: coverage for dataset: 100-1p on platform: apple-m3-max

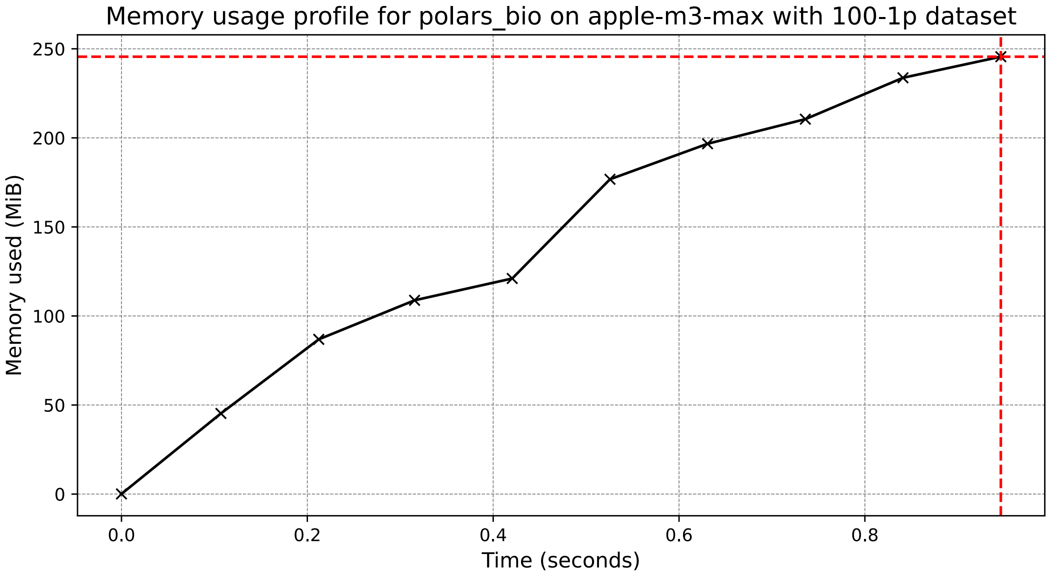


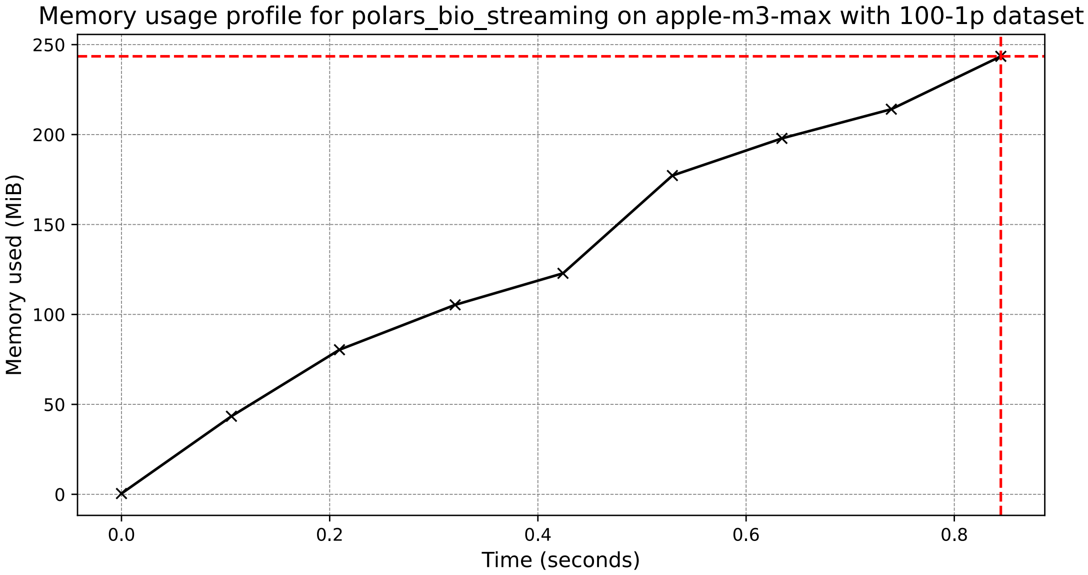

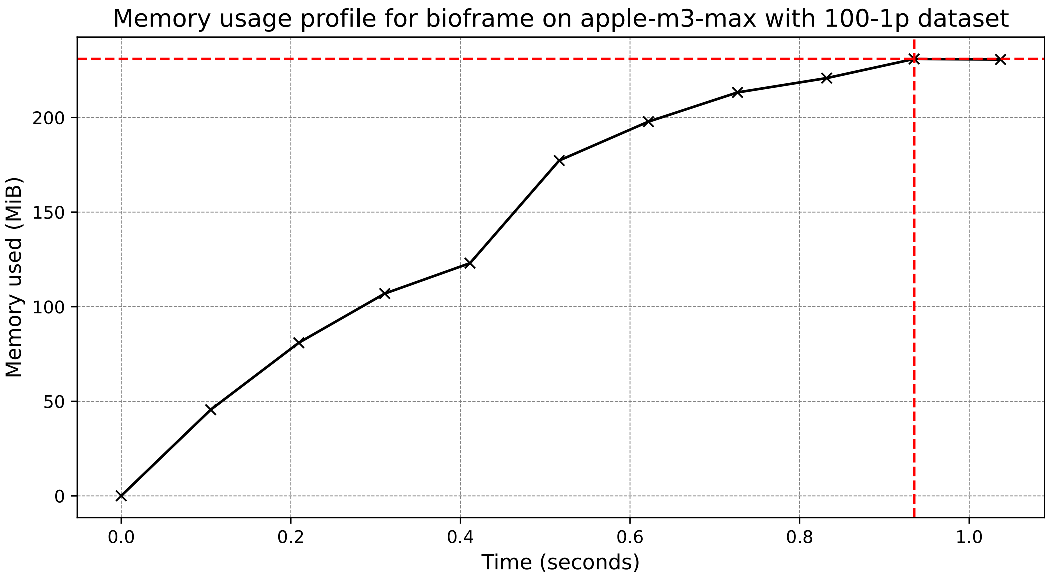
Memory usage profile for bioframe on apple-m3-max with 100-1p dataset
Memory used (MiB)
Time (seconds)
0
50
100
150
200
0.0
0.2
0.4
0.6
0.8
1.0

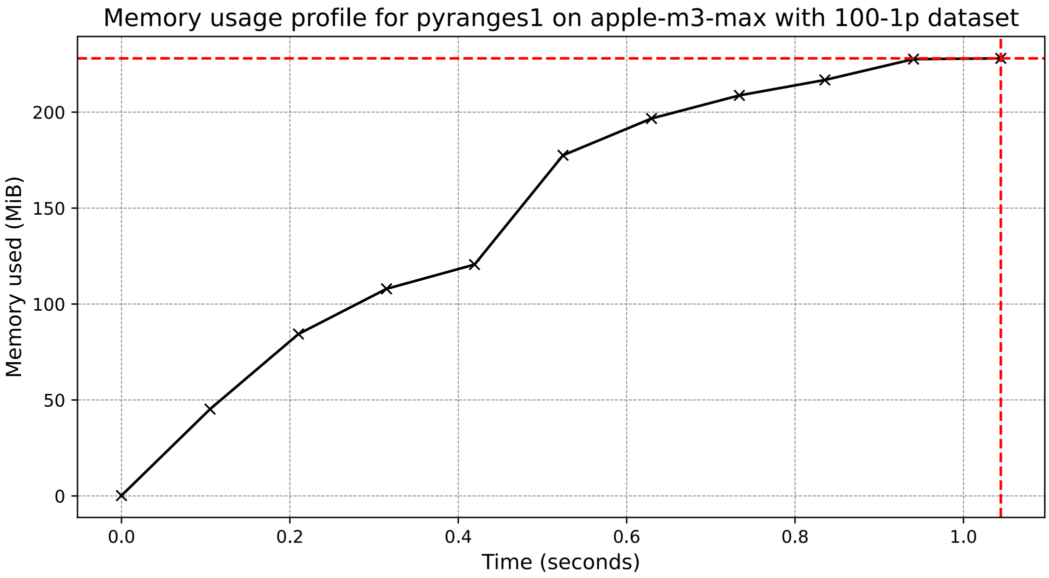
Memory usage profile for pyranges1 on apple-m3-max with 100-1p dataset
Memory used (MiB)
Time (seconds)
0
50
100
150
200
0.0
0.2
0.4
0.6
0.8
1.0

### 3.4.12 Operation: count-overlaps for dataset: 100-1p on platform: apple-m3-max

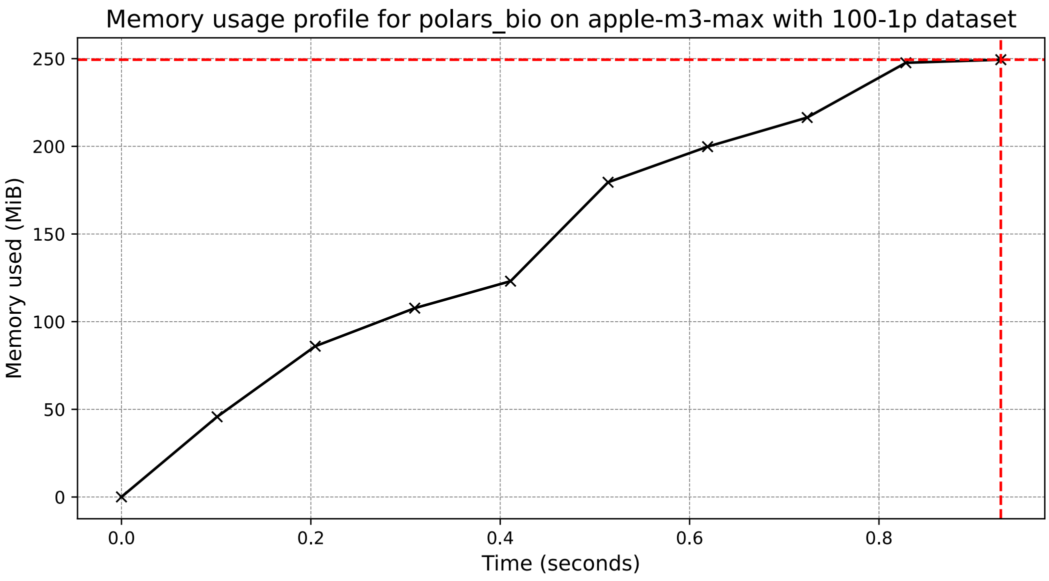


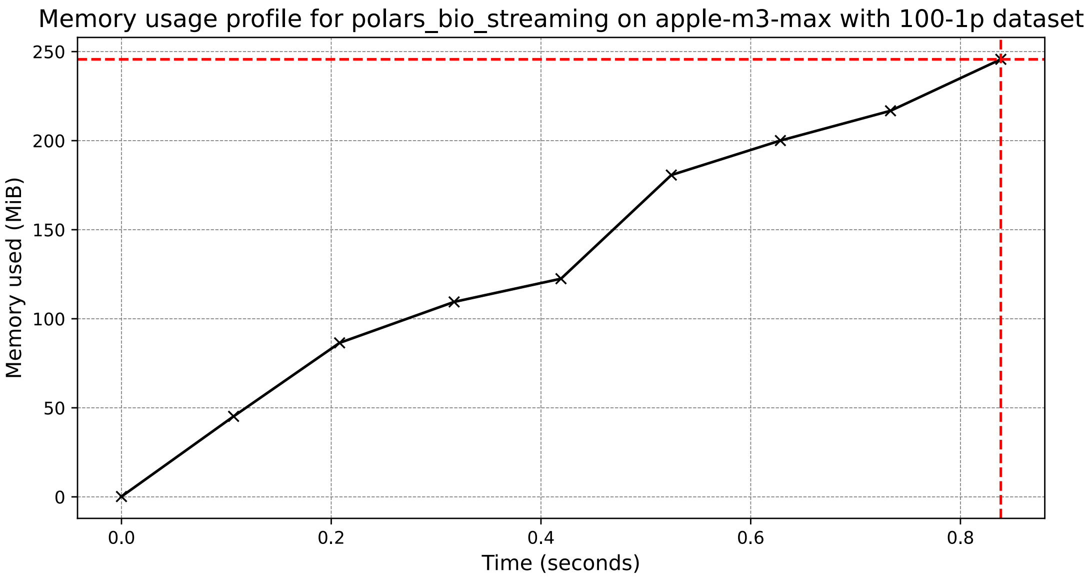

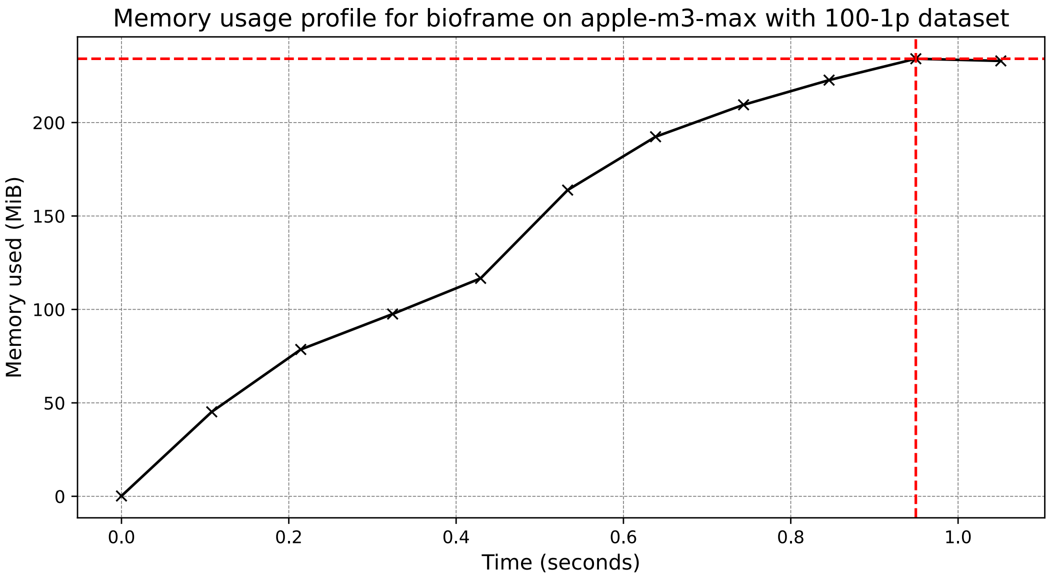
Memory usage profile for bioframe on apple-m3-max with 100-1p dataset
Memory used (MiB)
Time (seconds)
0
50
100
150
200
0.0
0.2
0.4
0.6
0.8
1.0

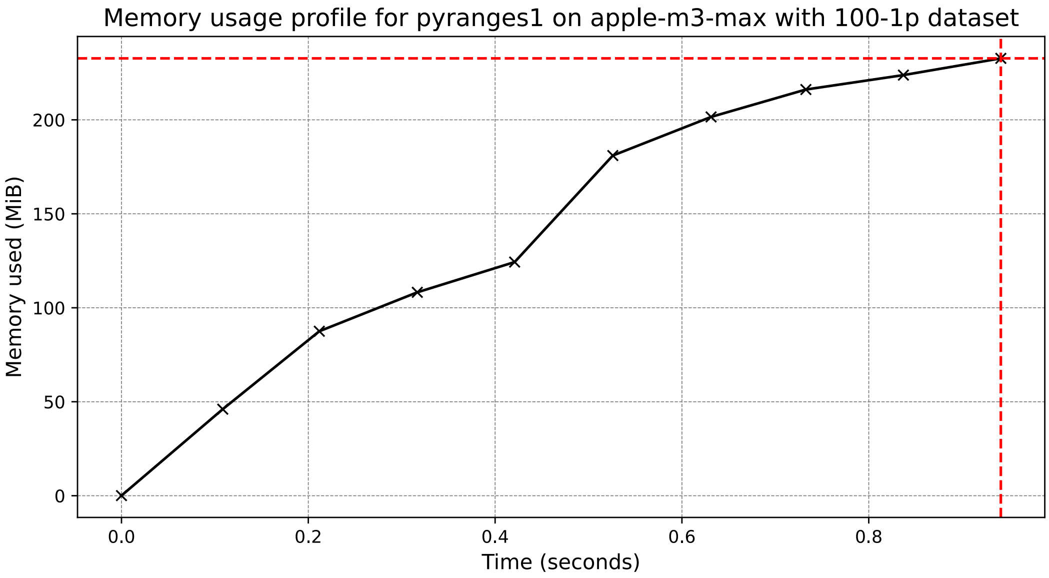
Memory usage profile for pyranges1 on apple-m3-max with 100-1p dataset
Memory used (MiB)
Time (seconds)
0
50
100
150
200
0.0
0.2
0.4
0.6
0.8

### 3.4.13 Operation: overlap for dataset: 10000000-1p on platform: apple-m3-max

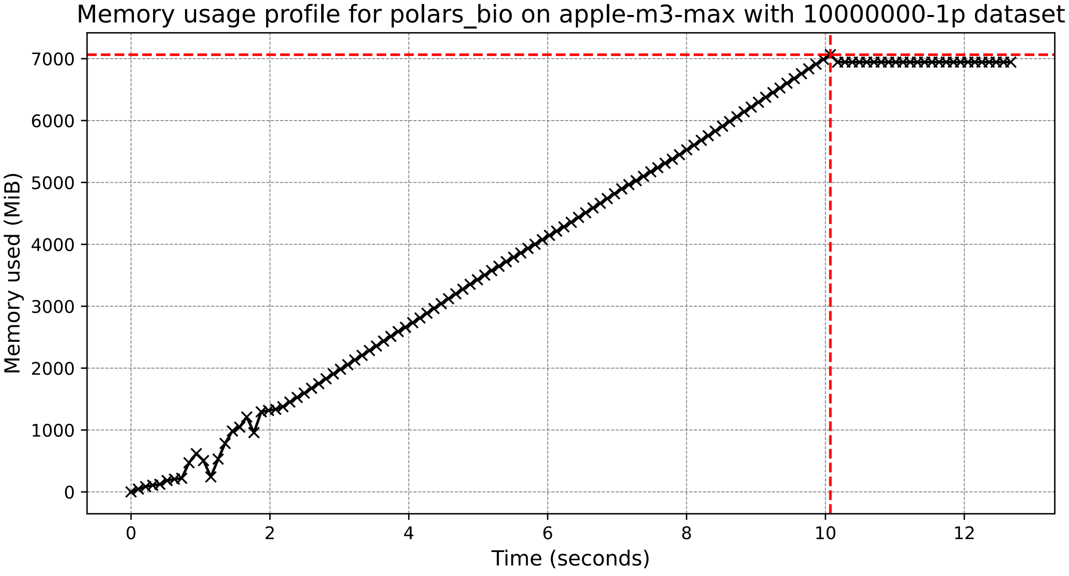


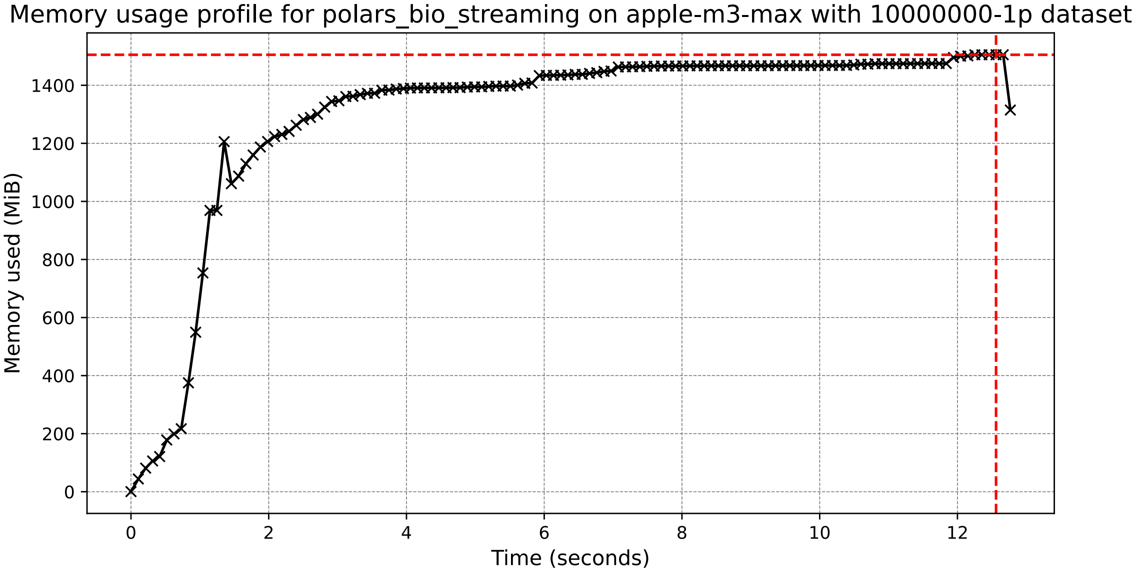

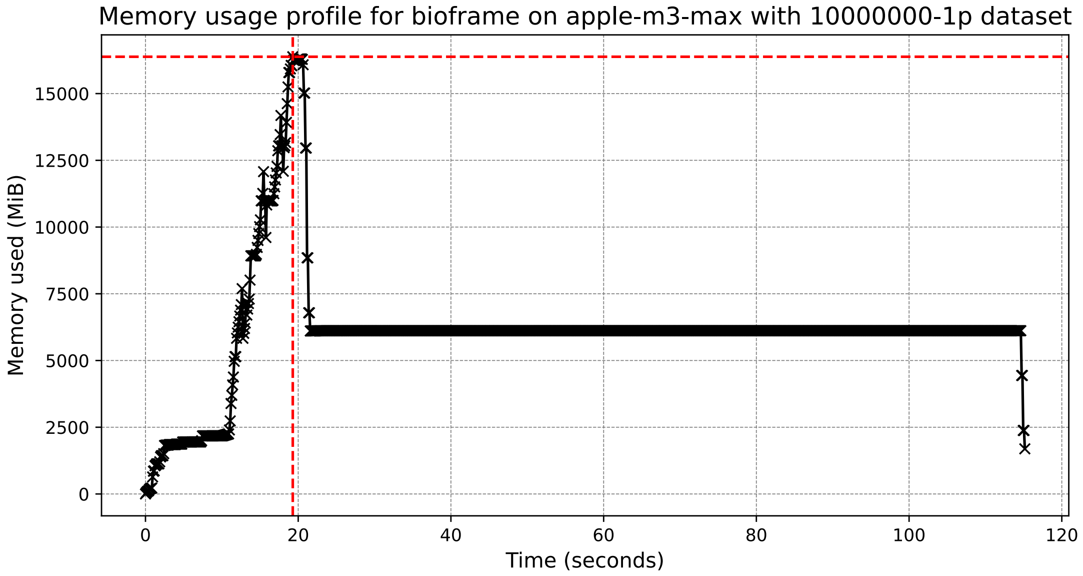
Memory usage profile for bioframe on apple-m3-max with 10000000-1p dataset
Memory used (MiB)
0
2500
5000
7500
10000
12500
15000
Time (seconds)
0
20
40
60
80
100
120

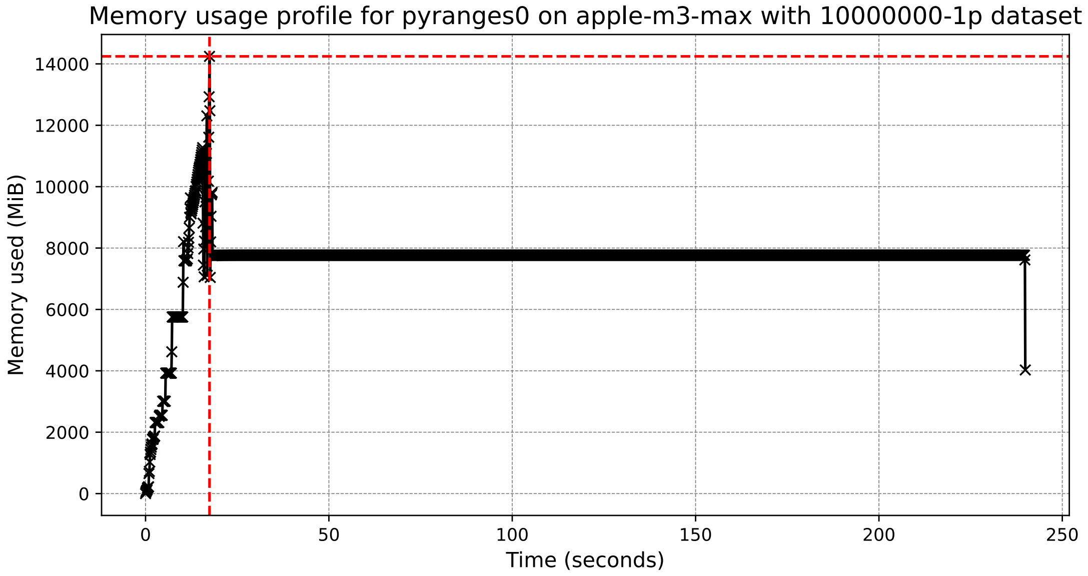
Memory usage profile for pyranges0 on apple-m3-max with 10000000-1p dataset
Memory used (MiB)
0
2000
4000
6000
8000
10000
12000
14000
Time (seconds)
0
50
100
150
200
250

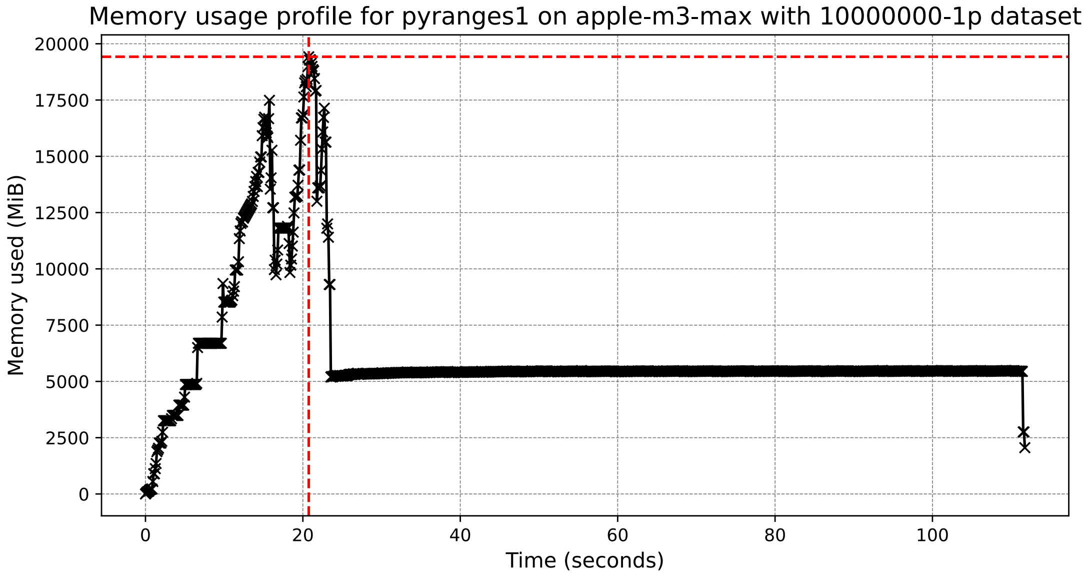


### 3.4.14 Operation: nearest for dataset: 10000000-1p on platform: apple-m3-max

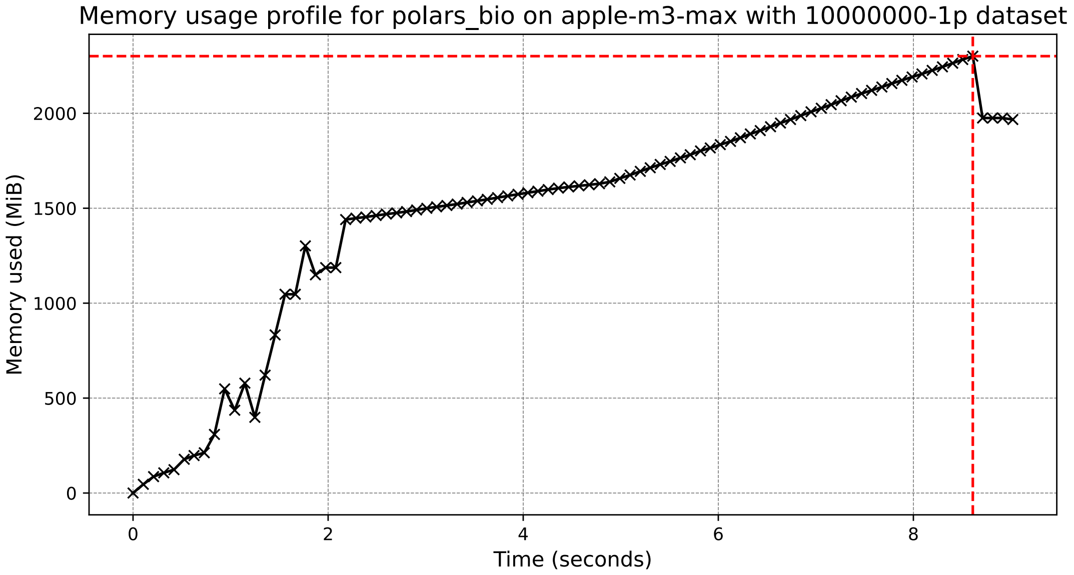

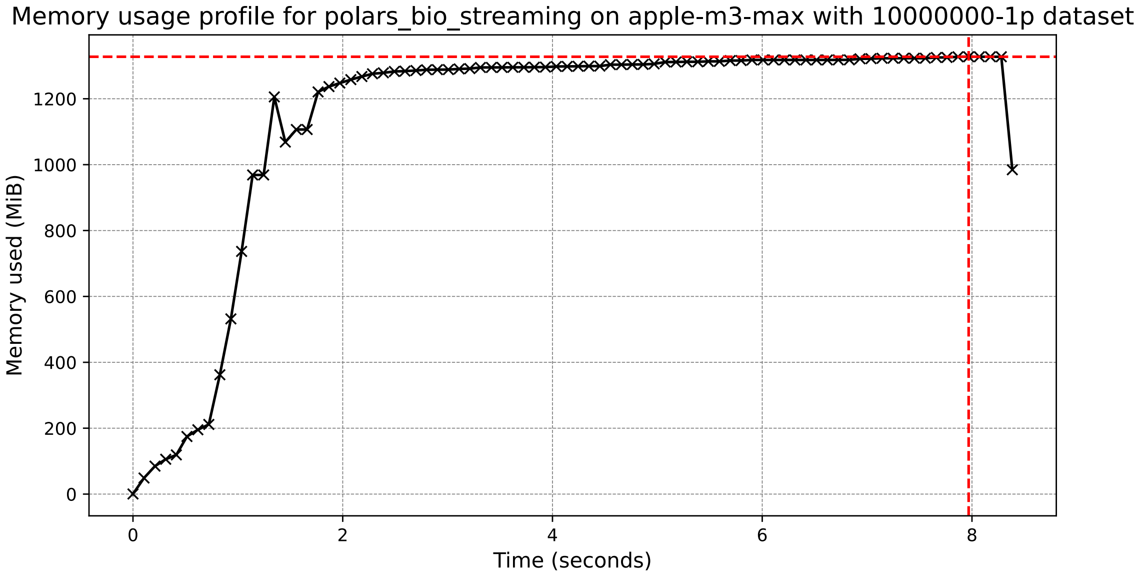
Memory usage profile for polars_bio_streaming on apple-m3-max with 10000000-1p dataset
Memory used (MiB)
Time (seconds)
0
200
400
600
800
1000
1200
0
2
4
6
8

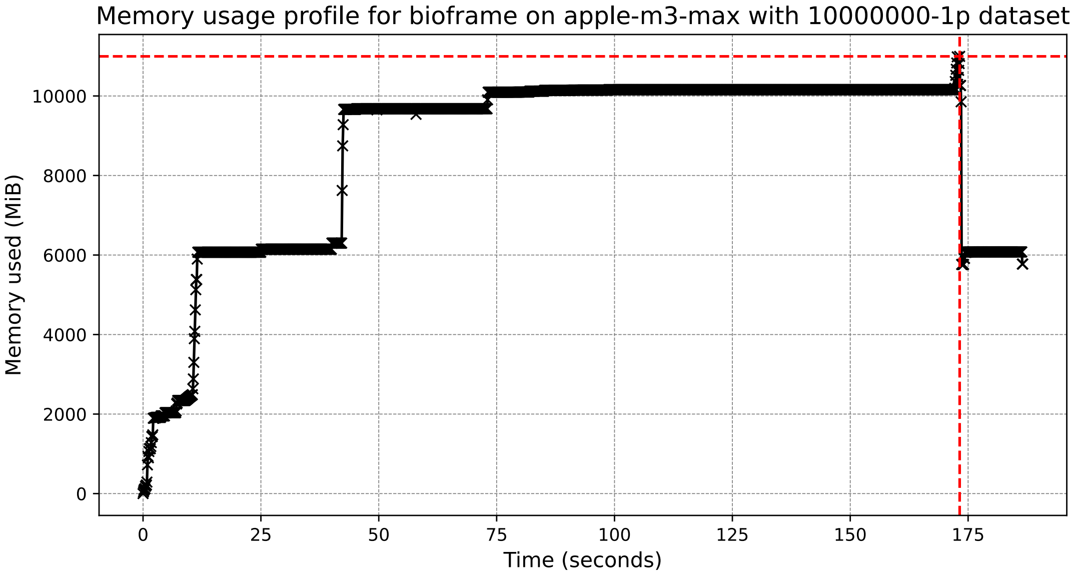
Memory usage profile for bioframe on apple-m3-max with 10000000-1p dataset
Memory used (MiB)
Time (seconds)
0
2000
4000
6000
8000
10000
0
25
50
75
100
125
150
175

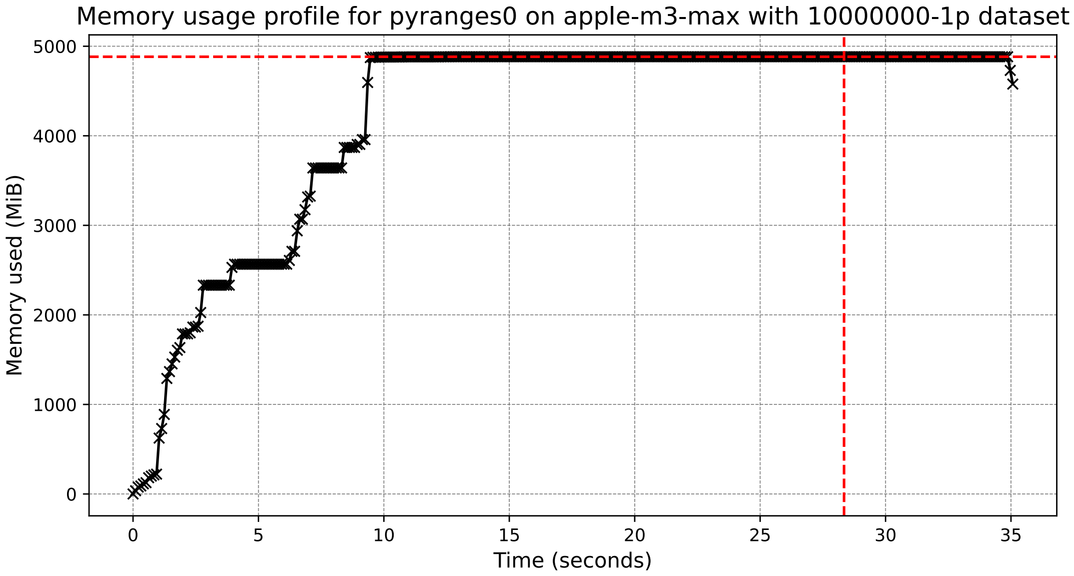
Memory usage profile for pyranges0 on apple-m3-max with 10000000-1p dataset
Memory used (MiB)
Time (seconds)
0
1000
2000
3000
4000
5000
0
5
10
15
20
25
30
35

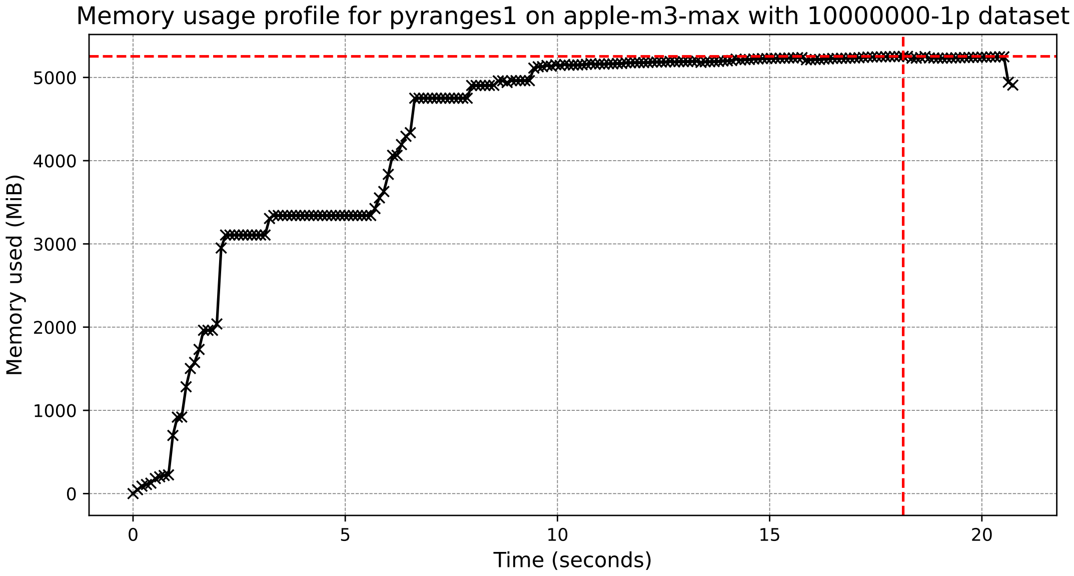
Memory usage profile for pyranges1 on apple-m3-max with 10000000-1p dataset
Memory used (MiB)
Time (seconds)
0
1000
2000
3000
4000
5000
0
5
10
15
20

#### 3.4.15 Operation: coverage for dataset: 10000000-1p on platform: apple-m3-max

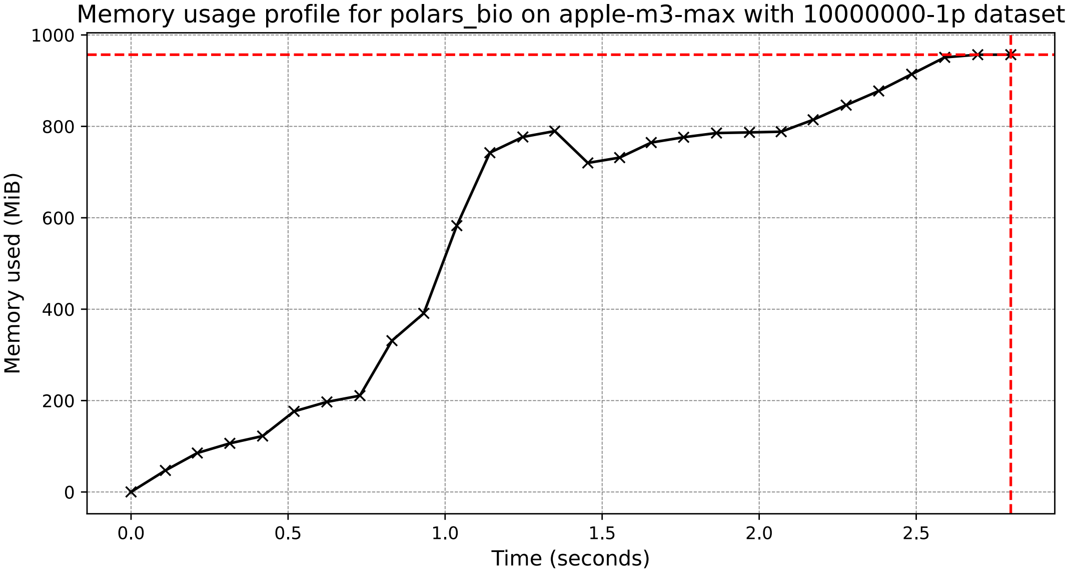


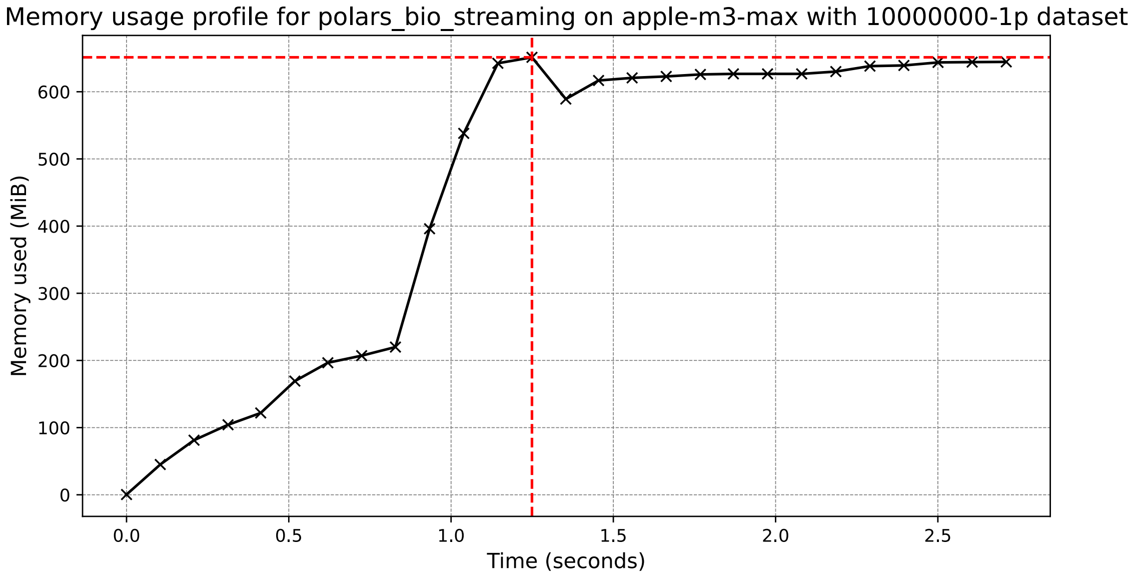

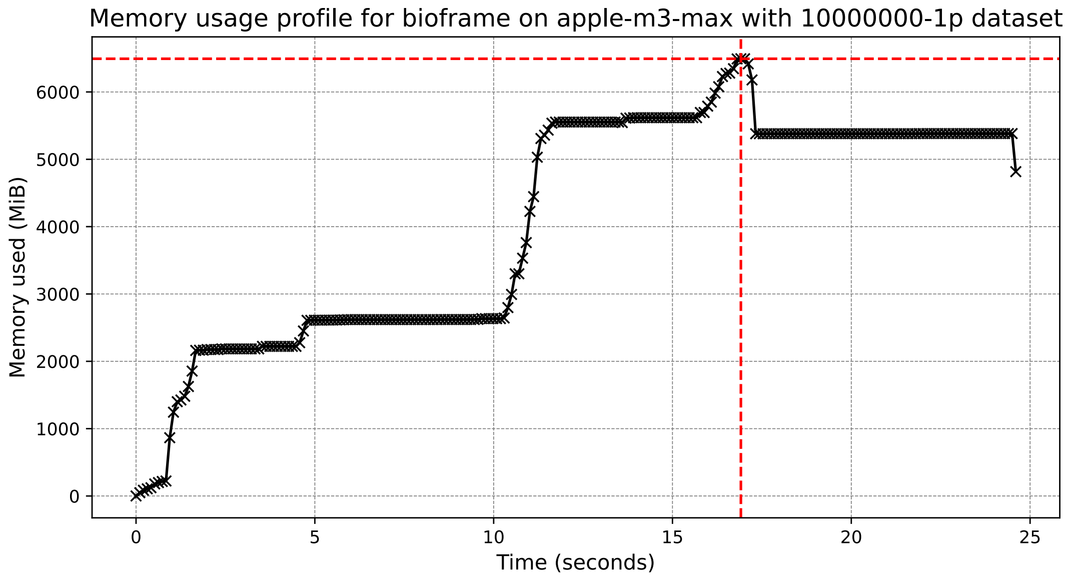
Memory usage profile for bioframe on apple-m3-max with 10000000-1p dataset
Memory used (MiB)
Time (seconds)
0
1000
2000
3000
4000
5000
6000
0
5
10
15
20
25

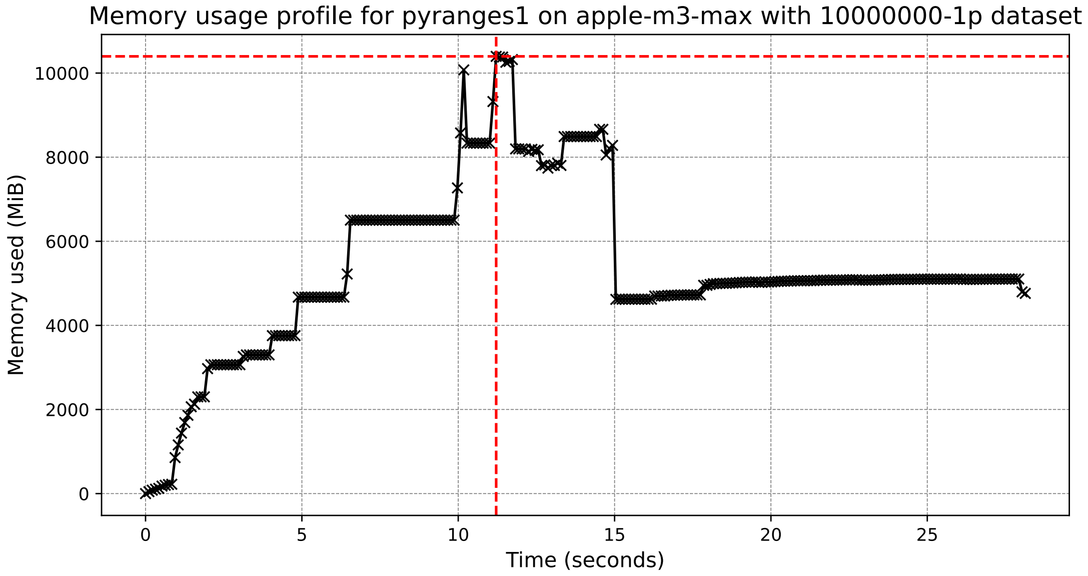
Memory usage profile for pyranges1 on apple-m3-max with 10000000-1p dataset
Memory used (MiB)
Time (seconds)
0
2000
4000
6000
8000
10000
0
5
10
15
20
25

### 3.4.16 Operation: count-overlaps for dataset: 10000000-1p on platform: apple-m3-max

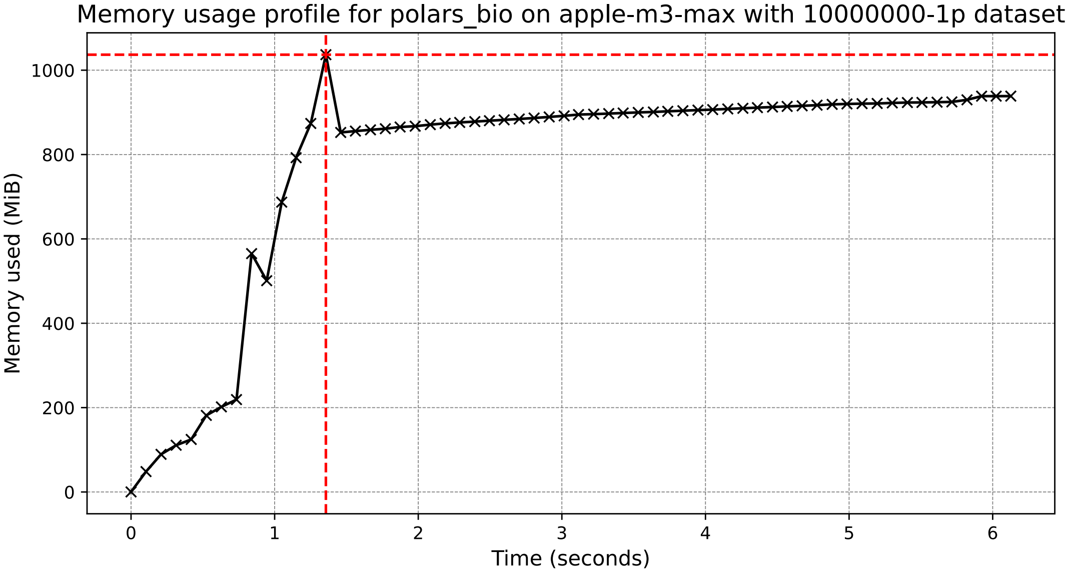


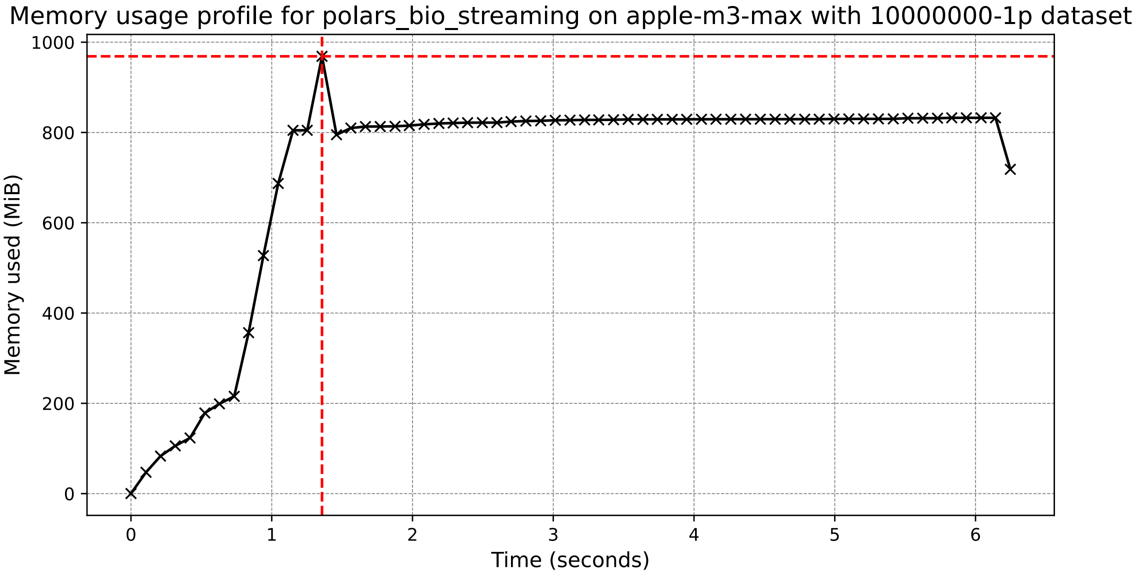

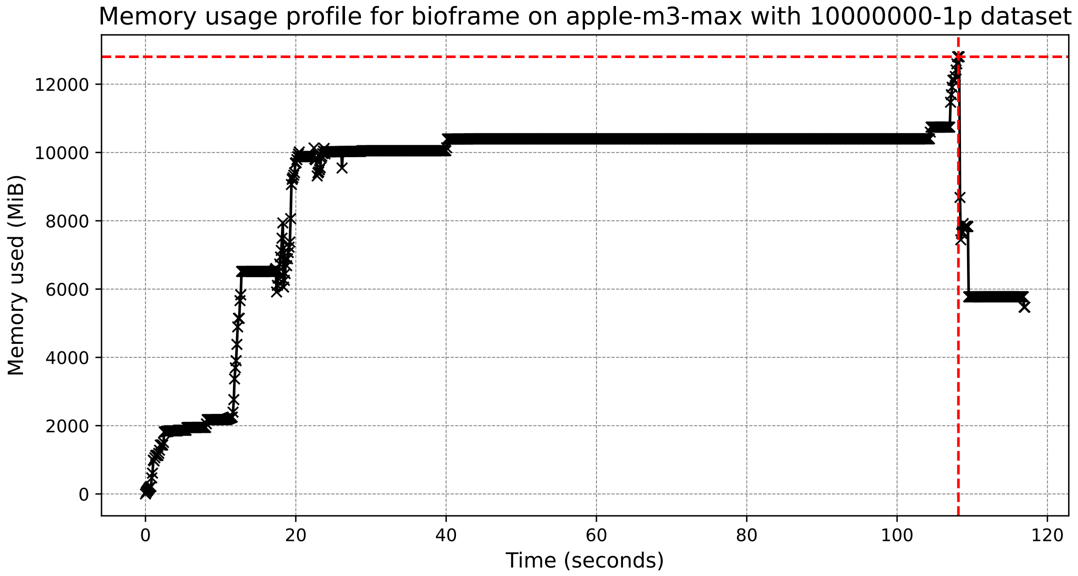
Memory usage profile for bioframe on apple-m3-max with 10000000-1p dataset
Memory used (MiB)
Time (seconds)

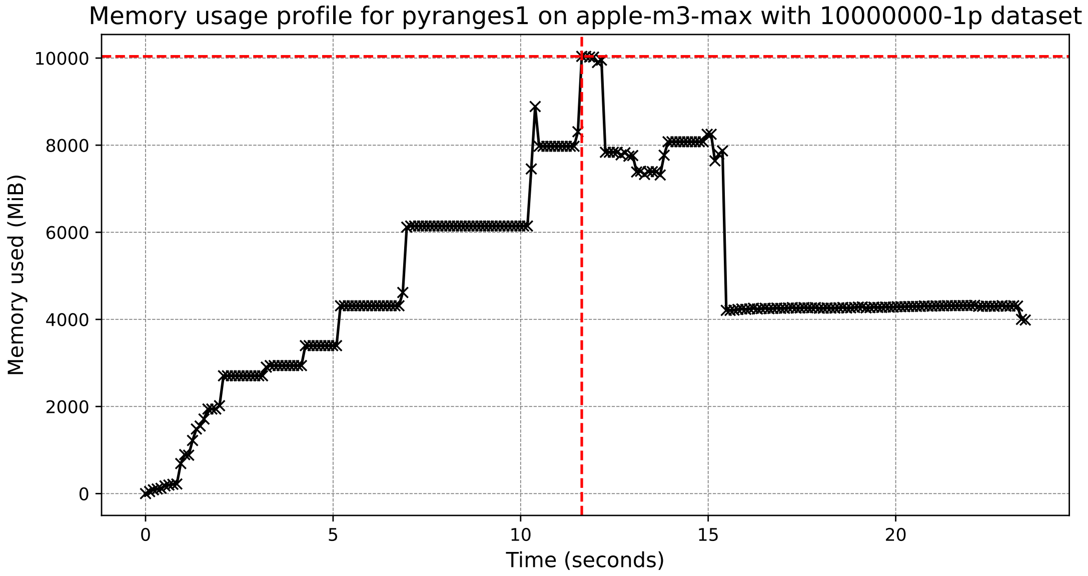
Memory usage profile for pyranges1 on apple-m3-max with 10000000-1p dataset
Memory used (MiB)
Time (seconds)

### 3.4.17 Operation: overlap for dataset: fBrain-vs-exons on platform: gcp-linux

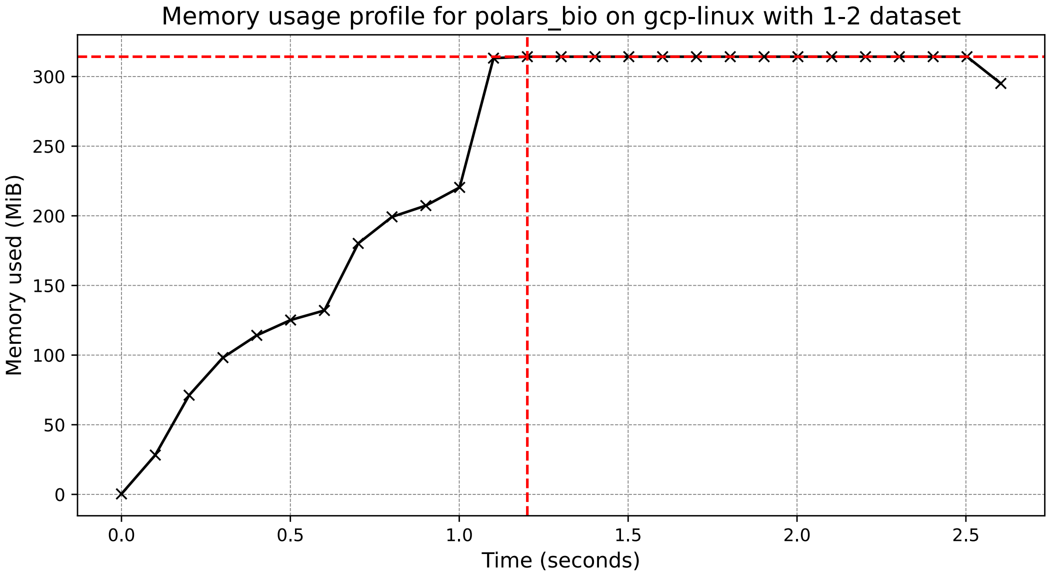


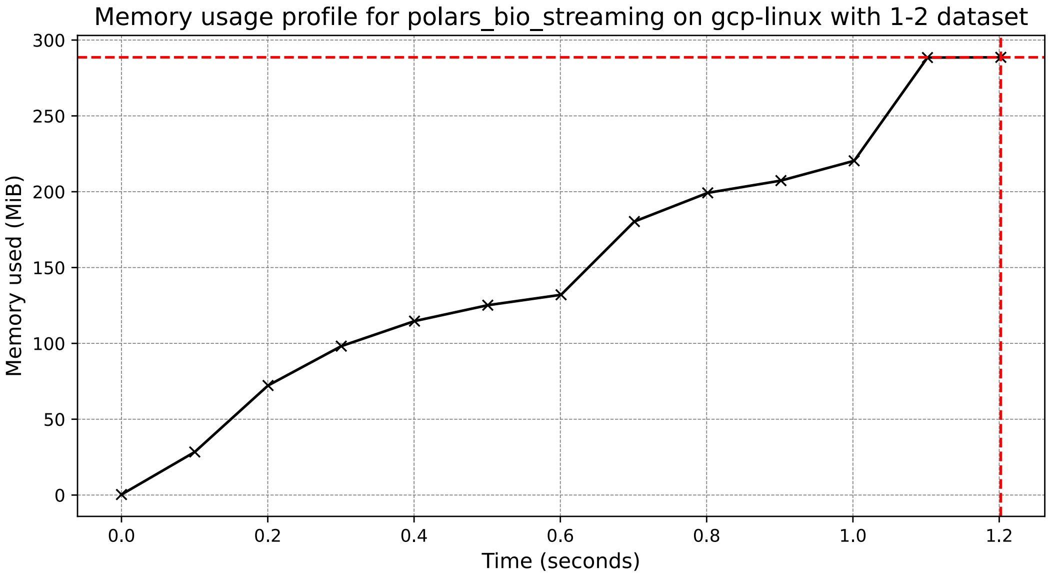

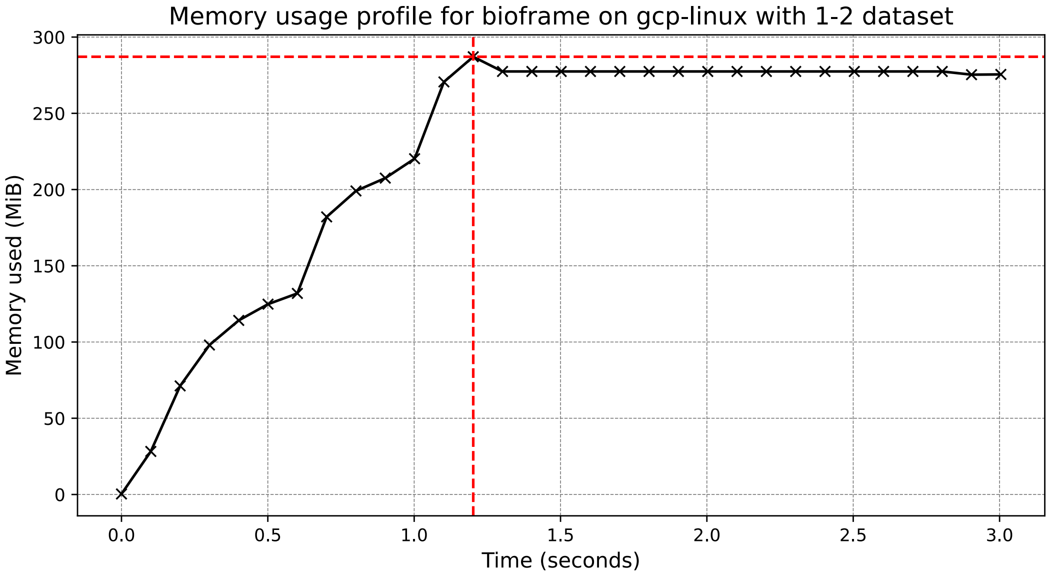
Memory usage profile for bioframe on gcp-linux with 1-2 dataset
Memory used (MiB)
Time (seconds)
0
50
100
150
200
250
300
0.0
0.5
1.0
1.5
2.0
2.5
3.0

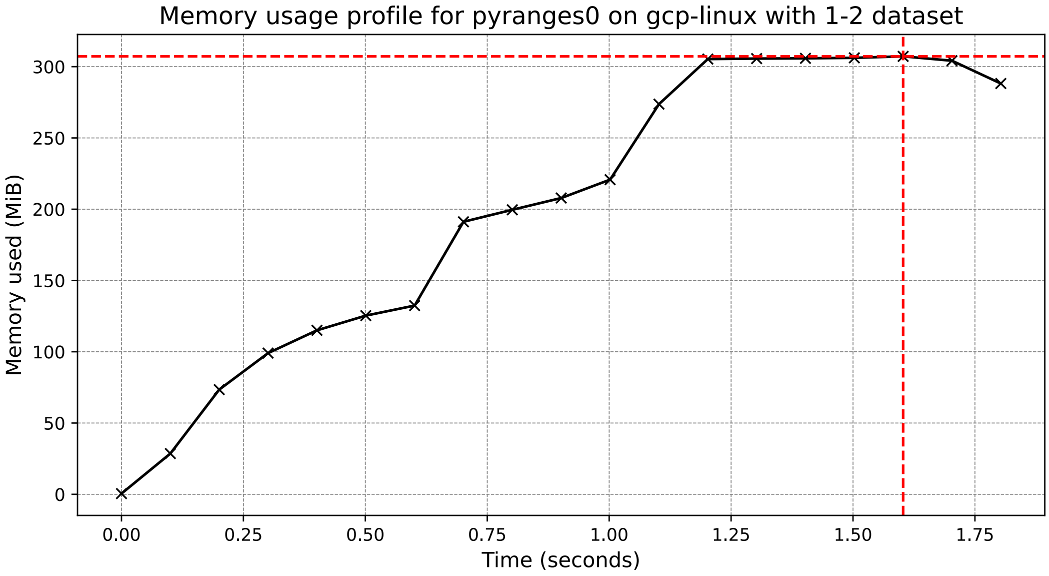
Memory usage profile for pyranges0 on gcp-linux with 1-2 dataset
Memory used (MiB)
Time (seconds)
0
50
100
150
200
250
300
0.00
0.25
0.50
0.75
1.00
1.25
1.50
1.75

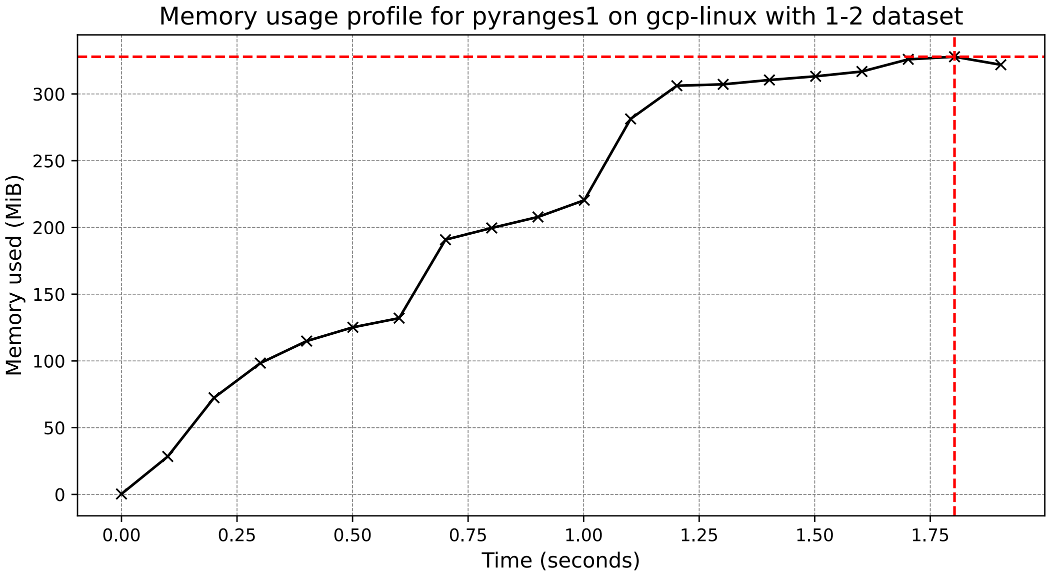


### 3.4.18 Operation: nearest for dataset: fBrain-vs-exons on platform: gcp-linux

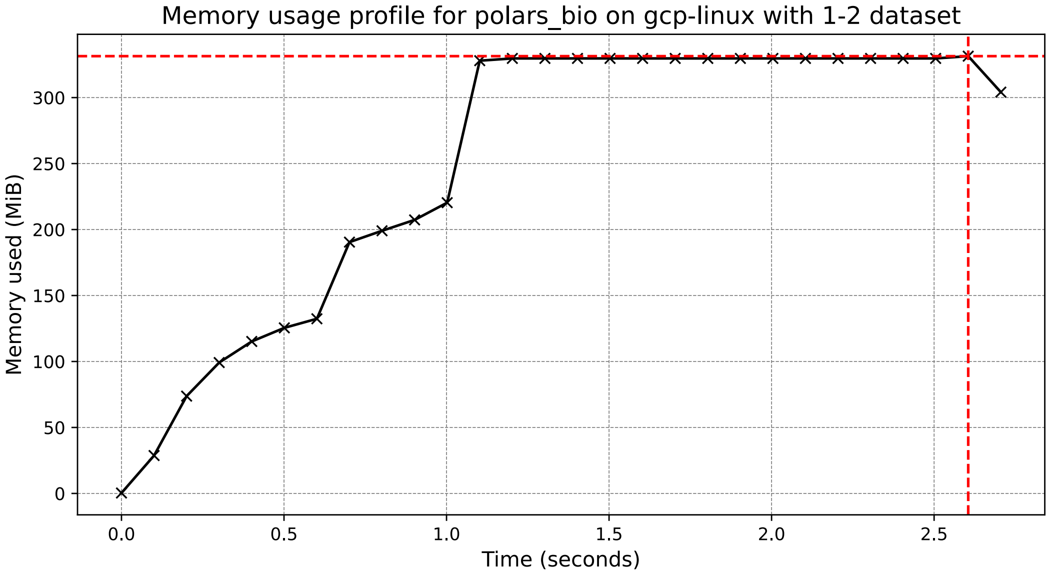

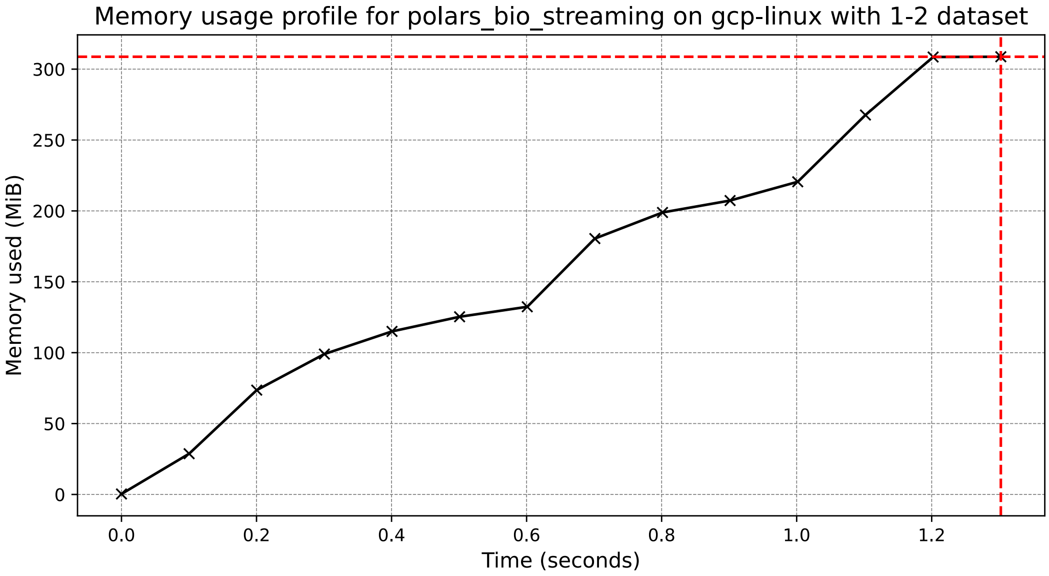
Memory usage profile for polars_bio_streaming on gcp-linux with 1-2 dataset
Memory used (MiB)
Time (seconds)
0
50
100
150
200
250
300
0.0
0.2
0.4
0.6
0.8
1.0
1.2

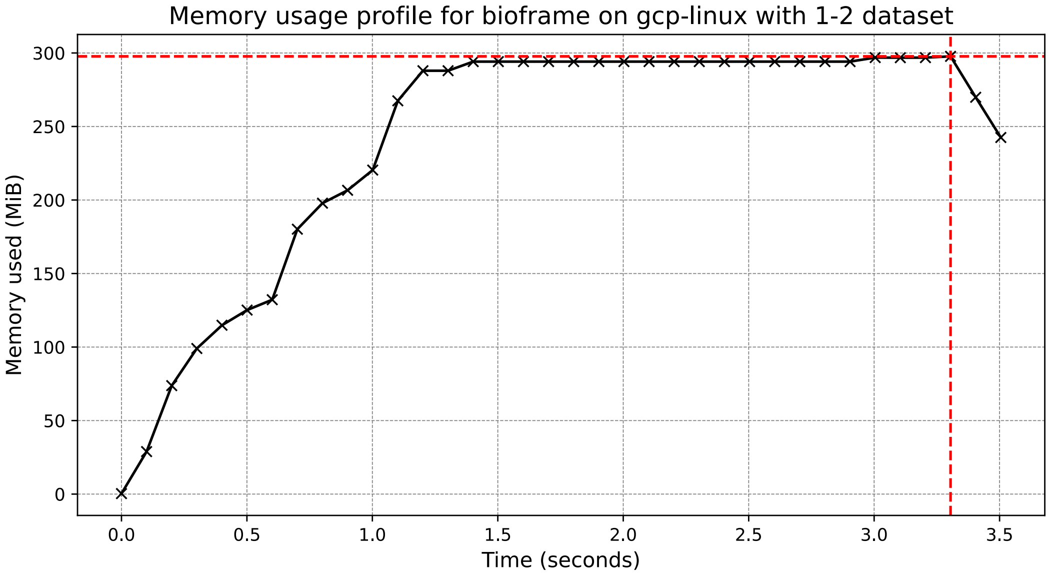
Memory usage profile for bioframe on gcp-linux with 1-2 dataset
Memory used (MiB)
Time (seconds)
0
50
100
150
200
250
300
0.0
0.5
1.0
1.5
2.0
2.5
3.0
3.5

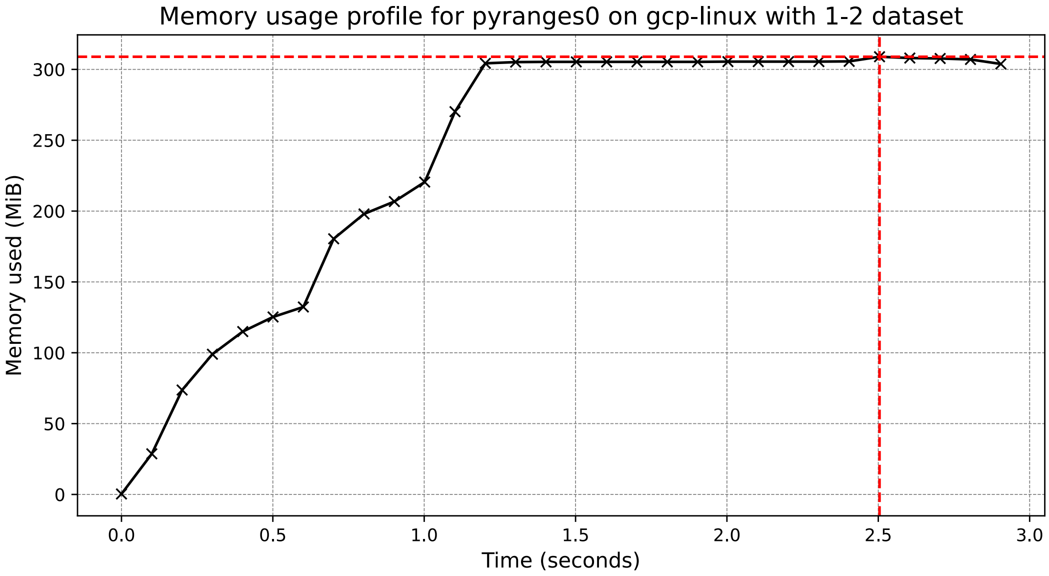
Memory usage profile for pyranges0 on gcp-linux with 1-2 dataset
Memory used (MiB)
Time (seconds)
0
50
100
150
200
250
300
0.0
0.5
1.0
1.5
2.0
2.5
3.0

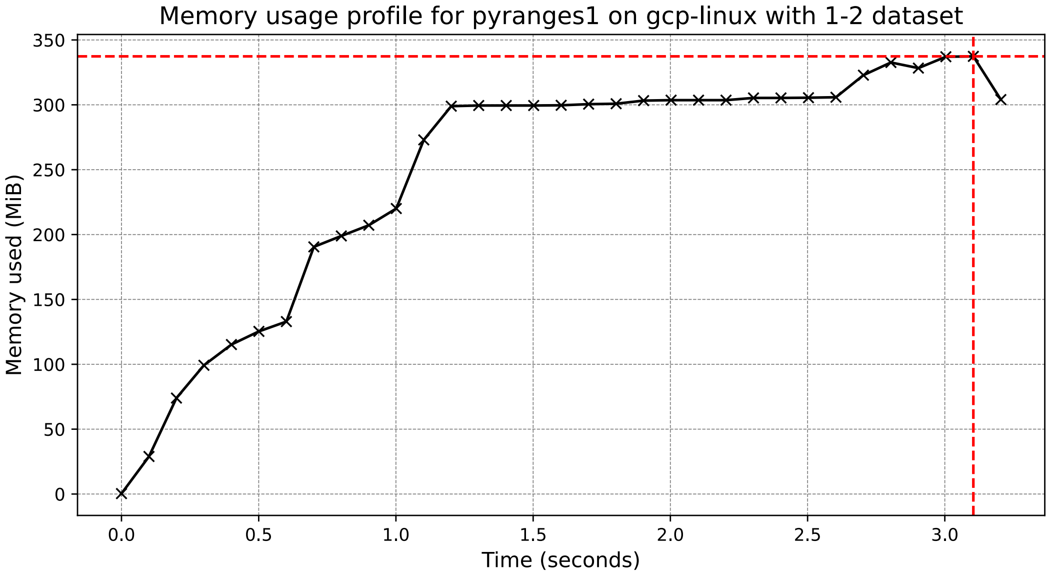
Memory usage profile for pyranges1 on gcp-linux with 1-2 dataset
Memory used (MiB)
Time (seconds)
0
50
100
150
200
250
300
350
0.0
0.5
1.0
1.5
2.0
2.5
3.0

### 3.4.19 Operation: coverage for dataset: fBrain-vs-exons on platform: gcp-linux

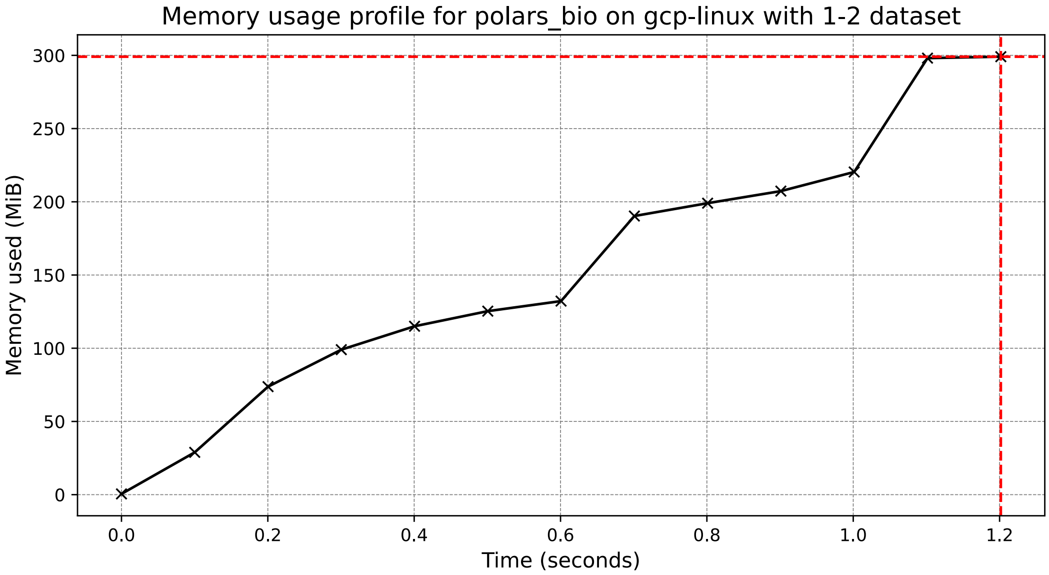


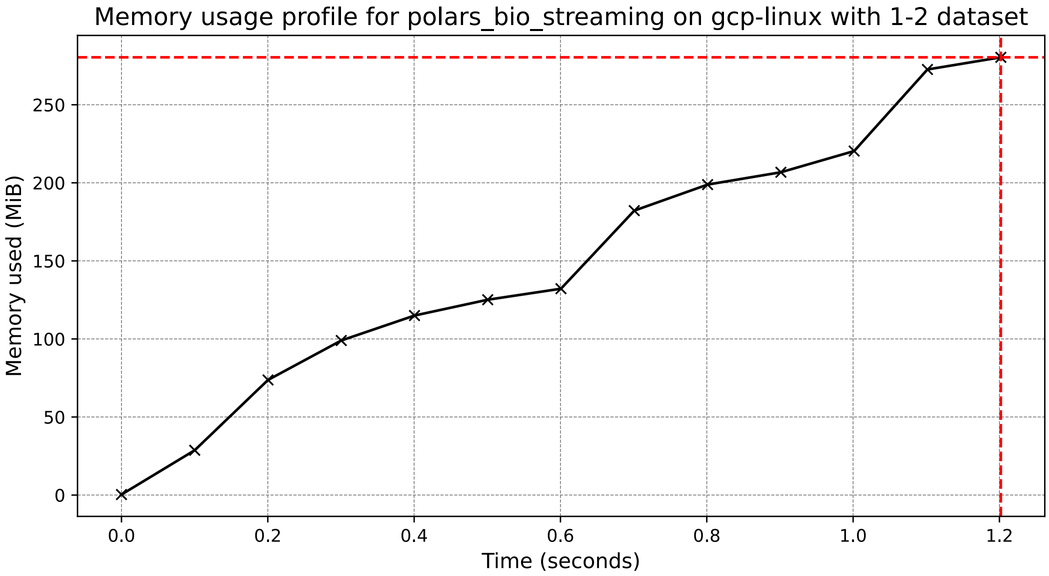

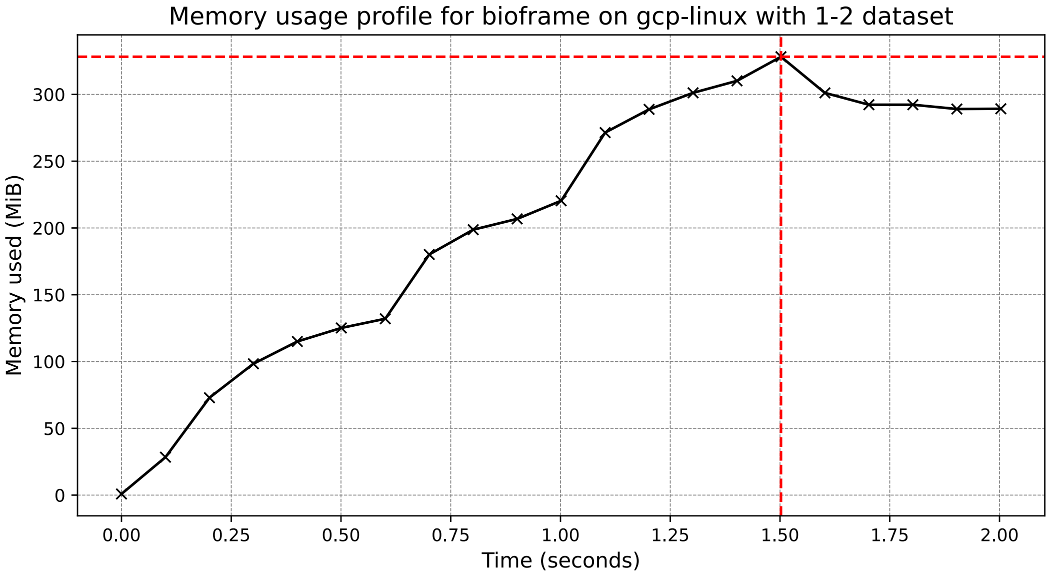
Memory usage profile for bioframe on gcp-linux with 1-2 dataset
Memory used (MiB)
Time (seconds)
0
50
100
150
200
250
300
0.00
0.25
0.50
0.75
1.00
1.25
1.50
1.75
2.00

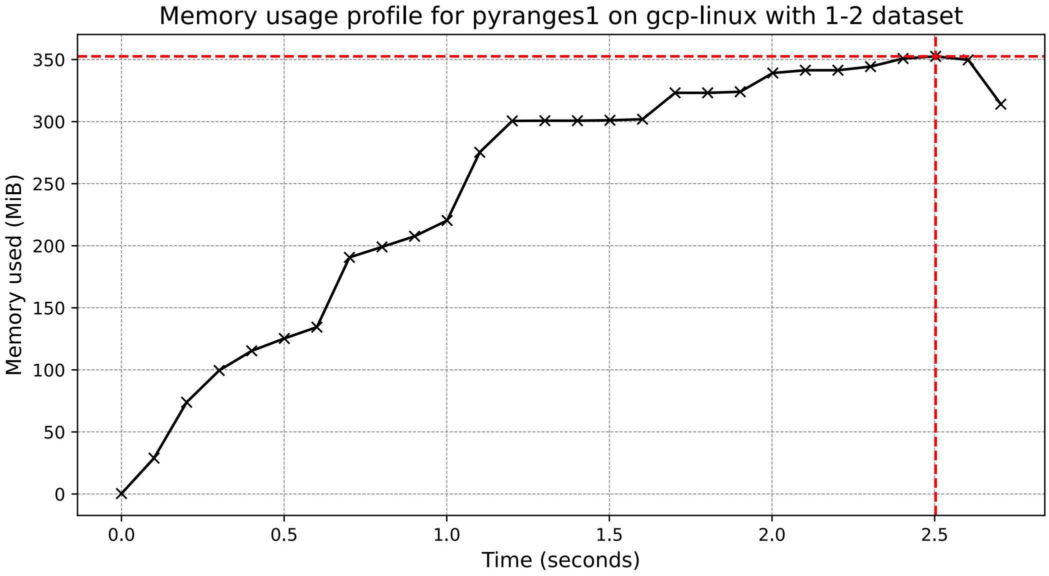
Memory usage profile for pyranges1 on gcp-linux with 1-2 dataset
Memory used (MiB)
Time (seconds)
0
50
100
150
200
250
300
350
0.0
0.5
1.0
1.5
2.0
2.5

### 3.4.20 Operation: count-overlaps for dataset: fBrain-vs-exons on platform: gcp-linux

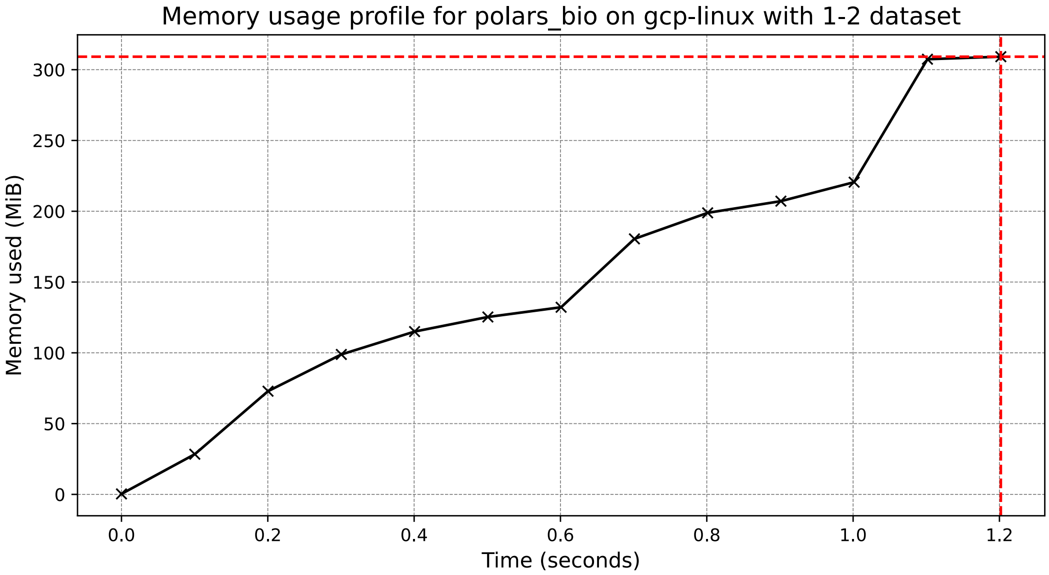


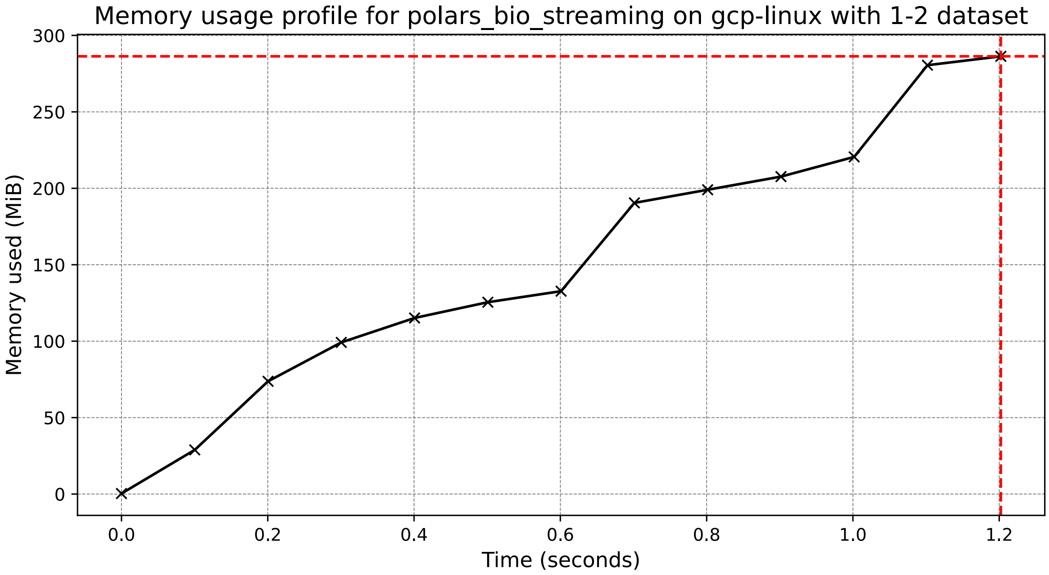

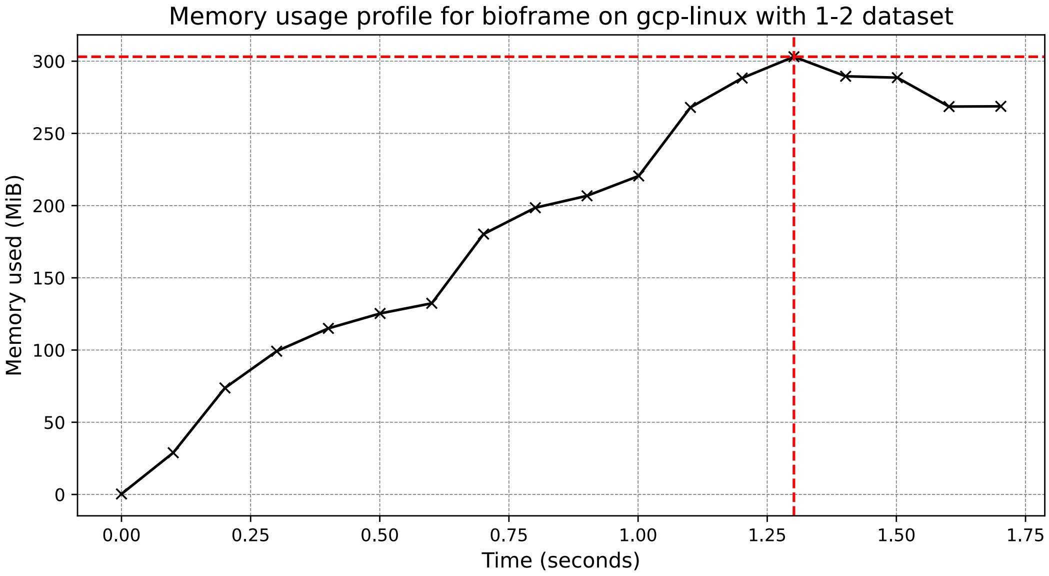

Memory usage profile for bioframe on gcp-linux with 1-2 dataset
Memory used (MiB)
Time (seconds)
0
50
100
150
200
250
300
0.00
0.25
0.50
0.75
1.00
1.25
1.50
1.75


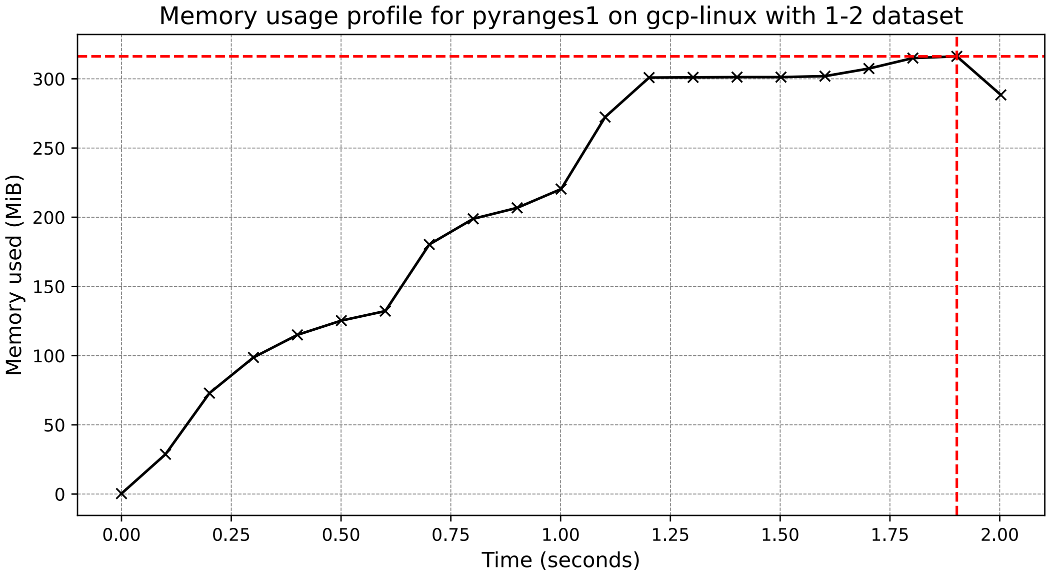

Memory usage profile for pyranges1 on gcp-linux with 1-2 dataset
Memory used (MiB)
Time (seconds)
0
50
100
150
200
250
300
0.00
0.25
0.50
0.75
1.00
1.25
1.50
1.75
2.00

### 3.4.21 Operation: overlap for dataset: ex-rna-vs-ex-anno on platform: gcp-linux

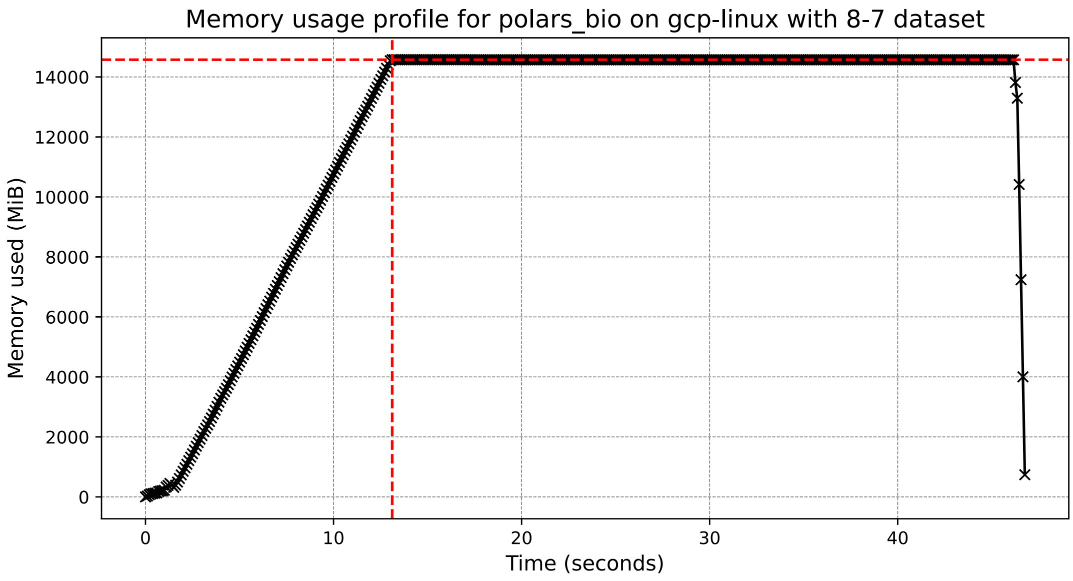


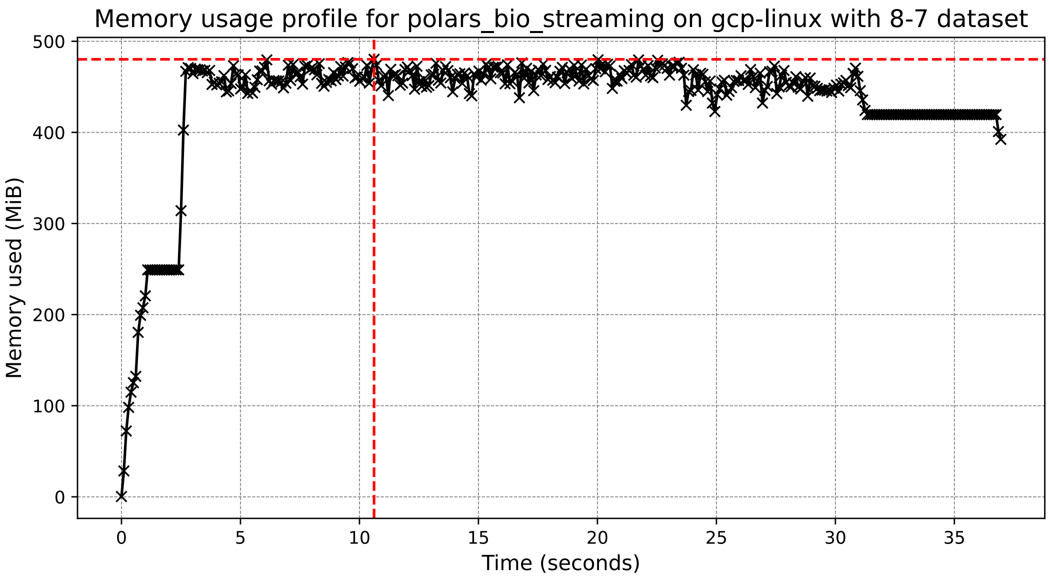

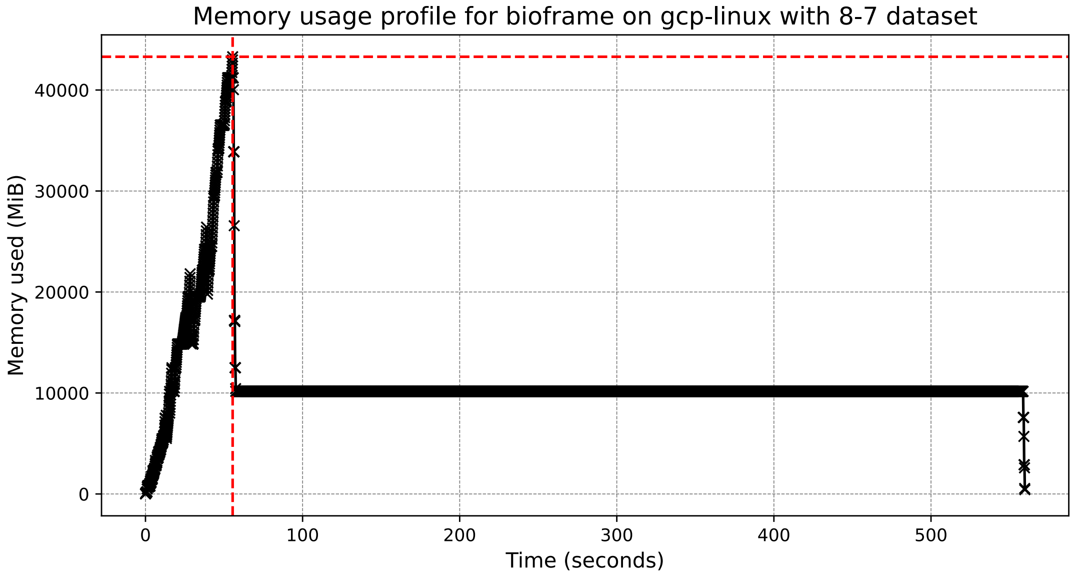
Memory usage profile for bioframe on gcp-linux with 8-7 dataset
Memory used (MiB)
40000
30000
20000
10000
0
0
100
200
300
400
500
Time (seconds)

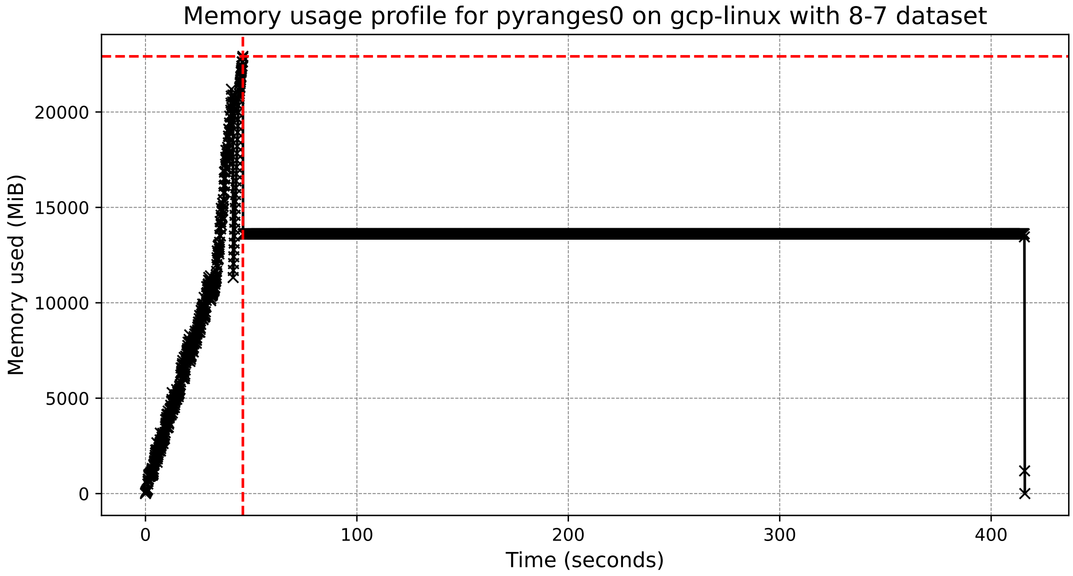
Memory usage profile for pyranges0 on gcp-linux with 8-7 dataset
Memory used (MiB)
20000
15000
10000
5000
0
0
100
200
300
400
Time (seconds)

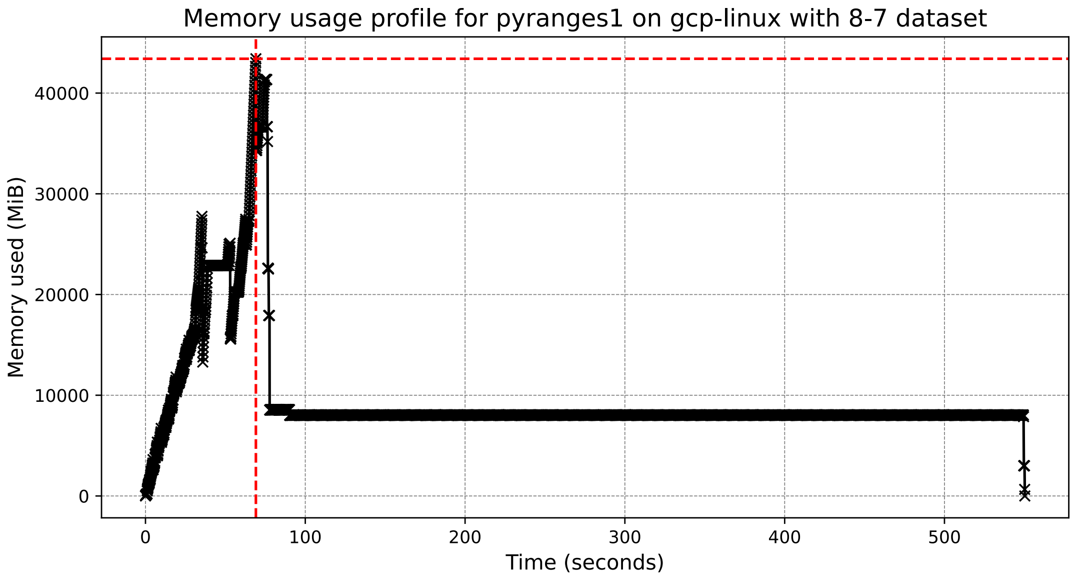


### 3.4.22 Operation: nearest for dataset: ex-rna-vs-ex-anno on platform: gcp-linux

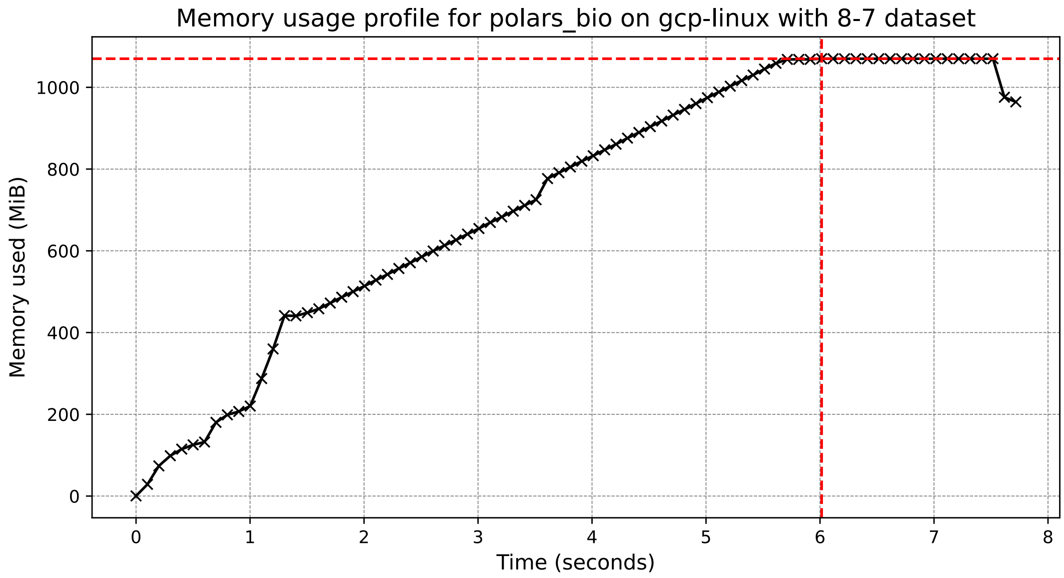

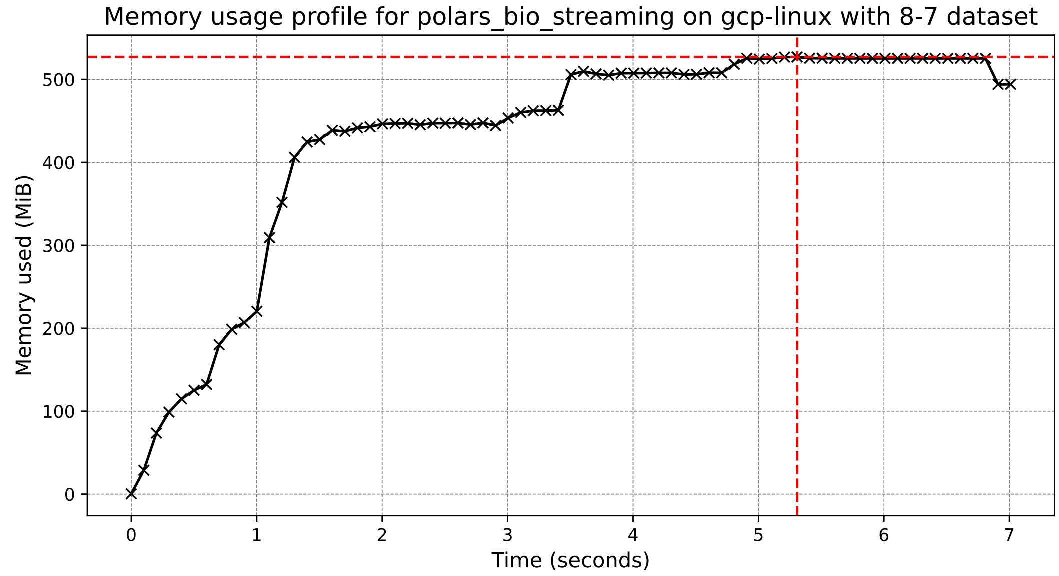

Memory usage profile for polars_bio_streaming on gcp-linux with 8-7 dataset
Memory used (MiB)
0
100
200
300
400
500
0
1
2
3
4
5
6
7
Time (seconds)


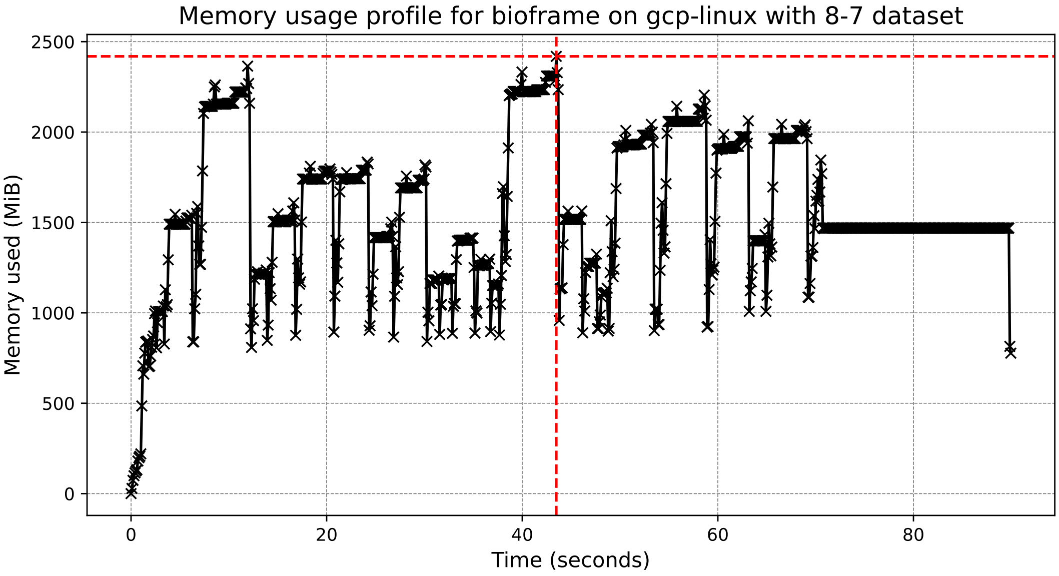

Memory usage profile for bioframe on gcp-linux with 8-7 dataset
Memory used (MiB)
0
500
1000
1500
2000
2500
0
20
40
60
80
Time (seconds)

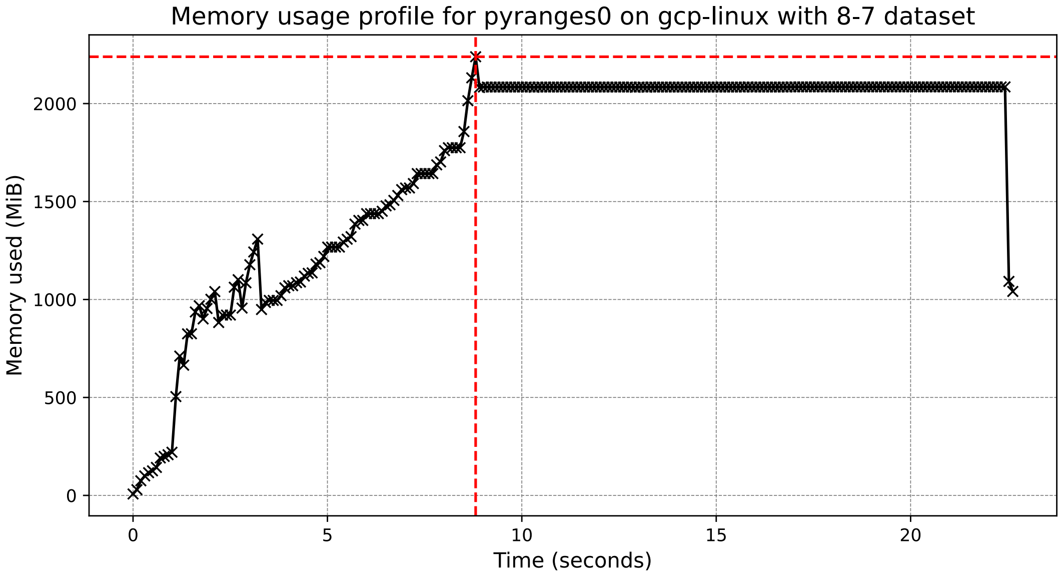
Memory usage profile for pyranges0 on gcp-linux with 8-7 dataset
Memory used (MiB)
Time (seconds)
0
500
1000
1500
2000
0
5
10
15
20

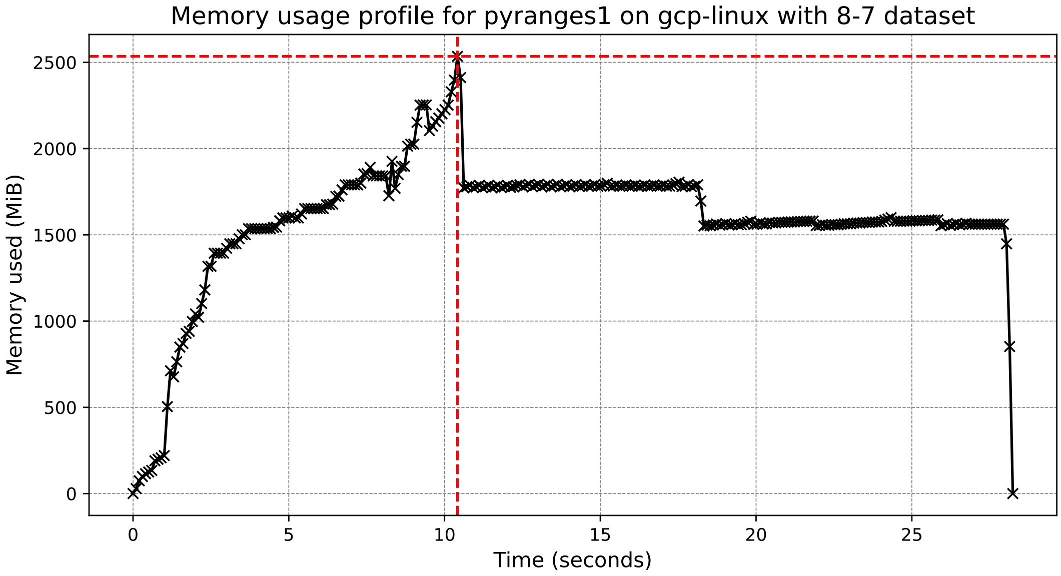
Memory usage profile for pyranges1 on gcp-linux with 8-7 dataset
Memory used (MiB)
Time (seconds)
0
500
1000
1500
2000
2500
0
5
10
15
20
25

### 3.4.23 Operation: coverage for dataset: ex-rna-vs-ex-anno on platform: gcp-linux

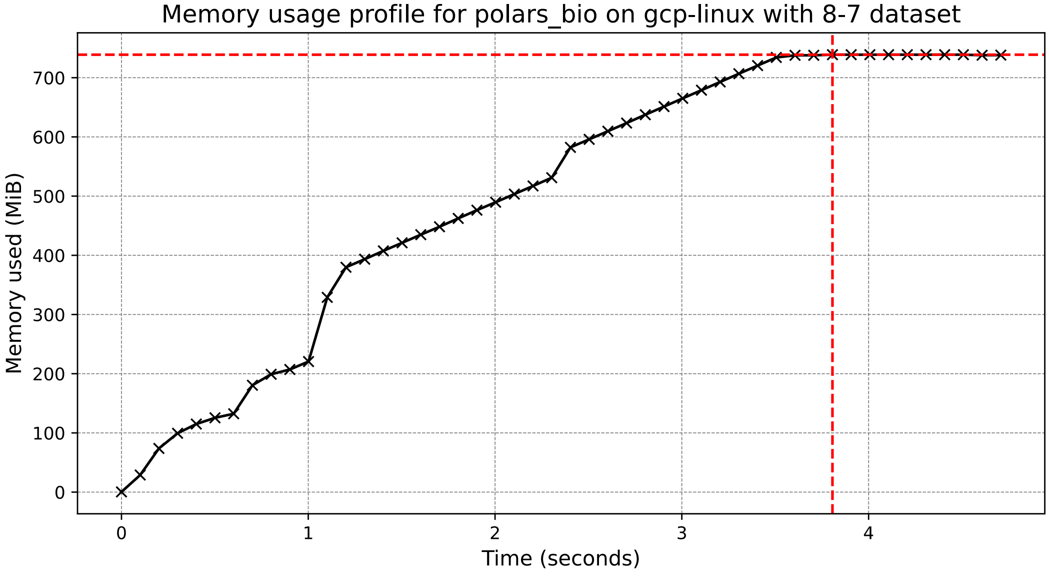


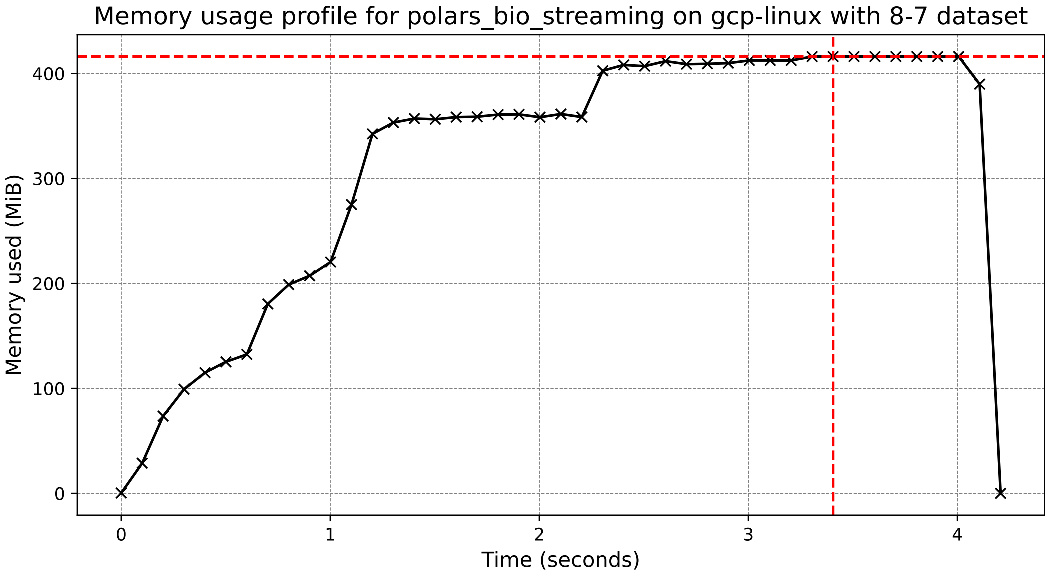

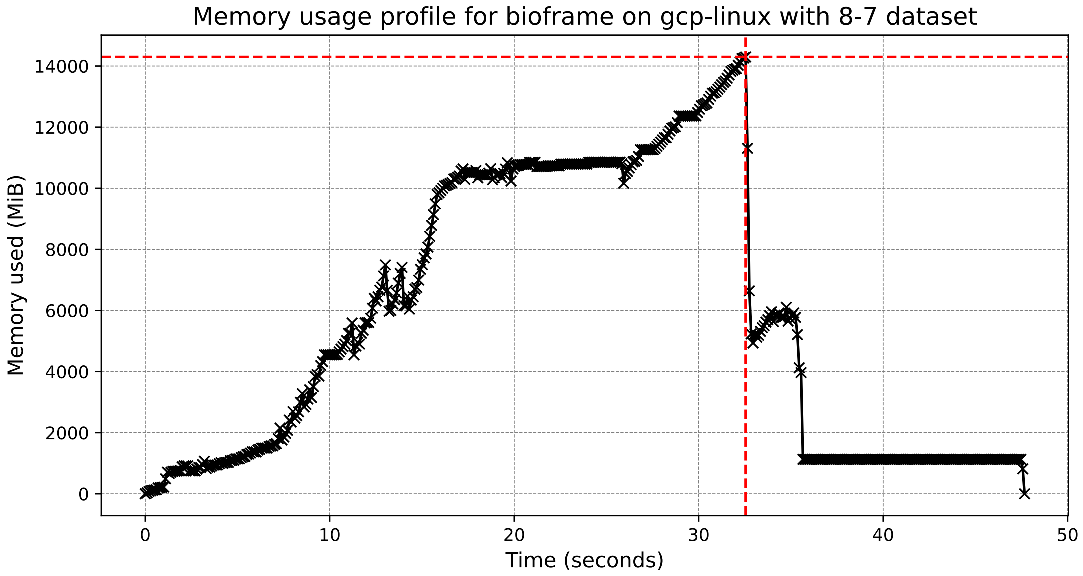

Memory usage profile for bioframe on gcp-linux with 8-7 dataset
Memory used (MiB)
Time (seconds)
0
2000
4000
6000
8000
10000
12000
14000
0
10
20
30
40
50


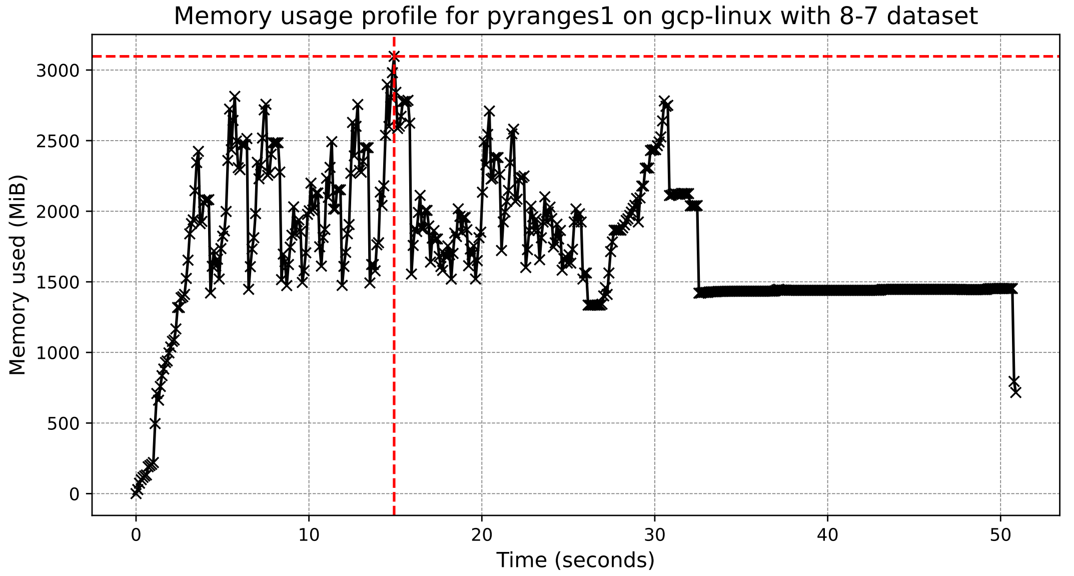

Memory usage profile for pyranges1 on gcp-linux with 8-7 dataset
Memory used (MiB)
Time (seconds)
0
500
1000
1500
2000
2500
3000
0
10
20
30
40
50

### 3.4.24 Operation: count-overlaps for dataset: ex-rna-vs-ex-anno on platform: gcp-linux

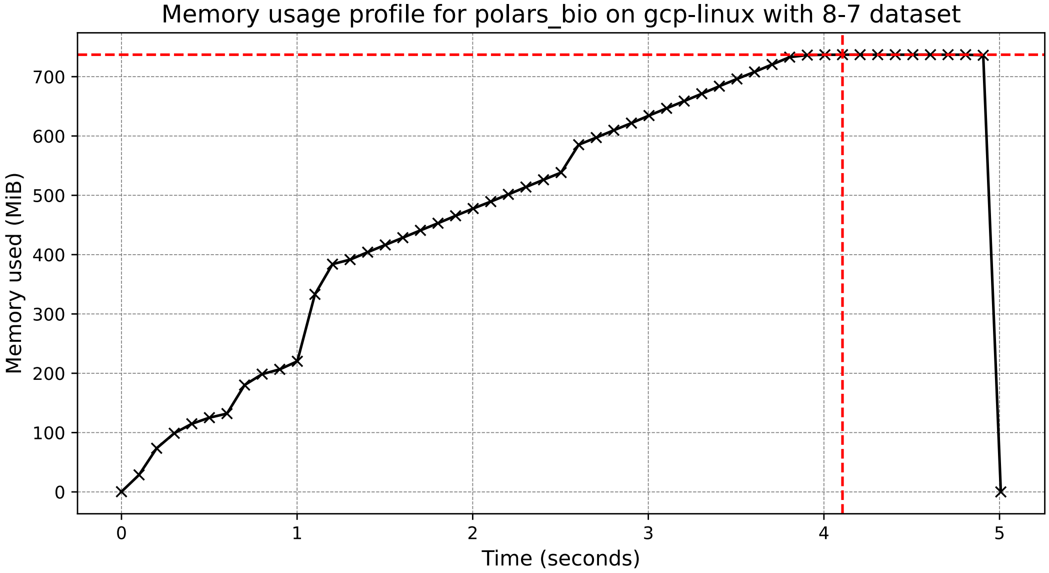


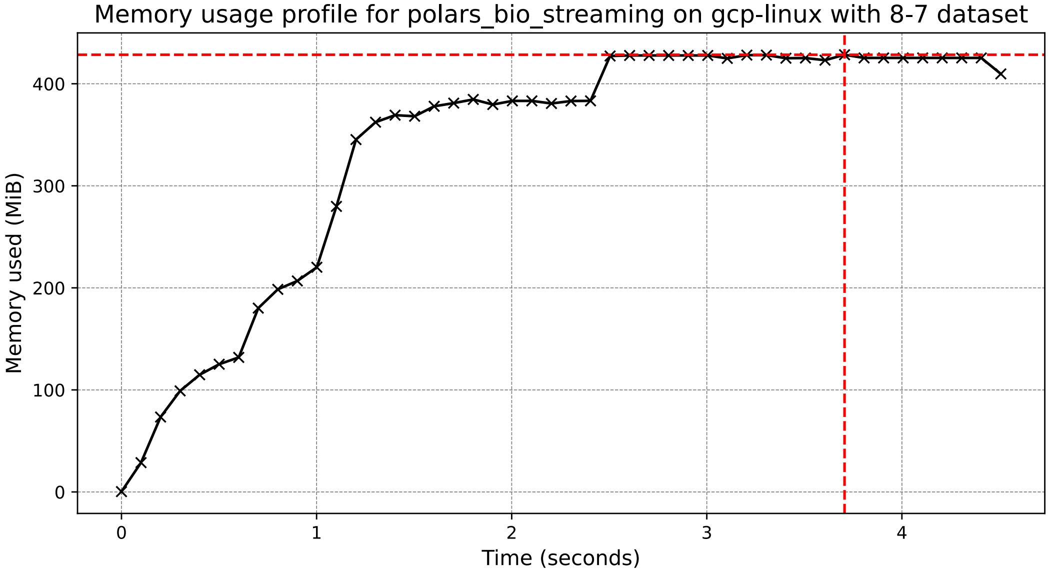

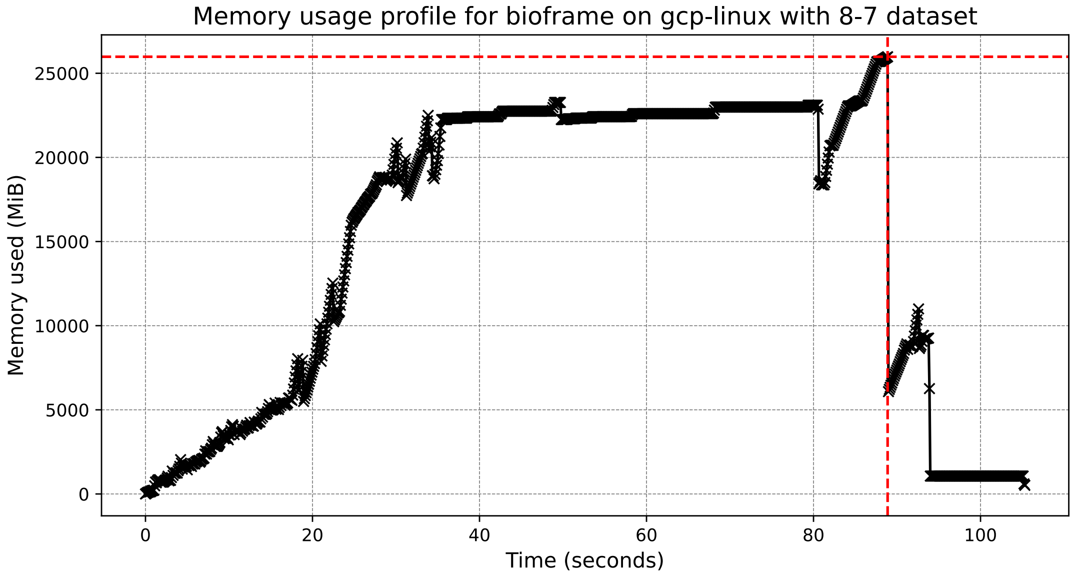

Memory usage profile for bioframe on gcp-linux with 8-7 dataset
Memory used (MiB)
Time (seconds)
0
5000
10000
15000
20000
25000
0
20
40
60
80
100


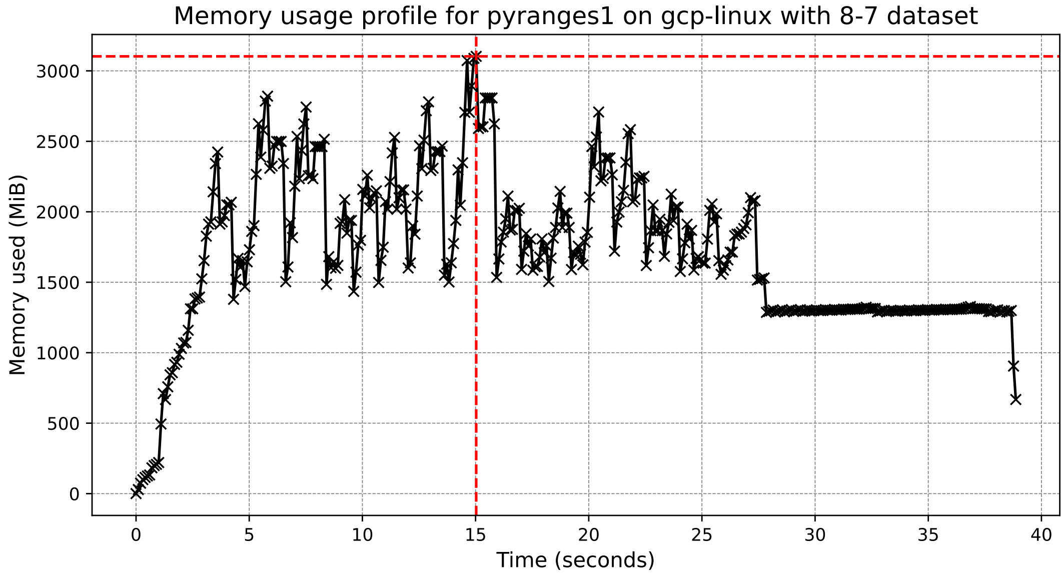

Memory usage profile for pyranges1 on gcp-linux with 8-7 dataset
Memory used (MiB)
Time (seconds)
0
500
1000
1500
2000
2500
3000
0
5
10
15
20
25
30
35
40

#### 3.4.25 Operation: overlap for dataset: 100-1p on platform: gcp-linux

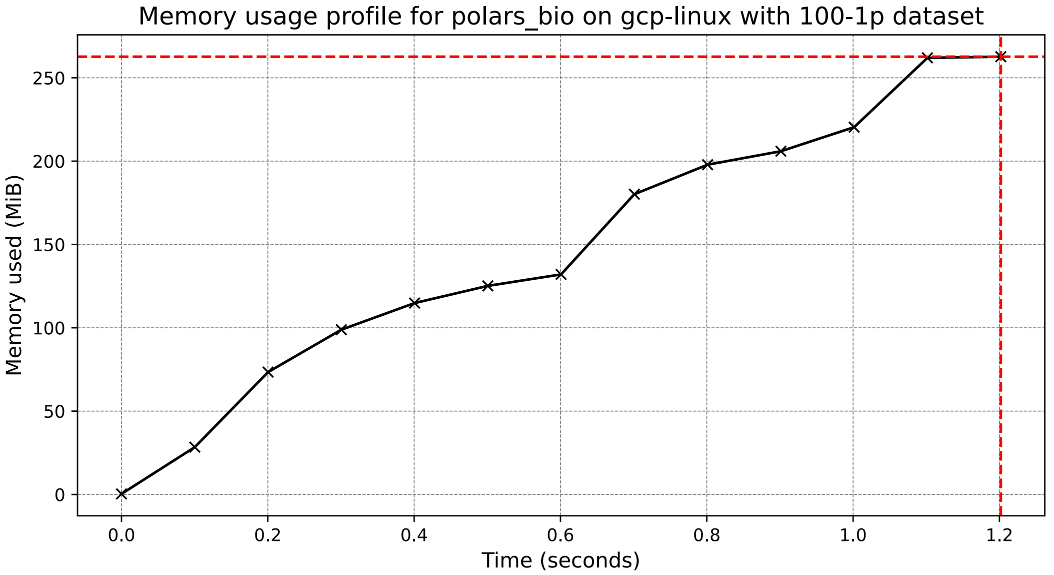


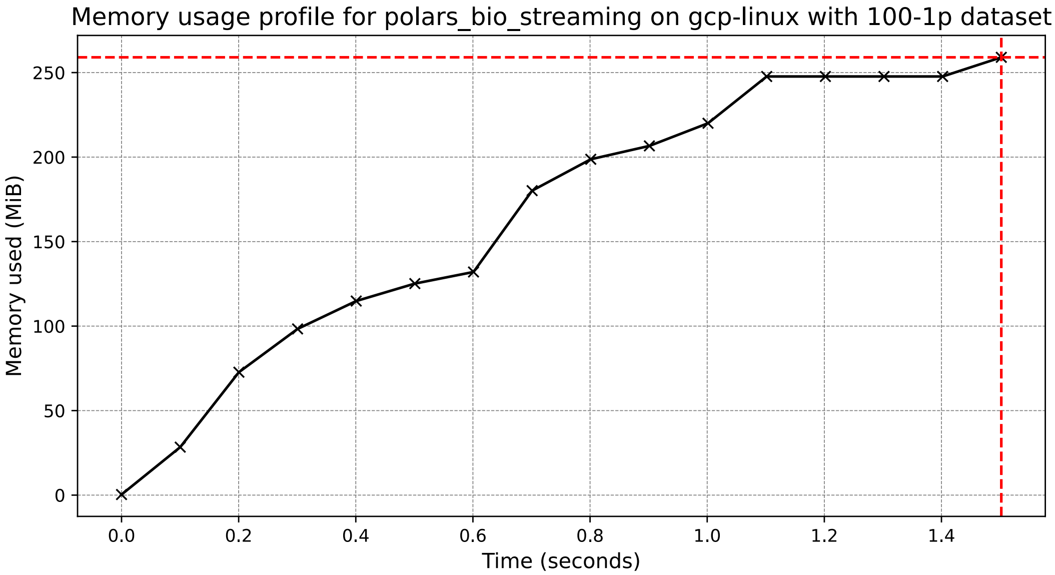

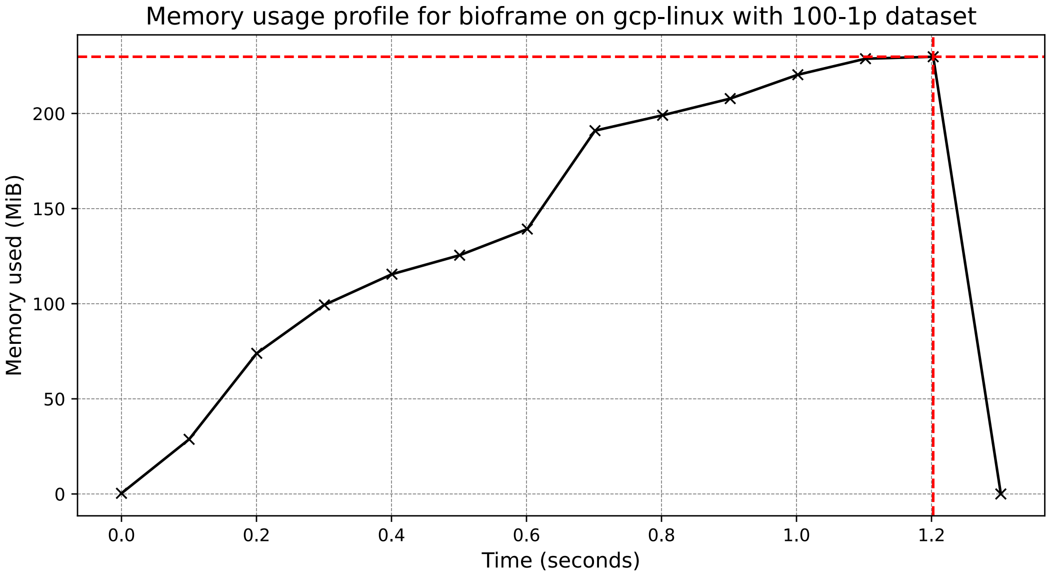
Memory usage profile for bioframe on gcp-linux with 100-1p dataset
Memory used (MiB)
Time (seconds)
0
50
100
150
200
0.0
0.2
0.4
0.6
0.8
1.0
1.2

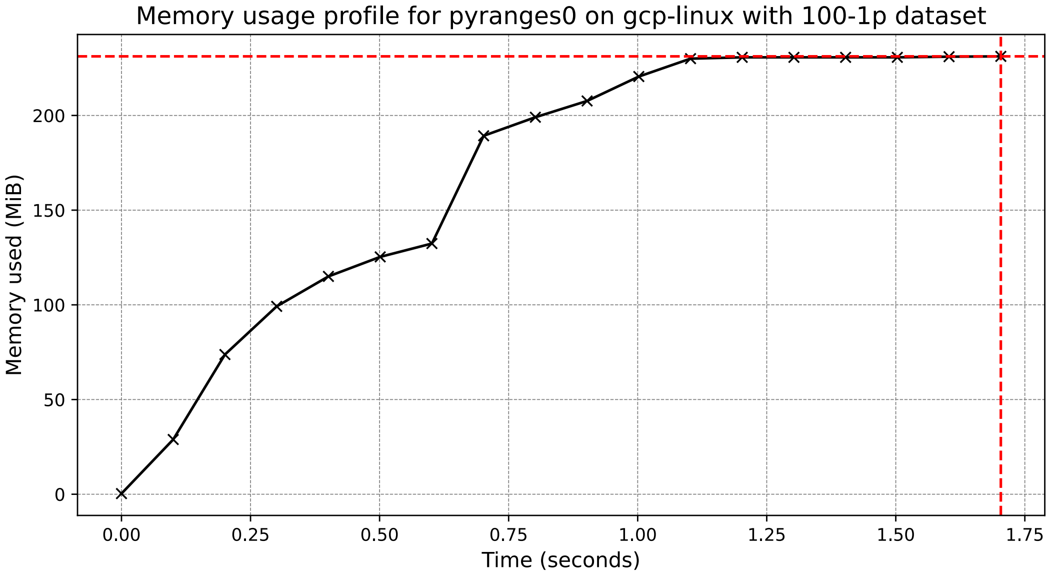
Memory usage profile for pyranges0 on gcp-linux with 100-1p dataset
Memory used (MiB)
Time (seconds)
0
50
100
150
200
0.00
0.25
0.50
0.75
1.00
1.25
1.50
1.75

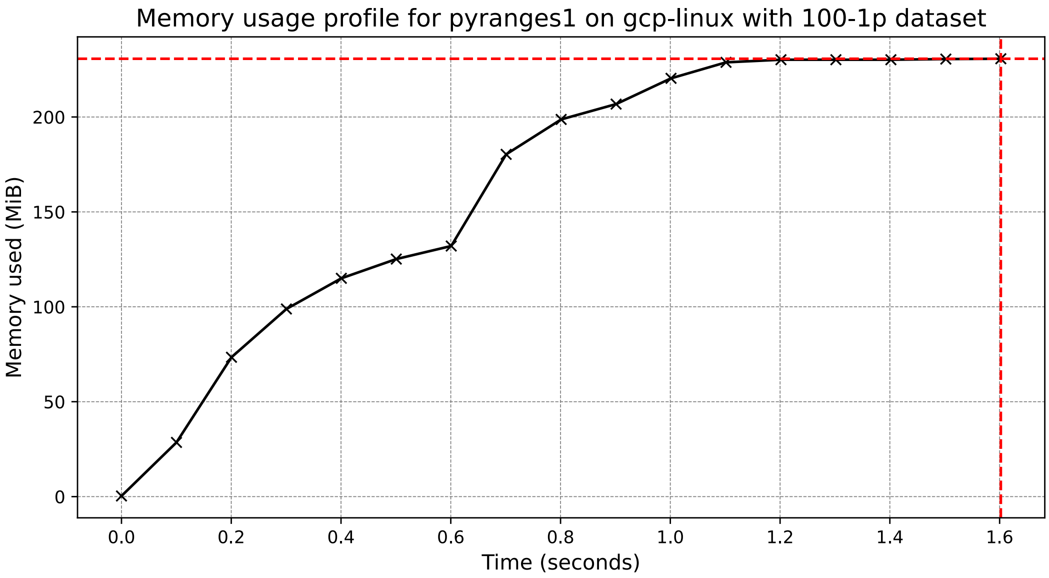


### 3.4.26 Operation: nearest for dataset: 100-1p on platform: gcp-linux

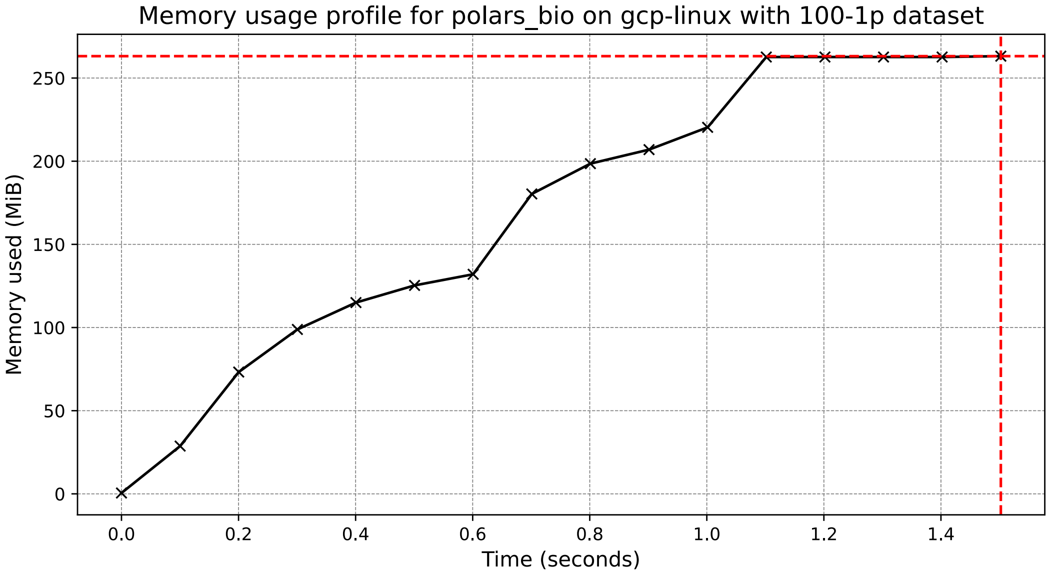

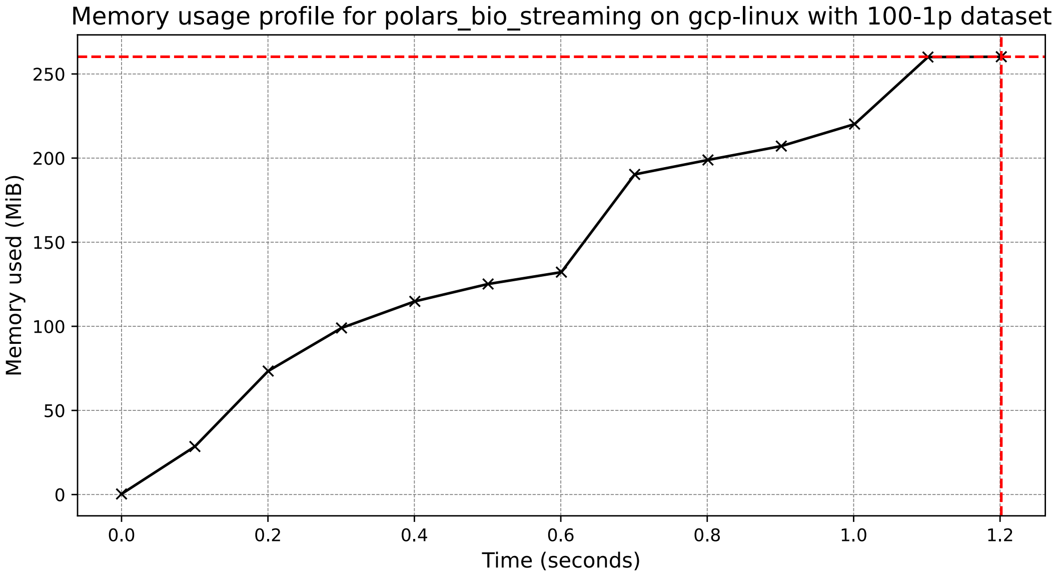
Memory usage profile for polars_bio_streaming on gcp-linux with 100-1p dataset
Memory used (MiB)
Time (seconds)
0
50
100
150
200
250
0.0
0.2
0.4
0.6
0.8
1.0
1.2

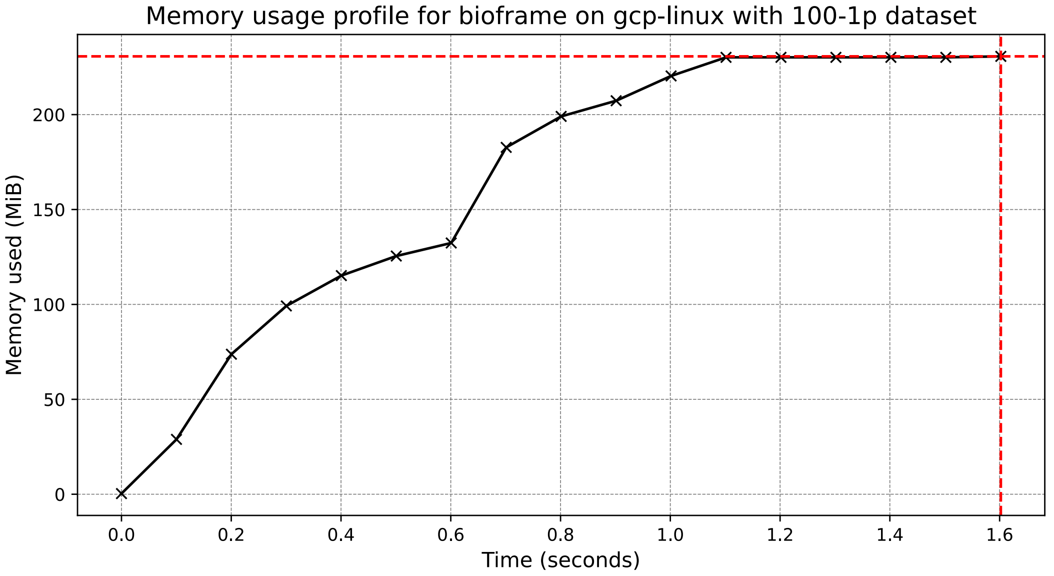
Memory usage profile for bioframe on gcp-linux with 100-1p dataset
Memory used (MiB)
Time (seconds)
0
50
100
150
200
0.0
0.2
0.4
0.6
0.8
1.0
1.2
1.4
1.6

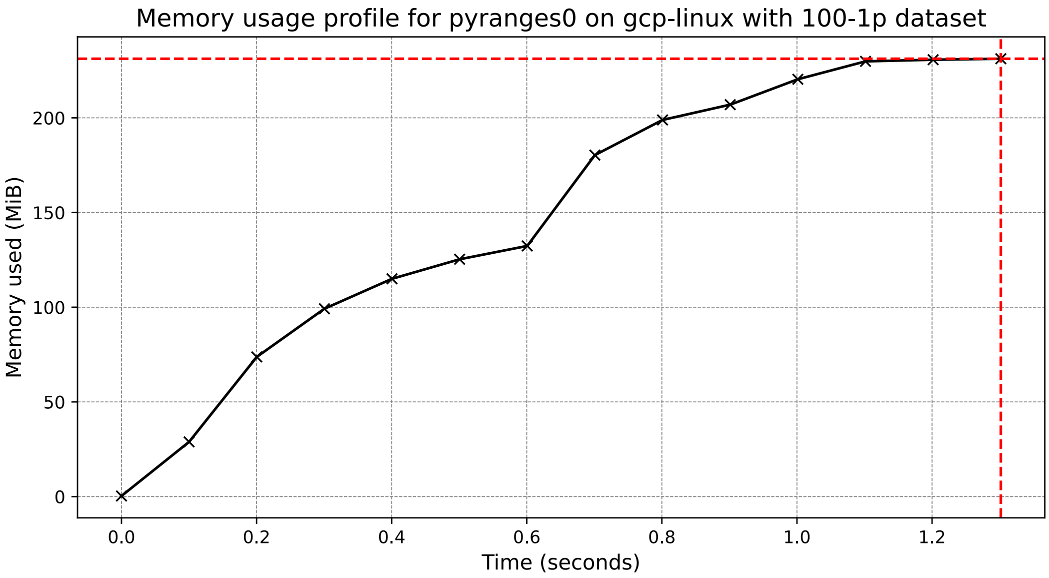
Memory usage profile for pyranges0 on gcp-linux with 100-1p dataset
Memory used (MiB)
Time (seconds)
0
50
100
150
200
0.0
0.2
0.4
0.6
0.8
1.0
1.2

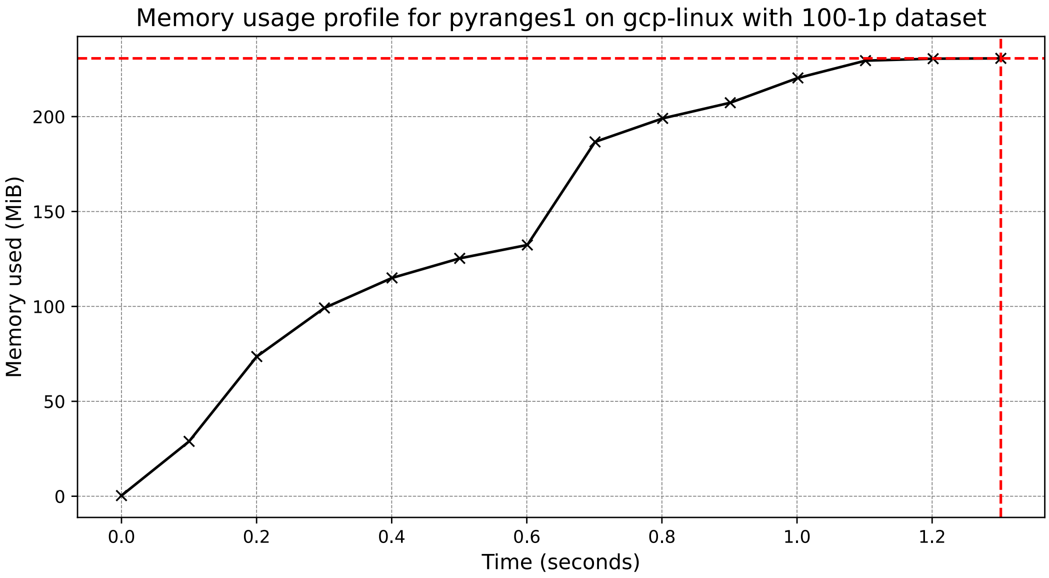
Memory usage profile for pyranges1 on gcp-linux with 100-1p dataset
Memory used (MiB)
Time (seconds)
0
50
100
150
200
0.0
0.2
0.4
0.6
0.8
1.0
1.2

### 3.4.27 Operation: coverage for dataset: 100-1p on platform: gcp-linux

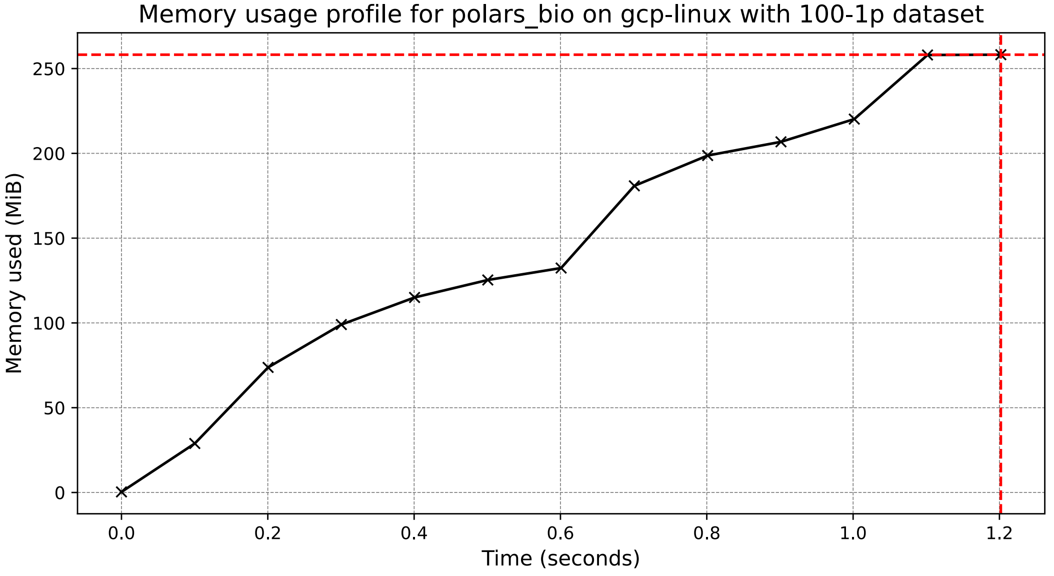


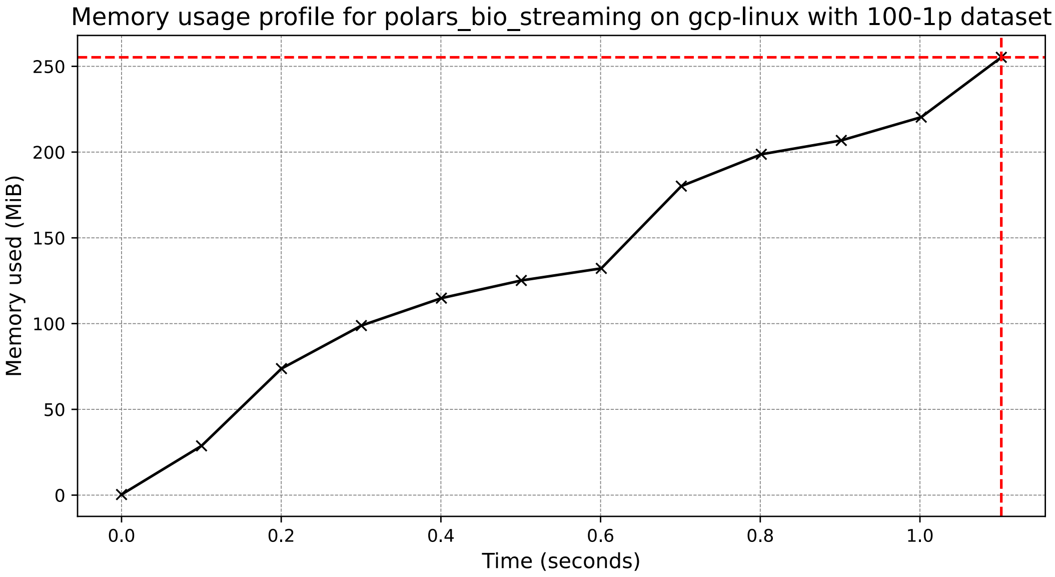

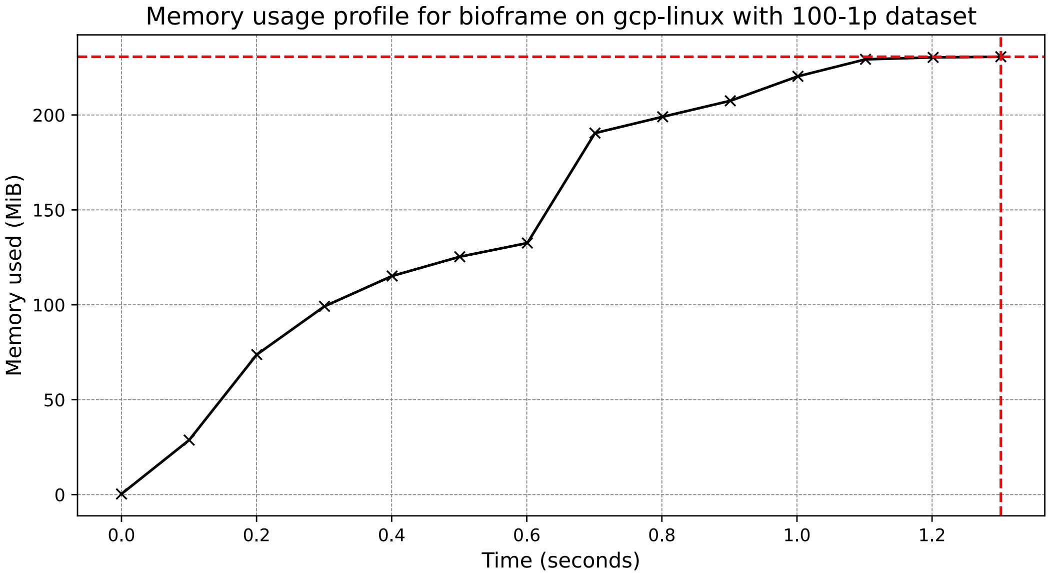
Memory usage profile for bioframe on gcp-linux with 100-1p dataset
Memory used (MiB)
Time (seconds)
0
50
100
150
200
0.0
0.2
0.4
0.6
0.8
1.0
1.2

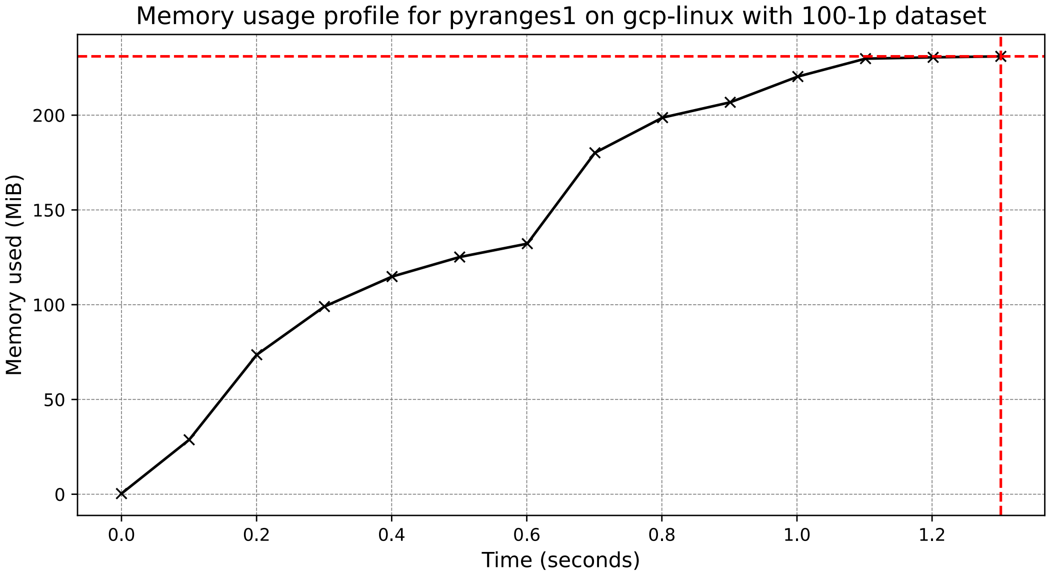
Memory usage profile for pyranges1 on gcp-linux with 100-1p dataset
Memory used (MiB)
Time (seconds)
0
50
100
150
200
0.0
0.2
0.4
0.6
0.8
1.0
1.2

### 3.4.28 Operation: count-overlaps for dataset: 100-1p on platform: gcp-linux

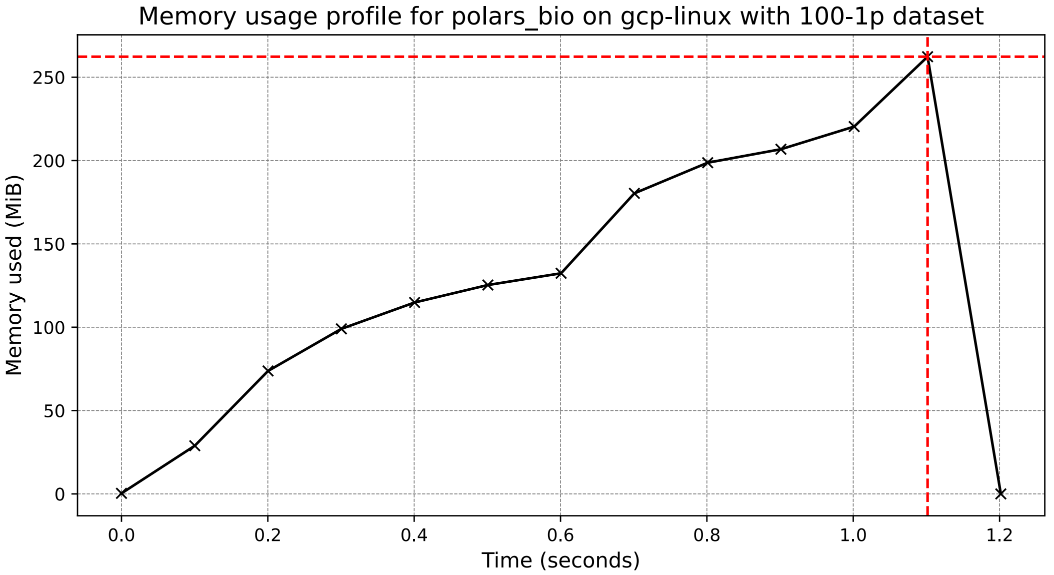


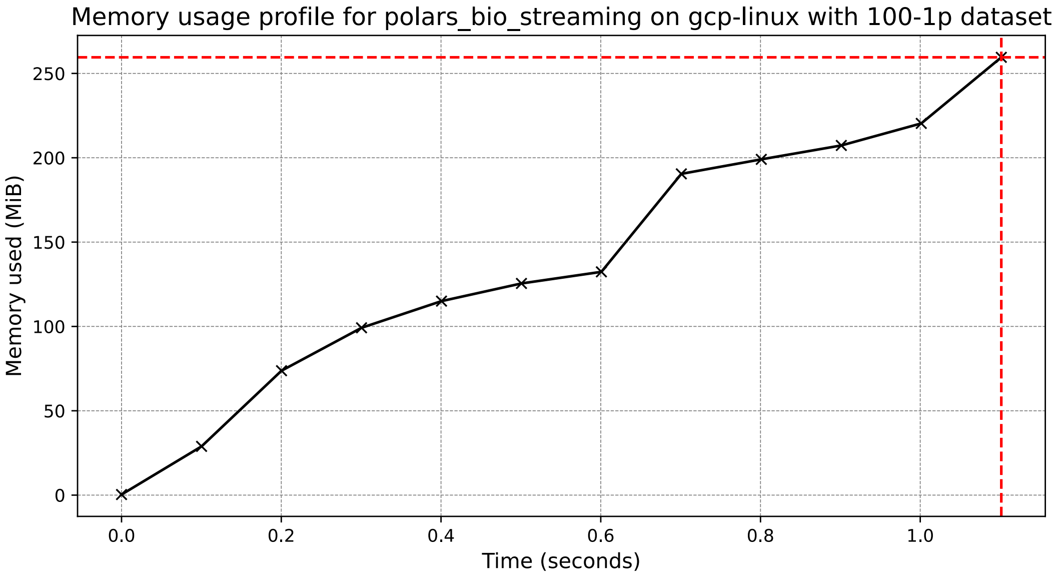

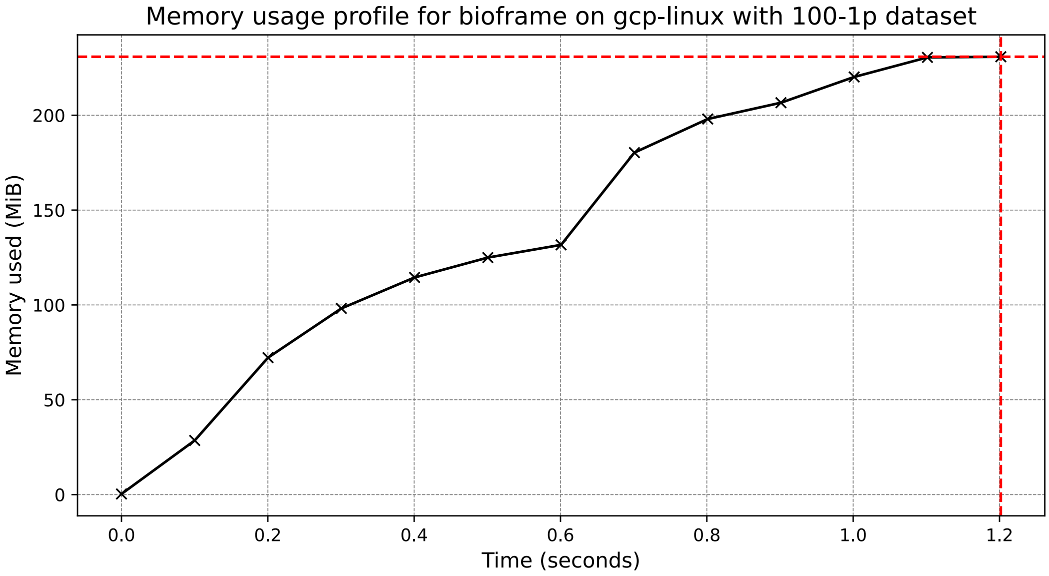
Memory usage profile for bioframe on gcp-linux with 100-1p dataset
Memory used (MiB)
Time (seconds)
0
50
100
150
200
0.0
0.2
0.4
0.6
0.8
1.0
1.2

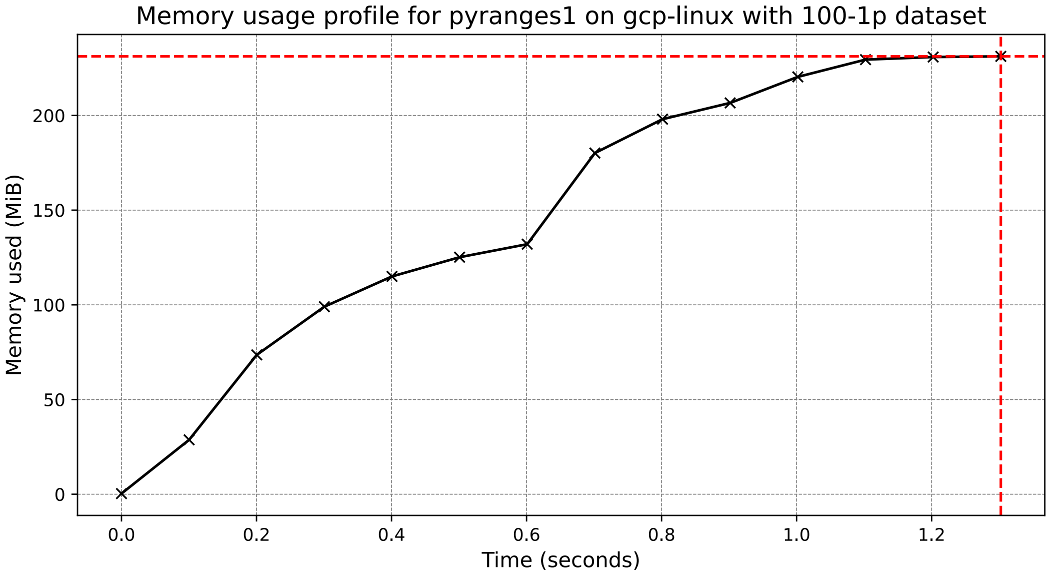
Memory usage profile for pyranges1 on gcp-linux with 100-1p dataset
Memory used (MiB)
Time (seconds)
0
50
100
150
200
0.0
0.2
0.4
0.6
0.8
1.0
1.2

### 3.4.29 Operation: overlap for dataset: 10000000-1p on platform: gcp-linux

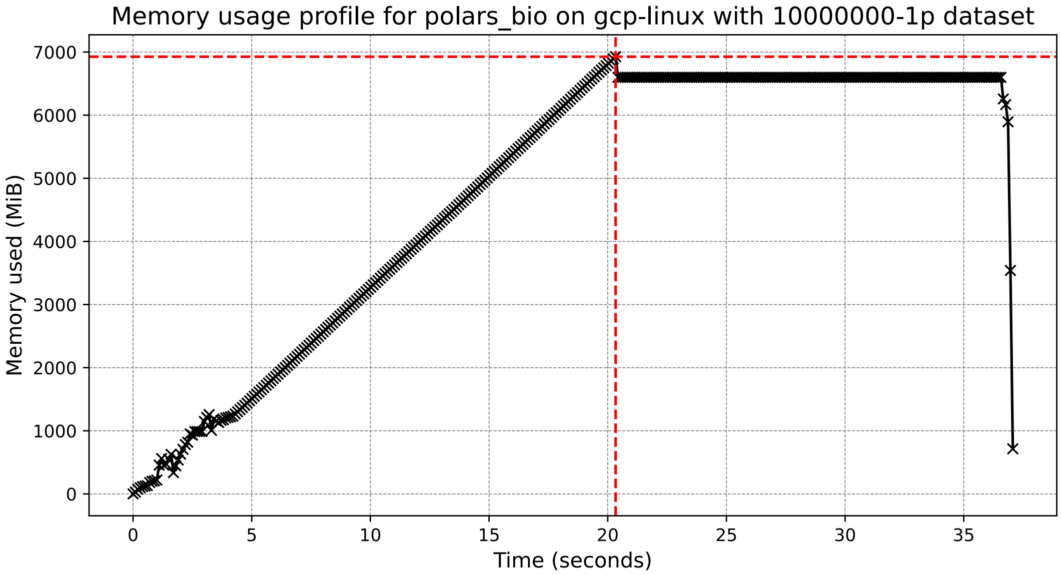


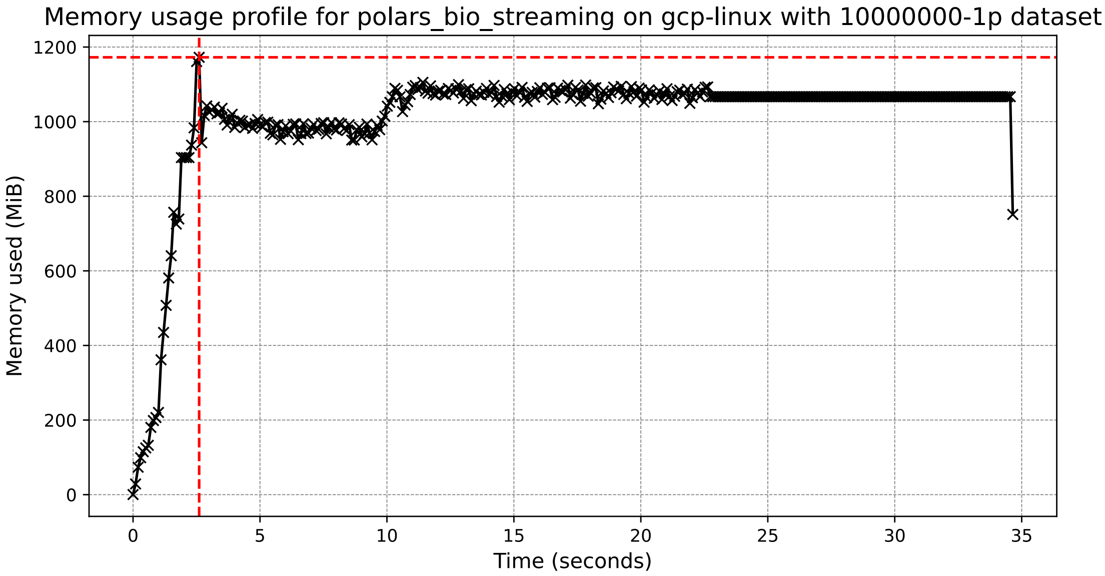

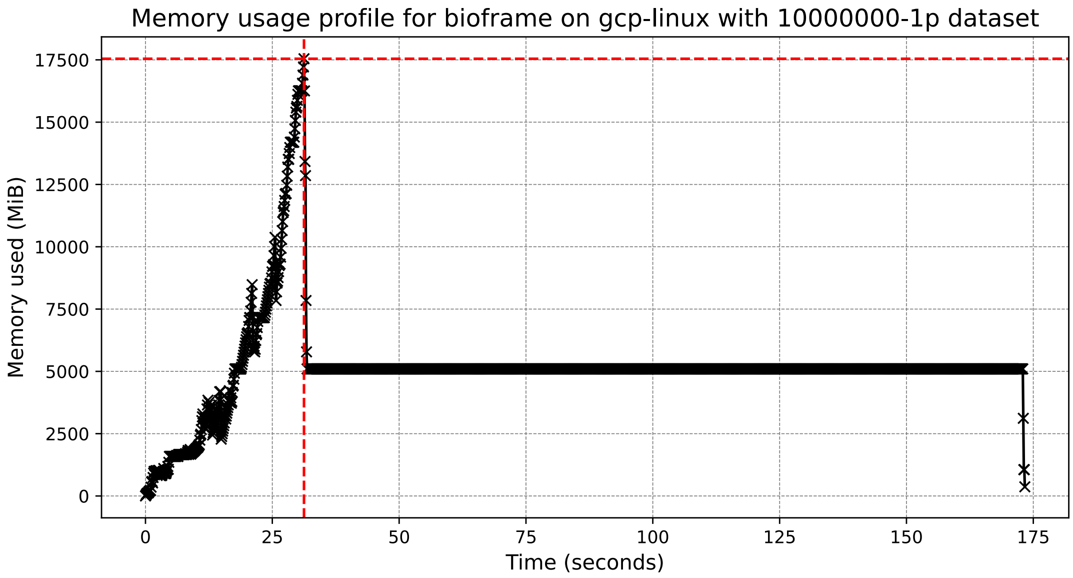
Memory usage profile for bioframe on gcp-linux with 10000000-1p dataset
Memory used (MiB)
Time (seconds)
0
2500
5000
7500
10000
12500
15000
17500
0
25
50
75
100
125
150
175

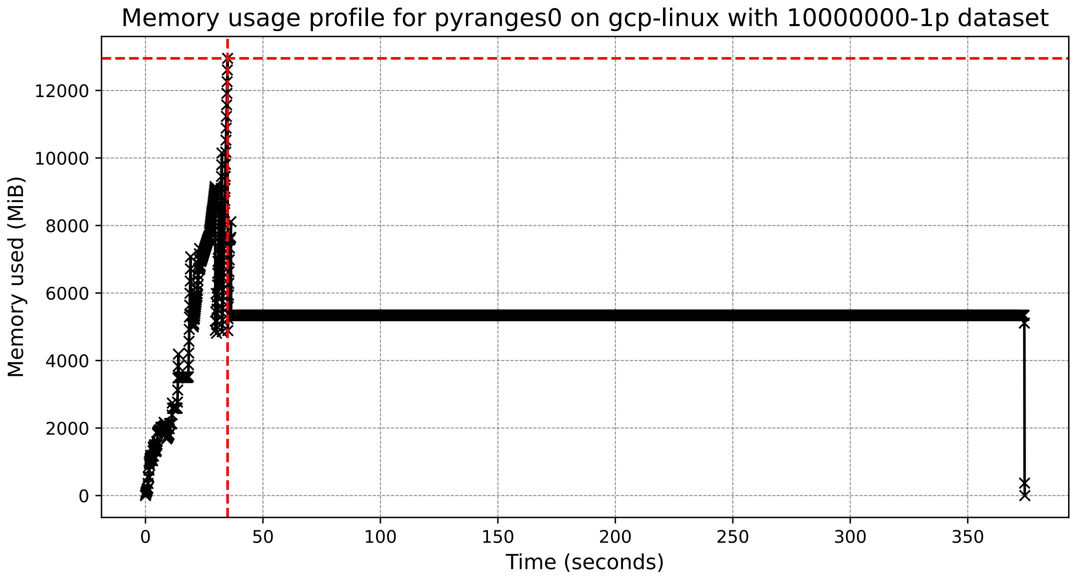
Memory usage profile for pyranges0 on gcp-linux with 10000000-1p dataset
Memory used (MiB)
Time (seconds)
0
2000
4000
6000
8000
10000
12000
0
50
100
150
200
250
300
350

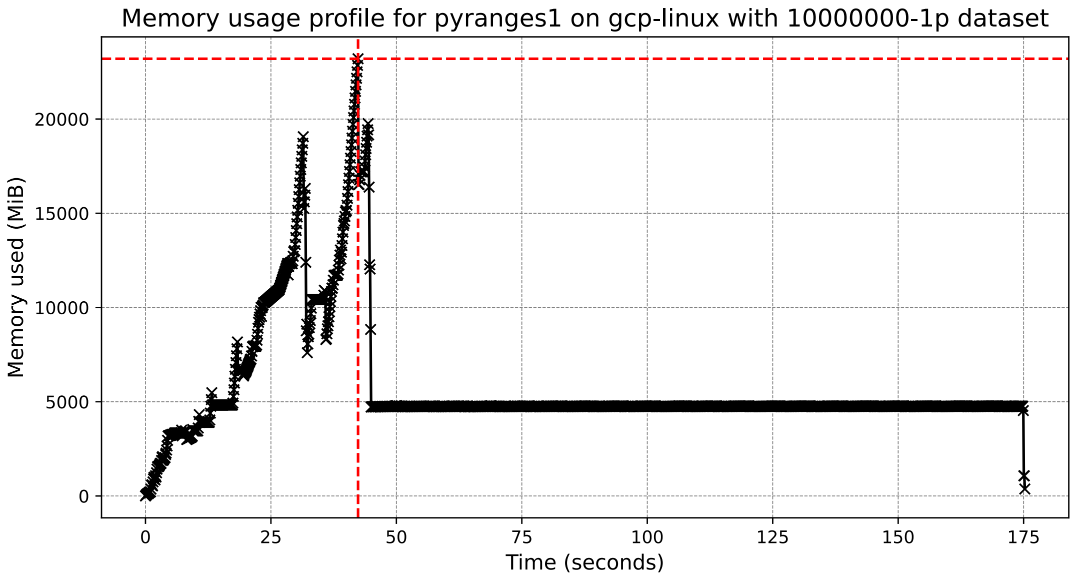


### 3.4.30 Operation: nearest for dataset: 10000000-1p on platform: gcp-linux

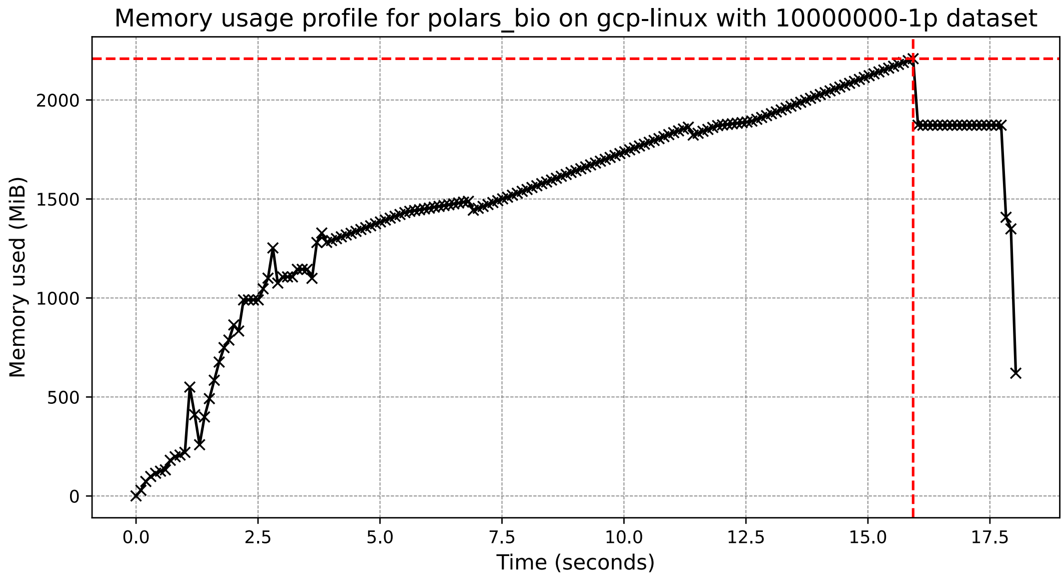

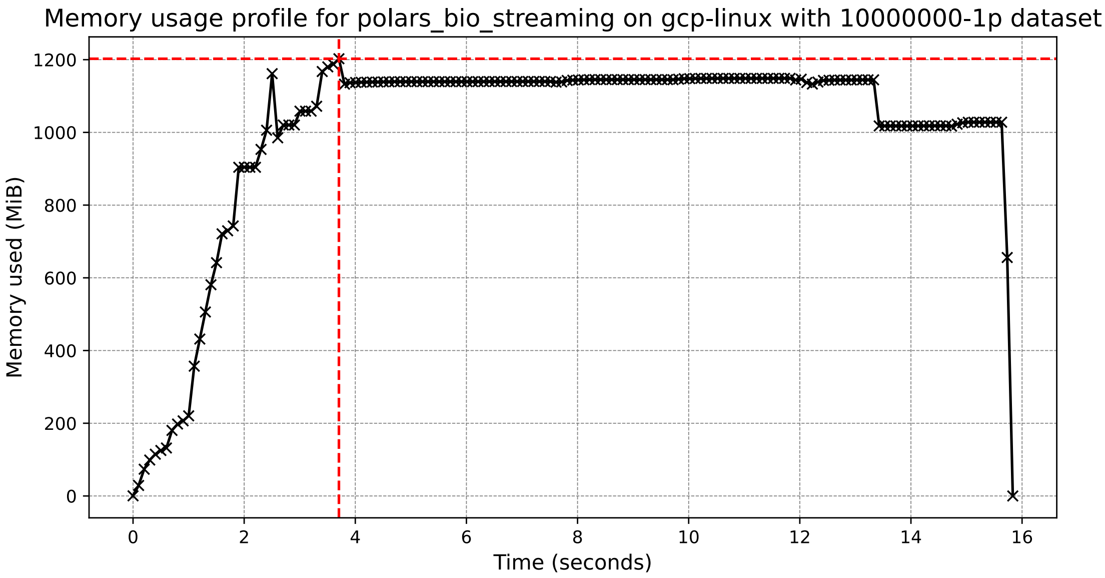
Memory usage profile for polars_bio_streaming on gcp-linux with 10000000-1p dataset
Memory used (MiB)
Time (seconds)

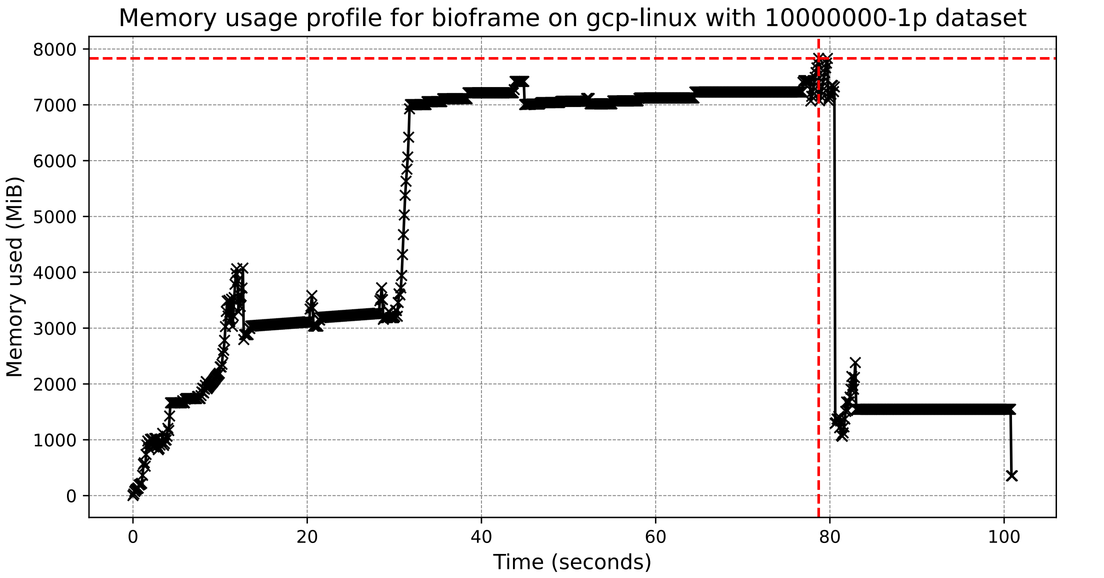
Memory usage profile for bioframe on gcp-linux with 10000000-1p dataset
Memory used (MiB)
Time (seconds)

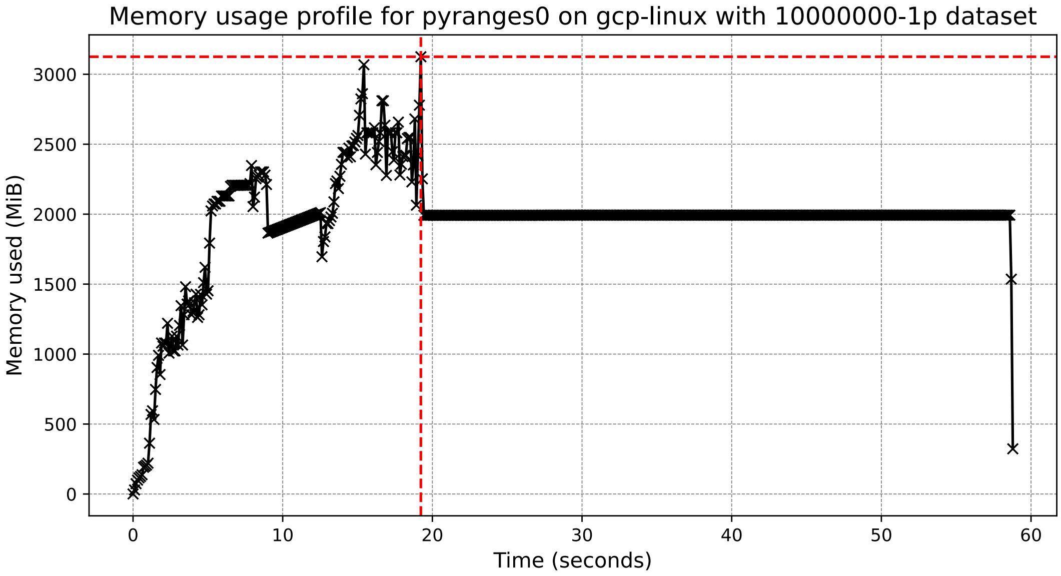
Memory usage profile for pyranges0 on gcp-linux with 10000000-1p dataset
Memory used (MiB)
Time (seconds)
0
500
1000
1500
2000
2500
3000
0
10
20
30
40
50
60

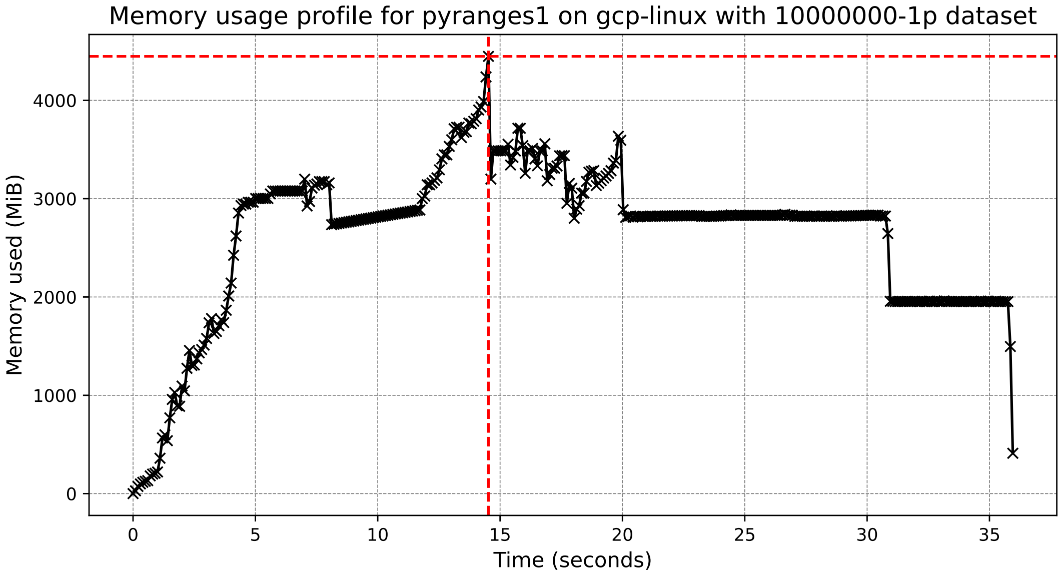
Memory usage profile for pyranges1 on gcp-linux with 10000000-1p dataset
Memory used (MiB)
Time (seconds)
0
1000
2000
3000
4000
0
5
10
15
20
25
30
35

### 3.4.31 Operation: coverage for dataset: 10000000-1p on platform: gcp-linux

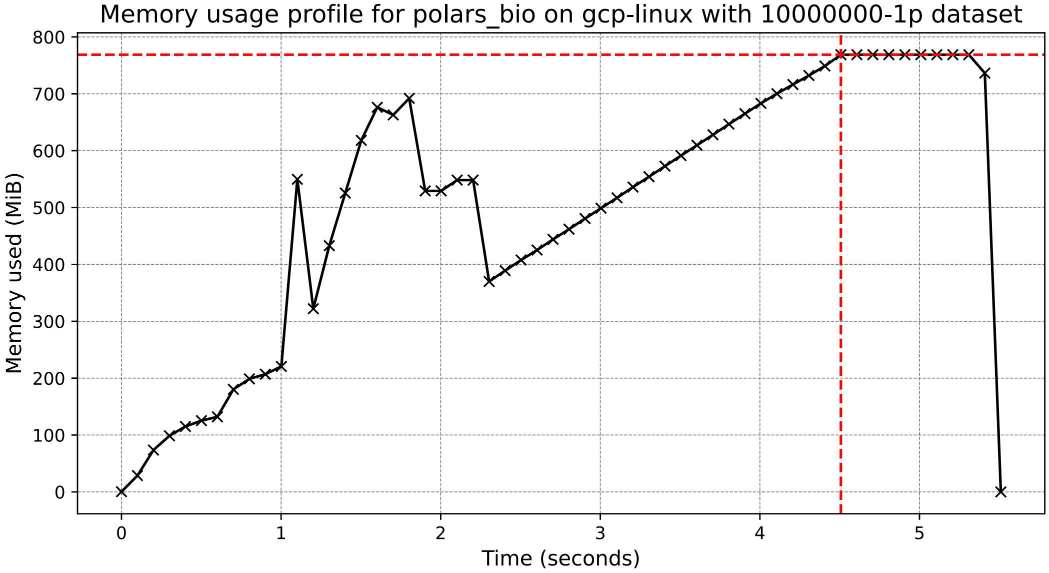


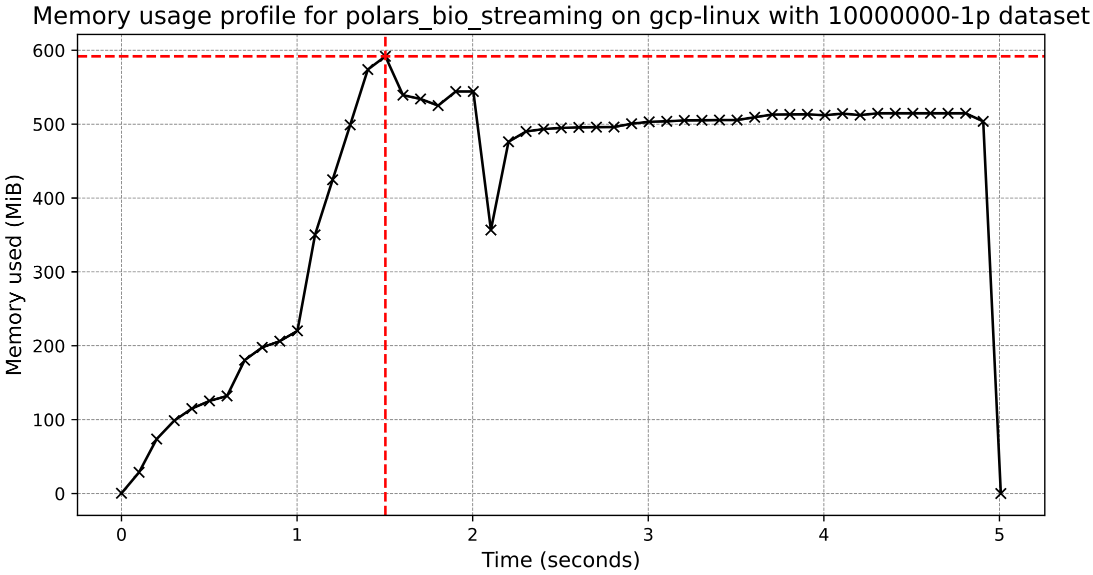

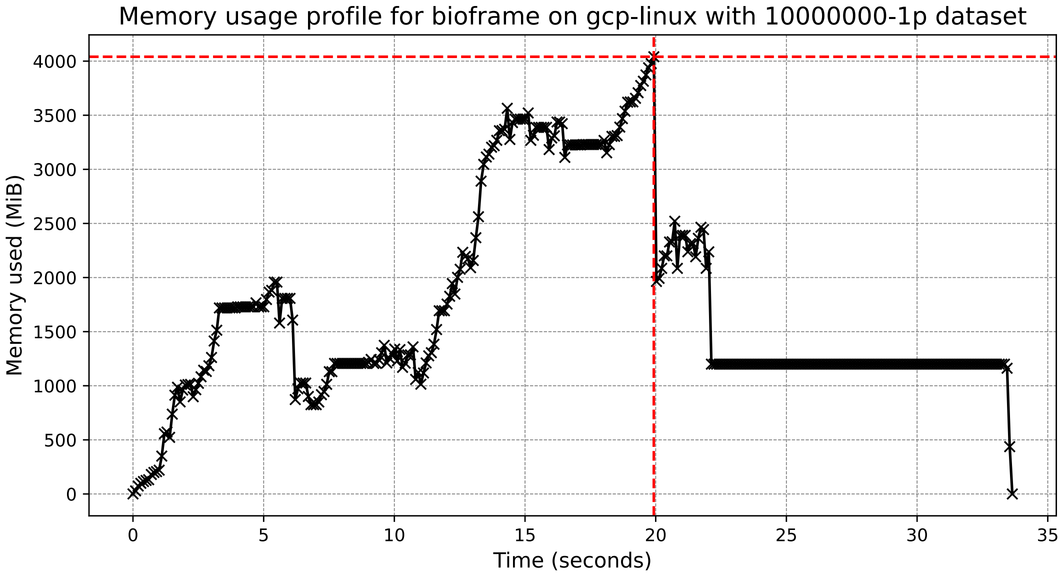

Memory usage profile for bioframe on gcp-linux with 10000000-1p dataset
Memory used (MiB)
Time (seconds)
0
500
1000
1500
2000
2500
3000
3500
4000
0
5
10
15
20
25
30
35


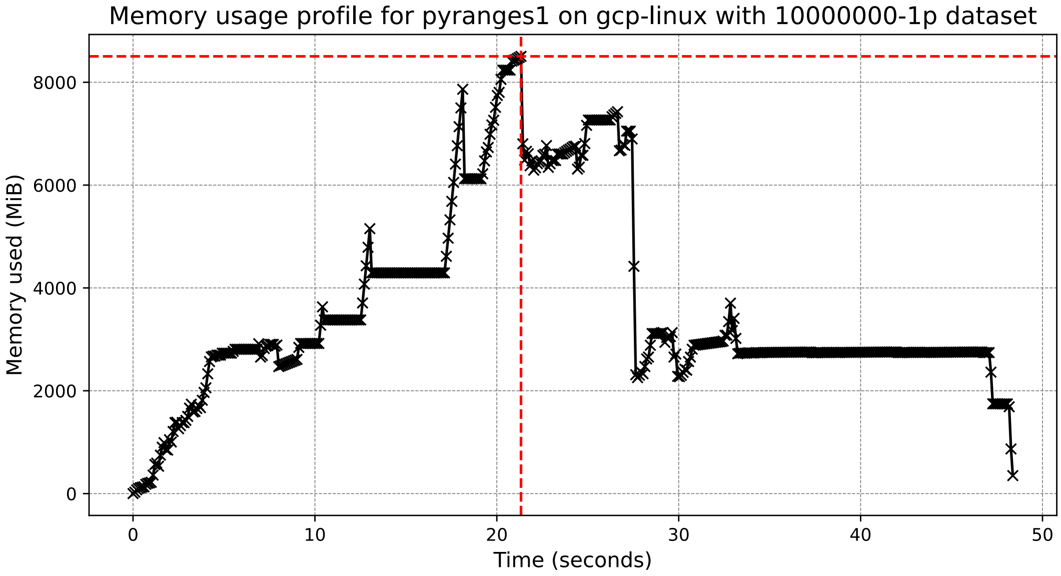

Memory usage profile for pyranges1 on gcp-linux with 10000000-1p dataset
Memory used (MiB)
Time (seconds)
0
2000
4000
6000
8000
0
10
20
30
40
50

### 3.4.32 Operation: count-overlaps for dataset: 10000000-1p on platform: gcp-linux

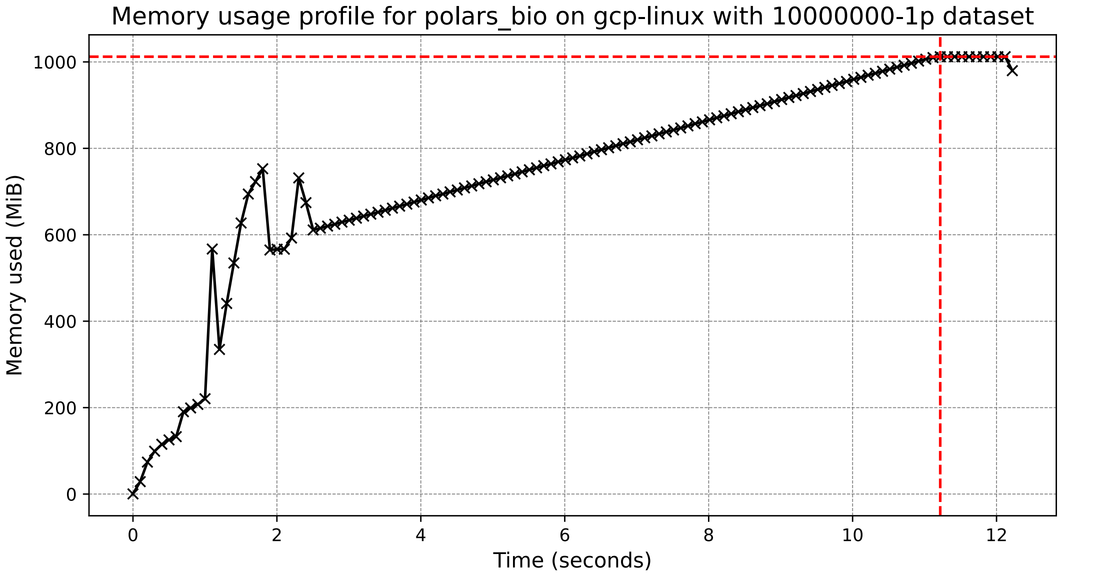


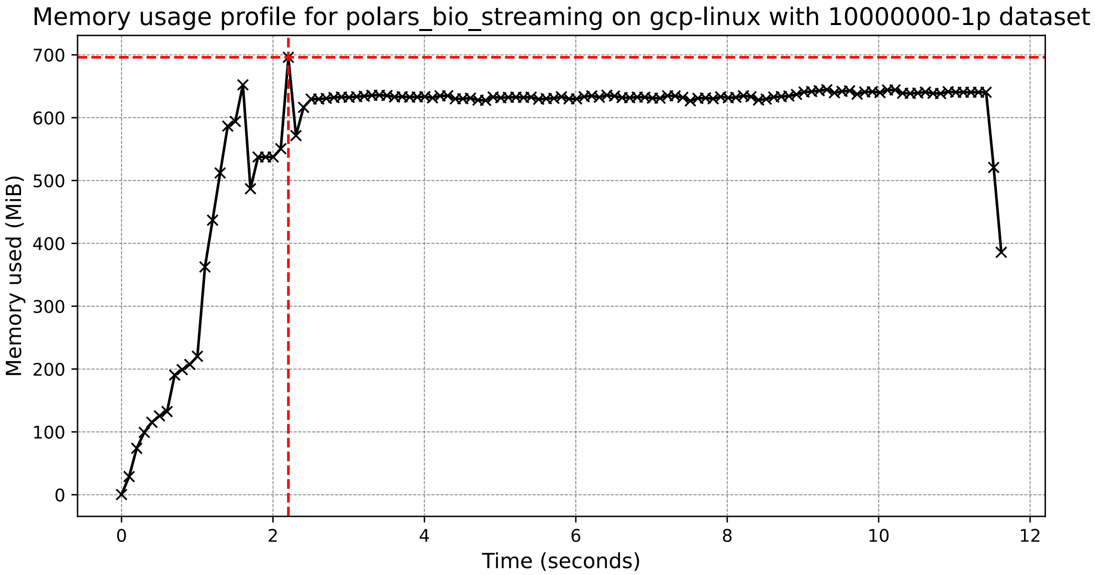

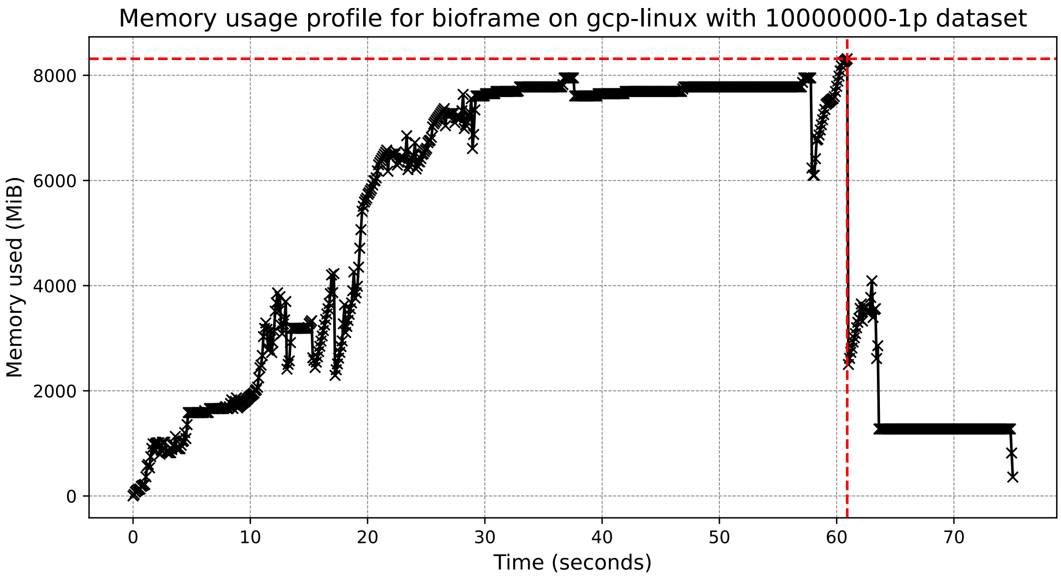


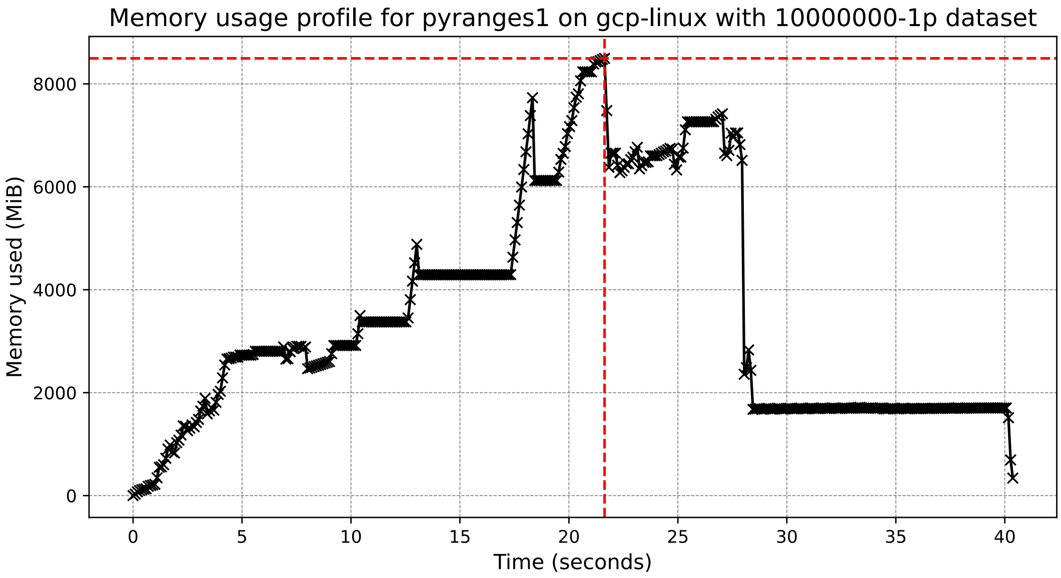


## 3.5 Comparison of the libraries

### 3.5.1 Data processing comparison

The table below compares `polars-bio` with other popular Python libraries for genomic ranges operations.

| Feature | polars-bio | Bioframe | PyRanges0 | PyRanges1 | pybedtools | PyGenomics | GenomicRanges |
|---|---|---|---|---|---|---|---|
| out-of-core processing | **Y** | ✗ | ✗ | ✗ | ✗ | ✗ | ✗ |
| parallel processing | **Y** | ✗ | **Y**[1] | ✗ | ✗ | ✗ | ✗ |

| Feature | polars-bio | Bioframe | PyRanges0 | PyRanges1 | pybedtools | PyGenomics | GenomicRanges |
|---|---|---|---|---|---|---|---|
| vectorized execution engine | **Y** | ✗ | ✗ | ✗ | ✗ | ✗ | ✗ |
| cloud object storage support | **Y** | **Y**/✗[2] | ✗ | ✗ | ✗ | ✗ | **Y** |
| Pandas/Polars DataFrame support | **Y**/**Y** | **Y**/✗ | **Y**/✗[3] | **Y**/✗[4] | ✗/✗ | ✗/✗ | **Y**/**Y** |

[1] PyRanges0 supports parallel processing with Ray, but it does not bring any performance benefits over single-threaded execution and it is not recommended. *Overlap* and *nearest* operations benchmark (1,2,4,6,8 threads) on *ex-rna-vs-ex-anno* on Apple M3 Max platfotm confirms this observation.

| Library | Min (s) | Max (s) | Mean (s) | Speedup |
|---|---|---|---|---|
| pyranges0 | 16.519153 | 17.889156 | 17.118936 | 1.00x |
| pyranges0-2 | 32.539549 | 34.858773 | 33.762477 | 0.51x |
| pyranges0-4 | 30.033927 | 30.367822 | 30.158362 | 0.57x |
| pyranges0-6 | 27.711752 | 33.280867 | 30.089641 | 0.57x |
| pyranges0-8 | 30.049501 | 33.257462 | 31.553328 | 0.54x |

| Library | Min (s) | Max (s) | Mean (s) | Speedup |
|---|---|---|---|---|
| pyranges0 | 1.580677 | 1.703093 | 1.630820 | 1.00x |
| pyranges0-2 | 3.954720 | 4.032619 | 3.997087 | 0.41x |
| pyranges0-4 | 3.716688 | 4.004058 | 3.847917 | 0.42x |
| pyranges0-6 | 3.853526 | 3.942475 | 3.883337 | 0.42x |
| pyranges0-8 | 3.861577 | 3.924950 | 3.902913 | 0.42x |

[2] Some input functions, such as `read_table` support cloud object storage

[3] Only export/import with data copying is supported

[4] RangeFrame class extends Pandas DataFrame

### 3.5.2 API comparison

There is no standard API for genomic ranges operations in Python. This table compares the API of the libraries. The table is not exhaustive and only shows the most common operations used in benchmarking.

Table 130: API comparison for genomic interval operations across Python libraries

| **Op.** | **Bioframe** | **polars-bio** | **PyRanges0** | **PyRanges1** | **Pybedtools** | **GenomicRanges** |
|---|---|---|---|---|---|---|
| overlap | `overlap`[1] | `overlap` | `join`[2] | `join_ranges` | `intersect`[3] | `find_overlaps`[4] |
| nearest | `closest`[5] | `nearest` | `nearest` | `nearest` | `closest`[6] | `nearest`[7] |
| coverage | `coverage`[8] | `coverage` | `count_overlaps`[9] | `count_overlaps`[10] | `coverage`[11] | `count_overlaps`[12] |
| count_overlaps | `count_overlaps`[13] | `count_overlaps` | `count_overlaps` | `count_overlaps` | `intersect`[14] | `count_overlaps` |

### 3.5.3 Schema and data types comparison

`polars-bio` tries to preserve the output schema of the `Bioframe` package, `PyRanges` uses its own internal representation that can be converted to a Pandas dataframe. It is also worth mentioning that `PyRanges0` always uses `int64` for start/end positions representation (*polars-bio* and *Bioframe* and *PyRanges1* determine it adaptively based on the input file formats/DataFrames datatypes used. *polars-bio* does not support interval operations on chromosomes longer than **2Gp**(issue)). However, in the analyzed test case (`ex-rna-vs-ex-anno`) input/output data structures have similar memory requirements. Please compare the following schema and memory size estimates of the input and output DataFrames for `ex-rna-vs-ex-anno` test case:

```
import Bioframe as bf
import polars_bio as pb
import pandas as pd
import polars as pl
import pyranges0 as pr0

DATA_DIR="/Users/mwiewior/research/polars-bio-benchmarking/data/"
df_1 = f"/ex-anno/*.parquet"
df_2 = f"/ex-rna/*.parquet"
df1 = pd.read_parquet(df_1.replace("*.parquet", ""))
df2 = pd.read_parquet(df_2.replace("*.parquet", ""))
cols = ["contig", "pos_start", "pos_end"]

def df2pr0(df):
    return pr0.pyranges(
        chromosomes=df.contig,
        starts=df.pos_start,
        ends=df.pos_end,
    )
```

Input datasets sizes and schemas

```
df1.info()

<class 'pandas.core.frame.DataFrame'>
RangeIndex: 1194285 entries, 0 to 1194284
Data columns (total 3 columns):
```

| # | Column | Non-Null Count | Dtype |
|---|---|---|---|
| 0 | contig | 1194285 non-null | object |

[1] bioframe.ops.overlap

[2] There is an `overlap` method in PyRanges, but its output is only limited to indices of intervals from the other DataFrame that overlap. In Bioframe's benchmark also `join` method instead of overlap was used. See: pyranges.PyRanges.join

[3] `wa` and `wb` options used to obtain a comparable output. See: bedtools intersect

[4] Output contains only a list with the same length as query, containing hits to overlapping indices. Data transformation is required to obtain the same output as in other libraries. Since the performance was far worse than in more efficient libraries anyway, additional data transformation was not included in the benchmark. See: GenomicRanges.find_overlaps

[5] bioframe.ops.closest

[6] `s=first` was used to obtain a comparable output. See: BedTool.closest

[7] `select="arbitrary"` was used to obtain a comparable output. See: GenomicRanges.nearest

[8] bioframe.ops.coverage

[9] PyRanges0 uses `count_overlaps` with parameter `calculate_coverage=False` (default). See: pyranges.PyRanges.count_overlaps

[10] PyRanges1 uses `count_overlaps` with parameter `calculate_coverage=True`. See: pyranges.PyRanges.count_overlaps

[11] `counts=True` parameter used. See: BedTool.coverage

[12] GenomicRanges.count_overlaps

[13] bioframe.ops.count_overlaps

[14] `wa=True` and `c=True` parameters used. See: bedtools intersect

| # | Column | Non-Null Count | Dtype |
|---|---|---|---|
| 1 | pos_start | 1194285 non-null | int32 |
| 2 | pos_end | 1194285 non-null | int32 |

```
dtypes: int32(2), object(1)
memory usage: 18.2+ MB
```

```
df2.info()
```

```
<class 'pandas.core.frame.DataFrame'>
RangeIndex: 9944559 entries, 0 to 9944558
Data columns (total 3 columns):
```

| # | Column | Dtype |
|---|---|---|
| 0 | contig | object |
| 1 | pos_start | int32 |
| 2 | pos_end | int32 |

```
dtypes: int32(2), object(1)
memory usage: 151.7+ MB
```

polars-bio output DataFrames schema and memory used (Polars and Pandas)

```
df_pb = pb.overlap(df_1, df_2, cols1=cols, cols2=cols, use_zero_based=True)
df_pb.count().collect()
```

```
307184634
```

```
df_pb.collect_schema()
```

```
Schema([('contig_1', String),
        ('pos_start_1', Int32),
        ('pos_end_1', Int32),
        ('contig_2', String),
        ('pos_start_2', Int32),
        ('pos_end_2', Int32)])
```

```
df_pb.collect().estimated_size("mb")
```

```
7360.232946395874
```

```
df_pb.collect().to_pandas().info()
```

```
<class 'pandas.core.frame.DataFrame'>
RangeIndex: 307184634 entries, 0 to 307184633
Data columns (total 6 columns):
```

| # | Column | Dtype |
|---|---|---|
| 0 | contig_1 | object |
| 1 | pos_start_1 | int32 |
| 2 | pos_end_1 | int32 |
| 3 | contig_2 | object |

| # | Column | Dtype |
|---|---|---|
| 4 | pos_start_2 | int32 |
| 5 | pos_end_2 | int32 |

```
dtypes: int32(4), object(2)
memory usage: 9.2+ GB
```

Bioframe output DataFrame schema and memory used (Pandas)

```
df_bf = bf.overlap(df1, df2, cols1=cols, cols2=cols, how="inner")
len(df_bf)
```

```
307184634
```

```
df_bf.info()
```

```
<class 'pandas.core.frame.DataFrame'>
RangeIndex: 307184634 entries, 0 to 307184633
Data columns (total 6 columns):
```

| # | Column | Dtype |
|---|---|---|
| 0 | contig | object |
| 1 | pos_start | int32 |
| 2 | pos_end | int32 |
| 3 | contig_ | object |
| 4 | pos_start_ | int32 |
| 5 | pos_end_ | int32 |

```
dtypes: int32(4), object(2)
memory usage: 9.2+ GB
```

PyRanges0 output DataFrame schema and memory used (Pandas)

```
df_pr0_1 = df2pr0(df1)
df_pr0_2 = df2pr0(df2)
```

```
df_pr0_1.df.info()
```

```
<class 'pandas.core.frame.DataFrame'>
RangeIndex: 1194285 entries, 0 to 1194284
Data columns (total 3 columns):
```

| # | Column | Non-Null Count | Dtype |
|---|---|---|---|
| 0 | Chromosome | 1194285 non-null | category |
| 1 | Start | 1194285 non-null | int64 |
| 2 | End | 1194285 non-null | int64 |

```
dtypes: category(1), int64(2)
memory usage: 19.4 MB
```

```
df_pr0_2.df.info()
```

```
<class 'pandas.core.frame.DataFrame'>
```

```
RangeIndex: 9944559 entries, 0 to 9944558
Data columns (total 3 columns):
```

| # | Column | Dtype |
|---|---|---|
| 0 | Chromosome | category |
| 1 | Start | int64 |
| 2 | End | int64 |

```
dtypes: category(1), int64(2)
memory usage: 161.2 MB
```

```
df_pr0 = df_pr0_1.join(df_pr0_2)
len(df_pr0)
```

```
307184634
```

```
df_pr0.df.info()
```

```
<class 'pandas.core.frame.DataFrame'>
RangeIndex: 307184634 entries, 0 to 307184633
Data columns (total 5 columns):
```

| # | Column | Dtype |
|---|---|---|
| 0 | Chromosome | category |
| 1 | Start | int64 |
| 2 | End | int64 |
| 3 | Start_b | int64 |
| 4 | End_b | int64 |

```
dtypes: category(1), int64(4)
memory usage: 9.4 GB
```

Note

Please note that `PyRanges0` unlike `PyRanges1`, *Bioframe* and *polars-bio* returns only one chromosome column.
